# Transcriptional Changes on Blight Fruiting Body of *Flammulina velutipes* Caused by Two New Bacterial Pathogens

**DOI:** 10.3389/fmicb.2019.02845

**Published:** 2019-12-11

**Authors:** Qing Wang, Mengpei Guo, Ruiping Xu, Jingcheng Zhang, Yinbing Bian, Yang Xiao

**Affiliations:** College of Plant Science and Technology, Institute of Applied Mycology, Huazhong Agricultural University, Wuhan, China

**Keywords:** *Flammulina velutipes*, bacteriosis, blight disease, transcriptome, co-expression network

## Abstract

A blight disease of *Flammulina velutipes* was identified with symptoms of growth cessation of young fruiting bodies, short stipe, and brown spots on the pileus. The pathogenic bacteria were identified as *Arthrobacter arilaitensis* and *Pseudomonas yamanorum* by Koch’s postulate, gram staining, morphological and 16S ribosomal RNA gene sequence analyses. Either of the pathogenic bacteria or both of them can cause the same symptoms. Transcriptome changes in blighted *F. velutipes* were investigated between diseased and normal samples. Compared to the control group, 1,099 differentially expressed genes (DEGs) were overlapping in the bacteria-infected groups. The DEGs were significantly enriched in pathways such as xenobiotic metabolism by cytochrome P450 and tyrosine metabolism. Based on weighted correlation network analysis (WGCNA), the module most correlated to the pathogen-treated *F. velutipes* samples and candidate hub genes in the co-regulatory network were identified. Furthermore, a potential diseased mechanism involved in cell wall non-extension, phenolic substrate oxidation, and stress defense response was proposed based on the up-regulation of differentially expressed genes encoding chitin deacetylase, tyrosinase, cytochrome P450, MFS transporter, and clavaminate synthase-like protein. This study provides insights into the underlying reactions of young fruiting body of *F. velutipes* suffering from blight disease and facilitates the understanding of the pathogenic procedure of bacteriosis in edible mushrooms.

## Introduction

*Flammulina velutipes*, also known as winter mushroom or golden needle mushroom, has a delicious taste and high nutrient values. Additionally, it is famous for its abundant content of compounds such as lysine, arginine, and zinc, which are beneficial to children’s intelligence development (Sharma et al., 2009). In East Asia, *F. velutipes* is widely and industrially cultivated on a large scale, with an annual production exceeding 2 million tons in China (Liu et al., 2016), indicating the high popularity of *F. velutipes* in the mushroom market.

However, diseases on fruiting body caused by fungi, bacteria, and viruses greatly limited the development of *F. velutipes* industry. For example, web cob disease caused by fungus *Cladobotryum varium*, red rust disease caused by fungus *Acrostalagmus luteo-albus*, brown discolored disease caused by *F. velutipes* browning virus (FvBV) were occasionally reported (Kim et al., 1999; Magae and Sunagawa, 2010; Zhang and Tang, 2014). Furthermore, bacteria should be mainly responsible for fruiting body diseases of *F. velutipes*. Black rot disease caused by *Pseudomonas tolaasii*, brown rot disease caused by *Ewingella americana*, yellow sticky disease caused by *Cedecea neteri*, pink disease caused by *Erwinia persicin*, tumor disease caused by *Ochrobactrum pseudogrignonense* have resulted in serious losses in *F. velutipes* production and quality, or even threatened human food safety (Han et al., 2012; Wu et al., 2015; Liu et al., 2018; Yan et al., 2019a,b). Thus, researches on disease-causing factors and disease-caused changes on needle mushroom are imperative.

Bacteria are also the major pathogens of other mushrooms; especially *Pseudomonas* spp., *P. fluorescent*, *P. putida*, *P. reactans*, and *P. tolaasii* often cause brown blotch disease or soft rot disease on fruiting body of mushrooms, including *Agaricus bisporus*, *Pleurotus* spp. (Russo et al., 2003; Zhang et al., 2007; Cantore and Iacobellis, 2014; Wang et al., 2016). Besides bacterial pathogens of *F. velutipes* and *Pseudomonas* spp. listed above, other bacteria such as *Pantoea beijingensis*, *Achromobacter xylosoxidans*, *Cryptococcus pseudolongus* were also reported to be linked with soft rot disease of *Pleurotus nebrodensis*, stipe rot disease of *Coprinus comatus*, and brown rot disease of *Lentinula edodes*, respectively (Xu et al., 2014; Kwon et al., 2016; Ye et al., 2018).

Most studies on mushroom disease focused on the identification and diversity of pathogen and less on the disease management and pathogenic mechanism. In only a few studies involving pathogenic mechanism, transcriptomic analysis has partially revealed the interaction mechanisms between fungus *Lecanicillium fungicola* and *A. bisporus* and between mushroom virus X (MVX) and *A. bisporus* (Bailey et al., 2013; Eastwood et al., 2015). Bacterium damaged to fungus often *via* producing an antibiotic compound, competition for limiting nutrients or a mycophagous manner such as causing hyphal damage and release of fungal contents (Mela et al., 2011). Fungus is able to recognize bacterial Microbe-Associated Molecular Patterns (MAMPs) and initiate rapid transcription responses or other innate immune response at early infection stage (Gkarmiri et al., 2015; Ipcho et al., 2016). For instance, in *A. bisporus*, *P. tolaasii* produced a lipodepsipeptide toxin called tolaasin and induced tyrosinase mRNA activation of host (Soler-Rivas et al., 2001). However, to our knowledge, there was no research on how mushroom fruiting body responds to bacterium infecting the transcriptional profile during symptomatic phase, which will contribute to clarify the physiological and biochemical states of mushroom fruiting bodies so as to guide the treatment of diseases.

Transcriptome is usually used to explain the interactions between fungal mycelia and bacterium. The lipopeptides of *Bacillus subtilis* can inhibit microsclerotia formation in *Verticillium dahlia* and the Gene Ontology (GO) analysis revealed significant enrichment of the genes associated with response to stress, cellular metabolic processes, and translation (Yu et al., 2018). In addition, the lipopeptides can inhibit the expression of genes related to secondary metabolism, protein catabolism, and the high-osmolarity glycerol (HOG) signaling pathway (Yu et al., 2018). In a non-contact co-inoculation confrontation assay between *Aspergillus niger* mycelia and bacterium *Collimonas fungivorans*, differentially expressed genes (DEGs) of *A. niger* included those involved in lipid, cell wall metabolism and cell defense (Mela et al., 2011). When *Fusarium graminearum* mycelia were exposed to bacterial MAMPs, a core set of genes were identified to be related to energy generation, transport, amino acid production, and secondary metabolism, especially to iron uptake (Ipcho et al., 2016). The genes induced upon the co-cultivation of *Coprinopsis cinerea* mycelia with *B. subtilis* and *Escherichia coli* tended to mainly encode secreted peptides and proteins with predicted antibacterial activities (Kombrink et al., 2019). Based on omics technologies, the weighted correlation network analysis (WGCNA) facilitates the analysis of highly multivariate and complex data (DiLeo et al., 2011). Clusters of highly correlated genes with high correlation coefficients were defined as modules. WGCNA can also be employed to construct gene networks in which each node represents a gene and the connecting lines (edges) between genes represent co-expression correlations. Genes that showed the most interconnections in the network were identified as hub genes, as indicated by their high connectivity value (Li et al., 2019). For instance, universal cadmium-responsive genes were identified by co-expression network analysis of the transcriptomes of rice roots exposed to various cadmium stresses (Tan et al., 2017). The genes and signaling pathways adaptively responsive to varied adverse stresses were identified in the insect fungal pathogen, *Beauveria bassiana* by comparative transcriptome and gene co-expression network analysis (He et al., 2018).

In the present study, morphological and molecular analyses were applied to identify the pathogens of a new bacterial disease termed as young fruiting body blight disease in *F. velutipes*. Moreover, transcriptome sequencing combined with WGCNA was used to uncover the vital gene module related to the diseased samples and establish a gene regulatory network. Lastly, a hypothetical mechanism model for the blight disease of young fruiting body was developed as well.

## Materials and Methods

### Disease Symptom Observation and Pathogen Isolation

In 2017, cultivation bottles of *F. velutipes* strain R6x2 with unusual growth were found in Wuhan Ruyiqing Mushroom High-tech Co. Ltd. (Wuhan, China). The symptoms including the phenotype of stipe and pileus could be observed visually. After rinsing the surface of the diseased fruiting bodies with distilled water, diseased tissues were scrubbed with 75% alcohol, followed by washing three times in sterile water, crushing in a 1.5 ml centrifuge tube containing 1 ml of sterile water, and diluting the initial suspension to a concentration of 10^−6^. Subsequently, 100 μl of the diluted suspensions were inoculated to Luria-Bertani (LB) medium (Tryptone 10 g/L, Yeast extract 5 g/L, NaCl 10 g/L, pH 7.0) plates in triplicate, followed by incubation in a constant-temperature incubator at 28°C. After 24 h, the bacterial colonies with different morphologies were transferred to new LB medium plates for further cultivation using the scribing method until pure culture was obtained.

### Verification of Pathogenicity

Briefly, the pathogenicity of single bacterial isolates was verified. The purified single colony was transplanted into liquid LB medium and shaken at 28°C for 24 h, followed by diluting the bacterial suspension to a concentration of 10^8^ CFU (Colony Forming Units) ml^−1^ as the inoculum for treatment groups. For further experiments, 16 bottles of *F. velutipes* in the initial young fruiting body period were prepared for each group, followed by spraying 5 ml of inoculum liquid onto the surface of the young fruiting bodies in each bottle. The control group was treated with 5 ml of sterile water. All the treatment groups were cultured at 15°C with an air relative humidity of at least 95%, and growth was recorded by visual examination for each group every 12 h. The pathogenicity of the mixed bacteria was evaluated by treating *F. velutipes* with suspension blended with 50% of each pathogenic bacterium.

### Phenotypic Characterization of Pathogens

The morphological characteristics of pathogens were analyzed in shape, diameter, elevation, margin, surface appearance, density, consistency, and color. Single colonies were subjected to gram staining and microscopic observation at a 100-fold objective lens magnification. The images were recorded and analyzed using Image-Pro Express 6.0 supplemented in the BX51 microscope (Olympus, Japan).

### Identification of Pathogenic Bacteria by *16S rRNA* Gene

Genomic DNA of the pathogenic bacteria was extracted as previously reported (Wilson, 2001). The primers used for 16S rRNA gene sequence amplification were 27F (5′-AGAGTTTGATCCTGGCTCAG-3′) and 1492R (5′-TACGGCTACCTTGTTACGACTT-3′) (Ye et al., 2018). The procedure of sequence amplification included pre-denaturation at 95°C for 5 min, 34 cycles of denaturation at 95°C for 30 s, annealing at 55°C for 30 s, extension at 72°C for 1 min and 30 s, and hold at 72°C for 10 min. The amplified product was purified by PCR purification kit (Sangon Biotech Co., Ltd., ShangHai, China), and cloned using the pClone 007 simple vector kit (Tsingke Biotech Co., Ltd., Beijing, China). The full-length *16S rRNA* gene was sequenced by ABI3730XL at Tianyi Huiyuan Biology Company (Wuhan, China). Blast was utilized to search homologous sequences of our *16S rRNA* gene, and the sequencing data were uploaded to the GenBank with the accession numbers MK346198 and MK346199. The phylogenetic trees were constructed using the neighbor-joining (NJ) method by MEGA7 (Kumar et al., 2016), with 1,000 bootstrap replications.

### Transcriptome Sequencing

Samples were collected on the outbreak of blight disease of young fruiting body in *F. velutipes* after bacterial inoculation, without any culture media residues included. Briefly, a 5 g sample of young fruiting bodies was collected from one bottle for RNA-seq. Samples from three different bottles were defined as three biological repeats for each group. All the samples were frozen in liquid nitrogen and immediately stored at −80°C for RNA extraction. Total RNA was extracted from *F. velutipes* samples using Trizol method (Rio et al., 2010). The cDNA library was established using NEBNext^®^ UltraTM II RNA Library Prep Kit (New England Biolabs, Inc., USA). Illumina X-Ten sequencing platform was used to sequence the *F. velutipes* samples. No less than 4 GB of clean data were obtained for each sample. RNA extraction, library construction, and sequencing were performed by Wuhan Genoseq Technology Co., Ltd.

### Transcriptome Analysis

Quality of raw sequencing reads was determined by the GC content, Q20 and Q30 values. Raw RNA-seq data were all uploaded to the GenBank with the accession number of PRJNA526334. Clean reads were trimmed using Trimmomatic-0.33 (Bolger et al., 2014) to filter low-quality reads. Hisat2 (Kim et al., 2015) was used to map clean reads to *F. velutipes* reference genome (Park et al., 2014), and duplicate reads were removed by Picard,^1^ followed by estimating the mapping rate by qualimap (Garcia-Alcalde et al., 2012). The mapped clean reads count was evaluated by HTseq (Anders et al., 2015) and converted to Transcripts Per Million (TPM) data through TBtools (Chen et al., 2018). EdgeR (Chen et al., 2014) was used to screen differentially expressed genes (DEGs) with |log2 (fold change)| >1 and false discovery rate (FDR) < 0.01. DEGs were annotated by Blast2GO (Conesa et al., 2005). All the genes were mapped to the seqdb database by BLAST (McGinnis and Madden, 2004), and their GO IDs were obtained by gene2go (Chawla and Kuiper, 2016). GO enrichment analysis was conducted using Fisher’s test and corrected by FDR <0.01. Kyoto Encyclopedia of Genes and Genomes (KEGG) pathway enrichment analysis was performed by KOBAS (Wu et al., 2006). GO and KEGG data were visualized by ggplot2^2^.

### Gene Co-expression Analysis

Genes with a maximum expression value <5 and a coefficient variation <0.2 from all expressed genes were filtered out, and the remaining genes were analyzed for WGCNA. An appropriate soft threshold power (soft power = 14) was selected by the pick Soft Threshold function of the R Package (Langfelder and Horvath, 2008), which could make the scale-free topology index greater than 0.8. WGCNA algorithm was used to construct gene modules and evaluate the connectivity of genes in a module. In our study, the eigengene value was calculated for each module and used to test the association with samples in each group. *F. velutipes* samples inoculated by sterile water, FvB1, FvB2, and their mixture were considered as different sample traits. Our targeted trait is the combination of *F. velutipes* samples in the three pathogens-treated groups. After the gene module with the highest relationship to the targeted trait was screened out, the overlapping DEGs in the three treated groups versus the control group were selected from the gene module to construct the co-expression network by Cytoscape (Kohl et al., 2011). DEGs with highest connectivity value were hub nodes in the network and their surrounding nodes were required to meet the following two conditions: (1) they are co-expressed with hub genes and differentially expressed in the three treated groups compared to the control group; (2) the weight value of the edge between the surrounding node and the hub node was higher than 0.1.

### Validation of Transcriptome Data by Quantitative Real Time PCR

To validate the transcriptome sequencing results, five genes were randomly selected for qRT-PCR analysis using SYBR select Master Mix (ABI, USA) with glyceraldehyde-3-phosphate dehydrogenase (GAPDH) as the internal control gene. cDNAs were reverse transcribed from 1 μg of RNA using RevertAid First Strand cDNA Synthesis Kit (Thermo Scientific, USA). qRT-PCR analysis was performed on CFX Connect Real-Time System (BIO-RAD, USA). Each qRT-PCR experiment was conducted in triplicate. The relative expression of genes was evaluated by the 2^−△△CT^ method. The information for each gene and primer is shown in Supplementary Table S1.

## Results

### Disease Symptoms and Pathogen Isolation

**Figure 1:**
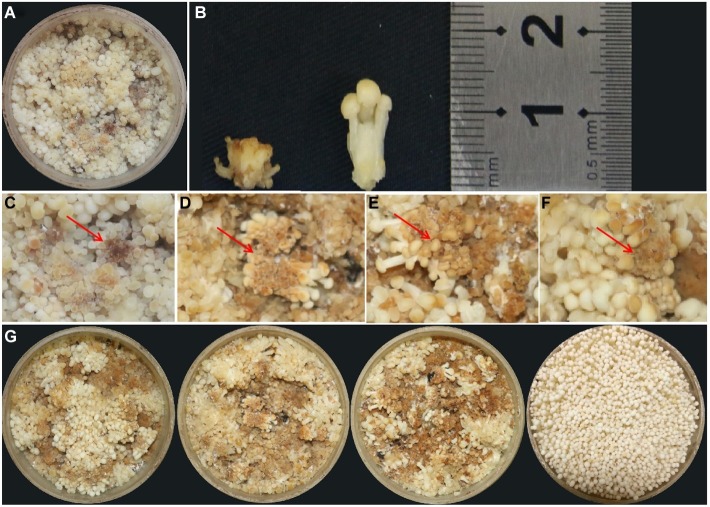
Observation of young fruiting body blight disease of *F. velutipes*. **(A)** Typical symptoms of blight disease of *F. velutipes* by natural infection. **(B)** Height comparison between diseased (left) and normal (right) fruiting body. The stipe height of the diseased *F. velutipes* was much shorter than that of the normal one. **(C)** Brown spots occurred on partial pileus. Red arrows refer to the brown pileus in diseased *F. velutipes*. **(D–F)** Similar symptoms of diseased *F. velutipes* in the three treated groups after 48 h of bacterial inoculation. They were inoculated by bacterial suspension of FvB1 **(D)**, FvB2 **(E)**, and mixture of FvB1 and FvB2 **(F)**, respectively. **(G)** Overall view of diseased *F. velutipes* in the three treated groups (three on the left), and normal one in the control group inoculated by sterile water (right).

Blight disease occurred on the young fruiting body of *F. velutipes*, leading to growth cessation with short stipe and brown spots on the pileus (Figures 1A–C). The diseased fruiting bodies are unable to grow into normal ones and need to be eliminated during cultivation, thus causing serious production losses. Our investigation revealed that the blight disease occurs commonly and frequently in the mushroom factories, especially under the conditions of high temperature and humidity. Once the disease broke out, all *F. velutipes* in the production room failed to grow or develop. From the diseased tissue, three pure cultured bacteria with different morphologies were ultimately isolated and named as FvB1, FvB2, and FvB3, respectively.

### Verification of Bacterial Pathogenicity

In the test of single bacterial pathogenicity, samples in FvB1- or FvB2-treated groups could induce young fruiting body blight disease, but not the samples in the FvB3-treated group (Supplementary Figure S1). The bacteria in diseased *F. velutipes* tissues were isolated and cultured again. The morphological characteristics and *16S rRNA* sequences of re-isolated bacteria are consistent with those of the original FvB1 and FvB2 (data not shown). Therefore, the two isolates (FvB1 and FvB2) were verified as the pathogens of blight disease of *F. velutipes* by the Koch’s rule.

Based on FvB1 and FvB2, the groups termed as S1, S2, and S3 were set up. In S1 and S2, *F. velutipes* was inoculated with FvB1 and FvB2, respectively. In the S3 group, *F. velutipes* was inoculated with bacterial suspension mixed with 50% FvB1 and 50% FvB2. After 48 h of inoculation, all the treatment groups were diseased, with consistent symptoms observed in natural infection (Figures 1D–G). The results showed that either FvB1 or FvB2, or their mixture, can cause young fruiting body blight disease in *F. velutipes.*

### Morphological Characteristics of Pathogens

**Figure 2:**
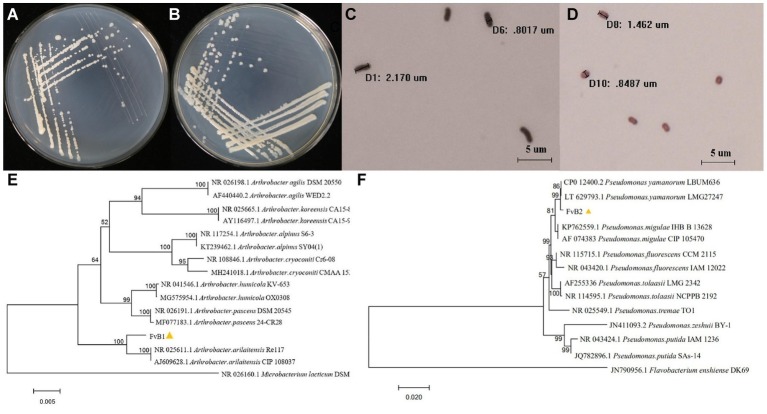
**(A–D)** Morphological characteristics of pathogens. The bacterial pathogens FvB1 **(A)** and FvB2 **(B)** streaked to single colonies on LB medium after 24 h of cultivation. Microscopic characteristics of bacteria FvB1 **(C)** and FvB2 **(D)** were observed under a 100-fold objective lens after gram staining. The microscopic size of each bacterium was determined and is displayed on the pictures. **(E,F)** 16S rRNA gene phylogenetic trees of pathogens. **(E)** Phylogenetic tree was constructed using the neighbor-joining method for comparison of the *16S rRNA* sequences of strain FvB1 and members of the *Arthrobacter* group from database. The bar represents a phylogenetic distance of 0.5%. *Microbacterium lacticum* strain DSM 20427 was used as outer group. **(F)** Phylogenetic tree was constructed using the neighbor-joining method for comparison of the *16S rRNA* sequences of strain FvB2 and members of the *Pseudomonas* group from database. The bar represents a phylogenetic distance of 2%. *Flavobacterium* enshiense strain DK69 was used as outer group.

After 24 h of inoculation, multiple single colonies arose on the surface of each plate. The morphological features of these two bacteria are shown in Table 1 and Figures 2A–D. FvB1 was gram-positive, with an irregular rod-like shape. FvB2 was gram-negative, with a short rod-like structure.

**Table 1:** Morphological characteristics of two pathogens FvB1 and FvB2.

Characteristics	FvB1	FvB2
Single colony diameter (mm)	1–2	≈3
Shape	Circle	Circle
Elevation	Flat	Little prominent
Margin	Neat	Sawtooth
Color	Milky yellow	Milky white
Surface appearance	Smooth	Smooth
Density	Opacity	Translucence
Consistency	Soft	Have mucus
Gram staining	Purple, positive	Red, negative
Shape under 100-fold lens	Irregular rods	Short rod-like

### Identification of Pathogens by *16S rRNA* Gene Sequence Analysis

The phylogenetic tree of FvB1 *16S rRNA* gene is shown in Figure 2E. It can be seen that standard strains of one species were clustered as a branch, and different species were located in different branches with a certain distance. FvB1 was clustered in a branch with the strains of *A. arilaitensis*, Re117 and CIP108037, supported by a bootstrap value of 100%. Additionally, the *16S rRNA* gene sequence of FvB1 and *A. arilaitensis* was more than 97% identical with each other, thus FvB1 was identified to be *A. arilaitensis*. In a similar way, FvB2 was clustered with *P. yamanorum* in a high bootstrap fraction (99%) and a similarity higher than 97% (Figure 2F), thus identified to be *P. yamanorum*.

### RNA Sequencing Data

In the three treated groups and the control group, the GC content is about 53%, and the Q20 and Q30 values of the sequencing data are more than 98 and 96% for all the samples, indicating that the sequencing data can be used for further analysis. After filtering raw reads from each group using Trimmomatic-0.33, the clean reads were collected and the mapping rates were calculated to be about 78% by qualimap2 (Supplementary Table S2).

### Screening of Differentially Expressed Genes

**Figure 3:**
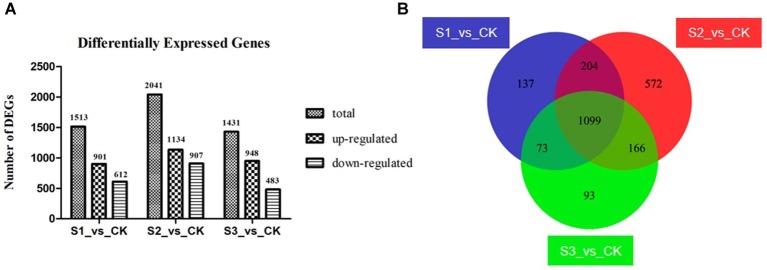
Differently expressed genes (DEGs) in the three bacteria-treated groups compared to the control group. **(A)** Information of up- and down-regulated DEGs. **(B)** Venn diagram of DEGs. Groups S1_vs._CK, S2_vs._CK, and S3_vs._CK respectively represent comparison between control group and FvB1-, FvB2-, and mixture of FvB1- and FvB2-infected groups. There are 1,099 shared DEGs in the three bacteria-treated groups compared with the control group.

The gene expression value in the three replicates of each treated group was determined to be more than 0.9 by Pearson’s correlation coefficient analysis (Supplementary Figure S2), illustrating the reliability of the replicates of samples. Compared with the control group, 1,513; 2,041; and 1,431 DEGs were identified in groups S1, S2, and S3, respectively (Figure 3A). Additionally, 1,099 DEGs were overlapping in the three treated groups (Figure 3B), with 714 of them being up-regulated and 385 of them down-regulated (Supplementary Tables S3, S4).

### Functional Annotation of Differentially Expressed Genes

**Figure 4:**
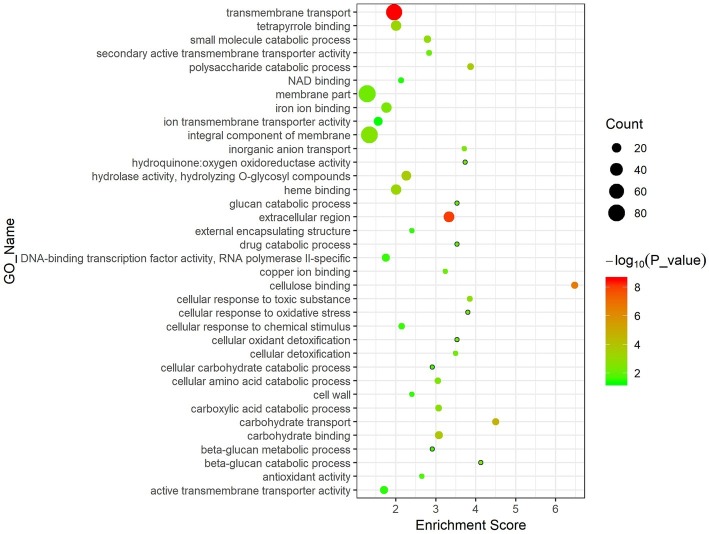
Bubble chart of GO enrichment analysis of the 1,099 DEGs shared in the three treated groups. The abscissa represents enrichment score and the ordinate represents different GO terms. Circle size represents the gene number while circle color represents the value of −log10 (*p*).

Results of GO enrichment analysis can illustrate the gene functional changes in diseased *F. velutipes*. The 1,099 DEGs shared in the three treated groups were mainly enriched in the following GO terms (Figure 4). In the Biological Process (BP) section, DEGs were enriched in the basic metabolism process such as transmembrane transport, carbohydrate metabolic process, oxidation–reduction process, and polysaccharide catabolic process. Moreover, they were also enriched in the GO terms related to *F. velutipes* defense response, such as cellular response to toxic substance, cellular detoxification, and cellular response to oxidative stress. In the Molecular Function (MF) section, they were enriched in the GO terms of fundamental gene molecular function, such as oxidoreductase activity, hydrolase activity, and transmembrane transporter activity. The GO term of antioxidant activity was also present in the MF section. For the cellular component (CC) section, the major GO terms were involved in extracellular region, integral component of membrane and cell wall.

### Co-expression Network

**Figure 5:**
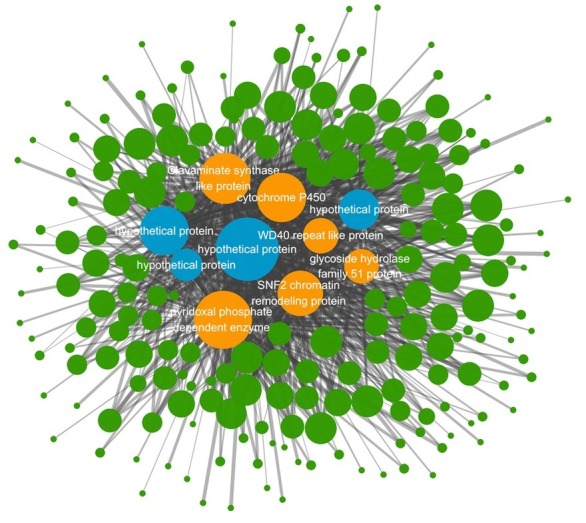
Co-expression network diagram from the MEblue gene module. The circle nodes represent genes and the straight lines represent edges. Node size stands for the connectivity value of genes and the width of straight line stands for weight value. Four blue nodes represent hub genes annotated as hypothetical proteins and six orange nodes represent other hub genes annotated as certain functional genes.

According to the clustering analysis result in WGCNA process, 13 different gene modules were obtained and distinguished by different colors (Supplementary Figure S3). The blue gene module named MEblue was analyzed to be the one superlatively correlated to the targeted trait representing blight samples in the three treated groups. The Pearson’s correlation coefficient reached 0.87 and the value of *p* was 2e-04 (Supplementary Figure S4). There are 797 genes in the MEblue module based on WGCNA of all the expressed genes, and 256 of them were differentially expressed in the three treated groups compared to the control group (Supplementary Table S5). Among the 256 DEGs, top 10 genes with highest connectivity value were chosen as hub nodes in the regulatory network (Table 2). A total of 208 hub-surrounded nodes were co-expressed with the 10 hub genes and a total of 859 edges with a weight value of over 0.1 between nodes were presented in the regulatory network (Figure 5, Supplementary Table S6). The 10 candidate hub genes encoded a glycoside hydrolase family 51 protein, a SNF2 chromatin remodeling protein, a cytochrome P450, a WD40 repeat-like protein, a clavaminate synthase-like protein, a pyridoxal phosphate-dependent enzyme, and four hypothetical proteins.

**Table 2:** Information of the 10 hub genes with highest connectivity value in the MEblue gene module.

Gene ID	Gene annotation	Connectivity value
chr08_AA_01027	hypothetical protein CYLTODRAFT_357890 [*Cylindrobasidium torrendii* FP15055 ss-10]	291.42
chr11_AA_01033	glycoside hydrolase family 51 protein [*Cylindrobasidium torrendii* FP15055 ss-10]	290.52
chr10_AA_00693	hypothetical protein ARMSODRAFT_947720 [*Armillaria solidipes*]	288.78
chr09_AA_00241	SNF2 chromatin remodeling protein [*Armillaria solidipes*]	288.25
chr08_AA_00858	cytochrome P450 [Cylindrobasidium torrendii FP15055 ss-10]	287.71
chr11_AA_00724	WD40 repeat-like protein [*Armillaria solidipes*]	287.32
chr02_AA_00171	hypothetical protein CYLTODRAFT_425697 [*Cylindrobasidium torrendii* FP15055 ss-10]	284.74
chr08_AA_00687	Clavaminate synthase-like protein [*Armillaria solidipes*]	281.87
chr11_AA_01711	hypothetical protein ARMGADRAFT_1046867 [*Armillaria gallica*]	281.10
chr06_AA_00738	pyridoxal phosphate-dependent enzyme, beta subunit [*Cylindrobasidium torrendii* FP15055 ss-10]	280.84

### Differentially Expressed Genes and Pathways Related to Responses to Bacterial Infection in Diseased *Flammulina velutipes*

In the 256 hub DEGs, 128 genes were annotated as hypothetical proteins and the remaining 128 genes were annotated as certain functional genes. Based on their expression and functional annotation, 19 typical genes were picked out to indicate the responses of *F. velutipes* infected by pathogenic bacteria (Supplementary Table S7).

Genes related to fungal cell wall chitin and glucan hydrolyzation were differentially expressed in the treated groups. The gene chr11_AA_00595 encoding chitin deacetylase was significantly up-regulated in the three treated groups, and so were the three genes (chr09_AA_00190, chr09_AA_00191, and chr06_AA_00433) encoding exo-beta-1,3-glucanase (Supplementary Table S7).

**Figure 6:**
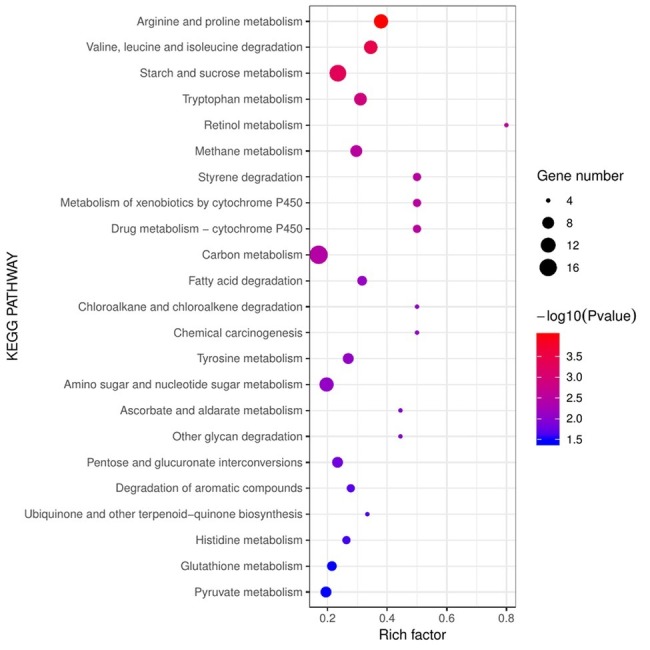
Bubble chart of KEGG enrichment analysis of the 1,099 DEGs shared in the three treated groups. The abscissa represents rich factor and the ordinate represents different KEGG pathways. Circle size represents the gene number while circle color represents the value of −log10 (*p*). Rich factor represents the rate of DEGs to background genes in the reference genome.

Pathways of stress defense were significantly enriched in diseased *F. velutipes* (Figure 6), including xenobiotics by cytochrome P450, drug metabolism-cytochrome P450, and glutathione metabolism. Six genes encoding cytochrome P450 were all up-regulated in groups S1, S2, and S3, among which chr08_AA_00858 was a hub gene. The gene chr08_AA_00018 encoding glutathione S-transferase was also induced (Supplementary Table S7). In the pathway of xenobiotics by cytochrome P450, cytochrome P450 catalyzed a series of xenobiotics such as benzpyrene, naphthalene, and aflatoxin B1, while glutathione S-transferase catalyzed the subsequent reactions (Supplementary Figure S5A). Additionally, glutathione S-transferase encoding genes were also enriched in the pathways of drug metabolism-cytochrome P450 and glutathione metabolism, which participated in the reaction process of cyclophosphamide to 4-Glutathionyl-CPA and glutathione to mercapturic acid, respectively (Supplementary Figures S5B,C). Besides, there are other stress defense-related genes in the MEblue gene module. Hub gene chr08_AA_00687 encoding clavaminate synthase-like protein was up-regulated in the diseased *F. velutipes*. Five genes encoding MFS general substrate transporter and the gene chr08_AA_01259 encoding Aldo/keto reductase were all up-regulated (Supplementary Table S7).

For the co-expression relationships of the above-mentioned genes, hub gene chr08_AA_00858 (encoding cytochrome P450) was co-expressed with genes chr11_AA_00504 (encoding MFS transporter) and chr08_AA_01259 (encoding Aldo/keto reductase) with a weight value of 0.27 and 0.24, respectively. This indicated that the potential stress defense process was co-regulated by polygenes. Additionally, hub gene chr08_AA_00687 (encoding clavaminate synthase-like protein) was co-expressed with gene chr09_AA_00191 (encoding exo-beta-1,3-glucanase) with a weight value of 0.15. This suggested that the biological process of stress defense was correlated with the process of cell wall glucan hydrolyzation in some way, thus regulating diseased *F. velutipes* symptoms together.

Tyrosinase metabolism related to melanin biosynthesis pathway was also enriched. Gene chr10_AA_00996 encoding tyrosinase (Supplementary Table S7) played an important role in this pathway. Tyrosinase catalyzed the substrate of tyrosine to produce dopaquinone and finally led to the synthesis of eumelanin and pheomelanin, both of which were related to melanin biosynthesis. In the present study, the reaction of melanin biosynthesis was exacerbated by the up-regulation of tyrosinase (Supplementary Figure S5D).

### Validation of RNA-seq Data by RT-qPCR

Five candidate DEGs were selected for validation experiments by RT-qPCR, including chr03_AA_00311 (encoding a glycerol-3-phosphate dehydrogenase), chr06_AA_00234 (encoding a putative lectin), chr07_AA_00645 (encoding aldehyde dehydrogenase), chr09_AA_01114 (encoding glutathione S-transferase), and chr09_AA_00190 (encoding a potassium/sodium eff). Changes in the expression levels of the five genes were in line with the results of RNA-seq, suggesting the accuracy of the transcriptome sequencing in this study (Supplementary Figure S6).

## Discussion

### Two Different Bacterial Pathogens Causing *Flammulina velutipes* Young Fruiting Body Blight Disease

This is the first report on the *F. velutipes* blight disease in the period of young fruiting body. In the mushroom house, cultivation bottles were frequently observed to be infected with this blight disease and needed to be picked out. Owing to the huge scale and continuity of industrial cultivation, it is difficult to control the occurrence and spread of the young fruiting body disease, which resulted in serious yield reduction. In addition, once the pathogenic bacteria spread onto the surface of harvestable *F. velutipes*, the potential food safety risk increased.

*Arthrobacter* strains are widely distributed in various environments such as soil, water, and air, and their functions focus on degradation of organic pollutants (Kim et al., 2008) and resistance to heavy metals (Bafana et al., 2010). *A. cumminsii* is the most frequently encountered *Arthrobacter* species in human clinical specimens (Funke et al., 1996). *Arthrobacter* species was isolated and identified to cause brown blotch in *A. bisporus* (Bessette, 1984). *A. arilaitensis,* a novel species isolated from the surface of cheeses (Irlinger et al., 2005), is able to adapt to the environment of cheese surface through catabolizing substrates such as lactic acid, lipids, and amino acids (Monnet et al., 2010). Members of the genus *Pseudomonas* play a pivotal part in pathogenesis of edible mushrooms and often cause rot diseases in the stipe and pileus of fruiting body. *P. agarici* was reported to cause a bacterial disease called drippy gill affecting the fruiting body of cultivated mushroom (Young, 1970). *P. tolaasii* and *P. gingeri* were reported to be responsible respectively for the brown blotch disease (Godfrey et al., 2001) and the ginger blotch disease of *A. bisporus* (Wong et al., 2008). This study uncovered a bacterium of genus *Arthrobacter* responsible for the blight disease of edible fungi and a new member in genus *Pseudomonas* that is pathogenic to *F. velutipes*, thus enriching the knowledge of bacterial diseases in edible mushrooms.

In this study, the two bacteria, *A. arilaitensis* and *P. yamanorum*, were identified to be pathogenic to *F. velutipes*. Interestingly, the disease symptoms were almost the same on the young fruiting bodies when infected by either of the two pathogens or both of them. Previous studies indicated that different bacteria caused the same disease symptoms in edible mushrooms. *Pantoea beijingensis* sp. nov. and *Pseudomonas putida* were reported to cause water-soaked lesions and soft rot in the fruiting body of *Pleurotus eryngii* (Liu et al., 2013; Wang et al., 2016). Besides *P. tolaasii*, *Burkholderia gladioli* pv. *agaricicola* was described to cause soft rot symptoms of *A. bitorquis* (Lincoln et al., 1991; Gill and Tsuneda, 1997). Additionally, *Serratia liquefiiciens* and *Cedecea davisae* were reported to result in yellow blotch on *A. bisporus* (Sivanesan, 2003).

Most DEGs were overlapping in the three treated groups compared to the control group, which is consistent with the high similarity of disease symptoms in the treated groups. The genes of *C. cinerea* induced upon co-cultivation with either *B. subtilis* or *E. coli* were highly overlapping, suggesting that the fungus used a similar arsenal of effectors against gram-positive and gram-negative bacteria (Kombrink et al., 2019). *A. arilaitensis* and *P. yamanorum* are gram-positive and gram-negative, respectively. In our study, the majority of DEGs were overlapping in the different bacteria-infected groups compared to the control group, possibly indicating that *F. velutipes* also utilizes a highly similar mechanism in response to different bacterial infections.

### A Potential Disease Mechanism

**Figure 7:**
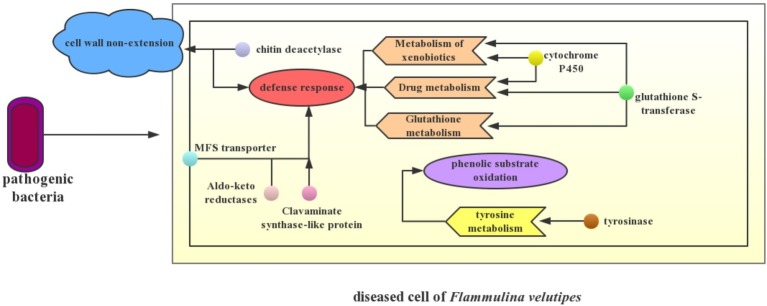
Potential disease mechanism model. The simple interaction between pathogenic bacteria and *F. velutipes* is presented in the schematic. Small circles with different colors in the fungal cell represent different DEGs. The arrow refers to the possible biological process that is mainly manipulated by these DEGs. When the pathogenic bacteria infected *F. velutipes* young fruiting bodies, up-regulation of genes encoding chitin deacetylase in the diseased cells not only leads to non-extension of stipe cell wall, but also enhances self-defense of the cell. Up-regulation of genes encoding MFS transporter, aldo-keto reductases, and clavaminate synthase-like protein primarily contributes to the defense response to pathogen infection. Enriched pathways of xenobiotic metabolism, drug metabolism, and glutathione metabolism including genes encoding cytochrome P450 and glutathione transferase demonstrate the stress resistance of *F. velutipes* after bacterial infection as well. Additionally, up-regulation of tyrosinase encoded gene triggers significant changes of tyrosine metabolism, thus causing synthesis of melanin that involves in brown spots occurrence.

Transcriptome changes can provide important evidence for phenotype changes in mushroom diseases. Pileus spotting disease of *A. bisporus* is caused by *L. fungicola*. Transcriptome analysis identified a significantly up-regulated DAG11 gene that is linked to the autolysis or host cell death occurring in the pileus with spot lesion symptom (Bailey et al., 2013). In *A. bisporus* showing brown pileus symptom infected by mushroom virus X, transcriptome analysis also identified a serine protease gene that involved in melanin biosynthesis and the formation of brown tissue, and a ribosomal protein gene that corresponded to the delay in primordium formation (Eastwood et al., 2015). Here, a potential disease mechanism model was developed to explain the symptoms of young fruiting body blight disease of *F. velutipes* based on the MEblue gene module and other pivotal DEGs or pathways (Figure 7), which were involved in cell wall non-extension, phenolic substrate oxidation, and stress defense.

Stipe non-elongation in bacteria-infected *F. velutipes* may be correlated to the up-regulation of chitin deacetylation of cell wall. Stipe elongation is an important process of fruiting body development in Basidiomycetes, and it is primarily resulted from manifold cell elongation instead of cell division (Kamada and Takemaru, 1977; Zhou et al., 2019). Further, the cell elongation requires cell wall extension to create space for the enlarging protoplast (Kang et al., 2019). Chitin and glucan, two dominating components of fungal cell wall, are hydrolyzed by chitinase and glucanase respectively. Cell wall extension is induced by the disruption of bonding between glucan chains or chitin chains (Fang et al., 2014). Chitin deacetylase catalyzes the nascent chitin component to generate more soluble chitosan. Chitin deacetylase functions synergistically with chitin synthase to construct the main structure of fungal cell wall, significantly contributing to its integrity (Wang et al., 2018b). The partial deacetylation of chitin by chitin deacetylases is usually considered to confer chitin resistance to hydrolysis by chitinases for self-defense and ability for coping with stress (Christodoulidou et al., 1996). In this study, genes encoding chitin deacetylase were significantly up-regulated after bacterial infection in *F. velutipes*, while three chitinase genes, chr07_AA_00553, chr09_AA_00036, and chr11_AA_00508 (|log2 (fold change)| >1), were found to be significantly down-regulated using Fisher’s test and corrected by FDR <0.05. It can be inferred that chitin was deacetylased to prevent the chitin microfibrils of cell wall from being hydrolyzed. On the one hand, changes of these two kinds of genes enhanced the cell wall integrity and defense of *F. velutipes* against pathogenic bacteria. On the other hand, up-regulation of chitin deacetylation and down-regulation of hydrolyzation of chitin microfibrils link bonds caused the non-extension of the cell wall, finally causing stipe non-elongation in *F. velutipes*.

Cell wall hydrolyzation by β-1,3-glucanases and chitinases often plays a major role in cell wall softening in yeast and filamentous fungi (Bowman and Free, 2006; Kang et al., 2019). Three genes encoding β-1-3-glucanase were up-regulated in the bacteria-infected *F. velutipes*, indicating that cell wall rigidity was damaged. Under the co-regulation of various genes including chitin deacetylase, chitinase, and β-1-3-glucanase, infected *F. velutipes* stipe presented the non-elongation phenotype in the end.

In the KEGG analysis, tyrosine metabolism pathway was significantly enriched. The up-regulation of tyrosinase-encoding gene possibly intensified the biosynthesis of melanin. Tyrosinase catalyzed phenolic compounds such as tyrosine to generate quinones, which undergo further non-enzymatic reactions leading to dark melanin pigments (Weijn et al., 2013). Further, tyrosine was regarded as one of the melanin precursors causing the browning of fruiting body of *A. bisporus* (Soler-Rivas et al., 1999). Phenolic substrates of the active tyrosinase were oxidized, proportional to the damage detectable on the mushroom pileus (Soler-Rivas et al., 1998). In addition, melanin biosynthesis was also reported to relate to resistance of *A. bisporus* to pathogens including *P. tolaasii* and *L. fungicola*, as toxic melanins circumscribe the growth of the pathogens (Foulongne-Oriol et al., 2012). In the young fruiting body of diseased *F. velutipes*, melanin synthesis derived from tyrosine oxidation by tyrosinase caused brown spot of pileus, as well as participated in defense of *F. velutipes* to pathogenic bacteria.

Stress defense played a pivotal role in response of *F. velutipes* to pathogenic bacteria. Genes encoding cytochrome P450 may serviceably function in defense response of *F. velutipes* to pathogenic bacteria. In addition, cytochrome P450 was closely related to the xenobiotic metabolism (Schalk et al., 1997). Up-regulation of cytochrome P450-encoding genes indicated that stipes of young fruiting body still tried to keep growing when *F. velutipes* suffered bacterial infection.

MFS general substrate transporter, a kind of membrane transporter related to the efflux of impurity substances, may be involved in the export of antibiotics produced by *C. cinerea* or antifungal compounds produced by bacteria out of the fungal cell (Coleman and Mylonakis, 2009; Kombrink et al., 2019). It may be important for self-defense (Wang et al., 2018a). Research also reported the complex roles of MFS transporters for indirect regulation of the stress response machinery and control of membrane potential and/or internal pH (Dos et al., 2014). Therefore, it can be inferred that the up-regulation of MFS transporter-encoding genes played pivotal roles in reducing toxin accumulation in blighted *F. velutipes* and stress defense.

In addition, genes potentially involved in *F. velutipes* defense response to pathogenic bacteria included genes encoding clavaminate synthase-like protein, Aldo/keto reductase, and glutathione S-transferase. The gene encoding clavaminate synthase-like protein could be involved in secondary metabolite production and catalyze the production of antibiotic compounds (Lundén et al., 2015). Aldo-keto reductases detoxify reactive aldehydes formed from exogenous toxicants, such as aflatoxin and endogenous toxicants as well as those formed from the breakdown of lipid peroxides. They are stress-regulated genes and play a central role in the cellular response to osmotic, electrophilic, and oxidative stress (Jin and Penning, 2007). Glutathione S-transferase genes were of high inducibility under a wide range of stress conditions, including biotic stress, and they are identified to be involved in the detoxification of toxic substances by their conjugation with glutathione, the attenuation of oxidative stress, and participation in hormone transport (Gullner et al., 2018). Glutathione and tryptophan metabolisms play crucial roles in *Arabidopsis* immunity during the hypersensitive response to hemibiotrophs by terminating the invasive growth of both non-adapted and adapted hemibiotrophs (Hiruma et al., 2013). In general, various stress defense-related genes were co-expressed to regulate the self-defense process of *F. velutipes* to the pathogenic bacteria.

When *F. velutipes* was infected by two different bacteria and their mixture, genes related to cell wall non-extension, melanin synthesis, and stress defense were differentially expressed in a similar way. Co-occurrence of the three processes resulted in similar symptoms of diseased *F. velutipes* in the three treated groups.

## Conclusion

In this study, we report for the first time the young fruiting body blight disease of *F. velutipes* and identify two bacteria (*A. arilaitensis* and *P. yamanorum*) to be the pathogenic bacteria for the disease. The potential disease mechanism was revealed through analyses of transcriptome sequencing and co-expression network, which also explain the similar symptoms of *F. velutipes* induced by either of the two pathogens or both of them. The vital genes affected by the bacteria included those encoding chitin deacetylase, cytochrome P450, clavaminate synthase-like protein, glutathione S-transferase, MFS transporter, tyrosinase, etc. A disease model involved in cell wall non-extension, phenolic substrate oxidation, and stress defense response was also developed here. In the future, the functions of the pivotal genes can be further verified by over-expression, RNAi and gene editing. Our study provided some reference information on *F. velutipes* bacterial disease and interactions between edible fungi and pathogenic bacteria. Study on stress response of *F. velutipes* to pathogenic bacteria may help identify genes resistant to bacteria, thus facilitating breeding resistant varieties in this mushroom.

## Data Availability Statement

The datasets generated for this study can be found in the NCBI, MK346198, MK346199, PRJNA526334.

## Author Contributions

YX and YB conceived and designed the experiments. QW performed the experiments and wrote the paper. MG, RX, and JZ contributed to modify and polish the paper.

### Conflict of Interest

The authors declare that the research was conducted in the absence of any commercial or financial relationships that could be construed as a potential conflict of interest.

## Footnotes

^1^http://broadinstitute.github.io/picard/

^2^http://had.co.nz/ggplot2/
