# Supplementary material for: Transcriptional Changes on Blight Fruiting Body of Flammulina velutipes Caused by Two New Bacterial Pathogens
Source: Front Microbiol. 2019 Dec 11;10:2845. doi: 10.3389/fmicb.2019.02845 (PMC6917577; doi:10.3389/fmicb.2019.02845)
Supplement: Supplementary file 1 [file Data_Sheet_1.docx]

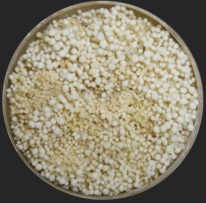


Supplementary Figure S1. Growth situation of *F.velutipes* after inoculation of bacterium FvB3.


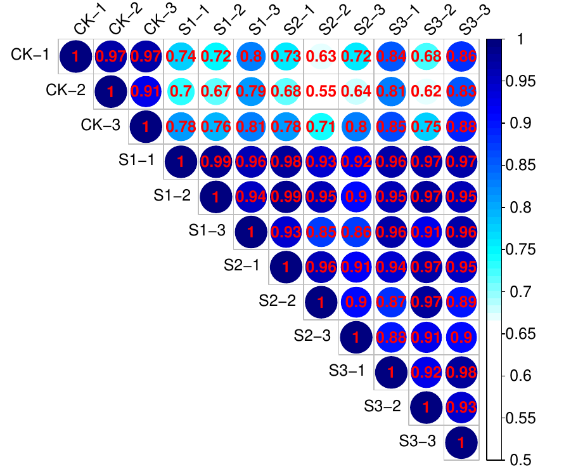


Supplementary Figure S2. Pearson correlation coefficient of gene expression between 12 samples. Control Check (CK) represents control group CK and S1, S2, S3 represent three treated groups S1, S2 and S3. -1, -2, -3 denote the three repeats in each group. The ruler color and nearby numbers indicate the Pearson correlation coefficient. Values in the circles represent specific Pearson correlation coefficient between each two samples.


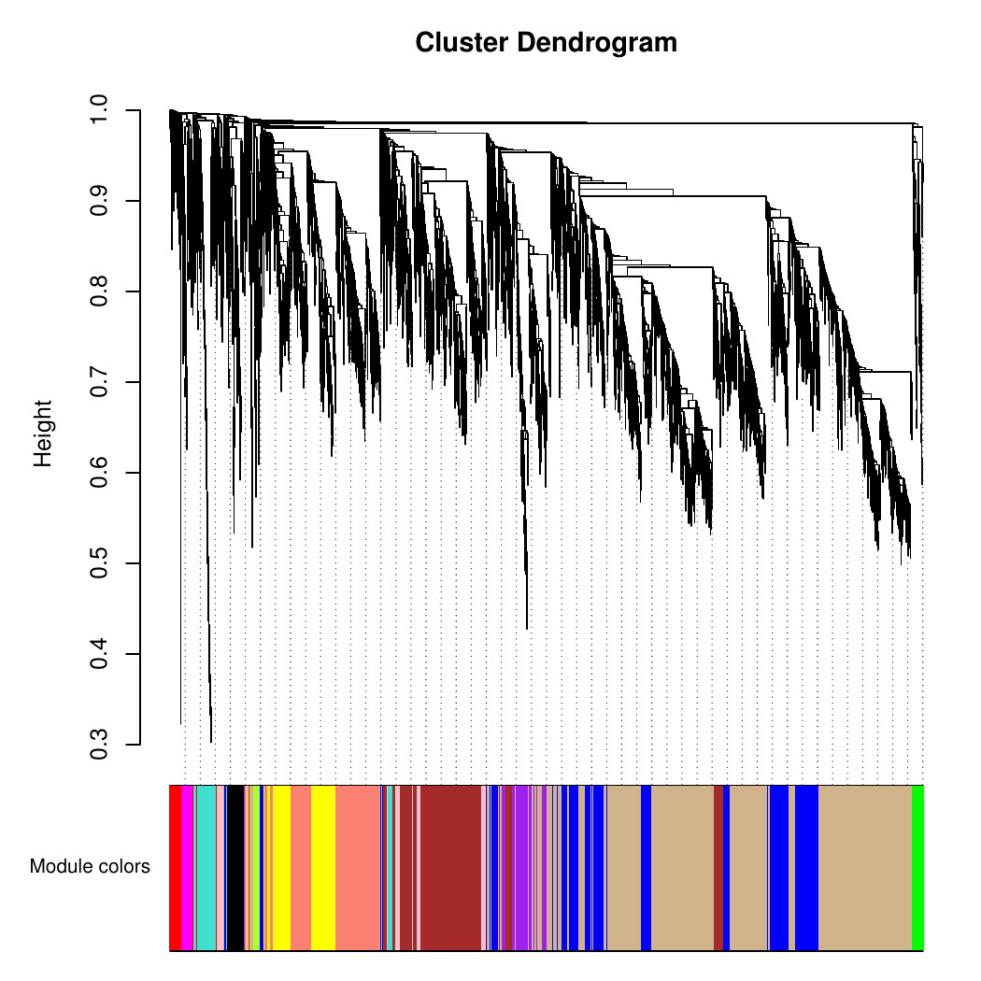


Supplementary Figure S3. Clustering dendrogram of genes, with dissimilarity based on topological overlap, together with assigned module colors. There are 13 different colored blocks shown in the bottom representing 13 different modules, with the color legend same as that in Fig. S4. The block size indicates the gene quantity in every module.


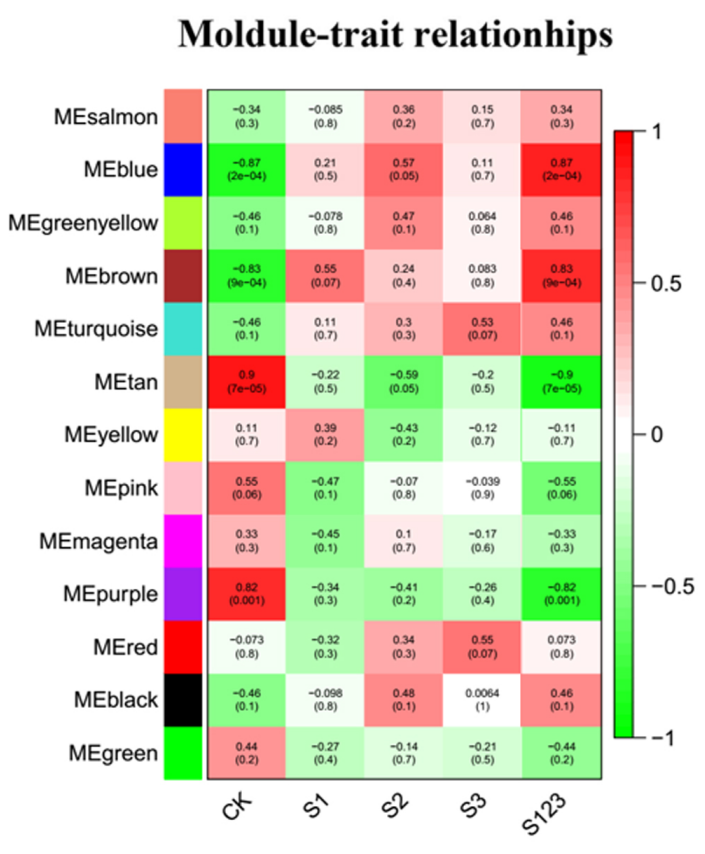


Supplementary Figure S4. Module-trait relationships. Each row corresponds to a gene module, column to a trait. CK, S1, S2, S3 respectively represent samples in groups CK, S1, S2 and S3. S123 indicate the combination of samples in groups S1, S2 and S3. The colored blocks on the left indicate 13 gene modules and colors of the rule on the right indicates Pearson correlation coefficient. Each cell contains the corresponding correlation coefficient and the P value.


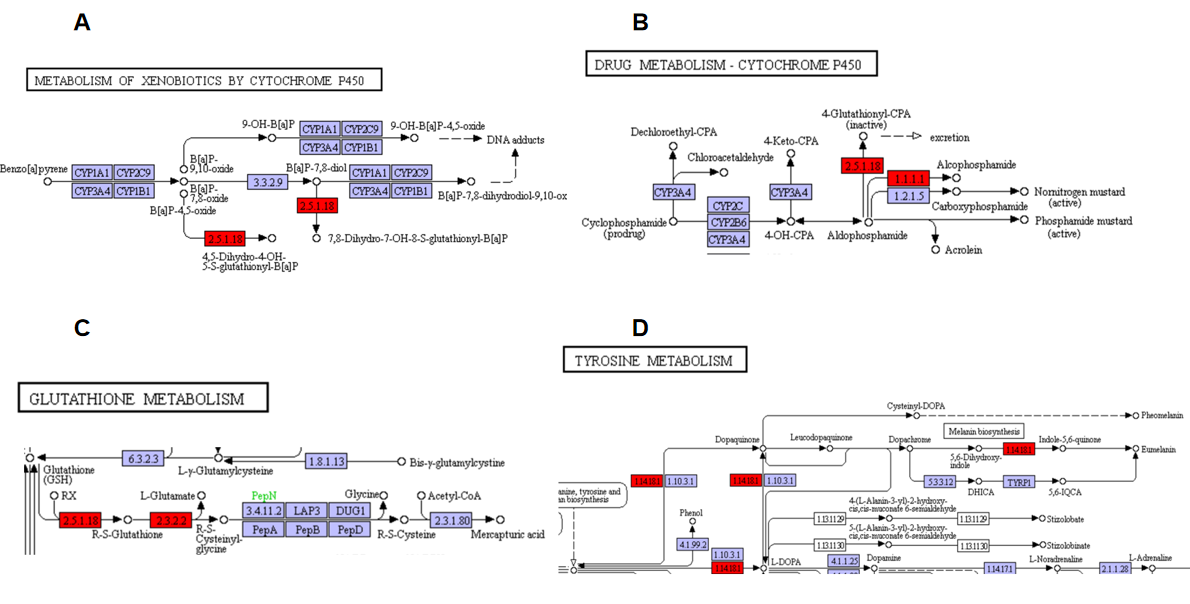


Supplementary Figure S5. Four enriched pathways including Metabolism of xenobiotics by cytochrome P450 (A), Drug metabolism - cytochrome P450 (B), Glutathione metabolism (C) and Tyrosine metablism (D). Red rectangles represent up-regulated genes, among which enzyme ID 1.14.18.1 represents tyrosinase and ID 2.5.1.18 represents glutathione S-transferase.


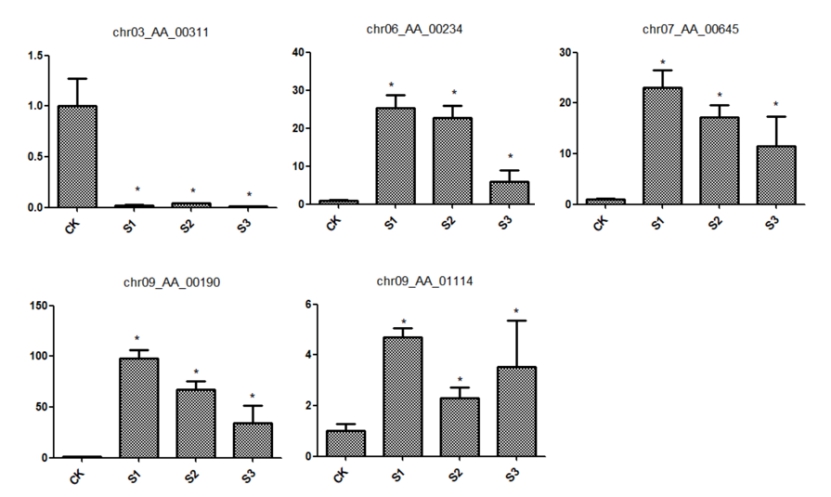


Supplementary Figure S6. The RT-qPCR results of the five selected DEGs. The horizontal axis represents different groups, including CK, S1, S2 and S3. The vertical axis represents expression folds. Gene expression levels in the CK group were regarded as one. * represents significance of difference.

Supplementary Table S1. Information and primers used for RT-qPCR of selected differentially expression genes.

| Gene ID | Gene annotation | Forward primer(5’-3’) | Reverse primer(5’-3’) |
| --- | --- | --- | --- |
| chr03_AA_00311 | glycerol-3-phosphate dehydrogenase | TGGCCAGACAAAGAAAACGG | AAGACTGCGTGCCATAAAGC |
| chr06_AA_00234 | putative lectin | TCAGGCGAGCTTTGCATTTG | ATGTGGCTGATGTTCTTGCG |
| chr07_AA_00645 | aldehyde dehydrogenase | TGTGCTTTCTGCCACCAAAC | TGTGGCGCGATTTCAGATAC |
| chr09_AA_01114 | glutathione S-transferase | CCCGCAATTGCATATGGTGG | CCTTTGCCAACAAGAGCACC |
| chr09_AA_00190 | potassium/sodium eff | AAGGCCGTCGCATTTTCATC | AAGGCCGTCGCATTTTCATC |
| GAPDH* | Glyceraldehyde phosphatedehydrogenase | TTCCACTGCCACCCAAAAGAC | GTAGGGACACGGAAAGCGAGA |

* represents the internal control gene

Supplementary Table S2. Information of RNA-seq data for the 12 samples.

| Sample IDs | GC(%) | Q20(%) | Q30(%) | Mapping rate |
| --- | --- | --- | --- | --- |
| CK-1  CK-2  CK-3  S1-1  S1-2  S1-3  S2-1  S2-2  S2-3  S3-1  S3-2  S3-3 | 53.22  53.32  53.26  53.23  53.24  53.50  53.24  53.22  53.40  53.21  53.21  53.13 | 99.15  99.16  99.15  99.11  99.10  98.99  99.06  99.09  99.10  99.11  99.12  99.12 | 97.11  97.14  97.11  96.99  96.98  96.64  96.86  96.93  96.97  97.01  97.02  97.03 | 78.33%  80.58%  78.22%  79.37%  79.53%  80.24%  79.65%  78.66%  79.35%  78.18%  79.39%  78.02% |

CK represents control group, and S1, S2, S3 represent three treated groups. -1, -2, -3 represent the three repeats in each group.

Supplementary Table S3. Information of 714 up-regulated genes shared in the three treated groups S1, S2 and S3 compared to CK group.

| Gene ID | Annotation | Log2 (fold change) |
| --- | --- | --- |
| chr07_AA_00048 | glycoside hydrolase family 61 protein [Moniliophthora roreri MCA 2997] &gt;KTB29261.1 hypothetical protein WG66_18149 [Moniliophthora roreri] | 8.23 |
| chr11_AA_01130 | putative carotenoid ester lipase precursor [Flammulina velutipes] | 8.09 |
| chr07_AA_00167 | hypothetical protein ARMSODRAFT_956944 [Armillaria solidipes] &gt;SJL10924.1 probable transporter (major facilitator superfamily) [Armillaria ostoyae] | 7.11 |
| chr08_AA_00195 | hypothetical protein CVT24_008519 [Panaeolus cyanescens] | 6.83 |
| chr11_AA_01429 | NA | 6.78 |
| chr09_AA_01190 | potassium/sodium eff [Cylindrobasidium torrendii FP15055 ss-10] | 6.78 |
| chr10_AA_00944 | general substrate transporter [Cylindrobasidium torrendii FP15055 ss-10] | 6.77 |
| chr06_AA_00234 | putative lectin [Flammulina velutipes] | 6.70 |
| chr03_AA_00126 | hypothetical protein CYLTODRAFT_412931 [Cylindrobasidium torrendii FP15055 ss-10] | 6.64 |
| chr09_AA_00717 | chitin deacetylase [Flammulina velutipes] | 6.58 |
| chr01_AA_00442 | hypothetical protein ARMGADRAFT_970088 [Armillaria gallica] | 6.52 |
| chr02_AA_00139 | related to putative tartrate transporter [Armillaria ostoyae] | 6.46 |
| chr10_AA_01322 | putative laccase 5 [Flammulina velutipes] | 6.37 |
| chr11_AA_01419 | hypothetical protein GYMLUDRAFT_246259 [Gymnopus luxurians FD-317 M1] | 6.35 |
| chr01_AA_00233 | related to D-arabinitol 2-dehydrogenase [Armillaria ostoyae] | 6.30 |
| chr09_AA_00051 | GMC oxidoreductase [Cylindrobasidium torrendii FP15055 ss-10] | 6.26 |
| chr10_AA_00718 | PHB depolymerase family esterase [Rhizoctonia solani] | 6.04 |
| chr06_AA_00433 | putative ectomycorrhiza-upregulated exo-beta-1,3-glucanase GH5 [Flammulina velutipes] | 6.02 |
| chr06_AA_00666 | hypothetical protein [Flammulina velutipes] | 6.02 |
| chr08_AA_00019 | glycoside hydrolase family 7 [Flammulina velutipes] | 6.00 |
| chr01_AA_00428 | putative protein lysine methyltransferase [Moniliophthora roreri MCA 2997] | 5.94 |
| chr10_AA_00761 | NA | 5.89 |
| chr09_AA_00955 | putative endo-1,4-beta-xylanase precursor [Flammulina velutipes] | 5.82 |
| chr01_AA_00232 | NAD(P)-binding protein [Armillaria gallica] | 5.82 |
| chr01_AA_00444 | APC amino acid permease [Cylindrobasidium torrendii FP15055 ss-10] | 5.70 |
| chr11_AA_00208 | NA | 5.69 |
| chr02_AA_00108 | asparaginase [Flammulina velutipes] | 5.65 |
| chr10_AA_00286 | B-(1-6) glucan synthase [Meira miltonrushii] &gt;PWN37625.1 B-(1-6) glucan synthase [Meira miltonrushii] | 5.62 |
| chr05_AA_00535 | putative endoglucanase II [Flammulina velutipes] | 5.60 |
| chr11_AA_00581 | laccase [Flammulina velutipes] | 5.46 |
| chr08_AA_00633 | Carbonic anhydrase 2 [Hypsizygus marmoreus] | 5.45 |
| chr09_AA_00719 | hypothetical protein PLEOSDRAFT_1093927 [Pleurotus ostreatus PC15] | 5.44 |
| chr10_AA_00915 | jacalin-related lectin [Flammulina velutipes] | 5.39 |
| chr09_AA_00052 | GMC oxidoreductase [Cylindrobasidium torrendii FP15055 ss-10] | 5.37 |
| chr11_AA_01423 | hypothetical protein ARMSODRAFT_549990 [Armillaria solidipes] | 5.34 |
| chr07_AA_00617 | NAD-dependent formate dehydrogenase [Moniliophthora perniciosa] | 5.33 |
| chr09_AA_00100 | hypothetical protein K503DRAFT_238266 [Rhizopogon vinicolor AM-OR11-026] | 5.32 |
| chr09_AA_00299 | NA | 5.32 |
| chr03_AA_00073 | NA | 5.31 |
| chr11_AA_01678 | sodium:inorganic phosphate symporter [Cylindrobasidium torrendii FP15055 ss-10] | 5.24 |
| chr09_AA_00314 | NA | 5.22 |
| chr09_AA_00190 | putative exo-beta-1,3-glucanase [Flammulina velutipes] | 5.18 |
| chr11_AA_00499 | GroES-like protein [Armillaria solidipes] | 5.17 |
| chr10_AA_01446 | NA | 5.12 |
| chr07_AA_00195 | copper radical oxidase [Cylindrobasidium torrendii FP15055 ss-10] | 5.07 |
| chr07_AA_00601 | hypothetical protein GYMLUDRAFT_71620 [Gymnopus luxurians FD-317 M1] | 5.07 |
| chr07_AA_00567 | hypothetical protein CYLTODRAFT_406012 [Cylindrobasidium torrendii FP15055 ss-10] | 5.05 |
| chr02_AA_00114 | glycoside hydrolase family 128 protein [Cylindrobasidium torrendii FP15055 ss-10] | 4.97 |
| chr10_AA_01458 | NA | 4.97 |
| chr06_AA_00926 | hypothetical protein CYLTODRAFT_377216 [Cylindrobasidium torrendii FP15055 ss-10] | 4.96 |
| chr08_AA_00363 | uncharacterized protein ARMOST_09751 [Armillaria ostoyae] | 4.94 |
| chr05_AA_00241 | hypothetical protein CYLTODRAFT_421179 [Cylindrobasidium torrendii FP15055 ss-10] | 4.91 |
| chr09_AA_00269 | glycoside hydrolase family 11 enzyme [uncultured eukaryote] | 4.86 |
| chr06_AA_00925 | hypothetical protein CYLTODRAFT_377216 [Cylindrobasidium torrendii FP15055 ss-10] | 4.78 |
| chr06_AA_00694 | Zinc-regulated transporter 1 [Hypsizygus marmoreus] | 4.77 |
| chr11_AA_01685 | 1-aminocyclopropane-1-carboxylate deaminase [Armillaria solidipes] | 4.77 |
| chr09_AA_00191 | exo-beta-1,3-glucanase [Armillaria solidipes] | 4.71 |
| chr09_AA_00739 | glycoside hydrolase family 7 protein [Cylindrobasidium torrendii FP15055 ss-10] | 4.70 |
| chr11_AA_01046 | hypothetical protein CYLTODRAFT_428160 [Cylindrobasidium torrendii FP15055 ss-10] | 4.64 |
| chr06_AA_00205 | uncharacterized protein ARMOST_06736 [Armillaria ostoyae] | 4.62 |
| chr10_AA_01440 | NA | 4.61 |
| chr09_AA_00831 | hypothetical protein CYLTODRAFT_417837 [Cylindrobasidium torrendii FP15055 ss-10] | 4.56 |
| chr09_AA_00461 | hypothetical protein CYLTODRAFT_436819 [Cylindrobasidium torrendii FP15055 ss-10] | 4.54 |
| chr10_AA_00243 | predicted protein [Mycena chlorophos] | 4.54 |
| chr06_AA_00968 | uncharacterized protein ARMOST_04336 [Armillaria ostoyae] | 4.54 |
| chr06_AA_00920 | hypothetical protein AMATHDRAFT_60193 [Amanita thiersii Skay4041] | 4.50 |
| chr03_AA_00465 | hypothetical protein ARMGADRAFT_1081249 [Armillaria gallica] | 4.48 |
| chr09_AA_00892 | hypothetical protein CYLTODRAFT_431446 [Cylindrobasidium torrendii FP15055 ss-10] | 4.46 |
| chr08_AA_00348 | NA | 4.40 |
| chr03_AA_00325 | hypothetical protein CYLTODRAFT_421760 [Cylindrobasidium torrendii FP15055 ss-10] | 4.39 |
| chr10_AA_00999 | hypothetical protein ARMSODRAFT_1019459 [Armillaria solidipes] | 4.33 |
| chr08_AA_00984 | cytochrome P450 monooxygenase pc-bph [Armillaria gallica] | 4.32 |
| chr03_AA_00127 | uncharacterized protein ARMOST_21085 [Armillaria ostoyae] | 4.31 |
| chr09_AA_01045 | glycoside hydrolase family 5 protein [Peniophora sp. CONT] | 4.29 |
| chr04_AA_00484 | hypothetical protein CYLTODRAFT_422976 [Cylindrobasidium torrendii FP15055 ss-10] | 4.28 |
| chr09_AA_00451 | NA | 4.26 |
| chr11_AA_01097 | glycoside hydrolase family 3 protein [Cylindrobasidium torrendii FP15055 ss-10] | 4.25 |
| chr11_AA_01422 | uncharacterized protein ARMOST_18856 [Armillaria ostoyae] | 4.24 |
| chr09_AA_00772 | hypothetical protein LENED_002377 [Lentinula edodes] | 4.24 |
| chr07_AA_00156 | uncharacterized protein ARMOST_00326 [Armillaria ostoyae] | 4.23 |
| chr09_AA_01099 | general substrate transporter [Armillaria gallica] | 4.19 |
| chr10_AA_00579 | hypothetical protein HETIRDRAFT_480361 [Heterobasidion irregulare TC 32-1] &gt;ETW76290.1 hypothetical protein HETIRDRAFT_480361 [Heterobasidion irregulare TC 32-1] | 4.17 |
| chr09_AA_00126 | glycoside hydrolase family 6 protein [Cylindrobasidium torrendii FP15055 ss-10] | 4.17 |
| chr04_AA_00132 | NA | 4.16 |
| chr11_AA_01002 | related to dehydrogenase [Armillaria ostoyae] | 4.13 |
| chr10_AA_01157 | glycoside hydrolase family 53 protein [Cylindrobasidium torrendii FP15055 ss-10] | 4.10 |
| chr10_AA_00728 | lytic polysaccharide monooxygenase [Cylindrobasidium torrendii FP15055 ss-10] | 4.08 |
| chr03_AA_00294 | hypothetical protein CYLTODRAFT_489528 [Cylindrobasidium torrendii FP15055 ss-10] | 4.07 |
| chr10_AA_01490 | hypothetical protein WG66_19536 [Moniliophthora roreri] | 4.07 |
| chr03_AA_00185 | hypothetical protein GYMLUDRAFT_251306 [Gymnopus luxurians FD-317 M1] | 4.01 |
| chr05_AA_00370 | hypothetical protein GALMADRAFT_90776 [Galerina marginata CBS 339.88] | 4.00 |
| chr11_AA_01635 | glycoside hydrolase family 7 [Flammulina velutipes] | 3.99 |
| chr11_AA_00965 | hypothetical protein CYLTODRAFT_346255 [Cylindrobasidium torrendii FP15055 ss-10] | 3.98 |
| chr11_AA_00094 | homogentisate 1,2-dioxygenase [Armillaria solidipes] | 3.97 |
| chr01_AA_00090 | uncharacterized protein ARMOST_15826 [Armillaria ostoyae] | 3.96 |
| chr03_AA_00186 | hypothetical protein Hypma_011894 [Hypsizygus marmoreus] | 3.93 |
| chr11_AA_01645 | 4-O-methyl-glucuronoyl methylesterase [Hypsizygus marmoreus] | 3.91 |
| chr07_AA_00919 | Six-hairpin glycosidase [Cylindrobasidium torrendii FP15055 ss-10] | 3.91 |
| chr05_AA_00278 | NA | 3.88 |
| chr10_AA_01475 | hypothetical protein CYLTODRAFT_380528 [Cylindrobasidium torrendii FP15055 ss-10] | 3.87 |
| chr03_AA_00302 | hypothetical protein CYLTODRAFT_424064 [Cylindrobasidium torrendii FP15055 ss-10] | 3.81 |
| chr07_AA_00467 | NA | 3.79 |
| chr08_AA_01046 | MFS general substrate transporter [Armillaria gallica] | 3.78 |
| chr04_AA_00141 | hypothetical protein CYLTODRAFT_422578 [Cylindrobasidium torrendii FP15055 ss-10] | 3.78 |
| chr09_AA_00799 | glycoside hydrolase family 16 protein [Cylindrobasidium torrendii FP15055 ss-10] | 3.77 |
| chr02_AA_00313 | NA | 3.74 |
| chr01_AA_00310 | hypothetical protein GYMLUDRAFT_150603 [Gymnopus luxurians FD-317 M1] | 3.70 |
| chr05_AA_00327 | isocitrate lyase [Cylindrobasidium torrendii FP15055 ss-10] | 3.70 |
| chr05_AA_00590 | hydrophobin [Flammulina velutipes] | 3.68 |
| chr11_AA_01487 | hypothetical protein CYLTODRAFT_383512 [Cylindrobasidium torrendii FP15055 ss-10] | 3.68 |
| chr04_AA_00751 | NA | 3.68 |
| chr01_AA_00109 | hydrophobin [Flammulina velutipes] | 3.68 |
| chr07_AA_00116 | iron reductase [Cylindrobasidium torrendii FP15055 ss-10] | 3.67 |
| chr09_AA_00271 | Endo-1,4-beta-xylanase 6 [Hypsizygus marmoreus] | 3.67 |
| chr10_AA_00969 | NAD(P)-binding protein [Armillaria gallica] | 3.66 |
| chr09_AA_00101 | hypothetical protein CYLTODRAFT_375620 [Cylindrobasidium torrendii FP15055 ss-10] | 3.66 |
| chr03_AA_00112 | NA | 3.65 |
| chr07_AA_00829 | general substrate transporter [Cylindrobasidium torrendii FP15055 ss-10] | 3.64 |
| chr06_AA_01031 | glycoside hydrolase family 43 protein [Periconia macrospinosa] | 3.61 |
| chr10_AA_00647 | hypothetical protein ARMSODRAFT_1082975 [Armillaria solidipes] | 3.60 |
| chr01_AA_00530 | NA | 3.56 |
| chr05_AA_00347 | potassium/sodium eff [Auricularia subglabra TFB-10046 SS5] | 3.54 |
| chr06_AA_01035 | NA | 3.54 |
| chr11_AA_01298 | hypothetical protein CVT25_009203 [Psilocybe cyanescens] | 3.53 |
| chr10_AA_01474 | hypothetical protein ARMGADRAFT_1052929 [Armillaria gallica] | 3.52 |
| chr05_AA_00394 | NA | 3.52 |
| chr11_AA_00595 | chitin deacetylase [Armillaria gallica] | 3.51 |
| chr07_AA_00804 | uncharacterized protein ARMOST_19843 [Armillaria ostoyae] | 3.51 |
| chr09_AA_00721 | chitin deacetylase [Flammulina velutipes] | 3.50 |
| chr09_AA_01224 | uncharacterized protein ARMOST_22339 [Armillaria ostoyae] | 3.50 |
| chr06_AA_00745 | MFS monosaccharide transporter [Armillaria gallica] | 3.49 |
| chr11_AA_01456 | uncharacterized protein ARMOST_16541 [Armillaria ostoyae] | 3.49 |
| chr01_AA_00422 | hypothetical protein M422DRAFT_28159 [Sphaerobolus stellatus SS14] | 3.49 |
| chr05_AA_00860 | hypothetical protein ARMGADRAFT_633196 [Armillaria gallica] | 3.49 |
| chr06_AA_00572 | NA | 3.48 |
| chr03_AA_00194 | acetyl-CoA synthetase-like protein [Gloeophyllum trabeum ATCC 11539] &gt;EPQ54396.1 acetyl-CoA synthetase-like protein [Gloeophyllum trabeum ATCC 11539] | 3.47 |
| chr05_AA_00224 | carotenoid ester lipase precursor [Cylindrobasidium torrendii FP15055 ss-10] | 3.47 |
| chr02_AA_00057 | hypothetical protein ARMSODRAFT_931909 [Armillaria solidipes] &gt;SJL01654.1 uncharacterized protein ARMOST_04977 [Armillaria ostoyae] | 3.47 |
| chr08_AA_00621 | hypothetical protein ARMGADRAFT_982650 [Armillaria gallica] | 3.46 |
| chr06_AA_00949 | hypothetical protein CYLTODRAFT_427642 [Cylindrobasidium torrendii FP15055 ss-10] | 3.45 |
| chr08_AA_00644 | cytochrome P450 [Armillaria solidipes] | 3.44 |
| chr01_AA_00105 | hydrophobin [Flammulina velutipes] | 3.42 |
| chr08_AA_01206 | NA | 3.42 |
| chr03_AA_00277 | Chain A, Crystal Structure Of Fip-fve Fungal Immunomodulatory Protein &gt;1OSY_B Chain B, Crystal Structure Of Fip-fve Fungal Immunomodulatory Protein | 3.42 |
| chr09_AA_00036 | hypothetical protein ARMGADRAFT_1070289 [Armillaria gallica] | 3.40 |
| chr10_AA_00562 | acetate--CoA ligase [Cylindrobasidium torrendii FP15055 ss-10] | 3.39 |
| chr11_AA_01619 | NA | 3.38 |
| chr02_AA_00090 | hypothetical protein ARMGADRAFT_967506 [Armillaria gallica] | 3.37 |
| chr10_AA_00618 | putative cellulase CEL6B [Flammulina velutipes] | 3.37 |
| chr10_AA_00733 | Zinc-regulated transporter 1 [Hypsizygus marmoreus] | 3.37 |
| chr10_AA_01078 | NA | 3.35 |
| chr06_AA_00366 | uncharacterized protein ARMOST_02422 [Armillaria ostoyae] | 3.35 |
| chr08_AA_00496 | amidase signature enzyme [Sanghuangporus baumii] | 3.34 |
| chr11_AA_00233 | hypothetical protein ARMGADRAFT_977028 [Armillaria gallica] | 3.33 |
| chr05_AA_00887 | hypothetical protein SERLA73DRAFT_126376 [Serpula lacrymans var. lacrymans S7.3] | 3.29 |
| chr05_AA_00121 | hypothetical protein CYLTODRAFT_419184 [Cylindrobasidium torrendii FP15055 ss-10] | 3.28 |
| chr11_AA_00579 | NA | 3.27 |
| chr08_AA_00755 | hypothetical protein AMATHDRAFT_60588 [Amanita thiersii Skay4041] | 3.27 |
| chr01_AA_00293 | NAD(P)-binding protein [Cylindrobasidium torrendii FP15055 ss-10] | 3.27 |
| chr09_AA_01114 | glutathione S-transferase family, partial [Flammulina velutipes] | 3.26 |
| chr01_AA_00413 | hypothetical protein ARMGADRAFT_602628 [Armillaria gallica] | 3.25 |
| chr07_AA_00446 | uncharacterized protein ARMOST_00676 [Armillaria ostoyae] | 3.22 |
| chr06_AA_00524 | hypothetical protein ARMGADRAFT_1111301 [Armillaria gallica] | 3.20 |
| chr11_AA_01557 | hypothetical protein CYLTODRAFT_444134 [Cylindrobasidium torrendii FP15055 ss-10] | 3.18 |
| chr08_AA_01205 | NA | 3.18 |
| chr10_AA_00343 | S-adenosyl-L-methionine-dependent methyltransferase [Armillaria solidipes] | 3.17 |
| chr01_AA_00125 | natural resistance-associated macrophage protein [Cylindrobasidium torrendii FP15055 ss-10] | 3.17 |
| chr11_AA_00928 | putative malate synthase [Flammulina velutipes] | 3.16 |
| chr09_AA_01350 | saccharopine dehydrogenase-like oxidoreductase [Cylindrobasidium torrendii FP15055 ss-10] | 3.15 |
| chr04_AA_00651 | uncharacterized protein ARMOST_02737 [Armillaria ostoyae] | 3.15 |
| chr05_AA_00845 | general substrate transporter [Armillaria gallica] | 3.13 |
| chr09_AA_00423 | hypothetical protein CYLTODRAFT_493244 [Cylindrobasidium torrendii FP15055 ss-10] | 3.13 |
| chr11_AA_00878 | hypothetical protein CYLTODRAFT_430286 [Cylindrobasidium torrendii FP15055 ss-10] | 3.13 |
| chr08_AA_01257 | hypothetical protein ARMSODRAFT_949486 [Armillaria solidipes] | 3.12 |
| chr09_AA_00023 | hypothetical protein WG66_107 [Moniliophthora roreri] | 3.10 |
| chr09_AA_00964 | hypothetical protein CYLTODRAFT_421025 [Cylindrobasidium torrendii FP15055 ss-10] | 3.10 |
| chr04_AA_00450 | hypothetical protein Moror_6730 [Moniliophthora roreri MCA 2997] | 3.09 |
| chr04_AA_00045 | hypothetical protein ARMGADRAFT_1084273 [Armillaria gallica] | 3.09 |
| chr08_AA_00240 | glutathione S-transferase [Cylindrobasidium torrendii FP15055 ss-10] | 3.08 |
| chr04_AA_00685 | hypothetical protein CYLTODRAFT_369578 [Cylindrobasidium torrendii FP15055 ss-10] | 3.08 |
| chr11_AA_00627 | NA | 3.08 |
| chr08_AA_01237 | PLP-dependent transferase [Cylindrobasidium torrendii FP15055 ss-10] | 3.07 |
| chr11_AA_00051 | hypothetical protein CYLTODRAFT_420245 [Cylindrobasidium torrendii FP15055 ss-10] | 3.06 |
| chr08_AA_00931 | hypothetical protein HYPSUDRAFT_32617 [Hypholoma sublateritium FD-334 SS-4] | 3.06 |
| chr01_AA_00089 | NA | 3.05 |
| chr09_AA_00312 | putative beta-glucosidase [Flammulina velutipes] | 3.05 |
| chr09_AA_01277 | hypothetical protein GSI_06742 [Ganoderma sinense ZZ0214-1] | 3.05 |
| chr05_AA_00517 | zincin [Cylindrobasidium torrendii FP15055 ss-10] | 3.04 |
| chr06_AA_00970 | glycoside hydrolase family 5 protein [Pleurotus ostreatus PC15] | 3.02 |
| chr06_AA_00971 | uncharacterized protein ARMOST_17209 [Armillaria ostoyae] | 3.02 |
| chr05_AA_00599 | glycoside hydrolase family 2 protein [Cylindrobasidium torrendii FP15055 ss-10] | 3.01 |
| chr08_AA_00284 | FAD/NAD-binding domain-containing protein [Armillaria solidipes] | 3.01 |
| chr11_AA_00333 | NA | 3.00 |
| chr07_AA_01122 | NA | 3.00 |
| chr09_AA_00115 | hypothetical protein CYLTODRAFT_383743 [Cylindrobasidium torrendii FP15055 ss-10] | 2.96 |
| chr11_AA_00103 | hypothetical protein M413DRAFT_370146 [Hebeloma cylindrosporum h7] | 2.96 |
| chr06_AA_00526 | hypothetical protein CYLTODRAFT_420910 [Cylindrobasidium torrendii FP15055 ss-10] | 2.95 |
| chr06_AA_00318 | hypothetical protein ARMGADRAFT_1051359 [Armillaria gallica] | 2.95 |
| chr07_AA_00528 | dihydroxy-acid dehydratase [Armillaria gallica] | 2.95 |
| chr08_AA_01077 | GroES-like protein [Cylindrobasidium torrendii FP15055 ss-10] | 2.94 |
| chr09_AA_00606 | carbohydrate esterase family 12 protein [Peniophora sp. CONT] | 2.94 |
| chr11_AA_01145 | hypothetical protein ARMSODRAFT_964448 [Armillaria solidipes] | 2.94 |
| chr10_AA_00754 | NA | 2.93 |
| chr03_AA_00406 | NA | 2.91 |
| chr05_AA_00686 | glycoside hydrolase family 95 protein [Cylindrobasidium torrendii FP15055 ss-10] | 2.91 |
| chr09_AA_00162 | hypothetical protein ARMGADRAFT_961910 [Armillaria gallica] | 2.90 |
| chr08_AA_00338 | DUF1479-domain-containing protein [Cylindrobasidium torrendii FP15055 ss-10] | 2.88 |
| chr07_AA_00420 | hypothetical protein GALMADRAFT_140021 [Galerina marginata CBS 339.88] | 2.88 |
| chr11_AA_00255 | putative endoglucanase [Flammulina velutipes] | 2.88 |
| chr01_AA_00528 | hypothetical protein ARMGADRAFT_964373 [Armillaria gallica] | 2.86 |
| chr08_AA_00476 | uncharacterized protein ARMOST_15733 [Armillaria ostoyae] | 2.85 |
| chr07_AA_00278 | hypothetical protein ARMSODRAFT_1090165 [Armillaria solidipes] | 2.84 |
| chr08_AA_00148 | hypothetical protein ARMGADRAFT_1015389 [Armillaria gallica] | 2.81 |
| chr08_AA_00960 | 3-ketoacyl-CoA thiolase [Cylindrobasidium torrendii FP15055 ss-10] | 2.81 |
| chr09_AA_00761 | NAD-P-binding protein [Cylindrobasidium torrendii FP15055 ss-10] | 2.80 |
| chr06_AA_00301 | NA | 2.80 |
| chr05_AA_00317 | hypothetical protein CYLTODRAFT_450559 [Cylindrobasidium torrendii FP15055 ss-10] | 2.80 |
| chr01_AA_00035 | uncharacterized protein ARMOST_05935 [Armillaria ostoyae] | 2.80 |
| chr01_AA_00373 | PTR2-domain-containing protein [Exidia glandulosa HHB12029] | 2.79 |
| chr07_AA_00483 | uncharacterized protein ARMOST_00645 [Armillaria ostoyae] | 2.78 |
| chr06_AA_00315 | mitochondrial carrier [Cylindrobasidium torrendii FP15055 ss-10] | 2.77 |
| chr01_AA_00396 | hypothetical protein SERLADRAFT_471207 [Serpula lacrymans var. lacrymans S7.9] &gt;EGN97179.1 hypothetical protein SERLA73DRAFT_183825 [Serpula lacrymans var. lacrymans S7.3] &gt;EGO22788.1 hypothetical protein SERLADRAFT_471207 [Serpula lacrymans var. lacrymans S7.9] | 2.76 |
| chr09_AA_00238 | hypothetical protein CYLTODRAFT_384793 [Cylindrobasidium torrendii FP15055 ss-10] | 2.75 |
| chr04_AA_00409 | hypothetical protein ARMGADRAFT_1013158 [Armillaria gallica] | 2.75 |
| chr10_AA_00499 | hypothetical protein WG66_16220 [Moniliophthora roreri] | 2.73 |
| chr05_AA_00208 | hypothetical protein ARMGADRAFT_1075882 [Armillaria gallica] | 2.73 |
| chr11_AA_01087 | hypothetical protein ARMSODRAFT_1086399 [Armillaria solidipes] | 2.73 |
| chr11_AA_01731 | benzoquinone reductase [Cylindrobasidium torrendii FP15055 ss-10] | 2.73 |
| chr10_AA_00732 | Metallo-dependent phosphatase [Cylindrobasidium torrendii FP15055 ss-10] | 2.72 |
| chr03_AA_00403 | hypothetical protein CYLTODRAFT_424860 [Cylindrobasidium torrendii FP15055 ss-10] | 2.72 |
| chr06_AA_00707 | glycerol kinase [Armillaria solidipes] | 2.71 |
| chr08_AA_00260 | CDF-like metal transporter [Armillaria solidipes] | 2.70 |
| chr08_AA_01091 | uncharacterized protein ARMOST_03386 [Armillaria ostoyae] | 2.68 |
| chr11_AA_00997 | Annexin [Cylindrobasidium torrendii FP15055 ss-10] | 2.68 |
| chr03_AA_00108 | NA | 2.67 |
| chr07_AA_00703 | potassium/sodium eff [Cylindrobasidium torrendii FP15055 ss-10] | 2.67 |
| chr11_AA_00213 | uncharacterized protein ARMOST_19703 [Armillaria ostoyae] | 2.66 |
| chr07_AA_01021 | hypothetical protein PLEOSDRAFT_1096996 [Pleurotus ostreatus PC15] | 2.65 |
| chr09_AA_01359 | glycoside hydrolase [Armillaria gallica] | 2.65 |
| chr09_AA_00718 | NAD(P)-binding protein [Armillaria solidipes] | 2.64 |
| chr11_AA_01386 | NA | 2.64 |
| chr07_AA_01020 | extracellular GDSL-like lipase/acylhydrolase [Glonium stellatum] | 2.64 |
| chr11_AA_01730 | nonribosomal peptide synthetase 12 [Lentinula edodes] | 2.64 |
| chr09_AA_00150 | NA | 2.63 |
| chr06_AA_00869 | carboxylic acid transport protein [Diplodia corticola] &gt;OJD39862.1 carboxylic acid transport protein [Diplodia corticola] | 2.63 |
| chr07_AA_01163 | NA | 2.62 |
| chr09_AA_01214 | hypothetical protein CYLTODRAFT_417942 [Cylindrobasidium torrendii FP15055 ss-10] | 2.61 |
| chr11_AA_00332 | hypothetical protein ARMSODRAFT_1021270 [Armillaria solidipes] | 2.61 |
| chr08_AA_01081 | uncharacterized protein ARMOST_20173 [Armillaria ostoyae] | 2.57 |
| chr06_AA_00347 | hypothetical protein NEOLEDRAFT_1063527, partial [Neolentinus lepideus HHB14362 ss-1] | 2.57 |
| chr05_AA_00118 | glycoside hydrolase family 37 protein [Cylindrobasidium torrendii FP15055 ss-10] | 2.56 |
| chr11_AA_00508 | glycoside hydrolase [Armillaria gallica] | 2.56 |
| chr09_AA_00448 | NA | 2.55 |
| chr07_AA_01017 | laccase [Flammulina velutipes] | 2.54 |
| chr07_AA_01129 | NA | 2.54 |
| chr06_AA_00703 | hypothetical protein CYLTODRAFT_398145 [Cylindrobasidium torrendii FP15055 ss-10] | 2.53 |
| chr07_AA_01051 | MFS general substrate transporter [Cylindrobasidium torrendii FP15055 ss-10] | 2.53 |
| chr05_AA_00715 | NAD(P)-binding protein [Cylindrobasidium torrendii FP15055 ss-10] | 2.53 |
| chr04_AA_00291 | hypothetical protein ARMGADRAFT_1063603 [Armillaria gallica] | 2.52 |
| chr07_AA_00484 | hypothetical protein ARMGADRAFT_932206 [Armillaria gallica] | 2.52 |
| chr07_AA_00414 | MFS general substrate transporter [Armillaria gallica] | 2.51 |
| chr01_AA_00436 | hypothetical protein ARMSODRAFT_960465 [Armillaria solidipes] | 2.51 |
| chr03_AA_00377 | NA | 2.50 |
| chr08_AA_00858 | cytochrome P450 [Cylindrobasidium torrendii FP15055 ss-10] | 2.50 |
| chr05_AA_00215 | 4-O-methyl-glucuronoyl methylesterase [Hypsizygus marmoreus] | 2.49 |
| chr07_AA_00494 | glycoside hydrolase family 5 protein [Cylindrobasidium torrendii FP15055 ss-10] | 2.49 |
| chr09_AA_01493 | NAD(P)-binding protein [Cylindrobasidium torrendii FP15055 ss-10] | 2.49 |
| chr03_AA_00488 | hypothetical protein GYMLUDRAFT_179287 [Gymnopus luxurians FD-317 M1] | 2.49 |
| chr02_AA_00154 | hypothetical protein ARMSODRAFT_1083492 [Armillaria solidipes] | 2.49 |
| chr02_AA_00260 | hypothetical protein CYLTODRAFT_418477 [Cylindrobasidium torrendii FP15055 ss-10] | 2.49 |
| chr06_AA_00424 | NA | 2.49 |
| chr11_AA_01555 | NA | 2.48 |
| chr02_AA_00138 | related to putative tartrate transporter [Armillaria ostoyae] | 2.48 |
| chr10_AA_01201 | acyltransferase ChoActase/COT/CPT [Armillaria gallica] | 2.48 |
| chr08_AA_00776 | Oligoxyloglucan reducing end-specific cellobiohydrolase [Armillaria gallica] | 2.47 |
| chr11_AA_01343 | NA | 2.47 |
| chr06_AA_00233 | hypothetical protein CYLTODRAFT_395535 [Cylindrobasidium torrendii FP15055 ss-10] | 2.47 |
| chr11_AA_01686 | hypothetical protein CYLTODRAFT_348076 [Cylindrobasidium torrendii FP15055 ss-10] | 2.46 |
| chr11_AA_00790 | uncharacterized protein ARMOST_05401 [Armillaria ostoyae] | 2.46 |
| chr11_AA_00606 | hypothetical protein CYLTODRAFT_418833 [Cylindrobasidium torrendii FP15055 ss-10] | 2.45 |
| chr08_AA_00140 | NA | 2.45 |
| chr10_AA_01414 | hypothetical protein ARMSODRAFT_995949 [Armillaria solidipes] | 2.45 |
| chr09_AA_00132 | hypothetical protein CYLTODRAFT_360437 [Cylindrobasidium torrendii FP15055 ss-10] | 2.45 |
| chr10_AA_00662 | Ureohydrolase [Armillaria gallica] | 2.45 |
| chr10_AA_01231 | hypothetical protein ARMSODRAFT_1019532 [Armillaria solidipes] | 2.45 |
| chr02_AA_00393 | related to GRE2-methylglyoxal reductase (NADPH-dependent) [Armillaria ostoyae] | 2.44 |
| chr11_AA_01311 | NA | 2.44 |
| chr09_AA_00095 | hypothetical protein CYLTODRAFT_422329 [Cylindrobasidium torrendii FP15055 ss-10] | 2.43 |
| chr10_AA_00270 | branched-chain alpha-keto acid dehydrogenase E1-alpha subunit [Cylindrobasidium torrendii FP15055 ss-10] | 2.43 |
| chr04_AA_00470 | hypothetical protein PLICRDRAFT_179967 [Plicaturopsis crispa FD-325 SS-3] | 2.43 |
| chr09_AA_00700 | glutathione S-transferase [Cylindrobasidium torrendii FP15055 ss-10] | 2.42 |
| chr05_AA_00719 | lipolytic enzyme [Coprinopsis cinerea okayama7#130] &gt;EAU92167.1 lipolytic enzyme [Coprinopsis cinerea okayama7#130] | 2.42 |
| chr05_AA_00082 | glycoside hydrolase family 20 protein [Cylindrobasidium torrendii FP15055 ss-10] | 2.40 |
| chr09_AA_00429 | uncharacterized protein ARMOST_07282 [Armillaria ostoyae] | 2.39 |
| chr11_AA_00882 | hypothetical protein HETIRDRAFT_480361 [Heterobasidion irregulare TC 32-1] &gt;ETW76290.1 hypothetical protein HETIRDRAFT_480361 [Heterobasidion irregulare TC 32-1] | 2.39 |
| chr08_AA_00595 | phenylacetyl-CoA ligase [Cylindrobasidium torrendii FP15055 ss-10] | 2.38 |
| chr07_AA_00187 | MFS general substrate transporter [Cylindrobasidium torrendii FP15055 ss-10] | 2.38 |
| chr11_AA_00888 | NA | 2.36 |
| chr03_AA_00608 | NA | 2.36 |
| chr08_AA_00518 | Aldo/keto reductase [Cylindrobasidium torrendii FP15055 ss-10] | 2.36 |
| chr09_AA_00762 | transketolase [Armillaria solidipes] | 2.36 |
| chr05_AA_00666 | MATE efflux family protein [Armillaria solidipes] | 2.35 |
| chr07_AA_00874 | hypothetical protein ARMGADRAFT_762077 [Armillaria gallica] | 2.34 |
| chr02_AA_00067 | NA | 2.34 |
| chr08_AA_00167 | Aldo/keto reductase [Armillaria gallica] | 2.33 |
| chr09_AA_00551 | hypothetical protein CYLTODRAFT_392634 [Cylindrobasidium torrendii FP15055 ss-10] | 2.33 |
| chr09_AA_00816 | mandelate racemase muconate lactonizing enzyme family protein [Cylindrobasidium torrendii FP15055 ss-10] | 2.32 |
| chr11_AA_01194 | NA | 2.32 |
| chr10_AA_00134 | hypothetical protein CYLTODRAFT_349864 [Cylindrobasidium torrendii FP15055 ss-10] | 2.32 |
| chr02_AA_00274 | Isochorismatase hydrolase [Cylindrobasidium torrendii FP15055 ss-10] | 2.31 |
| chr04_AA_00062 | glycosyltransferase family 25 domain-containing protein [Rhizoctonia solani AG-1 IA] | 2.31 |
| chr10_AA_00381 | hypothetical protein SCHCODRAFT_51005 [Schizophyllum commune H4-8] &gt;EFJ01265.1 hypothetical protein SCHCODRAFT_51005 [Schizophyllum commune H4-8] | 2.31 |
| chr10_AA_00667 | hypothetical protein CYLTODRAFT_379376 [Cylindrobasidium torrendii FP15055 ss-10] | 2.31 |
| chr02_AA_00301 | Transposon Ty3-G Gag-Pol polyprotein [Trametes pubescens] | 2.31 |
| chr11_AA_00779 | MFS general substrate transporter [Cylindrobasidium torrendii FP15055 ss-10] | 2.31 |
| chr03_AA_00303 | uncharacterized protein ARMOST_16736 [Armillaria ostoyae] | 2.30 |
| chr10_AA_01002 | hypothetical protein ARMGADRAFT_307908 [Armillaria gallica] | 2.30 |
| chr11_AA_00778 | NA | 2.29 |
| chr11_AA_01064 | FAD-binding domain-containing protein [Ceraceosorus guamensis] &gt;PWN39261.1 FAD-binding domain-containing protein [Ceraceosorus guamensis] | 2.29 |
| chr03_AA_00189 | hypothetical protein GYMLUDRAFT_251306 [Gymnopus luxurians FD-317 M1] | 2.29 |
| chr08_AA_00124 | putative hscarg dehydrogenase [Armillaria solidipes] | 2.29 |
| chr09_AA_00018 | oxaloacetate acetylhydrolase [Cylindrobasidium torrendii FP15055 ss-10] | 2.29 |
| chr05_AA_00056 | glycoside hydrolase family 20 protein [Cylindrobasidium torrendii FP15055 ss-10] | 2.29 |
| chr06_AA_00246 | RTA1 like protein [Cylindrobasidium torrendii FP15055 ss-10] | 2.27 |
| chr09_AA_01072 | hypothetical protein GSI_02408 [Ganoderma sinense ZZ0214-1] | 2.27 |
| chr06_AA_00564 | uncharacterized protein ARMOST_07065 [Armillaria ostoyae] | 2.27 |
| chr09_AA_00427 | hypothetical protein DAEQUDRAFT_691752, partial [Daedalea quercina L-15889] | 2.27 |
| chr11_AA_00240 | hypothetical protein GYMLUDRAFT_241830 [Gymnopus luxurians FD-317 M1] | 2.26 |
| chr09_AA_00938 | NA | 2.26 |
| chr09_AA_01225 | NAD(P)-binding protein [Cylindrobasidium torrendii FP15055 ss-10] | 2.26 |
| chr04_AA_00320 | sulfite reductase flavoprotein alpha-component [Moniliophthora roreri MCA 2997] &gt;KTB45206.1 putative nitric-oxide synthase, salivary gland [Moniliophthora roreri] | 2.26 |
| chr03_AA_00654 | MFS general substrate transporter [Cylindrobasidium torrendii FP15055 ss-10] | 2.25 |
| chr03_AA_00291 | hypothetical protein ARMGADRAFT_1170747 [Armillaria gallica] | 2.25 |
| chr09_AA_00406 | uncharacterized protein ARMOST_17180 [Armillaria ostoyae] | 2.25 |
| chr03_AA_00380 | NAD(P)-binding protein [Cylindrobasidium torrendii FP15055 ss-10] | 2.25 |
| chr09_AA_00714 | Clavaminate synthase-like protein, partial [Armillaria gallica] | 2.25 |
| chr09_AA_00149 | hypothetical protein Hypma_006345 [Hypsizygus marmoreus] | 2.24 |
| chr08_AA_00948 | probable glutamate dehydrogenase, NAD(+)-specific [Armillaria ostoyae] | 2.24 |
| chr04_AA_00314 | 3-deoxy-7-phosphoheptulonate synthase [Armillaria solidipes] | 2.24 |
| chr11_AA_01454 | NA | 2.23 |
| chr11_AA_01033 | glycoside hydrolase family 51 protein [Cylindrobasidium torrendii FP15055 ss-10] | 2.23 |
| chr09_AA_00410 | hypothetical protein AMATHDRAFT_73341 [Amanita thiersii Skay4041] | 2.23 |
| chr01_AA_00397 | putative isomerase YbhE [Cylindrobasidium torrendii FP15055 ss-10] | 2.22 |
| chr11_AA_00086 | hypothetical protein ARMGADRAFT_550561 [Armillaria gallica] | 2.22 |
| chr06_AA_00297 | hypothetical protein Hypma_009103 [Hypsizygus marmoreus] | 2.22 |
| chr05_AA_00699 | hydrophobin [Flammulina velutipes] | 2.21 |
| chr08_AA_01278 | hypothetical protein ARMGADRAFT_1007332 [Armillaria gallica] | 2.21 |
| chr10_AA_01143 | uncharacterized protein ARMOST_08409 [Armillaria ostoyae] | 2.21 |
| chr09_AA_00934 | hypothetical protein ARMSODRAFT_950051 [Armillaria solidipes] | 2.21 |
| chr06_AA_00537 | NA | 2.21 |
| chr07_AA_00079 | NA | 2.21 |
| chr09_AA_01106 | multidrug transporter [Cylindrobasidium torrendii FP15055 ss-10] | 2.20 |
| chr02_AA_00121 | vacuolar amino acid permease [Armillaria gallica] | 2.19 |
| chr05_AA_00483 | thioredoxin-like protein [Cylindrobasidium torrendii FP15055 ss-10] | 2.19 |
| chr10_AA_01039 | NA | 2.18 |
| chr09_AA_00411 | hypothetical protein CYLTODRAFT_488269 [Cylindrobasidium torrendii FP15055 ss-10] | 2.18 |
| chr11_AA_00236 | hypothetical protein GYMLUDRAFT_994181 [Gymnopus luxurians FD-317 M1] | 2.18 |
| chr02_AA_00216 | S-adenosyl-L-methionine-dependent methyltransferase [Armillaria solidipes] | 2.18 |
| chr07_AA_01034 | uncharacterized protein ARMOST_01203 [Armillaria ostoyae] | 2.18 |
| chr10_AA_00220 | hypothetical protein ARMGADRAFT_962646, partial [Armillaria gallica] | 2.17 |
| chr01_AA_00108 | NA | 2.17 |
| chr05_AA_00365 | hypothetical protein CYLTODRAFT_441111 [Cylindrobasidium torrendii FP15055 ss-10] | 2.16 |
| chr07_AA_00464 | cytochrome P450 [Cylindrobasidium torrendii FP15055 ss-10] | 2.16 |
| chr05_AA_00627 | MFS general substrate transporter [Cylindrobasidium torrendii FP15055 ss-10] | 2.16 |
| chr08_AA_01097 | hypothetical protein GYMLUDRAFT_32937 [Gymnopus luxurians FD-317 M1] | 2.15 |
| chr10_AA_01063 | glycoside hydrolase family 43 protein [Amanita thiersii Skay4041] | 2.15 |
| chr10_AA_00943 | hypothetical protein CYLTODRAFT_417473 [Cylindrobasidium torrendii FP15055 ss-10] | 2.15 |
| chr08_AA_01254 | cytochrome P450 [Cylindrobasidium torrendii FP15055 ss-10] | 2.14 |
| chr10_AA_01056 | hypothetical protein ARMGADRAFT_1020988 [Armillaria gallica] | 2.14 |
| chr10_AA_00646 | putative flavin-containing monooxygenase YUCCA3 [Cylindrobasidium torrendii FP15055 ss-10] | 2.14 |
| chr07_AA_00188 | NA | 2.13 |
| chr11_AA_01418 | NA | 2.13 |
| chr09_AA_01152 | general substrate transporter [Armillaria solidipes] | 2.13 |
| chr09_AA_00338 | hypothetical protein ARMGADRAFT_1021227 [Armillaria gallica] | 2.13 |
| chr11_AA_00031 | glycoside hydrolase family 61 protein [Gymnopus luxurians FD-317 M1] | 2.12 |
| chr05_AA_00875 | hypothetical protein CYLTODRAFT_490261 [Cylindrobasidium torrendii FP15055 ss-10] | 2.12 |
| chr08_AA_01106 | hypothetical protein ARMSODRAFT_963093 [Armillaria solidipes] | 2.12 |
| chr09_AA_00351 | cytochrome P450 [Armillaria gallica] | 2.12 |
| chr02_AA_00341 | oxidoreductase [Armillaria solidipes] | 2.12 |
| chr10_AA_00155 | uncharacterized protein ARMOST_08333 [Armillaria ostoyae] | 2.12 |
| chr09_AA_00564 | NA | 2.10 |
| chr10_AA_01664 | hypothetical protein CYLTODRAFT_404266 [Cylindrobasidium torrendii FP15055 ss-10] | 2.10 |
| chr01_AA_00302 | hypothetical protein CYLTODRAFT_420700 [Cylindrobasidium torrendii FP15055 ss-10] | 2.09 |
| chr07_AA_00603 | hypothetical protein GYMLUDRAFT_69434 [Gymnopus luxurians FD-317 M1] | 2.09 |
| chr03_AA_00462 | hypothetical protein GYMLUDRAFT_942491 [Gymnopus luxurians FD-317 M1] | 2.09 |
| chr04_AA_00384 | hypothetical protein ARMSODRAFT_784657 [Armillaria solidipes] | 2.09 |
| chr06_AA_00506 | MFS general substrate transporter [Cylindrobasidium torrendii FP15055 ss-10] | 2.08 |
| chr07_AA_00978 | putative polyketide synthase [Armillaria gallica] | 2.08 |
| chr10_AA_00574 | 2-oxoisovalerate dehydrogenase subunit beta, mitochondrial [Hypsizygus marmoreus] | 2.07 |
| chr08_AA_00280 | hypothetical protein ARMGADRAFT_1006514 [Armillaria gallica] | 2.07 |
| chr11_AA_00719 | hypothetical protein ARMGADRAFT_1019784, partial [Armillaria gallica] | 2.07 |
| chr09_AA_00325 | uncharacterized protein ARMOST_05718 [Armillaria ostoyae] | 2.07 |
| chr05_AA_00812 | acid protease [Armillaria gallica] | 2.07 |
| chr10_AA_00441 | alpha/beta-hydrolase [Armillaria solidipes] | 2.06 |
| chr03_AA_00063 | hypothetical protein CYLTODRAFT_422586 [Cylindrobasidium torrendii FP15055 ss-10] | 2.06 |
| chr08_AA_00313 | hypothetical protein CYLTODRAFT_376076 [Cylindrobasidium torrendii FP15055 ss-10] | 2.06 |
| chr08_AA_00101 | MFS general substrate transporter [Cylindrobasidium torrendii FP15055 ss-10] | 2.06 |
| chr07_AA_00616 | NA | 2.06 |
| chr03_AA_00188 | O-methylsterigmatocystin oxidoreductase [Termitomyces sp. J132] | 2.05 |
| chr03_AA_00148 | hypothetical protein CYLTODRAFT_456087 [Cylindrobasidium torrendii FP15055 ss-10] | 2.05 |
| chr01_AA_00418 | aromatic compound dioxygenase [Cylindrobasidium torrendii FP15055 ss-10] | 2.05 |
| chr08_AA_01196 | hypothetical protein ARMGADRAFT_1007481 [Armillaria gallica] | 2.05 |
| chr08_AA_00651 | related to isp4-oligopeptide transporter [Armillaria ostoyae] | 2.03 |
| chr01_AA_00146 | putative hydrophobin [Flammulina velutipes] | 2.03 |
| chr10_AA_00911 | uncharacterized protein ARMOST_09741 [Armillaria ostoyae] | 2.02 |
| chr10_AA_01309 | hypothetical protein PHLCEN_2v2282 [Phlebia centrifuga] | 2.02 |
| chr07_AA_00291 | uncharacterized protein ARMOST_18337 [Armillaria ostoyae] | 2.02 |
| chr11_AA_00831 | hypothetical protein GYMLUDRAFT_71628 [Gymnopus luxurians FD-317 M1] | 2.01 |
| chr08_AA_00716 | hypothetical protein ARMGADRAFT_399850 [Armillaria gallica] | 2.00 |
| chr04_AA_00044 | NA | 2.00 |
| chr07_AA_00120 | hypothetical protein ARMGADRAFT_1163980 [Armillaria gallica] | 2.00 |
| chr03_AA_00413 | hypothetical protein CYLTODRAFT_491889 [Cylindrobasidium torrendii FP15055 ss-10] | 1.99 |
| chr10_AA_00671 | MFS general substrate transporter [Cylindrobasidium torrendii FP15055 ss-10] | 1.98 |
| chr11_AA_01241 | ubiquinone biosynthesis O-methyltransferase [Cylindrobasidium torrendii FP15055 ss-10] | 1.98 |
| chr07_AA_00495 | hypothetical protein PLEOSDRAFT_1114137 [Pleurotus ostreatus PC15] | 1.97 |
| chr06_AA_00683 | amidohydrolase [Armillaria gallica] | 1.97 |
| chr08_AA_00542 | hypothetical protein ARMGADRAFT_1159894 [Armillaria gallica] | 1.96 |
| chr07_AA_00115 | hypothetical protein CYLTODRAFT_357659 [Cylindrobasidium torrendii FP15055 ss-10] | 1.96 |
| chr07_AA_00561 | hypothetical protein ARMGADRAFT_1031622 [Armillaria gallica] | 1.96 |
| chr08_AA_01032 | hypothetical protein CYLTODRAFT_445755 [Cylindrobasidium torrendii FP15055 ss-10] | 1.96 |
| chr10_AA_01310 | DUF124-domain-containing protein [Cylindrobasidium torrendii FP15055 ss-10] | 1.96 |
| chr02_AA_00171 | hypothetical protein CYLTODRAFT_425697 [Cylindrobasidium torrendii FP15055 ss-10] | 1.96 |
| chr10_AA_01036 | hypothetical protein CYLTODRAFT_401684 [Cylindrobasidium torrendii FP15055 ss-10] | 1.96 |
| chr08_AA_00834 | related to galactinol synthase [Armillaria ostoyae] | 1.95 |
| chr09_AA_00826 | hypothetical protein ARMGADRAFT_1010212 [Armillaria gallica] | 1.94 |
| chr09_AA_01103 | Metallo-dependent phosphatase [Armillaria solidipes] | 1.94 |
| chr07_AA_01006 | enolase C-terminal domain-like protein [Cylindrobasidium torrendii FP15055 ss-10] | 1.93 |
| chr11_AA_01099 | hypothetical protein CYLTODRAFT_187395 [Cylindrobasidium torrendii FP15055 ss-10] | 1.93 |
| chr07_AA_00645 | aldehyde dehydrogenase [Armillaria gallica] | 1.93 |
| chr03_AA_00525 | jacalin-related lectin [Flammulina velutipes] | 1.92 |
| chr09_AA_00838 | cytochrome P450 [Armillaria gallica] | 1.92 |
| chr06_AA_00346 | hypothetical protein CVT25_010141 [Psilocybe cyanescens] | 1.91 |
| chr10_AA_00584 | NA | 1.91 |
| chr03_AA_00259 | class I glutamine amidotransferase-like protein [Cylindrobasidium torrendii FP15055 ss-10] | 1.91 |
| chr08_AA_00577 | hypothetical protein ARMSODRAFT_610398 [Armillaria solidipes] &gt;PBK62736.1 hypothetical protein ARMSODRAFT_560192 [Armillaria solidipes] | 1.91 |
| chr07_AA_00419 | hypothetical protein CYLTODRAFT_277105 [Cylindrobasidium torrendii FP15055 ss-10] | 1.91 |
| chr10_AA_00947 | hypothetical protein AZE42_04166, partial [Rhizopogon vesiculosus] | 1.91 |
| chr08_AA_00018 | putative peroxiredoxin Q [Flammulina velutipes] | 1.90 |
| chr07_AA_00826 | Metallo-hydrolase/oxidoreductase [Armillaria gallica] | 1.89 |
| chr09_AA_01087 | NAD(P)-binding protein [Cylindrobasidium torrendii FP15055 ss-10] | 1.89 |
| chr08_AA_01176 | hypothetical protein HYPSUDRAFT_61393 [Hypholoma sublateritium FD-334 SS-4] | 1.89 |
| chr11_AA_01549 | hypothetical protein CYLTODRAFT_455062 [Cylindrobasidium torrendii FP15055 ss-10] | 1.89 |
| chr11_AA_01711 | hypothetical protein ARMGADRAFT_1046867 [Armillaria gallica] | 1.89 |
| chr09_AA_01116 | hypothetical protein CYLTODRAFT_375858 [Cylindrobasidium torrendii FP15055 ss-10] | 1.89 |
| chr11_AA_01188 | hypothetical protein CYLTODRAFT_386603 [Cylindrobasidium torrendii FP15055 ss-10] | 1.89 |
| chr10_AA_01213 | hypothetical protein JAAARDRAFT_210932 [Jaapia argillacea MUCL 33604] | 1.88 |
| chr08_AA_01259 | Aldo/keto reductase [Armillaria gallica] | 1.88 |
| chr05_AA_00339 | copper radical oxidase [Amanita thiersii Skay4041] | 1.87 |
| chr06_AA_00530 | hypothetical protein CYLTODRAFT_373087 [Cylindrobasidium torrendii FP15055 ss-10] | 1.87 |
| chr10_AA_00398 | NA | 1.86 |
| chr03_AA_00192 | O-methylsterigmatocystin oxidoreductase [Termitomyces sp. J132] | 1.86 |
| chr11_AA_00265 | FAD-linked oxidoreductase [Armillaria solidipes] | 1.85 |
| chr09_AA_00354 | NA | 1.85 |
| chr09_AA_01246 | NA | 1.85 |
| chr04_AA_00090 | alpha/beta-hydrolase [Polyporus brumalis] | 1.85 |
| chr05_AA_00668 | homogentisate 1,2-dioxygenase [Armillaria solidipes] | 1.85 |
| chr11_AA_00076 | putative beta-glucan synthesis-associated protein [Flammulina velutipes] | 1.85 |
| chr04_AA_00050 | hypothetical protein CYLTODRAFT_421179 [Cylindrobasidium torrendii FP15055 ss-10] | 1.84 |
| chr03_AA_00181 | chitin deacetylase [Coprinopsis cinerea okayama7#130] &gt;EAU83261.2 chitin deacetylase [Coprinopsis cinerea okayama7#130] | 1.84 |
| chr04_AA_00040 | ornithine aminotransferase [Cylindrobasidium torrendii FP15055 ss-10] | 1.84 |
| chr10_AA_01576 | D-arabinitol 2-dehydrogenase [ribulose-forming] [Hypsizygus marmoreus] | 1.84 |
| chr02_AA_00129 | hypothetical protein CYLTODRAFT_366704 [Cylindrobasidium torrendii FP15055 ss-10] | 1.84 |
| chr08_AA_00826 | hypothetical protein PLEOSDRAFT_1062660 [Pleurotus ostreatus PC15] | 1.83 |
| chr08_AA_01252 | hypothetical protein ARMGADRAFT_1059619 [Armillaria gallica] | 1.83 |
| chr07_AA_00560 | hypothetical protein CYLTODRAFT_416229 [Cylindrobasidium torrendii FP15055 ss-10] | 1.83 |
| chr11_AA_01644 | hypothetical protein CYLTODRAFT_438918 [Cylindrobasidium torrendii FP15055 ss-10] | 1.83 |
| chr11_AA_00814 | BNR/Asp-box repeat protein [Armillaria solidipes] | 1.82 |
| chr10_AA_00252 | hypothetical protein CVT24_007015 [Panaeolus cyanescens] | 1.82 |
| chr03_AA_00414 | putative pheromone receptor [Flammulina velutipes] | 1.82 |
| chr07_AA_00194 | putative GNAT family acetyltransferase [Cylindrobasidium torrendii FP15055 ss-10] | 1.81 |
| chr07_AA_00917 | glycosyltransferase family 69 protein [Cylindrobasidium torrendii FP15055 ss-10] | 1.81 |
| chr07_AA_00666 | hypothetical protein CVT25_013058 [Psilocybe cyanescens] | 1.81 |
| chr09_AA_01324 | uncharacterized protein ARMOST_03294 [Armillaria ostoyae] | 1.80 |
| chr10_AA_00256 | uncharacterized protein ARMOST_03672 [Armillaria ostoyae] | 1.80 |
| chr09_AA_00753 | NA | 1.80 |
| chr04_AA_00466 | glycosyltransferase family 90 protein [Amanita muscaria Koide BX008] | 1.79 |
| chr11_AA_00701 | uncharacterized protein ARMOST_05344 [Armillaria ostoyae] | 1.79 |
| chr05_AA_00800 | NA | 1.79 |
| chr11_AA_00498 | hypothetical protein K443DRAFT_677394 [Laccaria amethystina LaAM-08-1] | 1.78 |
| chr01_AA_00228 | hypothetical protein CYLTODRAFT_424895 [Cylindrobasidium torrendii FP15055 ss-10] | 1.78 |
| chr07_AA_00024 | hypothetical protein CYLTODRAFT_485887 [Cylindrobasidium torrendii FP15055 ss-10] | 1.78 |
| chr10_AA_01214 | NA | 1.78 |
| chr07_AA_00859 | hypothetical protein ARMSODRAFT_875663 [Armillaria solidipes] | 1.78 |
| chr04_AA_00509 | hypothetical protein CYLTODRAFT_397913 [Cylindrobasidium torrendii FP15055 ss-10] | 1.78 |
| chr07_AA_00739 | hypothetical protein CYLTODRAFT_417357 [Cylindrobasidium torrendii FP15055 ss-10] | 1.77 |
| chr07_AA_00907 | uncharacterized protein ARMOST_00985 [Armillaria ostoyae] | 1.77 |
| chr06_AA_00308 | hypothetical protein CYLTODRAFT_397357 [Cylindrobasidium torrendii FP15055 ss-10] | 1.77 |
| chr11_AA_01636 | hypothetical protein CYLTODRAFT_421880 [Cylindrobasidium torrendii FP15055 ss-10] | 1.77 |
| chr08_AA_00106 | hypothetical protein ARMGADRAFT_100162 [Armillaria gallica] | 1.75 |
| chr05_AA_00841 | hypothetical protein CYLTODRAFT_372585 [Cylindrobasidium torrendii FP15055 ss-10] | 1.75 |
| chr07_AA_00281 | NAD(P)-binding protein [Armillaria gallica] | 1.74 |
| chr04_AA_00052 | hypothetical protein CYLTODRAFT_421165 [Cylindrobasidium torrendii FP15055 ss-10] | 1.73 |
| chr08_AA_01172 | carbohydrate esterase family 1 protein, partial [Cylindrobasidium torrendii FP15055 ss-10] | 1.73 |
| chr01_AA_00234 | related to GABA transport protein [Armillaria ostoyae] | 1.73 |
| chr08_AA_01026 | cytochrome P450 [Armillaria solidipes] | 1.72 |
| chr08_AA_00265 | peroxisomal copper amine oxidase [Armillaria solidipes] | 1.71 |
| chr11_AA_01106 | related to stomatin [Armillaria ostoyae] | 1.71 |
| chr10_AA_01390 | hypothetical protein CYLTODRAFT_414265 [Cylindrobasidium torrendii FP15055 ss-10] | 1.71 |
| chr07_AA_01044 | uncharacterized protein ARMOST_19175 [Armillaria ostoyae] | 1.71 |
| chr11_AA_01324 | terpenoid synthase [Cylindrobasidium torrendii FP15055 ss-10] | 1.70 |
| chr06_AA_00639 | actin-like ATPase domain-containing protein [Cylindrobasidium torrendii FP15055 ss-10] | 1.70 |
| chr07_AA_00029 | OPT-domain-containing protein [Armillaria gallica] | 1.70 |
| chr05_AA_00182 | NA | 1.69 |
| chr01_AA_00365 | related to lactonohydrolase [Armillaria ostoyae] | 1.68 |
| chr09_AA_00088 | uncharacterized protein ARMOST_21406 [Armillaria ostoyae] | 1.68 |
| chr11_AA_01135 | hexose transporter [Armillaria gallica] | 1.68 |
| chr10_AA_00912 | hypothetical protein ARMSODRAFT_948409 [Armillaria solidipes] | 1.67 |
| chr07_AA_01032 | glycoside hydrolase family 92 protein [Armillaria gallica] | 1.67 |
| chr07_AA_00201 | NA | 1.66 |
| chr05_AA_00152 | NA | 1.66 |
| chr09_AA_00009 | NA | 1.66 |
| chr11_AA_00027 | hypothetical protein CYLTODRAFT_423477 [Cylindrobasidium torrendii FP15055 ss-10] | 1.66 |
| chr07_AA_01060 | Flavocytochrome c [Armillaria gallica] | 1.66 |
| chr09_AA_00473 | uncharacterized protein ARMOST_08501 [Armillaria ostoyae] | 1.66 |
| chr07_AA_00274 | related to nucleoside-diphosphate-sugar epimerase family protein [Armillaria ostoyae] | 1.65 |
| chr01_AA_00447 | related to permeases of the major facilitator superfamily [Armillaria ostoyae] | 1.65 |
| chr07_AA_01018 | putative laccase 17 [Flammulina velutipes] | 1.65 |
| chr11_AA_01399 | NA | 1.65 |
| chr10_AA_01427 | NA | 1.65 |
| chr06_AA_00897 | hypothetical protein WG66_4545 [Moniliophthora roreri] | 1.64 |
| chr03_AA_00191 | NA | 1.64 |
| chr07_AA_00098 | hypothetical protein ARMGADRAFT_926019 [Armillaria gallica] | 1.64 |
| chr01_AA_00005 | hypothetical protein K503DRAFT_771265 [Rhizopogon vinicolor AM-OR11-026] | 1.64 |
| chr06_AA_00394 | hypothetical protein PLICRDRAFT_113002 [Plicaturopsis crispa FD-325 SS-3] | 1.64 |
| chr05_AA_00607 | hypothetical protein CYLTODRAFT_458172 [Cylindrobasidium torrendii FP15055 ss-10] | 1.63 |
| chr11_AA_00724 | WD40 repeat-like protein [Armillaria solidipes] | 1.63 |
| chr11_AA_01256 | uncharacterized protein ARMOST_07146 [Armillaria ostoyae] | 1.63 |
| chr11_AA_00222 | glycoside hydrolase family 18 protein [Cylindrobasidium torrendii FP15055 ss-10] | 1.63 |
| chr09_AA_01174 | hypothetical protein ARMSODRAFT_1089157 [Armillaria solidipes] | 1.63 |
| chr05_AA_00112 | hypothetical protein STEHIDRAFT_155150 [Stereum hirsutum FP-91666 SS1] &gt;EIM87778.1 hypothetical protein STEHIDRAFT_155150 [Stereum hirsutum FP-91666 SS1] | 1.62 |
| chr07_AA_00457 | uncharacterized protein ARMOST_00617 [Armillaria ostoyae] | 1.62 |
| chr11_AA_01733 | glycoside hydrolase family 76 protein [Cylindrobasidium torrendii FP15055 ss-10] | 1.62 |
| chr09_AA_01024 | putative peroxysomal citrate synthase [Flammulina velutipes] | 1.62 |
| chr01_AA_00217 | NA | 1.61 |
| chr11_AA_00300 | NA | 1.61 |
| chr07_AA_00773 | uncharacterized protein ARMOST_00939 [Armillaria ostoyae] | 1.61 |
| chr09_AA_00724 | hypothetical protein A7U60_g7603 [Sanghuangporus baumii] | 1.61 |
| chr10_AA_01032 | hypothetical protein CYLTODRAFT_408716 [Cylindrobasidium torrendii FP15055 ss-10] | 1.61 |
| chr10_AA_00164 | dehydrogenase E1 and transketolase domain-containing protein 1 [Cylindrobasidium torrendii FP15055 ss-10] | 1.61 |
| chr01_AA_00479 | uncharacterized protein ARMOST_12330 [Armillaria ostoyae] | 1.60 |
| chr06_AA_00670 | NA | 1.60 |
| chr11_AA_00752 | hypothetical protein ARMGADRAFT_1159005 [Armillaria gallica] | 1.59 |
| chr08_AA_00856 | cytochrome p450 [Moniliophthora roreri MCA 2997] | 1.59 |
| chr11_AA_01115 | probable Delta-1-pyrroline-5-carboxylate dehydrogenase [Armillaria ostoyae] | 1.59 |
| chr11_AA_01313 | hypothetical protein Hypma_000372 [Hypsizygus marmoreus] | 1.59 |
| chr11_AA_00670 | hypothetical protein ARMSODRAFT_956526 [Armillaria solidipes] &gt;SJL02113.1 uncharacterized protein ARMOST_05437 [Armillaria ostoyae] | 1.58 |
| chr07_AA_00827 | hypothetical protein LENED_003150 [Lentinula edodes] | 1.58 |
| chr05_AA_00423 | Geranylgeranyl transferase type-2 subunit beta [Grifola frondosa] | 1.58 |
| chr11_AA_00504 | MFS general substrate transporter [Cylindrobasidium torrendii FP15055 ss-10] | 1.57 |
| chr07_AA_01003 | Pkinase-domain-containing protein [Armillaria gallica] | 1.56 |
| chr08_AA_01071 | MFS general substrate transporter [Cylindrobasidium torrendii FP15055 ss-10] | 1.56 |
| chr03_AA_00411 | STE-domain-containing protein [Armillaria solidipes] | 1.56 |
| chr11_AA_01622 | uncharacterized protein ARMOST_13792 [Armillaria ostoyae] | 1.56 |
| chr11_AA_01015 | acid protease [Armillaria gallica] | 1.56 |
| chr08_AA_00847 | acetyl-CoA synthetase-like protein [Armillaria solidipes] | 1.56 |
| chr08_AA_00501 | hypothetical protein CYLTODRAFT_416851 [Cylindrobasidium torrendii FP15055 ss-10] | 1.55 |
| chr10_AA_00996 | photo-regulated tyrosinase [Armillaria solidipes] | 1.55 |
| chr05_AA_00473 | hypothetical protein CYLTODRAFT_449880 [Cylindrobasidium torrendii FP15055 ss-10] | 1.55 |
| chr11_AA_01732 | glycoside hydrolase family 76 protein [Cylindrobasidium torrendii FP15055 ss-10] | 1.55 |
| chr01_AA_00488 | acid protease [Armillaria gallica] | 1.55 |
| chr11_AA_01609 | uncharacterized protein ARMOST_13777 [Armillaria ostoyae] | 1.54 |
| chr05_AA_00425 | copper radical oxidase [Cylindrobasidium torrendii FP15055 ss-10] | 1.54 |
| chr11_AA_00145 | hypothetical protein CYLTODRAFT_243491 [Cylindrobasidium torrendii FP15055 ss-10] | 1.54 |
| chr03_AA_00376 | uncharacterized protein ARMOST_10275 [Armillaria ostoyae] | 1.54 |
| chr09_AA_00055 | Pyranose dehydrogenase 3 [Hypsizygus marmoreus] | 1.53 |
| chr10_AA_01442 | NA | 1.53 |
| chr05_AA_00481 | hypothetical protein CYLTODRAFT_424727 [Cylindrobasidium torrendii FP15055 ss-10] | 1.53 |
| chr07_AA_01008 | cytochrome P450 [Armillaria solidipes] | 1.53 |
| chr11_AA_00580 | putative laccase 6 [Flammulina velutipes] &gt;AIW01081.1 laccase [Flammulina velutipes] | 1.53 |
| chr03_AA_00230 | hypothetical protein CYLTODRAFT_457867 [Cylindrobasidium torrendii FP15055 ss-10] | 1.52 |
| chr08_AA_01215 | hypothetical protein ARMSODRAFT_1014129 [Armillaria solidipes] &gt;SJL07826.1 uncharacterized protein ARMOST_11178 [Armillaria ostoyae] | 1.52 |
| chr06_AA_00237 | acyl-CoA dehydrogenase NM domain-like protein, partial [Armillaria solidipes] | 1.52 |
| chr08_AA_00383 | NAD(P)-binding protein [Armillaria gallica] | 1.52 |
| chr07_AA_00203 | NA | 1.52 |
| chr08_AA_00936 | MFS general substrate transporter [Armillaria gallica] | 1.52 |
| chr10_AA_00651 | hypothetical protein ARMGADRAFT_897215, partial [Armillaria gallica] | 1.51 |
| chr01_AA_00076 | FUN34 transmembrane protein [Cylindrobasidium torrendii FP15055 ss-10] | 1.51 |
| chr08_AA_01175 | hypothetical protein HYPSUDRAFT_61393 [Hypholoma sublateritium FD-334 SS-4] | 1.50 |
| chr01_AA_00227 | NA | 1.50 |
| chr11_AA_01078 | hypothetical protein CYLTODRAFT_371110, partial [Cylindrobasidium torrendii FP15055 ss-10] | 1.50 |
| chr11_AA_01665 | NA | 1.50 |
| chr09_AA_00860 | glycosyltransferase family 90 protein [Cylindrobasidium torrendii FP15055 ss-10] | 1.50 |
| chr09_AA_00880 | Pkinase-domain-containing protein [Armillaria solidipes] | 1.49 |
| chr09_AA_00704 | hypothetical protein ARMGADRAFT_1157373 [Armillaria gallica] &gt;SJL10003.1 uncharacterized protein ARMOST_13385 [Armillaria ostoyae] | 1.49 |
| chr07_AA_00817 | DNA glycosylase [Cylindrobasidium torrendii FP15055 ss-10] | 1.49 |
| chr03_AA_00412 | NA | 1.49 |
| chr06_AA_00426 | hypothetical protein ARMGADRAFT_984775 [Armillaria gallica] | 1.49 |
| chr08_AA_01183 | hypothetical protein ARMSODRAFT_937831 [Armillaria solidipes] | 1.48 |
| chr09_AA_01129 | cytochrome P450 [Armillaria gallica] | 1.48 |
| chr08_AA_01040 | hypothetical protein A0H81_13690 [Grifola frondosa] | 1.48 |
| chr11_AA_00495 | hypothetical protein ARMSODRAFT_946498 [Armillaria solidipes] | 1.48 |
| chr09_AA_00182 | uncharacterized protein ARMOST_20181 [Armillaria ostoyae] | 1.48 |
| chr09_AA_01392 | aldolase [Armillaria gallica] | 1.47 |
| chr10_AA_01509 | hypothetical protein ARMSODRAFT_309875 [Armillaria solidipes] | 1.47 |
| chr09_AA_01410 | hypothetical protein CYLTODRAFT_489277 [Cylindrobasidium torrendii FP15055 ss-10] | 1.47 |
| chr01_AA_00120 | aldehyde dehydrogenase [Armillaria solidipes] &gt;SJL12506.1 probable Aldehyde dehydrogenase [Armillaria ostoyae] | 1.46 |
| chr11_AA_01445 | hypothetical protein ARMSODRAFT_950555 [Armillaria solidipes] &gt;SJL10115.1 uncharacterized protein ARMOST_13499 [Armillaria ostoyae] | 1.46 |
| chr08_AA_00612 | NA | 1.46 |
| chr08_AA_00287 | hypothetical protein PLICRDRAFT_118622 [Plicaturopsis crispa FD-325 SS-3] | 1.46 |
| chr01_AA_00268 | ubiquitin family protein [Moniliophthora roreri MCA 2997] &gt;KTB31855.1 hypothetical protein WG66_15567 [Moniliophthora roreri] | 1.45 |
| chr03_AA_00179 | 6-phosphogluconate dehydrogenase C-terminal domain-like protein [Fistulina hepatica ATCC 64428] | 1.45 |
| chr02_AA_00284 | alcohol dehydrogenase [Armillaria solidipes] | 1.45 |
| chr07_AA_00951 | acyl-CoA dehydrogenase domain-containing protein [Cylindrobasidium torrendii FP15055 ss-10] | 1.45 |
| chr08_AA_01008 | NA | 1.45 |
| chr10_AA_01341 | glucuronyl hydrolase [Armillaria solidipes] | 1.45 |
| chr09_AA_01058 | hypothetical protein ARMSODRAFT_976614 [Armillaria solidipes] | 1.44 |
| chr07_AA_01000 | hypothetical protein CYLTODRAFT_445440 [Cylindrobasidium torrendii FP15055 ss-10] | 1.44 |
| chr01_AA_00445 | NA | 1.44 |
| chr11_AA_00653 | xylitol dehydrogenase [Armillaria gallica] | 1.44 |
| chr09_AA_00859 | uncharacterized protein ARMOST_13189 [Armillaria ostoyae] | 1.44 |
| chr08_AA_00713 | biotin synthase [Armillaria gallica] | 1.43 |
| chr09_AA_00139 | hypothetical protein CYLTODRAFT_424555 [Cylindrobasidium torrendii FP15055 ss-10] | 1.43 |
| chr01_AA_00484 | NA | 1.43 |
| chr07_AA_01089 | cysteine proteinase [Cylindrobasidium torrendii FP15055 ss-10] | 1.42 |
| chr08_AA_00687 | Clavaminate synthase-like protein [Armillaria solidipes] | 1.42 |
| chr05_AA_00865 | hypothetical protein CYLTODRAFT_450275 [Cylindrobasidium torrendii FP15055 ss-10] | 1.41 |
| chr01_AA_00360 | P-loop containing nucleoside triphosphate hydrolase protein [Cylindrobasidium torrendii FP15055 ss-10] | 1.41 |
| chr10_AA_00148 | sulfate permease [Cylindrobasidium torrendii FP15055 ss-10] | 1.40 |
| chr08_AA_01149 | hypothetical protein ARMSODRAFT_965063 [Armillaria solidipes] | 1.40 |
| chr01_AA_00301 | manganese and iron superoxide dismutase [Cylindrobasidium torrendii FP15055 ss-10] | 1.40 |
| chr04_AA_00046 | hypothetical protein PILCRDRAFT_3731 [Piloderma croceum F 1598] | 1.39 |
| chr11_AA_01321 | hypothetical protein CYLTODRAFT_423832 [Cylindrobasidium torrendii FP15055 ss-10] | 1.39 |
| chr01_AA_00369 | sugar transporter [Cylindrobasidium torrendii FP15055 ss-10] | 1.38 |
| chr04_AA_00481 | putative flavonol reductase/cinnamoyl-CoA reductase [Flammulina velutipes] | 1.38 |
| chr10_AA_00616 | hypothetical protein ARMGADRAFT_1012740 [Armillaria gallica] | 1.38 |
| chr11_AA_00042 | kinase-like protein [Cylindrobasidium torrendii FP15055 ss-10] | 1.38 |
| chr08_AA_01069 | alpha/beta-hydrolase [Armillaria solidipes] | 1.38 |
| chr08_AA_01114 | hypothetical protein CYLTODRAFT_399177 [Cylindrobasidium torrendii FP15055 ss-10] | 1.37 |
| chr07_AA_00055 | metallopeptidase M36 [Flammulina velutipes] | 1.37 |
| chr11_AA_00277 | uncharacterized protein ARMOST_10602 [Armillaria ostoyae] | 1.37 |
| chr06_AA_00738 | pyridoxal phosphate-dependent enzyme, beta subunit [Cylindrobasidium torrendii FP15055 ss-10] | 1.37 |
| chr08_AA_01155 | related to 3-oxoacyl CoA thiolase [Armillaria ostoyae] | 1.37 |
| chr10_AA_00699 | hypothetical protein ARMSODRAFT_873731 [Armillaria solidipes] | 1.37 |
| chr09_AA_01141 | hypothetical protein ARMGADRAFT_1063089 [Armillaria gallica] | 1.37 |
| chr01_AA_00426 | hypothetical protein CYLTODRAFT_371735 [Cylindrobasidium torrendii FP15055 ss-10] | 1.36 |
| chr10_AA_01591 | hypothetical protein AGABI2DRAFT_179892 [Agaricus bisporus var. bisporus H97] &gt;EKV45447.1 hypothetical protein AGABI2DRAFT_179892 [Agaricus bisporus var. bisporus H97] | 1.36 |
| chr03_AA_00240 | hypothetical protein CYLTODRAFT_426591 [Cylindrobasidium torrendii FP15055 ss-10] | 1.36 |
| chr01_AA_00121 | hypothetical protein CYLTODRAFT_28398 [Cylindrobasidium torrendii FP15055 ss-10] | 1.36 |
| chr06_AA_00927 | Clavaminate synthase-like protein [Armillaria solidipes] | 1.36 |
| chr07_AA_00159 | FAD-binding domain-containing protein [Armillaria solidipes] | 1.35 |
| chr11_AA_01664 | hypothetical protein CYLTODRAFT_421876 [Cylindrobasidium torrendii FP15055 ss-10] | 1.35 |
| chr07_AA_00772 | related to Allantoinase [Armillaria ostoyae] | 1.35 |
| chr10_AA_00517 | hypothetical protein CYLTODRAFT_256211 [Cylindrobasidium torrendii FP15055 ss-10] | 1.34 |
| chr11_AA_00638 | hypothetical protein ARMSODRAFT_953107 [Armillaria solidipes] | 1.34 |
| chr11_AA_00534 | sugar transporter [Cylindrobasidium torrendii FP15055 ss-10] | 1.34 |
| chr07_AA_01096 | hypothetical protein CYLTODRAFT_212928 [Cylindrobasidium torrendii FP15055 ss-10] | 1.33 |
| chr05_AA_00156 | hypothetical protein Moror_2973 [Moniliophthora roreri MCA 2997] &gt;KTB44036.1 hypothetical protein WG66_3386 [Moniliophthora roreri] | 1.33 |
| chr06_AA_00669 | glycoside hydrolase family 47 protein [Cylindrobasidium torrendii FP15055 ss-10] | 1.32 |
| chr08_AA_00900 | UPF0103-domain-containing protein [Cylindrobasidium torrendii FP15055 ss-10] | 1.32 |
| chr11_AA_00896 | carboxyl transferase [Cylindrobasidium torrendii FP15055 ss-10] | 1.32 |
| chr08_AA_01038 | hypothetical protein CYLTODRAFT_424885 [Cylindrobasidium torrendii FP15055 ss-10] | 1.32 |
| chr09_AA_01171 | uncharacterized protein ARMOST_13025 [Armillaria ostoyae] | 1.31 |
| chr10_AA_00746 | hypothetical protein CYLTODRAFT_419225 [Cylindrobasidium torrendii FP15055 ss-10] | 1.31 |
| chr11_AA_00819 | SAICAR synthase-like protein [Cylindrobasidium torrendii FP15055 ss-10] | 1.31 |
| chr01_AA_00277 | OPT superfamily oligopeptide transporter [Cylindrobasidium torrendii FP15055 ss-10] | 1.31 |
| chr09_AA_00394 | cytochrome P450 [Armillaria gallica] | 1.31 |
| chr08_AA_00375 | fumble [Armillaria gallica] | 1.30 |
| chr07_AA_00410 | cytochrome P450 [Cylindrobasidium torrendii FP15055 ss-10] | 1.30 |
| chr08_AA_00333 | hypothetical protein ARMGADRAFT_916371 [Armillaria gallica] | 1.30 |
| chr10_AA_00790 | hypothetical protein CYLTODRAFT_392047 [Cylindrobasidium torrendii FP15055 ss-10] | 1.30 |
| chr06_AA_01002 | Pkinase-domain-containing protein [Cylindrobasidium torrendii FP15055 ss-10] | 1.30 |
| chr01_AA_00265 | hypothetical protein CYLTODRAFT_383163 [Cylindrobasidium torrendii FP15055 ss-10] | 1.29 |
| chr09_AA_00874 | hypothetical protein CYLTODRAFT_419025 [Cylindrobasidium torrendii FP15055 ss-10] | 1.29 |
| chr04_AA_00507 | NA | 1.27 |
| chr09_AA_01004 | proline iminopeptidase [Cylindrobasidium torrendii FP15055 ss-10] | 1.26 |
| chr08_AA_01027 | hypothetical protein CYLTODRAFT_357890 [Cylindrobasidium torrendii FP15055 ss-10] | 1.26 |
| chr09_AA_00913 | general substrate transporter [Armillaria solidipes] | 1.26 |
| chr07_AA_00328 | PLP-dependent transferase [Cylindrobasidium torrendii FP15055 ss-10] | 1.26 |
| chr02_AA_00144 | hexokinase [Cylindrobasidium torrendii FP15055 ss-10] | 1.26 |
| chr11_AA_01530 | uncharacterized protein ARMOST_13587 [Armillaria ostoyae] | 1.26 |
| chr07_AA_00756 | SAICAR synthase-like protein [Cylindrobasidium torrendii FP15055 ss-10] | 1.25 |
| chr11_AA_00784 | NAD(P)-binding protein [Armillaria solidipes] | 1.24 |
| chr09_AA_00352 | ClpP/crotonase [Cylindrobasidium torrendii FP15055 ss-10] | 1.24 |
| chr06_AA_00378 | hypothetical protein CYLTODRAFT_421179 [Cylindrobasidium torrendii FP15055 ss-10] | 1.24 |
| chr04_AA_00750 | hypothetical protein PILCRDRAFT_811913 [Piloderma croceum F 1598] | 1.24 |
| chr11_AA_00795 | NAD-P-binding protein [Cylindrobasidium torrendii FP15055 ss-10] | 1.24 |
| chr09_AA_00758 | NA | 1.23 |
| chr09_AA_00134 | Extradiol ring-cleavage dioxygenase class III enzyme subunit B [Cylindrobasidium torrendii FP15055 ss-10] | 1.23 |
| chr02_AA_00058 | hypothetical protein ARMSODRAFT_706237 [Armillaria solidipes] &gt;SJL01655.1 uncharacterized protein ARMOST_04978 [Armillaria ostoyae] | 1.23 |
| chr03_AA_00298 | hypothetical protein CYLTODRAFT_458738 [Cylindrobasidium torrendii FP15055 ss-10] | 1.23 |
| chr03_AA_00227 | NAD-P-binding protein [Armillaria solidipes] | 1.22 |
| chr11_AA_01710 | hypothetical protein K443DRAFT_681953 [Laccaria amethystina LaAM-08-1] | 1.22 |
| chr06_AA_00250 | hypothetical protein CYLTODRAFT_374506 [Cylindrobasidium torrendii FP15055 ss-10] | 1.22 |
| chr06_AA_00511 | hypothetical protein CYLTODRAFT_487823 [Cylindrobasidium torrendii FP15055 ss-10] | 1.22 |
| chr06_AA_00736 | hypothetical protein CYLTODRAFT_486816 [Cylindrobasidium torrendii FP15055 ss-10] | 1.22 |
| chr09_AA_00229 | succinyl-CoA:3-ketoacid-coenzyme A transferase [Cylindrobasidium torrendii FP15055 ss-10] | 1.21 |
| chr11_AA_01510 | hypothetical protein ARMGADRAFT_1015707 [Armillaria gallica] | 1.21 |
| chr09_AA_01248 | hypothetical protein WOLCODRAFT_120682 [Wolfiporia cocos MD-104 SS10] | 1.21 |
| chr07_AA_00722 | hypothetical protein K443DRAFT_93998 [Laccaria amethystina LaAM-08-1] | 1.21 |
| chr01_AA_00482 | hypothetical protein ARMGADRAFT_1083600 [Armillaria gallica] | 1.20 |
| chr08_AA_00786 | NA | 1.20 |
| chr10_AA_00693 | hypothetical protein ARMSODRAFT_947720 [Armillaria solidipes] | 1.19 |
| chr03_AA_00407 | NA | 1.19 |
| chr11_AA_01042 | acetoin reductase family protein [Cylindrobasidium torrendii FP15055 ss-10] | 1.18 |
| chr03_AA_00170 | zf-CSL-domain-containing protein [Armillaria solidipes] &gt;SJL06478.1 related to Diphthamide biosynthesis protein 3 [Armillaria ostoyae] | 1.18 |
| chr08_AA_00040 | hypothetical protein ARMSODRAFT_958370 [Armillaria solidipes] | 1.18 |
| chr09_AA_00518 | AAA-domain-containing protein [Cylindrobasidium torrendii FP15055 ss-10] | 1.18 |
| chr09_AA_00241 | SNF2 chromatin remodeling protein [Armillaria solidipes] | 1.17 |
| chr05_AA_00197 | heterotrimeric G protein alpha subunit C [Cylindrobasidium torrendii FP15055 ss-10] | 1.16 |
| chr01_AA_00457 | hypothetical protein M422DRAFT_775760, partial [Sphaerobolus stellatus SS14] | 1.16 |
| chr10_AA_00573 | WD40 repeat-like protein [Cylindrobasidium torrendii FP15055 ss-10] | 1.15 |
| chr11_AA_00039 | NA | 1.15 |
| chr07_AA_00676 | hypothetical protein CYLTODRAFT_397668 [Cylindrobasidium torrendii FP15055 ss-10] | 1.14 |
| chr11_AA_00702 | acetoacetyl-CoA synthetase [Cylindrobasidium torrendii FP15055 ss-10] | 1.13 |
| chr05_AA_00421 | ribosomal protein S5 domain 2-like protein [Cylindrobasidium torrendii FP15055 ss-10] | 1.13 |
| chr11_AA_00826 | uncharacterized protein ARMOST_05512 [Armillaria ostoyae] | 1.13 |
| chr06_AA_00682 | hypothetical protein HYDPIDRAFT_112868 [Hydnomerulius pinastri MD-312] | 1.12 |
| chr10_AA_01365 | NA | 1.12 |
| chr07_AA_00288 | uncharacterized protein ARMOST_09162 [Armillaria ostoyae] | 1.10 |
| chr07_AA_00380 | ectomycorrhiza-regulated esterase [Armillaria solidipes] | 1.09 |
| chr11_AA_01431 | hypothetical protein CYLTODRAFT_369482 [Cylindrobasidium torrendii FP15055 ss-10] | 1.09 |
| chr10_AA_00353 | hypothetical protein CYLTODRAFT_418182 [Cylindrobasidium torrendii FP15055 ss-10] | 1.08 |
| chr07_AA_00675 | multidrug resistance protein 1 [Armillaria gallica] | 1.08 |
| chr08_AA_00413 | hypothetical protein ARMGADRAFT_391312 [Armillaria gallica] | 1.02 |

The first two rows represent gene ID and gene annotation respectively. "NA" represents no annottaion. The third row represents the value of log2(fold change) derived from average value of 1099 differentially expressed genes shared in three treated groups compared to the control group.

Supplementary Table S4. Information of 385 down-regulated genes shared in the three treated groups S1, S2 and S3 compared to the CK group.

| Gene ID | Annotation | Log2 (fold change) |
| --- | --- | --- |
| chr08_AA_00201 | uncharacterized protein ARMOST_11116 [Armillaria ostoyae] | -5.72 |
| chr09_AA_00526 | NA | -5.41 |
| chr10_AA_00645 | hypothetical protein ARMGADRAFT_1165285 [Armillaria gallica] | -5.21 |
| chr07_AA_01075 | hypothetical protein CYLTODRAFT_477482 [Cylindrobasidium torrendii FP15055 ss-10] | -4.85 |
| chr11_AA_01325 | short chain type [Moniliophthora roreri MCA 2997] | -4.79 |
| chr03_AA_00311 | related to glycerol-3-phosphate dehydrogenase (NAD) [Armillaria ostoyae] | -4.77 |
| chr10_AA_01530 | NA | -4.60 |
| chr10_AA_01528 | hypothetical protein CYLTODRAFT_446046 [Cylindrobasidium torrendii FP15055 ss-10] | -4.54 |
| chr09_AA_00682 | hypothetical protein CY34DRAFT_417575 [Suillus luteus UH-Slu-Lm8-n1] | -4.45 |
| chr09_AA_01180 | related to Enoyl-CoA hydratase [Armillaria ostoyae] | -4.30 |
| chr10_AA_00930 | NA | -4.23 |
| chr03_AA_00312 | related to HOR2-DL-glycerol phosphatase [Armillaria ostoyae] | -4.21 |
| chr07_AA_00498 | NA | -4.10 |
| chr02_AA_00198 | NA | -4.06 |
| chr07_AA_01168 | putative B-(1-6) glucan synthase [Flammulina velutipes] | -4.01 |
| chr08_AA_00174 | NA | -3.92 |
| chr07_AA_00507 | NA | -3.85 |
| chr06_AA_00697 | glycoside hydrolase family 71 protein [Cylindrobasidium torrendii FP15055 ss-10] | -3.84 |
| chr04_AA_00460 | NA | -3.84 |
| chr05_AA_00280 | NA | -3.78 |
| chr06_AA_00240 | hypothetical protein GYMLUDRAFT_279025 [Gymnopus luxurians FD-317 M1] | -3.68 |
| chr05_AA_00688 | terpenoid synthase [Moniliophthora roreri MCA 2997] | -3.66 |
| chr03_AA_00235 | NA | -3.56 |
| chr10_AA_01262 | hypothetical protein AURDEDRAFT_175315 [Auricularia subglabra TFB-10046 SS5] | -3.55 |
| chr08_AA_01320 | hexose transporter [Cylindrobasidium torrendii FP15055 ss-10] | -3.53 |
| chr09_AA_00374 | peptidyl-Lys metalloendopeptidase [Armillaria gallica] | -3.47 |
| chr10_AA_00637 | hypothetical protein CYLTODRAFT_492301 [Cylindrobasidium torrendii FP15055 ss-10] | -3.39 |
| chr11_AA_00305 | NA | -3.39 |
| chr09_AA_00579 | acid phosphatase/Vanadium-dependent haloperoxidase [Armillaria gallica] | -3.37 |
| chr09_AA_01273 | aspartic peptidase A1 [Armillaria gallica] | -3.35 |
| chr07_AA_00182 | NA | -3.33 |
| chr03_AA_00446 | NA | -3.33 |
| chr08_AA_00113 | uncharacterized protein ARMOST_11486 [Armillaria ostoyae] | -3.32 |
| chr09_AA_01215 | calcium/proton exchanger [Cylindrobasidium torrendii FP15055 ss-10] | -3.32 |
| chr08_AA_00515 | hypothetical protein ARMSODRAFT_244836 [Armillaria solidipes] | -3.30 |
| chr06_AA_00371 | hypothetical protein ARMGADRAFT_1162048 [Armillaria gallica] | -3.30 |
| chr11_AA_00068 | hypothetical protein Moror_972 [Moniliophthora roreri MCA 2997] &gt;KTB32292.1 hypothetical protein WG66_15142 [Moniliophthora roreri] | -3.27 |
| chr02_AA_00161 | hypothetical protein ARMSODRAFT_1002098 [Armillaria solidipes] | -3.24 |
| chr06_AA_00608 | glycoside hydrolase family 13 protein [Armillaria gallica] | -3.21 |
| chr10_AA_00976 | uncharacterized protein ARMOST_06141 [Armillaria ostoyae] | -3.16 |
| chr05_AA_00546 | hypothetical protein CYLTODRAFT_435337 [Cylindrobasidium torrendii FP15055 ss-10] | -3.16 |
| chr08_AA_00514 | hypothetical protein ARMGADRAFT_1159913 [Armillaria gallica] | -3.13 |
| chr11_AA_01351 | ankyrin [Armillaria solidipes] | -3.13 |
| chr04_AA_00707 | uncharacterized protein ARMOST_14599 [Armillaria ostoyae] | -3.12 |
| chr01_AA_00067 | metallo peptidase M36 [Flammulina velutipes] | -3.09 |
| chr01_AA_00285 | NA | -3.04 |
| chr10_AA_01055 | delta 9-fatty acid desaturase protein [Armillaria solidipes] | -3.01 |
| chr09_AA_00344 | related to SnodProt1 precursor [Armillaria ostoyae] | -3.00 |
| chr04_AA_00676 | hypothetical protein CYLTODRAFT_427270 [Cylindrobasidium torrendii FP15055 ss-10] | -3.00 |
| chr05_AA_00180 | NA | -2.99 |
| chr02_AA_00063 | NA | -2.99 |
| chr09_AA_00247 | carboxylic acid transporter protein [Cylindrobasidium torrendii FP15055 ss-10] | -2.98 |
| chr08_AA_00516 | hypothetical protein ARMSODRAFT_948657 [Armillaria solidipes] | -2.96 |
| chr11_AA_01388 | MFS general substrate transporter [Armillaria gallica] | -2.96 |
| chr10_AA_00610 | MFS general substrate transporter [Armillaria solidipes] | -2.95 |
| chr06_AA_00042 | putative nitrilase [Cylindrobasidium torrendii FP15055 ss-10] | -2.92 |
| chr08_AA_00325 | hypothetical protein ARMSODRAFT_874491 [Armillaria solidipes] | -2.91 |
| chr10_AA_00351 | lysine/ornithine N-monooxygenase [Cylindrobasidium torrendii FP15055 ss-10] | -2.91 |
| chr11_AA_01512 | cytochrome P450 [Armillaria solidipes] | -2.89 |
| chr08_AA_00388 | NA | -2.84 |
| chr11_AA_00688 | hypothetical protein CYLTODRAFT_365132 [Cylindrobasidium torrendii FP15055 ss-10] | -2.82 |
| chr06_AA_00591 | GMC oxidoreductase [Cylindrobasidium torrendii FP15055 ss-10] | -2.82 |
| chr05_AA_00859 | hypothetical protein ARMGADRAFT_1075799 [Armillaria gallica] | -2.81 |
| chr05_AA_00905 | putative endo-1,4-beta-xylanase precursor [Flammulina velutipes] | -2.81 |
| chr03_AA_00025 | hypothetical protein M413DRAFT_59902 [Hebeloma cylindrosporum h7] | -2.78 |
| chr06_AA_00714 | NAD(P)-binding protein [Cylindrobasidium torrendii FP15055 ss-10] | -2.78 |
| chr06_AA_00228 | NA | -2.77 |
| chr01_AA_00443 | hypothetical protein GYMLUDRAFT_35183 [Gymnopus luxurians FD-317 M1] | -2.77 |
| chr09_AA_01311 | phosphate permease [Cylindrobasidium torrendii FP15055 ss-10] | -2.77 |
| chr11_AA_00864 | uncharacterized protein ARMOST_05667 [Armillaria ostoyae] | -2.76 |
| chr06_AA_00753 | MFS general substrate transporter [Cylindrobasidium torrendii FP15055 ss-10] | -2.76 |
| chr08_AA_00041 | NA | -2.75 |
| chr10_AA_00510 | MFS general substrate transporter [Armillaria solidipes] | -2.68 |
| chr07_AA_00857 | uncharacterized protein ARMOST_01010 [Armillaria ostoyae] | -2.66 |
| chr05_AA_00157 | aromatic peroxygenase [Rhizoctonia solani AG-3 Rhs1AP] | -2.65 |
| chr11_AA_01738 | hypothetical protein PHLGIDRAFT_119585 [Phlebiopsis gigantea 11061_1 CR5-6] | -2.64 |
| chr08_AA_00247 | NA | -2.63 |
| chr08_AA_00169 | TPR-like protein [Armillaria solidipes] | -2.61 |
| chr09_AA_01310 | agmatinase [Armillaria gallica] | -2.61 |
| chr05_AA_00295 | glycoside hydrolase [Armillaria gallica] | -2.60 |
| chr10_AA_00695 | hypothetical protein GALMADRAFT_232387 [Galerina marginata CBS 339.88] | -2.60 |
| chr10_AA_00379 | hypothetical protein ARMSODRAFT_947234 [Armillaria solidipes] | -2.58 |
| chr05_AA_00660 | short-chain dehydrogenase [Armillaria gallica] | -2.57 |
| chr10_AA_01391 | FMN-linked oxidoreductase [Armillaria solidipes] | -2.52 |
| chr09_AA_00345 | related to SnodProt1 precursor [Armillaria ostoyae] | -2.52 |
| chr06_AA_00103 | 60S ribosomal protein L37 [Coprinopsis cinerea okayama7#130] &gt;EAU89987.1 ribosomal protein L37e [Coprinopsis cinerea okayama7#130] | -2.51 |
| chr09_AA_01175 | kinase-like protein [Armillaria solidipes] | -2.50 |
| chr01_AA_00489 | related to synaptic vesicle transporter SVOP and related transporters (major facilitator superfamily) [Armillaria ostoyae] | -2.49 |
| chr02_AA_00381 | NA | -2.49 |
| chr11_AA_01372 | uncharacterized protein ARMOST_10662 [Armillaria ostoyae] | -2.48 |
| chr02_AA_00162 | cytochrome P450 [Armillaria solidipes] | -2.46 |
| chr11_AA_00526 | uncharacterized protein ARMOST_05138 [Armillaria ostoyae] | -2.46 |
| chr06_AA_00249 | probable ribonucleoside-diphosphate reductase small chain [Armillaria ostoyae] | -2.45 |
| chr09_AA_00974 | hypothetical protein CYLTODRAFT_422665 [Cylindrobasidium torrendii FP15055 ss-10] | -2.45 |
| chr04_AA_00633 | NA | -2.44 |
| chr10_AA_01207 | permease of the major facilitator superfamily [Cylindrobasidium torrendii FP15055 ss-10] | -2.43 |
| chr03_AA_00142 | NA | -2.42 |
| chr11_AA_01511 | related to cytochrome P450 CYP2 subfamily [Armillaria ostoyae] | -2.40 |
| chr09_AA_00731 | NA | -2.40 |
| chr06_AA_00668 | putative peptide transporter ptr2 [Hypsizygus marmoreus] | -2.39 |
| chr08_AA_00278 | hypothetical protein ARMGADRAFT_995516 [Armillaria gallica] | -2.34 |
| chr06_AA_01007 | NA | -2.33 |
| chr11_AA_01366 | hypothetical protein HYDPIDRAFT_27225 [Hydnomerulius pinastri MD-312] | -2.32 |
| chr07_AA_00918 | hypothetical protein CYLTODRAFT_421928 [Cylindrobasidium torrendii FP15055 ss-10] | -2.32 |
| chr05_AA_00497 | hypothetical protein ARMSODRAFT_492082 [Armillaria solidipes] | -2.29 |
| chr06_AA_00752 | MFS general substrate transporter [Cylindrobasidium torrendii FP15055 ss-10] | -2.29 |
| chr07_AA_00407 | uncharacterized protein ARMOST_00605 [Armillaria ostoyae] | -2.29 |
| chr11_AA_01624 | aquaporin [Armillaria gallica] | -2.29 |
| chr01_AA_00045 | hypothetical protein CYLTODRAFT_387229 [Cylindrobasidium torrendii FP15055 ss-10] | -2.27 |
| chr07_AA_00113 | uncharacterized protein ARMOST_12460 [Armillaria ostoyae] | -2.27 |
| chr07_AA_01074 | hypothetical protein CYLTODRAFT_356116 [Cylindrobasidium torrendii FP15055 ss-10] | -2.25 |
| chr11_AA_01323 | hypothetical protein ARMSODRAFT_981381 [Armillaria solidipes] | -2.23 |
| chr11_AA_00656 | NA | -2.22 |
| chr01_AA_00499 | hypothetical protein ARMSODRAFT_961651 [Armillaria solidipes] | -2.21 |
| chr05_AA_00685 | related to Cytidine deaminase [Armillaria ostoyae] | -2.20 |
| chr06_AA_00948 | hypothetical protein ARMGADRAFT_985773 [Armillaria gallica] | -2.20 |
| chr08_AA_01133 | uncharacterized protein ARMOST_08917 [Armillaria ostoyae] | -2.19 |
| chr07_AA_00295 | P-loop containing nucleoside triphosphate hydrolase protein [Cylindrobasidium torrendii FP15055 ss-10] | -2.18 |
| chr03_AA_00518 | putative pheromone receptor [Flammulina velutipes] | -2.18 |
| chr09_AA_00723 | Indoleamine 2,3-dioxygenase [Armillaria gallica] | -2.18 |
| chr07_AA_01015 | uncharacterized protein ARMOST_16245 [Armillaria ostoyae] | -2.17 |
| chr07_AA_00450 | hypothetical protein CYLTODRAFT_445037 [Cylindrobasidium torrendii FP15055 ss-10] | -2.17 |
| chr11_AA_01739 | NA | -2.16 |
| chr09_AA_00263 | NAD(P)-binding protein [Cylindrobasidium torrendii FP15055 ss-10] | -2.16 |
| chr07_AA_00148 | hypothetical protein CYLTODRAFT_427081 [Cylindrobasidium torrendii FP15055 ss-10] | -2.16 |
| chr08_AA_00142 | NA | -2.15 |
| chr05_AA_00279 | hypothetical protein ARMSODRAFT_951210 [Armillaria solidipes] | -2.15 |
| chr10_AA_01388 | FMN-linked oxidoreductase [Armillaria gallica] | -2.15 |
| chr05_AA_00642 | NA | -2.14 |
| chr06_AA_00794 | heme peroxidase, partial [Cylindrobasidium torrendii FP15055 ss-10] | -2.14 |
| chr04_AA_00463 | related to tropinone reductase [Armillaria ostoyae] | -2.12 |
| chr07_AA_00639 | hypothetical protein ARMSODRAFT_952112 [Armillaria solidipes] | -2.11 |
| chr09_AA_00412 | hypothetical protein CYLTODRAFT_413158 [Cylindrobasidium torrendii FP15055 ss-10] | -2.09 |
| chr01_AA_00485 | NA | -2.08 |
| chr02_AA_00281 | NA | -2.08 |
| chr05_AA_00608 | kinase-like protein [Cylindrobasidium torrendii FP15055 ss-10] | -2.07 |
| chr10_AA_01506 | putative cytochrome P450 [Moniliophthora roreri] | -2.07 |
| chr09_AA_01221 | uncharacterized protein ARMOST_12972 [Armillaria ostoyae] | -2.07 |
| chr05_AA_00292 | carbohydrate esterase family 16 protein [Cylindrobasidium torrendii FP15055 ss-10] | -2.05 |
| chr11_AA_01672 | uncharacterized protein ARMOST_16352 [Armillaria ostoyae] | -2.05 |
| chr03_AA_00244 | NAD(P)-binding protein [Armillaria solidipes] | -2.05 |
| chr01_AA_00161 | hypothetical protein ARMGADRAFT_622034 [Armillaria gallica] | -2.04 |
| chr01_AA_00456 | NA | -2.03 |
| chr10_AA_01287 | uncharacterized protein ARMOST_14737 [Armillaria ostoyae] | -2.02 |
| chr07_AA_01116 | NA | -2.02 |
| chr01_AA_00122 | polyamine transporter [Cylindrobasidium torrendii FP15055 ss-10] | -2.02 |
| chr07_AA_00902 | hypothetical protein ARMSODRAFT_951934 [Armillaria solidipes] | -2.01 |
| chr08_AA_00647 | hypothetical protein CYLTODRAFT_490559 [Cylindrobasidium torrendii FP15055 ss-10] | -1.99 |
| chr10_AA_00592 | hypothetical protein ARMGADRAFT_827535 [Armillaria gallica] | -1.98 |
| chr11_AA_00975 | hypothetical protein ARMSODRAFT_996151 [Armillaria solidipes] | -1.98 |
| chr08_AA_00203 | Caleosin-domain-containing protein [Cylindrobasidium torrendii FP15055 ss-10] | -1.97 |
| chr10_AA_01556 | NA | -1.97 |
| chr10_AA_01105 | hypothetical protein ARMGADRAFT_1014692 [Armillaria gallica] | -1.97 |
| chr03_AA_00681 | uncharacterized protein ARMOST_04080 [Armillaria ostoyae] | -1.97 |
| chr10_AA_00146 | histon H1 [Flammulina velutipes] | -1.97 |
| chr10_AA_00636 | hypothetical protein CYLTODRAFT_423535 [Cylindrobasidium torrendii FP15055 ss-10] | -1.97 |
| chr11_AA_00091 | hypothetical protein ARMGADRAFT_1070575 [Armillaria gallica] | -1.96 |
| chr10_AA_00496 | hypothetical protein CYLTODRAFT_417203 [Cylindrobasidium torrendii FP15055 ss-10] | -1.96 |
| chr09_AA_00339 | hypothetical protein CYLTODRAFT_489487 [Cylindrobasidium torrendii FP15055 ss-10] | -1.96 |
| chr10_AA_00213 | hypothetical protein Moror_6438 [Moniliophthora roreri MCA 2997] | -1.96 |
| chr08_AA_01019 | probable DNA repair protein RAD51 [Armillaria ostoyae] | -1.96 |
| chr10_AA_01272 | hypothetical protein GYMLUDRAFT_88395 [Gymnopus luxurians FD-317 M1] | -1.95 |
| chr11_AA_00060 | uncharacterized protein ARMOST_02888 [Armillaria ostoyae] | -1.95 |
| chr11_AA_00071 | NA | -1.94 |
| chr08_AA_00188 | hypothetical protein HYPSUDRAFT_50744 [Hypholoma sublateritium FD-334 SS-4] | -1.94 |
| chr05_AA_00641 | cytochrome P450 [Cylindrobasidium torrendii FP15055 ss-10] | -1.94 |
| chr10_AA_00054 | uncharacterized protein ARMOST_05030 [Armillaria ostoyae] | -1.94 |
| chr07_AA_01072 | hypothetical protein ARMGADRAFT_1168847 [Armillaria gallica] | -1.93 |
| chr03_AA_00328 | hypothetical protein SCHCODRAFT_231913 [Schizophyllum commune H4-8] &gt;EFJ02757.1 hypothetical protein SCHCODRAFT_231913 [Schizophyllum commune H4-8] | -1.93 |
| chr11_AA_00631 | hypothetical protein ARMGADRAFT_990224 [Armillaria gallica] | -1.92 |
| chr11_AA_00225 | NA | -1.92 |
| chr11_AA_00985 | hypothetical protein ARMSODRAFT_956744 [Armillaria solidipes] | -1.92 |
| chr10_AA_00631 | sphingolipid C9-methyltransferase [Cylindrobasidium torrendii FP15055 ss-10] | -1.92 |
| chr03_AA_00711 | uncharacterized protein ARMOST_06712 [Armillaria ostoyae] | -1.92 |
| chr01_AA_00235 | related to DNA helicase [Armillaria ostoyae] | -1.92 |
| chr09_AA_00193 | hypothetical protein CYLTODRAFT_317886, partial [Cylindrobasidium torrendii FP15055 ss-10] | -1.91 |
| chr10_AA_01116 | hypothetical protein ARMGADRAFT_305201 [Armillaria gallica] | -1.91 |
| chr05_AA_00527 | hypothetical protein NEOLEDRAFT_1026610, partial [Neolentinus lepideus HHB14362 ss-1] | -1.91 |
| chr01_AA_00344 | hypothetical protein ARMGADRAFT_1167792 [Armillaria gallica] | -1.90 |
| chr08_AA_00340 | NA | -1.90 |
| chr08_AA_00606 | hypothetical protein CYLTODRAFT_489146 [Cylindrobasidium torrendii FP15055 ss-10] | -1.88 |
| chr09_AA_00225 | CDC45-like protein [Cylindrobasidium torrendii FP15055 ss-10] | -1.88 |
| chr10_AA_01128 | uncharacterized protein ARMOST_02528 [Armillaria ostoyae] | -1.87 |
| chr08_AA_01004 | hypothetical protein HYPSUDRAFT_32859 [Hypholoma sublateritium FD-334 SS-4] | -1.87 |
| chr06_AA_00265 | NA | -1.87 |
| chr10_AA_01577 | uncharacterized protein ARMOST_14840 [Armillaria ostoyae] | -1.86 |
| chr11_AA_01659 | hypothetical protein CYLTODRAFT_423687 [Cylindrobasidium torrendii FP15055 ss-10] | -1.85 |
| chr11_AA_00876 | hypothetical protein CYLTODRAFT_215242 [Cylindrobasidium torrendii FP15055 ss-10] | -1.85 |
| chr07_AA_00552 | hypothetical protein PLEOSDRAFT_1091553 [Pleurotus ostreatus PC15] | -1.84 |
| chr08_AA_00883 | related to DPS1-aspartyl-tRNA synthetase, cytosolic [Armillaria ostoyae] | -1.84 |
| chr05_AA_00303 | hypothetical protein CYLTODRAFT_202455 [Cylindrobasidium torrendii FP15055 ss-10] | -1.84 |
| chr08_AA_00465 | hypothetical protein ARMSODRAFT_1078744 [Armillaria solidipes] | -1.83 |
| chr07_AA_01065 | hypothetical protein ARMGADRAFT_1017035 [Armillaria gallica] | -1.83 |
| chr03_AA_00199 | NA | -1.82 |
| chr07_AA_01014 | hypothetical protein CYLTODRAFT_494738 [Cylindrobasidium torrendii FP15055 ss-10] | -1.82 |
| chr11_AA_00852 | related to homoserine O-acetyltransferase [Armillaria ostoyae] | -1.81 |
| chr05_AA_00493 | mitochondrial carrier [Cylindrobasidium torrendii FP15055 ss-10] | -1.79 |
| chr11_AA_00226 | hypothetical protein LENED_000613 [Lentinula edodes] | -1.77 |
| chr06_AA_00340 | uncharacterized protein ARMOST_06868 [Armillaria ostoyae] | -1.77 |
| chr06_AA_00743 | uncharacterized protein ARMOST_17512 [Armillaria ostoyae] | -1.76 |
| chr06_AA_00533 | aldo-keto reductase [Mycena chlorophos] | -1.75 |
| chr07_AA_00207 | uncharacterized protein ARMOST_06588 [Armillaria ostoyae] | -1.74 |
| chr11_AA_00126 | hypothetical protein CVT25_006991 [Psilocybe cyanescens] | -1.73 |
| chr05_AA_00444 | uncharacterized protein ARMOST_10896 [Armillaria ostoyae] | -1.73 |
| chr09_AA_01314 | uncharacterized protein ARMOST_03305 [Armillaria ostoyae] | -1.72 |
| chr07_AA_00424 | putative chitin synthase [Flammulina velutipes] | -1.72 |
| chr05_AA_00196 | uncharacterized protein ARMOST_11068 [Armillaria ostoyae] | -1.71 |
| chr10_AA_00364 | related to RAD54-DNA-dependent ATPase of the Snf2p family [Armillaria ostoyae] | -1.71 |
| chr11_AA_00678 | hypothetical protein ARMSODRAFT_931426 [Armillaria solidipes] | -1.71 |
| chr10_AA_00729 | septin ring protein [Cylindrobasidium torrendii FP15055 ss-10] | -1.70 |
| chr04_AA_00269 | NA | -1.70 |
| chr10_AA_00688 | MFS general substrate transporter [Stereum hirsutum FP-91666 SS1] &gt;EIM91397.1 MFS general substrate transporter [Stereum hirsutum FP-91666 SS1] | -1.70 |
| chr08_AA_00700 | hypothetical protein CYLTODRAFT_455891 [Cylindrobasidium torrendii FP15055 ss-10] | -1.70 |
| chr11_AA_00092 | kinesin-domain-containing protein [Cylindrobasidium torrendii FP15055 ss-10] | -1.69 |
| chr07_AA_00480 | hypothetical protein CVT25_015028 [Psilocybe cyanescens] | -1.69 |
| chr09_AA_00967 | related to fatty acid synthase, beta and alpha chains [Armillaria ostoyae] | -1.69 |
| chr08_AA_00920 | hypothetical protein ARMSODRAFT_1079374 [Armillaria solidipes] | -1.68 |
| chr08_AA_00952 | hypothetical protein ARMSODRAFT_949138 [Armillaria solidipes] | -1.68 |
| chr03_AA_00676 | primordium development defect 1 [Flammulina velutipes] | -1.68 |
| chr03_AA_00098 | hypothetical protein CYLTODRAFT_318169, partial [Cylindrobasidium torrendii FP15055 ss-10] | -1.68 |
| chr10_AA_01311 | putative histone H2A-1 [Cylindrobasidium torrendii FP15055 ss-10] | -1.68 |
| chr07_AA_00133 | PALP-domain-containing protein [Cylindrobasidium torrendii FP15055 ss-10] | -1.67 |
| chr05_AA_00404 | cytochrome P450 [Armillaria solidipes] | -1.67 |
| chr01_AA_00416 | hypothetical protein CYLTODRAFT_388873 [Cylindrobasidium torrendii FP15055 ss-10] | -1.67 |
| chr11_AA_01533 | hypothetical protein AMATHDRAFT_60323 [Amanita thiersii Skay4041] | -1.66 |
| chr01_AA_00163 | putative laccase 2 [Flammulina velutipes] | -1.65 |
| chr09_AA_00561 | cytochrome P450 [Armillaria gallica] | -1.65 |
| chr08_AA_00747 | NA | -1.65 |
| chr03_AA_00234 | Aldo/keto reductase [Armillaria gallica] | -1.65 |
| chr05_AA_00250 | hypothetical protein STEHIDRAFT_98286 [Stereum hirsutum FP-91666 SS1] &gt;EIM85978.1 hypothetical protein STEHIDRAFT_98286 [Stereum hirsutum FP-91666 SS1] | -1.65 |
| chr08_AA_00402 | hypothetical protein CYLTODRAFT_416993 [Cylindrobasidium torrendii FP15055 ss-10] | -1.65 |
| chr09_AA_00430 | FAD/NAD(P)-binding domain-containing protein [Armillaria gallica] | -1.65 |
| chr07_AA_00336 | mitochondrial carrier [Armillaria gallica] | -1.64 |
| chr05_AA_00830 | putative pheromone receptor [Flammulina velutipes] | -1.64 |
| chr10_AA_00129 | DUF537-domain-containing protein [Cylindrobasidium torrendii FP15055 ss-10] | -1.64 |
| chr08_AA_01170 | probable ribose-5-phosphate isomerase [Armillaria ostoyae] | -1.63 |
| chr06_AA_00914 | hypothetical protein GYMLUDRAFT_44733 [Gymnopus luxurians FD-317 M1] | -1.63 |
| chr09_AA_01090 | hypothetical protein CYLTODRAFT_408042 [Cylindrobasidium torrendii FP15055 ss-10] | -1.63 |
| chr07_AA_01016 | hypothetical protein CYLTODRAFT_494738 [Cylindrobasidium torrendii FP15055 ss-10] | -1.63 |
| chr07_AA_00726 | hypothetical protein CYLTODRAFT_342910 [Cylindrobasidium torrendii FP15055 ss-10] | -1.63 |
| chr10_AA_01356 | hypothetical protein PLEOSDRAFT_158699 [Pleurotus ostreatus PC15] | -1.63 |
| chr09_AA_00878 | uncharacterized protein ARMOST_13194 [Armillaria ostoyae] | -1.62 |
| chr07_AA_00153 | NA | -1.61 |
| chr08_AA_00014 | putative peroxiredoxin Q [Flammulina velutipes] | -1.61 |
| chr08_AA_00648 | kinesin-domain-containing protein [Cylindrobasidium torrendii FP15055 ss-10] | -1.61 |
| chr11_AA_00693 | alpha-ketoacid dehydrogenase kinase [Armillaria gallica] | -1.60 |
| chr09_AA_00966 | Gtr1/RagA G protein Gtr2 [Cylindrobasidium torrendii FP15055 ss-10] | -1.60 |
| chr06_AA_00664 | hypothetical protein CYLTODRAFT_440959 [Cylindrobasidium torrendii FP15055 ss-10] | -1.60 |
| chr08_AA_00956 | hypothetical protein CYLTODRAFT_389979 [Cylindrobasidium torrendii FP15055 ss-10] | -1.60 |
| chr05_AA_00594 | alpha/beta-hydrolase [Armillaria gallica] | -1.59 |
| chr10_AA_01183 | pleiotropic drug resistance ABC transporter [Cylindrobasidium torrendii FP15055 ss-10] | -1.59 |
| chr10_AA_00285 | hypothetical protein ARMGADRAFT_989033 [Armillaria gallica] | -1.59 |
| chr07_AA_00051 | uncharacterized protein ARMOST_00188 [Armillaria ostoyae] | -1.58 |
| chr10_AA_00629 | hypothetical protein ARMGADRAFT_948479 [Armillaria gallica] | -1.58 |
| chr10_AA_00283 | cytochrome P450 [Cylindrobasidium torrendii FP15055 ss-10] | -1.58 |
| chr11_AA_01063 | RNA-binding domain-containing protein [Cylindrobasidium torrendii FP15055 ss-10] | -1.58 |
| chr09_AA_00277 | hypothetical protein ARMSODRAFT_962094 [Armillaria solidipes] | -1.58 |
| chr08_AA_00599 | DHS-like NAD/FAD-binding domain-containing protein [Armillaria solidipes] | -1.56 |
| chr04_AA_00455 | hypothetical protein CYLTODRAFT_454483 [Cylindrobasidium torrendii FP15055 ss-10] | -1.56 |
| chr10_AA_00528 | 40S ribosomal protein S12 [Armillaria gallica] &gt;SJL00780.1 probable 40S ribosomal protein S12 [Armillaria ostoyae] | -1.54 |
| chr06_AA_00595 | cytosine-5--methyltransferase [Cylindrobasidium torrendii FP15055 ss-10] | -1.54 |
| chr08_AA_00255 | related to NADP+-dependent malic enzyme [Armillaria ostoyae] | -1.54 |
| chr10_AA_00280 | uncharacterized protein ARMOST_03698 [Armillaria ostoyae] | -1.54 |
| chr11_AA_00602 | hypothetical protein CYLTODRAFT_389217 [Cylindrobasidium torrendii FP15055 ss-10] | -1.54 |
| chr10_AA_01472 | histone H2A [Armillaria gallica] | -1.53 |
| chr10_AA_00191 | probable TRP2-anthranilate synthase component I [Armillaria ostoyae] | -1.53 |
| chr08_AA_00116 | hypothetical protein ARMSODRAFT_945289 [Armillaria solidipes] | -1.52 |
| chr03_AA_00106 | hypothetical protein ARMGADRAFT_528752 [Armillaria gallica] | -1.52 |
| chr09_AA_00853 | NA | -1.51 |
| chr06_AA_00641 | related to DNA repair and recombination protein pif1, mitochondrial precursor [Armillaria ostoyae] | -1.51 |
| chr06_AA_00415 | terpenoid synthase [Cylindrobasidium torrendii FP15055 ss-10] | -1.50 |
| chr05_AA_00526 | hypothetical protein CYLTODRAFT_419050 [Cylindrobasidium torrendii FP15055 ss-10] | -1.50 |
| chr05_AA_00420 | hypothetical protein GALMADRAFT_138969 [Galerina marginata CBS 339.88] | -1.50 |
| chr08_AA_00789 | hypothetical protein HYPSUDRAFT_75704 [Hypholoma sublateritium FD-334 SS-4] | -1.49 |
| chr07_AA_00920 | hypothetical protein CYLTODRAFT_399491 [Cylindrobasidium torrendii FP15055 ss-10] | -1.49 |
| chr10_AA_01212 | FAD/NAD(P)-binding domain-containing protein [Cylindrobasidium torrendii FP15055 ss-10] | -1.48 |
| chr08_AA_00887 | translation elongation factor Tu [Lentinula edodes] | -1.48 |
| chr10_AA_00204 | hypothetical protein CYLTODRAFT_493277 [Cylindrobasidium torrendii FP15055 ss-10] | -1.48 |
| chr08_AA_01020 | hypothetical protein ARMGADRAFT_983908 [Armillaria gallica] | -1.47 |
| chr11_AA_00053 | hypothetical protein ARMSODRAFT_300619 [Armillaria solidipes] | -1.47 |
| chr04_AA_00286 | hypothetical protein CYLTODRAFT_452090 [Cylindrobasidium torrendii FP15055 ss-10] | -1.47 |
| chr08_AA_00748 | WD40 repeat-like protein [Cylindrobasidium torrendii FP15055 ss-10] | -1.46 |
| chr05_AA_00348 | UbiE/COQ5 methyltransferase [Cylindrobasidium torrendii FP15055 ss-10] | -1.46 |
| chr05_AA_00305 | alpha/beta-hydrolase [Armillaria solidipes] | -1.45 |
| chr06_AA_00396 | hypothetical protein PLICRDRAFT_56844 [Plicaturopsis crispa FD-325 SS-3] | -1.45 |
| chr11_AA_00304 | hypothetical protein DAEQUDRAFT_660381, partial [Daedalea quercina L-15889] | -1.45 |
| chr06_AA_00636 | hypothetical protein CYLTODRAFT_440945 [Cylindrobasidium torrendii FP15055 ss-10] | -1.45 |
| chr11_AA_01004 | serine peptidase S28 [Flammulina velutipes] | -1.44 |
| chr08_AA_00946 | PIN domain-like protein [Cylindrobasidium torrendii FP15055 ss-10] | -1.44 |
| chr09_AA_00965 | SPX-domain-containing protein [Armillaria gallica] | -1.43 |
| chr07_AA_00870 | HAD-superfamily hydrolase [Armillaria solidipes] | -1.43 |
| chr10_AA_00652 | hypothetical protein M413DRAFT_44499, partial [Hebeloma cylindrosporum h7] | -1.43 |
| chr09_AA_01117 | hypothetical protein CVT26_002629 [Gymnopilus dilepis] | -1.43 |
| chr03_AA_00152 | hypothetical protein CYLTODRAFT_487517 [Cylindrobasidium torrendii FP15055 ss-10] | -1.43 |
| chr10_AA_00691 | uncharacterized protein ARMOST_11173 [Armillaria ostoyae] | -1.42 |
| chr08_AA_00085 | putative peroxiredoxin Q [Flammulina velutipes] | -1.42 |
| chr11_AA_00127 | putative pectate lyase D, partial [Leucoagaricus sp. SymC.cos] | -1.42 |
| chr09_AA_00259 | hypothetical protein ARMSODRAFT_989925 [Armillaria solidipes] &gt;SJL04724.1 uncharacterized protein ARMOST_08094 [Armillaria ostoyae] | -1.42 |
| chr11_AA_01121 | hypothetical protein CYLTODRAFT_488150 [Cylindrobasidium torrendii FP15055 ss-10] | -1.42 |
| chr11_AA_00735 | hypothetical protein PAXINDRAFT_166311 [Paxillus involutus ATCC 200175] | -1.40 |
| chr11_AA_01536 | hypothetical protein Moror_3774 [Moniliophthora roreri MCA 2997] | -1.40 |
| chr07_AA_00652 | putative UTP--glucose-1-phosphate uridylyltransferase [Hypsizygus marmoreus] | -1.39 |
| chr11_AA_01067 | related to PGC1-phosphatidyl glycerol phospholipase C [Armillaria ostoyae] | -1.39 |
| chr08_AA_00987 | cell division control/GTP binding protein [Laccaria bicolor S238N-H82] &gt;EDR15555.1 cell division control/GTP binding protein [Laccaria bicolor S238N-H82] | -1.39 |
| chr08_AA_00922 | GNS1/SUR4 membrane protein, partial [Cylindrobasidium torrendii FP15055 ss-10] | -1.39 |
| chr07_AA_00418 | hypothetical protein AURDEDRAFT_147076 [Auricularia subglabra TFB-10046 SS5] | -1.38 |
| chr09_AA_00254 | PLC-like phosphodiesterase [Armillaria solidipes] | -1.38 |
| chr10_AA_01594 | WD40 repeat-like protein [Cylindrobasidium torrendii FP15055 ss-10] | -1.38 |
| chr09_AA_00533 | Nop domain-containing protein [Armillaria gallica] | -1.38 |
| chr08_AA_00373 | hypothetical protein ARMSODRAFT_969464 [Armillaria solidipes] | -1.38 |
| chr10_AA_01574 | hypothetical protein CYLTODRAFT_492309 [Cylindrobasidium torrendii FP15055 ss-10] | -1.38 |
| chr06_AA_00409 | OPT oligopeptide transporter [Armillaria gallica] | -1.37 |
| chr10_AA_00121 | alpha beta-hydrolase [Armillaria gallica] | -1.37 |
| chr06_AA_00339 | DUF1212-domain-containing protein [Cylindrobasidium torrendii FP15055 ss-10] | -1.37 |
| chr10_AA_00284 | hypothetical protein ARMGADRAFT_1010402 [Armillaria gallica] | -1.37 |
| chr09_AA_01218 | hypothetical protein CYLTODRAFT_388706 [Cylindrobasidium torrendii FP15055 ss-10] | -1.37 |
| chr08_AA_00710 | hypothetical protein ARMGADRAFT_957046 [Armillaria gallica] | -1.36 |
| chr06_AA_00980 | chitin deacetylase [Armillaria gallica] | -1.36 |
| chr10_AA_00262 | hypothetical protein CYLTODRAFT_81489 [Cylindrobasidium torrendii FP15055 ss-10] | -1.35 |
| chr07_AA_00173 | peptidyl-tRNA hydrolase [Cylindrobasidium torrendii FP15055 ss-10] | -1.35 |
| chr11_AA_00197 | hypothetical protein CERSUDRAFT_116953 [Gelatoporia subvermispora B] | -1.34 |
| chr09_AA_01403 | uncharacterized protein ARMOST_01927 [Armillaria ostoyae] | -1.34 |
| chr07_AA_00539 | hypothetical protein CYLTODRAFT_416196 [Cylindrobasidium torrendii FP15055 ss-10] | -1.34 |
| chr09_AA_00891 | hypothetical protein CYLTODRAFT_438015 [Cylindrobasidium torrendii FP15055 ss-10] | -1.34 |
| chr10_AA_00128 | hypothetical protein CYLTODRAFT_427543 [Cylindrobasidium torrendii FP15055 ss-10] | -1.34 |
| chr05_AA_00125 | hypothetical protein ARMGADRAFT_1052356 [Armillaria gallica] | -1.34 |
| chr05_AA_00548 | hypothetical protein CYLTODRAFT_451099 [Cylindrobasidium torrendii FP15055 ss-10] | -1.33 |
| chr05_AA_00450 | hypothetical protein GYMLUDRAFT_74308 [Gymnopus luxurians FD-317 M1] | -1.33 |
| chr11_AA_01684 | hypothetical protein SERLADRAFT_479913 [Serpula lacrymans var. lacrymans S7.9] &gt;EGN94040.1 hypothetical protein SERLA73DRAFT_189201 [Serpula lacrymans var. lacrymans S7.3] &gt;EGO19389.1 hypothetical protein SERLADRAFT_479913 [Serpula lacrymans var. lacrymans S7.9] | -1.32 |
| chr04_AA_00285 | probable CDC31-spindle pole body component, centrin [Armillaria ostoyae] | -1.32 |
| chr04_AA_00335 | proliferating cell nuclear antigen [Armillaria gallica] | -1.32 |
| chr11_AA_00207 | NA | -1.31 |
| chr05_AA_00282 | NA | -1.31 |
| chr03_AA_00327 | glycosyltransferase family 3 protein [Cylindrobasidium torrendii FP15055 ss-10] | -1.30 |
| chr11_AA_01694 | urea transporter [Laccaria bicolor S238N-H82] &gt;EDR10274.1 urea transporter [Laccaria bicolor S238N-H82] | -1.30 |
| chr06_AA_00650 | arginyl-tRNA synthetase [Cylindrobasidium torrendii FP15055 ss-10] | -1.30 |
| chr04_AA_00336 | hypothetical protein CYLTODRAFT_409767 [Cylindrobasidium torrendii FP15055 ss-10] | -1.29 |
| chr03_AA_00177 | hypothetical protein ARMGADRAFT_1075117 [Armillaria gallica] | -1.29 |
| chr07_AA_00421 | kinase-like protein [Armillaria gallica] | -1.29 |
| chr11_AA_01608 | hypothetical protein ARMGADRAFT_1067232 [Armillaria gallica] | -1.28 |
| chr08_AA_00756 | hypothetical protein CVT24_006050 [Panaeolus cyanescens] | -1.28 |
| chr01_AA_00412 | P-loop containing nucleoside triphosphate hydrolase protein [Cylindrobasidium torrendii FP15055 ss-10] | -1.27 |
| chr05_AA_00606 | beta-glucan synthesis-associated protein [Armillaria gallica] | -1.27 |
| chr06_AA_00962 | hypothetical protein ARMGADRAFT_959585 [Armillaria gallica] &gt;SJL13756.1 probable Rpc11-DNA-directed RNA polymerase III subunit C11 [Armillaria ostoyae] | -1.26 |
| chr02_AA_00258 | FAD/NAD(P)-binding domain-containing protein [Armillaria solidipes] | -1.26 |
| chr11_AA_01461 | ricin B-like lectin [Armillaria gallica] | -1.25 |
| chr03_AA_00383 | hypothetical protein CYLTODRAFT_422417 [Cylindrobasidium torrendii FP15055 ss-10] | -1.24 |
| chr02_AA_00145 | predicted protein [Mycena chlorophos] | -1.24 |
| chr01_AA_00056 | hypothetical protein ARMSODRAFT_944675 [Armillaria solidipes] | -1.24 |
| chr01_AA_00057 | hypothetical protein ARMGADRAFT_1026134 [Armillaria gallica] | -1.24 |
| chr07_AA_00212 | hypothetical protein CYLTODRAFT_495068 [Cylindrobasidium torrendii FP15055 ss-10] | -1.24 |
| chr05_AA_00475 | uncharacterized protein ARMOST_10857 [Armillaria ostoyae] | -1.24 |
| chr06_AA_00644 | NA | -1.24 |
| chr06_AA_00370 | ribonuclease III [Cylindrobasidium torrendii FP15055 ss-10] | -1.23 |
| chr01_AA_00424 | hypothetical protein CYLTODRAFT_55692 [Cylindrobasidium torrendii FP15055 ss-10] | -1.23 |
| chr05_AA_00105 | hsp10-like protein [Dichomitus squalens LYAD-421 SS1] &gt;EJF55993.1 hsp10-like protein [Dichomitus squalens LYAD-421 SS1] | -1.22 |
| chr10_AA_00305 | kinesin-domain-containing protein [Cylindrobasidium torrendii FP15055 ss-10] | -1.21 |
| chr09_AA_01236 | hypothetical protein CYLTODRAFT_417978 [Cylindrobasidium torrendii FP15055 ss-10] | -1.21 |
| chr07_AA_01069 | WD repeat-containing protein slp1 [Cylindrobasidium torrendii FP15055 ss-10] | -1.20 |
| chr11_AA_01136 | hypothetical protein CYLTODRAFT_421620 [Cylindrobasidium torrendii FP15055 ss-10] | -1.20 |
| chr11_AA_00098 | hypothetical protein ARMGADRAFT_979593 [Armillaria gallica] | -1.19 |
| chr06_AA_00317 | hypothetical protein CYLTODRAFT_398740 [Cylindrobasidium torrendii FP15055 ss-10] | -1.19 |
| chr07_AA_00432 | hypothetical protein CYLTODRAFT_433656 [Cylindrobasidium torrendii FP15055 ss-10] | -1.19 |
| chr11_AA_00133 | hypothetical protein ARMGADRAFT_1162737 [Armillaria gallica] | -1.19 |
| chr10_AA_00368 | DUF726-domain-containing protein [Armillaria gallica] | -1.19 |
| chr07_AA_00434 | hypothetical protein ARMGADRAFT_1166177 [Armillaria gallica] | -1.18 |
| chr05_AA_00565 | uncharacterized protein ARMOST_06210 [Armillaria ostoyae] | -1.18 |
| chr04_AA_00436 | related to RPO41-DNA-directed RNA polymerase, mitochondrial [Armillaria ostoyae] | -1.18 |
| chr07_AA_00458 | hypothetical protein ARMSODRAFT_649359 [Armillaria solidipes] | -1.18 |
| chr10_AA_00321 | hypothetical protein CYLTODRAFT_418149 [Cylindrobasidium torrendii FP15055 ss-10] | -1.18 |
| chr04_AA_00494 | related to KRE6-glucan synthase subunit [Armillaria ostoyae] | -1.18 |
| chr01_AA_00245 | hypothetical protein CYLTODRAFT_343854, partial [Cylindrobasidium torrendii FP15055 ss-10] | -1.18 |
| chr06_AA_00655 | hypothetical protein GLOTRDRAFT_140973 [Gloeophyllum trabeum ATCC 11539] &gt;EPQ51580.1 hypothetical protein GLOTRDRAFT_140973 [Gloeophyllum trabeum ATCC 11539] | -1.17 |
| chr11_AA_01028 | hypothetical protein CYLTODRAFT_485083 [Cylindrobasidium torrendii FP15055 ss-10] | -1.17 |
| chr08_AA_00626 | hypothetical protein ARMGADRAFT_1006661 [Armillaria gallica] | -1.16 |
| chr07_AA_00226 | P-loop containing nucleoside triphosphate hydrolase protein [Mycena chlorophos] | -1.15 |
| chr10_AA_00735 | glycoside hydrolase family 31 protein [Cylindrobasidium torrendii FP15055 ss-10] | -1.15 |
| chr09_AA_00680 | hypothetical protein CYLTODRAFT_395198 [Cylindrobasidium torrendii FP15055 ss-10] | -1.14 |
| chr05_AA_00342 | hypothetical protein ARMGADRAFT_987831 [Armillaria gallica] | -1.14 |
| chr06_AA_00369 | hypothetical protein CYLTODRAFT_379951 [Cylindrobasidium torrendii FP15055 ss-10] | -1.12 |
| chr06_AA_00411 | Nitrate/Nitrite transporter [Heterobasidion irregulare TC 32-1] &gt;ETW81289.1 Nitrate/Nitrite transporter [Heterobasidion irregulare TC 32-1] | -1.11 |
| chr11_AA_01032 | FAD/NAD(P)-binding domain-containing protein [Cylindrobasidium torrendii FP15055 ss-10] | -1.10 |
| chr07_AA_00522 | hypothetical protein CYLTODRAFT_484594 [Cylindrobasidium torrendii FP15055 ss-10] | -1.09 |
| chr08_AA_00282 | beta-tubulin 2 [Cylindrobasidium torrendii FP15055 ss-10] | -1.08 |
| chr04_AA_00456 | hypothetical protein CYLTODRAFT_376171 [Cylindrobasidium torrendii FP15055 ss-10] | -1.05 |

The first two rows represent gene ID and gene annotation respectively. "NA" represents no annotation information. The third row represents the value of log2(fold change) derived from average value of 1099 differentially expressed genes shared in three treated groups compared to the control group.

Supplementary Table S5. Information of the 256 differentially expressed genes in MEblue module.

| Gene ID | Gene annotation | Log2(fold change) | Correlation value |
| --- | --- | --- | --- |
| chr09_AA_01190 | potassium/sodium eff [Cylindrobasidium torrendii FP15055 ss-10] | 6.84 | 151.16 |
| chr10_AA_00944 | general substrate transporter [Cylindrobasidium torrendii FP15055 ss-10] | 6.81 | 217.37 |
| chr10_AA_01322 | putative laccase 5 [Flammulina velutipes] | 6.65 | 126.40 |
| chr06_AA_00666 | hypothetical protein [Flammulina velutipes] | 6.46 | 128.12 |
| chr01_AA_00442 | hypothetical protein ARMGADRAFT_970088 [Armillaria gallica] | 6.23 | 235.84 |
| chr06_AA_00433 | putative ectomycorrhiza-upregulated exo-beta-1,3-glucanase GH5 [Flammulina velutipes] | 6.22 | 152.92 |
| chr09_AA_00051 | GMC oxidoreductase [Cylindrobasidium torrendii FP15055 ss-10] | 6.18 | 241.06 |
| chr01_AA_00428 | putative protein lysine methyltransferase [Moniliophthora roreri MCA 2997] | 6.02 | 176.38 |
| chr09_AA_00955 | putative endo-1,4-beta-xylanase precursor [Flammulina velutipes] | 6.02 | 128.52 |
| chr01_AA_00233 | related to D-arabinitol 2-dehydrogenase [Armillaria ostoyae] | 5.96 | 71.53 |
| chr11_AA_00581 | laccase [Flammulina velutipes] | 5.96 | 98.19 |
| chr02_AA_00108 | asparaginase [Flammulina velutipes] | 5.84 | 174.06 |
| chr01_AA_00232 | NAD(P)-binding protein [Armillaria gallica] | 5.57 | 101.40 |
| chr09_AA_00719 | hypothetical protein PLEOSDRAFT_1093927 [Pleurotus ostreatus PC15] | 5.40 | 146.96 |
| chr09_AA_00831 | hypothetical protein CYLTODRAFT_417837 [Cylindrobasidium torrendii FP15055 ss-10] | 5.36 | 50.56 |
| chr09_AA_00052 | GMC oxidoreductase [Cylindrobasidium torrendii FP15055 ss-10] | 5.30 | 165.36 |
| chr09_AA_00100 | hypothetical protein K503DRAFT_238266 [Rhizopogon vinicolor AM-OR11-026] | 5.29 | 232.55 |
| chr07_AA_00617 | NAD-dependent formate dehydrogenase [Moniliophthora perniciosa] | 5.22 | 172.54 |
| chr07_AA_00601 | hypothetical protein GYMLUDRAFT_71620 [Gymnopus luxurians FD-317 M1] | 5.13 | 200.95 |
| chr07_AA_00567 | hypothetical protein CYLTODRAFT_406012 [Cylindrobasidium torrendii FP15055 ss-10] | 5.12 | 164.37 |
| chr11_AA_01002 | related to dehydrogenase [Armillaria ostoyae] | 4.98 | 16.52 |
| chr11_AA_00499 | GroES-like protein [Armillaria solidipes] | 4.94 | 266.35 |
| chr06_AA_00694 | Zinc-regulated transporter 1 [Hypsizygus marmoreus] | 4.69 | 171.75 |
| chr11_AA_01685 | 1-aminocyclopropane-1-carboxylate deaminase [Armillaria solidipes] | 4.66 | 187.74 |
| chr11_AA_01046 | hypothetical protein CYLTODRAFT_428160 [Cylindrobasidium torrendii FP15055 ss-10] | 4.58 | 245.50 |
| chr09_AA_00190 | putative exo-beta-1,3-glucanase [Flammulina velutipes] | 4.50 | 113.83 |
| chr08_AA_00984 | cytochrome P450 monooxygenase pc-bph [Armillaria gallica] | 4.30 | 234.70 |
| chr11_AA_01097 | glycoside hydrolase family 3 protein [Cylindrobasidium torrendii FP15055 ss-10] | 4.27 | 209.86 |
| chr07_AA_00804 | uncharacterized protein ARMOST_19843 [Armillaria ostoyae] | 4.19 | 56.62 |
| chr03_AA_00325 | hypothetical protein CYLTODRAFT_421760 [Cylindrobasidium torrendii FP15055 ss-10] | 4.15 | 168.97 |
| chr09_AA_00892 | hypothetical protein CYLTODRAFT_431446 [Cylindrobasidium torrendii FP15055 ss-10] | 4.09 | 251.43 |
| chr11_AA_00595 | chitin deacetylase [Armillaria gallica] | 4.07 | 54.78 |
| chr09_AA_00191 | exo-beta-1,3-glucanase [Armillaria solidipes] | 4.06 | 112.52 |
| chr03_AA_00294 | hypothetical protein CYLTODRAFT_489528 [Cylindrobasidium torrendii FP15055 ss-10] | 4.04 | 200.00 |
| chr07_AA_00156 | uncharacterized protein ARMOST_00326 [Armillaria ostoyae] | 3.99 | 137.55 |
| chr09_AA_01099 | general substrate transporter [Armillaria gallica] | 3.96 | 209.49 |
| chr05_AA_00370 | hypothetical protein GALMADRAFT_90776 [Galerina marginata CBS 339.88] | 3.95 | 258.76 |
| chr04_AA_00484 | hypothetical protein CYLTODRAFT_422976 [Cylindrobasidium torrendii FP15055 ss-10] | 3.90 | 237.94 |
| chr11_AA_00094 | homogentisate 1,2-dioxygenase [Armillaria solidipes] | 3.87 | 230.55 |
| chr09_AA_01114 | glutathione S-transferase family, partial [Flammulina velutipes] | 3.80 | 21.40 |
| chr01_AA_00310 | hypothetical protein GYMLUDRAFT_150603 [Gymnopus luxurians FD-317 M1] | 3.79 | 222.54 |
| chr08_AA_01046 | MFS general substrate transporter [Armillaria gallica] | 3.79 | 238.87 |
| chr10_AA_01157 | glycoside hydrolase family 53 protein [Cylindrobasidium torrendii FP15055 ss-10] | 3.79 | 188.58 |
| chr06_AA_00366 | uncharacterized protein ARMOST_02422 [Armillaria ostoyae] | 3.70 | 137.12 |
| chr06_AA_00949 | hypothetical protein CYLTODRAFT_427642 [Cylindrobasidium torrendii FP15055 ss-10] | 3.64 | 164.78 |
| chr06_AA_01031 | glycoside hydrolase family 43 protein [Periconia macrospinosa] | 3.60 | 208.06 |
| chr09_AA_00964 | hypothetical protein CYLTODRAFT_421025 [Cylindrobasidium torrendii FP15055 ss-10] | 3.58 | 73.66 |
| chr11_AA_01456 | uncharacterized protein ARMOST_16541 [Armillaria ostoyae] | 3.58 | 236.36 |
| chr08_AA_00621 | hypothetical protein ARMGADRAFT_982650 [Armillaria gallica] | 3.54 | 207.16 |
| chr07_AA_00829 | general substrate transporter [Cylindrobasidium torrendii FP15055 ss-10] | 3.52 | 219.84 |
| chr10_AA_00733 | Zinc-regulated transporter 1 [Hypsizygus marmoreus] | 3.44 | 151.40 |
| chr04_AA_00045 | hypothetical protein ARMGADRAFT_1084273 [Armillaria gallica] | 3.41 | 109.51 |
| chr08_AA_00496 | amidase signature enzyme [Sanghuangporus baumii] | 3.41 | 122.06 |
| chr09_AA_00126 | glycoside hydrolase family 6 protein [Cylindrobasidium torrendii FP15055 ss-10] | 3.41 | 9.80 |
| chr05_AA_00224 | carotenoid ester lipase precursor [Cylindrobasidium torrendii FP15055 ss-10] | 3.38 | 80.48 |
| chr05_AA_00860 | hypothetical protein ARMGADRAFT_633196 [Armillaria gallica] | 3.31 | 228.71 |
| chr06_AA_00524 | hypothetical protein ARMGADRAFT_1111301 [Armillaria gallica] | 3.30 | 200.26 |
| chr02_AA_00057 | hypothetical protein ARMSODRAFT_931909 [Armillaria solidipes] &gt;SJL01654.1 uncharacterized protein ARMOST_04977 [Armillaria ostoyae] | 3.26 | 158.43 |
| chr11_AA_00233 | hypothetical protein ARMGADRAFT_977028 [Armillaria gallica] | 3.13 | 171.15 |
| chr09_AA_01224 | uncharacterized protein ARMOST_22339 [Armillaria ostoyae] | 3.12 | 164.55 |
| chr09_AA_00312 | putative beta-glucosidase [Flammulina velutipes] | 3.11 | 168.21 |
| chr05_AA_00845 | general substrate transporter [Armillaria gallica] | 3.05 | 223.09 |
| chr08_AA_01257 | hypothetical protein ARMSODRAFT_949486 [Armillaria solidipes] | 2.99 | 260.80 |
| chr04_AA_00685 | hypothetical protein CYLTODRAFT_369578 [Cylindrobasidium torrendii FP15055 ss-10] | 2.99 | 225.39 |
| chr08_AA_01077 | GroES-like protein [Cylindrobasidium torrendii FP15055 ss-10] | 2.97 | 57.14 |
| chr09_AA_00162 | hypothetical protein ARMGADRAFT_961910 [Armillaria gallica] | 2.96 | 257.73 |
| chr11_AA_00878 | hypothetical protein CYLTODRAFT_430286 [Cylindrobasidium torrendii FP15055 ss-10] | 2.91 | 260.60 |
| chr09_AA_00036 | hypothetical protein ARMGADRAFT_1070289 [Armillaria gallica] | 2.87 | 158.83 |
| chr11_AA_00928 | putative malate synthase [Flammulina velutipes] | 2.86 | 190.66 |
| chr09_AA_00271 | Endo-1,4-beta-xylanase 6 [Hypsizygus marmoreus] | 2.83 | 9.76 |
| chr05_AA_00599 | glycoside hydrolase family 2 protein [Cylindrobasidium torrendii FP15055 ss-10] | 2.80 | 194.94 |
| chr08_AA_00338 | DUF1479-domain-containing protein [Cylindrobasidium torrendii FP15055 ss-10] | 2.78 | 260.48 |
| chr04_AA_00291 | hypothetical protein ARMGADRAFT_1063603 [Armillaria gallica] | 2.77 | 92.23 |
| chr10_AA_00499 | hypothetical protein WG66_16220 [Moniliophthora roreri] | 2.75 | 211.53 |
| chr08_AA_00260 | CDF-like metal transporter [Armillaria solidipes] | 2.74 | 225.57 |
| chr09_AA_00023 | hypothetical protein WG66_107 [Moniliophthora roreri] | 2.73 | 77.01 |
| chr11_AA_00997 | Annexin [Cylindrobasidium torrendii FP15055 ss-10] | 2.71 | 208.07 |
| chr06_AA_00707 | glycerol kinase [Armillaria solidipes] | 2.69 | 203.06 |
| chr11_AA_00103 | hypothetical protein M413DRAFT_370146 [Hebeloma cylindrosporum h7] | 2.68 | 253.73 |
| chr11_AA_01731 | benzoquinone reductase [Cylindrobasidium torrendii FP15055 ss-10] | 2.68 | 98.19 |
| chr06_AA_00315 | mitochondrial carrier [Cylindrobasidium torrendii FP15055 ss-10] | 2.67 | 263.41 |
| chr01_AA_00035 | uncharacterized protein ARMOST_05935 [Armillaria ostoyae] | 2.63 | 160.25 |
| chr10_AA_01414 | hypothetical protein ARMSODRAFT_995949 [Armillaria solidipes] | 2.62 | 37.39 |
| chr04_AA_00470 | hypothetical protein PLICRDRAFT_179967 [Plicaturopsis crispa FD-325 SS-3] | 2.61 | 123.90 |
| chr05_AA_00686 | glycoside hydrolase family 95 protein [Cylindrobasidium torrendii FP15055 ss-10] | 2.61 | 205.99 |
| chr10_AA_01309 | hypothetical protein PHLCEN_2v2282 [Phlebia centrifuga] | 2.60 | 84.48 |
| chr09_AA_00551 | hypothetical protein CYLTODRAFT_392634 [Cylindrobasidium torrendii FP15055 ss-10] | 2.59 | 117.28 |
| chr07_AA_00703 | potassium/sodium eff [Cylindrobasidium torrendii FP15055 ss-10] | 2.58 | 270.93 |
| chr05_AA_00118 | glycoside hydrolase family 37 protein [Cylindrobasidium torrendii FP15055 ss-10] | 2.57 | 231.93 |
| chr03_AA_00403 | hypothetical protein CYLTODRAFT_424860 [Cylindrobasidium torrendii FP15055 ss-10] | 2.56 | 245.23 |
| chr05_AA_00317 | hypothetical protein CYLTODRAFT_450559 [Cylindrobasidium torrendii FP15055 ss-10] | 2.54 | 212.36 |
| chr08_AA_01091 | uncharacterized protein ARMOST_03386 [Armillaria ostoyae] | 2.50 | 257.47 |
| chr08_AA_01081 | uncharacterized protein ARMOST_20173 [Armillaria ostoyae] | 2.49 | 218.70 |
| chr01_AA_00436 | hypothetical protein ARMSODRAFT_960465 [Armillaria solidipes] | 2.49 | 229.10 |
| chr10_AA_00270 | branched-chain alpha-keto acid dehydrogenase E1-alpha subunit [Cylindrobasidium torrendii FP15055 ss-10] | 2.47 | 254.26 |
| chr02_AA_00121 | vacuolar amino acid permease [Armillaria gallica] | 2.46 | 130.99 |
| chr06_AA_00703 | hypothetical protein CYLTODRAFT_398145 [Cylindrobasidium torrendii FP15055 ss-10] | 2.43 | 257.46 |
| chr09_AA_00095 | hypothetical protein CYLTODRAFT_422329 [Cylindrobasidium torrendii FP15055 ss-10] | 2.43 | 250.61 |
| chr10_AA_01231 | hypothetical protein ARMSODRAFT_1019532 [Armillaria solidipes] | 2.41 | 219.92 |
| chr08_AA_00858 | cytochrome P450 [Cylindrobasidium torrendii FP15055 ss-10] | 2.40 | 287.71 |
| chr10_AA_01201 | acyltransferase ChoActase/COT/CPT [Armillaria gallica] | 2.39 | 232.43 |
| chr10_AA_00662 | Ureohydrolase [Armillaria gallica] | 2.36 | 188.94 |
| chr11_AA_01686 | hypothetical protein CYLTODRAFT_348076 [Cylindrobasidium torrendii FP15055 ss-10] | 2.35 | 156.03 |
| chr11_AA_00606 | hypothetical protein CYLTODRAFT_418833 [Cylindrobasidium torrendii FP15055 ss-10] | 2.34 | 246.72 |
| chr07_AA_01051 | MFS general substrate transporter [Cylindrobasidium torrendii FP15055 ss-10] | 2.34 | 208.78 |
| chr10_AA_00134 | hypothetical protein CYLTODRAFT_349864 [Cylindrobasidium torrendii FP15055 ss-10] | 2.33 | 231.34 |
| chr06_AA_00970 | glycoside hydrolase family 5 protein [Pleurotus ostreatus PC15] | 2.30 | 76.81 |
| chr05_AA_00082 | glycoside hydrolase family 20 protein [Cylindrobasidium torrendii FP15055 ss-10] | 2.30 | 186.30 |
| chr11_AA_00790 | uncharacterized protein ARMOST_05401 [Armillaria ostoyae] | 2.29 | 175.83 |
| chr09_AA_00762 | transketolase [Armillaria solidipes] | 2.29 | 189.20 |
| chr09_AA_01214 | hypothetical protein CYLTODRAFT_417942 [Cylindrobasidium torrendii FP15055 ss-10] | 2.29 | 214.53 |
| chr02_AA_00393 | related to GRE2-methylglyoxal reductase (NADPH-dependent) [Armillaria ostoyae] | 2.28 | 177.14 |
| chr07_AA_00414 | MFS general substrate transporter [Armillaria gallica] | 2.26 | 188.29 |
| chr07_AA_01020 | extracellular GDSL-like lipase/acylhydrolase [Glonium stellatum] | 2.26 | 201.88 |
| chr05_AA_00056 | glycoside hydrolase family 20 protein [Cylindrobasidium torrendii FP15055 ss-10] | 2.24 | 173.83 |
| chr05_AA_00715 | NAD(P)-binding protein [Cylindrobasidium torrendii FP15055 ss-10] | 2.24 | 75.35 |
| chr07_AA_00464 | cytochrome P450 [Cylindrobasidium torrendii FP15055 ss-10] | 2.23 | 225.58 |
| chr03_AA_00291 | hypothetical protein ARMGADRAFT_1170747 [Armillaria gallica] | 2.22 | 202.36 |
| chr10_AA_00667 | hypothetical protein CYLTODRAFT_379376 [Cylindrobasidium torrendii FP15055 ss-10] | 2.22 | 34.90 |
| chr10_AA_01310 | DUF124-domain-containing protein [Cylindrobasidium torrendii FP15055 ss-10] | 2.20 | 65.46 |
| chr01_AA_00418 | aromatic compound dioxygenase [Cylindrobasidium torrendii FP15055 ss-10] | 2.16 | 230.99 |
| chr03_AA_00380 | NAD(P)-binding protein [Cylindrobasidium torrendii FP15055 ss-10] | 2.16 | 88.52 |
| chr10_AA_01056 | hypothetical protein ARMGADRAFT_1020988 [Armillaria gallica] | 2.16 | 159.66 |
| chr09_AA_00406 | uncharacterized protein ARMOST_17180 [Armillaria ostoyae] | 2.14 | 215.81 |
| chr09_AA_01225 | NAD(P)-binding protein [Cylindrobasidium torrendii FP15055 ss-10] | 2.14 | 85.03 |
| chr09_AA_00816 | mandelate racemase muconate lactonizing enzyme family protein [Cylindrobasidium torrendii FP15055 ss-10] | 2.12 | 152.90 |
| chr08_AA_00542 | hypothetical protein ARMGADRAFT_1159894 [Armillaria gallica] | 2.11 | 182.73 |
| chr07_AA_01021 | hypothetical protein PLEOSDRAFT_1096996 [Pleurotus ostreatus PC15] | 2.10 | 102.60 |
| chr10_AA_00220 | hypothetical protein ARMGADRAFT_962646, partial [Armillaria gallica] | 2.08 | 280.35 |
| chr10_AA_01143 | uncharacterized protein ARMOST_08409 [Armillaria ostoyae] | 2.08 | 159.29 |
| chr10_AA_00912 | hypothetical protein ARMSODRAFT_948409 [Armillaria solidipes] | 2.08 | 71.18 |
| chr11_AA_00719 | hypothetical protein ARMGADRAFT_1019784, partial [Armillaria gallica] | 2.04 | 177.29 |
| chr11_AA_01033 | glycoside hydrolase family 51 protein [Cylindrobasidium torrendii FP15055 ss-10] | 2.04 | 290.52 |
| chr02_AA_00341 | oxidoreductase [Armillaria solidipes] | 2.02 | 259.11 |
| chr05_AA_00812 | acid protease [Armillaria gallica] | 1.99 | 214.06 |
| chr09_AA_01106 | multidrug transporter [Cylindrobasidium torrendii FP15055 ss-10] | 1.95 | 124.59 |
| chr01_AA_00397 | putative isomerase YbhE [Cylindrobasidium torrendii FP15055 ss-10] | 1.94 | 188.18 |
| chr09_AA_00838 | cytochrome P450 [Armillaria gallica] | 1.94 | 101.42 |
| chr08_AA_00847 | acetyl-CoA synthetase-like protein [Armillaria solidipes] | 1.94 | 84.22 |
| chr07_AA_00098 | hypothetical protein ARMGADRAFT_926019 [Armillaria gallica] | 1.90 | 52.63 |
| chr07_AA_00419 | hypothetical protein CYLTODRAFT_277105 [Cylindrobasidium torrendii FP15055 ss-10] | 1.90 | 162.23 |
| chr10_AA_00441 | alpha/beta-hydrolase [Armillaria solidipes] | 1.90 | 263.94 |
| chr07_AA_00645 | aldehyde dehydrogenase [Armillaria gallica] | 1.89 | 242.55 |
| chr02_AA_00171 | hypothetical protein CYLTODRAFT_425697 [Cylindrobasidium torrendii FP15055 ss-10] | 1.87 | 284.74 |
| chr07_AA_00561 | hypothetical protein ARMGADRAFT_1031622 [Armillaria gallica] | 1.86 | 210.38 |
| chr07_AA_00739 | hypothetical protein CYLTODRAFT_417357 [Cylindrobasidium torrendii FP15055 ss-10] | 1.85 | 255.08 |
| chr08_AA_01176 | hypothetical protein HYPSUDRAFT_61393 [Hypholoma sublateritium FD-334 SS-4] | 1.85 | 237.18 |
| chr11_AA_01644 | hypothetical protein CYLTODRAFT_438918 [Cylindrobasidium torrendii FP15055 ss-10] | 1.84 | 151.70 |
| chr08_AA_00948 | probable glutamate dehydrogenase, NAD(+)-specific [Armillaria ostoyae] | 1.84 | 196.82 |
| chr09_AA_00860 | glycosyltransferase family 90 protein [Cylindrobasidium torrendii FP15055 ss-10] | 1.83 | 70.85 |
| chr09_AA_01103 | Metallo-dependent phosphatase [Armillaria solidipes] | 1.83 | 253.14 |
| chr10_AA_01063 | glycoside hydrolase family 43 protein [Amanita thiersii Skay4041] | 1.83 | 150.42 |
| chr08_AA_00651 | related to isp4-oligopeptide transporter [Armillaria ostoyae] | 1.83 | 165.94 |
| chr10_AA_00256 | uncharacterized protein ARMOST_03672 [Armillaria ostoyae] | 1.82 | 217.07 |
| chr06_AA_00683 | amidohydrolase [Armillaria gallica] | 1.81 | 113.51 |
| chr11_AA_00265 | FAD-linked oxidoreductase [Armillaria solidipes] | 1.80 | 247.36 |
| chr11_AA_01106 | related to stomatin [Armillaria ostoyae] | 1.79 | 193.35 |
| chr10_AA_01664 | hypothetical protein CYLTODRAFT_404266 [Cylindrobasidium torrendii FP15055 ss-10] | 1.77 | 183.06 |
| chr05_AA_00719 | lipolytic enzyme [Coprinopsis cinerea okayama7#130] &gt;EAU92167.1 lipolytic enzyme [Coprinopsis cinerea okayama7#130] | 1.77 | 10.21 |
| chr11_AA_01711 | hypothetical protein ARMGADRAFT_1046867 [Armillaria gallica] | 1.76 | 281.10 |
| chr07_AA_00560 | hypothetical protein CYLTODRAFT_416229 [Cylindrobasidium torrendii FP15055 ss-10] | 1.76 | 256.15 |
| chr11_AA_00701 | uncharacterized protein ARMOST_05344 [Armillaria ostoyae] | 1.76 | 137.17 |
| chr08_AA_00018 | putative peroxiredoxin Q [Flammulina velutipes] | 1.75 | 253.69 |
| chr04_AA_00052 | hypothetical protein CYLTODRAFT_421165 [Cylindrobasidium torrendii FP15055 ss-10] | 1.75 | 125.29 |
| chr08_AA_01259 | Aldo/keto reductase [Armillaria gallica] | 1.74 | 276.12 |
| chr07_AA_00826 | Metallo-hydrolase/oxidoreductase [Armillaria gallica] | 1.74 | 187.75 |
| chr11_AA_01324 | terpenoid synthase [Cylindrobasidium torrendii FP15055 ss-10] | 1.73 | 235.42 |
| chr11_AA_01636 | hypothetical protein CYLTODRAFT_421880 [Cylindrobasidium torrendii FP15055 ss-10] | 1.72 | 255.32 |
| chr02_AA_00129 | hypothetical protein CYLTODRAFT_366704 [Cylindrobasidium torrendii FP15055 ss-10] | 1.71 | 207.68 |
| chr08_AA_00106 | hypothetical protein ARMGADRAFT_100162 [Armillaria gallica] | 1.70 | 162.90 |
| chr10_AA_01576 | D-arabinitol 2-dehydrogenase [ribulose-forming] [Hypsizygus marmoreus] | 1.69 | 208.25 |
| chr01_AA_00121 | hypothetical protein CYLTODRAFT_28398 [Cylindrobasidium torrendii FP15055 ss-10] | 1.69 | 108.32 |
| chr07_AA_01006 | enolase C-terminal domain-like protein [Cylindrobasidium torrendii FP15055 ss-10] | 1.67 | 102.89 |
| chr10_AA_01213 | hypothetical protein JAAARDRAFT_210932 [Jaapia argillacea MUCL 33604] | 1.66 | 249.95 |
| chr06_AA_00426 | hypothetical protein ARMGADRAFT_984775 [Armillaria gallica] | 1.65 | 80.98 |
| chr01_AA_00479 | uncharacterized protein ARMOST_12330 [Armillaria ostoyae] | 1.65 | 256.41 |
| chr07_AA_00907 | uncharacterized protein ARMOST_00985 [Armillaria ostoyae] | 1.65 | 228.07 |
| chr08_AA_00856 | cytochrome p450 [Moniliophthora roreri MCA 2997] | 1.65 | 220.84 |
| chr07_AA_00281 | NAD(P)-binding protein [Armillaria gallica] | 1.64 | 267.60 |
| chr09_AA_01324 | uncharacterized protein ARMOST_03294 [Armillaria ostoyae] | 1.64 | 75.75 |
| chr10_AA_00164 | dehydrogenase E1 and transketolase domain-containing protein 1 [Cylindrobasidium torrendii FP15055 ss-10] | 1.63 | 256.29 |
| chr09_AA_00410 | hypothetical protein AMATHDRAFT_73341 [Amanita thiersii Skay4041] | 1.62 | 111.82 |
| chr11_AA_01733 | glycoside hydrolase family 76 protein [Cylindrobasidium torrendii FP15055 ss-10] | 1.62 | 263.67 |
| chr11_AA_00027 | hypothetical protein CYLTODRAFT_423477 [Cylindrobasidium torrendii FP15055 ss-10] | 1.61 | 100.27 |
| chr08_AA_01097 | hypothetical protein GYMLUDRAFT_32937 [Gymnopus luxurians FD-317 M1] | 1.59 | 122.19 |
| chr11_AA_01732 | glycoside hydrolase family 76 protein [Cylindrobasidium torrendii FP15055 ss-10] | 1.57 | 256.62 |
| chr05_AA_00112 | hypothetical protein STEHIDRAFT_155150 [Stereum hirsutum FP-91666 SS1] &gt;EIM87778.1 hypothetical protein STEHIDRAFT_155150 [Stereum hirsutum FP-91666 SS1] | 1.57 | 184.71 |
| chr11_AA_00724 | WD40 repeat-like protein [Armillaria solidipes] | 1.56 | 287.32 |
| chr05_AA_00607 | hypothetical protein CYLTODRAFT_458172 [Cylindrobasidium torrendii FP15055 ss-10] | 1.56 | 148.71 |
| chr11_AA_00145 | hypothetical protein CYLTODRAFT_243491 [Cylindrobasidium torrendii FP15055 ss-10] | 1.55 | 165.76 |
| chr08_AA_00287 | hypothetical protein PLICRDRAFT_118622 [Plicaturopsis crispa FD-325 SS-3] | 1.55 | 209.77 |
| chr06_AA_00308 | hypothetical protein CYLTODRAFT_397357 [Cylindrobasidium torrendii FP15055 ss-10] | 1.55 | 124.99 |
| chr08_AA_00501 | hypothetical protein CYLTODRAFT_416851 [Cylindrobasidium torrendii FP15055 ss-10] | 1.55 | 244.15 |
| chr11_AA_00504 | MFS general substrate transporter [Cylindrobasidium torrendii FP15055 ss-10] | 1.52 | 266.66 |
| chr06_AA_00237 | acyl-CoA dehydrogenase NM domain-like protein, partial [Armillaria solidipes] | 1.52 | 266.39 |
| chr09_AA_00139 | hypothetical protein CYLTODRAFT_424555 [Cylindrobasidium torrendii FP15055 ss-10] | 1.51 | 142.32 |
| chr09_AA_01058 | hypothetical protein ARMSODRAFT_976614 [Armillaria solidipes] | 1.51 | 243.69 |
| chr05_AA_00156 | hypothetical protein Moror_2973 [Moniliophthora roreri MCA 2997] &gt;KTB44036.1 hypothetical protein WG66_3386 [Moniliophthora roreri] | 1.51 | 101.60 |
| chr11_AA_01313 | hypothetical protein Hypma_000372 [Hypsizygus marmoreus] | 1.50 | 235.00 |
| chr05_AA_00481 | hypothetical protein CYLTODRAFT_424727 [Cylindrobasidium torrendii FP15055 ss-10] | 1.49 | 261.35 |
| chr10_AA_01390 | hypothetical protein CYLTODRAFT_414265 [Cylindrobasidium torrendii FP15055 ss-10] | 1.48 | 77.23 |
| chr07_AA_01003 | Pkinase-domain-containing protein [Armillaria gallica] | 1.47 | 127.50 |
| chr05_AA_00473 | hypothetical protein CYLTODRAFT_449880 [Cylindrobasidium torrendii FP15055 ss-10] | 1.45 | 196.48 |
| chr07_AA_01044 | uncharacterized protein ARMOST_19175 [Armillaria ostoyae] | 1.43 | 190.04 |
| chr08_AA_00383 | NAD(P)-binding protein [Armillaria gallica] | 1.42 | 100.13 |
| chr07_AA_01008 | cytochrome P450 [Armillaria solidipes] | 1.41 | 60.64 |
| chr03_AA_00240 | hypothetical protein CYLTODRAFT_426591 [Cylindrobasidium torrendii FP15055 ss-10] | 1.40 | 191.58 |
| chr02_AA_00284 | alcohol dehydrogenase [Armillaria solidipes] | 1.40 | 103.93 |
| chr08_AA_00936 | MFS general substrate transporter [Armillaria gallica] | 1.39 | 236.36 |
| chr09_AA_01141 | hypothetical protein ARMGADRAFT_1063089 [Armillaria gallica] | 1.37 | 260.77 |
| chr08_AA_00687 | Clavaminate synthase-like protein [Armillaria solidipes] | 1.37 | 281.87 |
| chr08_AA_01172 | carbohydrate esterase family 1 protein, partial [Cylindrobasidium torrendii FP15055 ss-10] | 1.37 | 163.25 |
| chr10_AA_01341 | glucuronyl hydrolase [Armillaria solidipes] | 1.35 | 186.60 |
| chr11_AA_00638 | hypothetical protein ARMSODRAFT_953107 [Armillaria solidipes] | 1.33 | 240.76 |
| chr09_AA_01410 | hypothetical protein CYLTODRAFT_489277 [Cylindrobasidium torrendii FP15055 ss-10] | 1.33 | 92.92 |
| chr11_AA_01445 | hypothetical protein ARMSODRAFT_950555 [Armillaria solidipes] &gt;SJL10115.1 uncharacterized protein ARMOST_13499 [Armillaria ostoyae] | 1.32 | 89.74 |
| chr11_AA_00042 | kinase-like protein [Cylindrobasidium torrendii FP15055 ss-10] | 1.32 | 274.94 |
| chr09_AA_00229 | succinyl-CoA:3-ketoacid-coenzyme A transferase [Cylindrobasidium torrendii FP15055 ss-10] | 1.31 | 203.67 |
| chr10_AA_00517 | hypothetical protein CYLTODRAFT_256211 [Cylindrobasidium torrendii FP15055 ss-10] | 1.30 | 256.01 |
| chr07_AA_00951 | acyl-CoA dehydrogenase domain-containing protein [Cylindrobasidium torrendii FP15055 ss-10] | 1.30 | 208.38 |
| chr01_AA_00265 | hypothetical protein CYLTODRAFT_383163 [Cylindrobasidium torrendii FP15055 ss-10] | 1.30 | 243.02 |
| chr07_AA_00159 | FAD-binding domain-containing protein [Armillaria solidipes] | 1.30 | 115.01 |
| chr08_AA_01155 | related to 3-oxoacyl CoA thiolase [Armillaria ostoyae] | 1.30 | 242.87 |
| chr11_AA_00277 | uncharacterized protein ARMOST_10602 [Armillaria ostoyae] | 1.29 | 183.29 |
| chr10_AA_00651 | hypothetical protein ARMGADRAFT_897215, partial [Armillaria gallica] | 1.29 | 189.96 |
| chr01_AA_00277 | OPT superfamily oligopeptide transporter [Cylindrobasidium torrendii FP15055 ss-10] | 1.29 | 214.03 |
| chr11_AA_01664 | hypothetical protein CYLTODRAFT_421876 [Cylindrobasidium torrendii FP15055 ss-10] | 1.28 | 227.11 |
| chr06_AA_00738 | pyridoxal phosphate-dependent enzyme, beta subunit [Cylindrobasidium torrendii FP15055 ss-10] | 1.28 | 280.84 |
| chr07_AA_01089 | cysteine proteinase [Cylindrobasidium torrendii FP15055 ss-10] | 1.26 | 274.79 |
| chr11_AA_01042 | acetoin reductase family protein [Cylindrobasidium torrendii FP15055 ss-10] | 1.25 | 125.88 |
| chr01_AA_00301 | manganese and iron superoxide dismutase [Cylindrobasidium torrendii FP15055 ss-10] | 1.25 | 64.36 |
| chr09_AA_01392 | aldolase [Armillaria gallica] | 1.21 | 57.94 |
| chr08_AA_01027 | hypothetical protein CYLTODRAFT_357890 [Cylindrobasidium torrendii FP15055 ss-10] | 1.20 | 291.42 |
| chr06_AA_00511 | hypothetical protein CYLTODRAFT_487823 [Cylindrobasidium torrendii FP15055 ss-10] | 1.20 | 210.02 |
| chr03_AA_00298 | hypothetical protein CYLTODRAFT_458738 [Cylindrobasidium torrendii FP15055 ss-10] | 1.17 | 234.25 |
| chr06_AA_00682 | hypothetical protein HYDPIDRAFT_112868 [Hydnomerulius pinastri MD-312] | 1.16 | 183.70 |
| chr07_AA_00722 | hypothetical protein K443DRAFT_93998 [Laccaria amethystina LaAM-08-1] | 1.15 | 224.70 |
| chr07_AA_00827 | hypothetical protein LENED_003150 [Lentinula edodes] | 1.14 | 156.50 |
| chr07_AA_00756 | SAICAR synthase-like protein [Cylindrobasidium torrendii FP15055 ss-10] | 1.13 | 168.36 |
| chr07_AA_00675 | multidrug resistance protein 1 [Armillaria gallica] | 1.13 | 110.27 |
| chr11_AA_01510 | hypothetical protein ARMGADRAFT_1015707 [Armillaria gallica] | 1.11 | 146.20 |
| chr08_AA_01069 | alpha/beta-hydrolase [Armillaria solidipes] | 1.09 | 141.21 |
| chr05_AA_00421 | ribosomal protein S5 domain 2-like protein [Cylindrobasidium torrendii FP15055 ss-10] | 1.08 | 166.11 |
| chr10_AA_00693 | hypothetical protein ARMSODRAFT_947720 [Armillaria solidipes] | 1.08 | 288.78 |
| chr01_AA_00426 | hypothetical protein CYLTODRAFT_371735 [Cylindrobasidium torrendii FP15055 ss-10] | 1.07 | 204.46 |
| chr09_AA_00241 | SNF2 chromatin remodeling protein [Armillaria solidipes] | 1.07 | 288.25 |
| chr09_AA_00518 | AAA-domain-containing protein [Cylindrobasidium torrendii FP15055 ss-10] | 1.07 | 260.72 |
| chr07_AA_00380 | ectomycorrhiza-regulated esterase [Armillaria solidipes] | 1.04 | 216.13 |
| chr08_AA_00413 | hypothetical protein ARMGADRAFT_391312 [Armillaria gallica] | 1.01 | 205.20 |
| chr04_AA_00456 | hypothetical protein CYLTODRAFT_376171 [Cylindrobasidium torrendii FP15055 ss-10] | -1.01 | 249.05 |
| chr10_AA_00735 | glycoside hydrolase family 31 protein [Cylindrobasidium torrendii FP15055 ss-10] | -1.04 | 211.20 |
| chr11_AA_01461 | ricin B-like lectin [Armillaria gallica] | -1.09 | 218.89 |
| chr09_AA_00680 | hypothetical protein CYLTODRAFT_395198 [Cylindrobasidium torrendii FP15055 ss-10] | -1.11 | 260.26 |
| chr01_AA_00056 | hypothetical protein ARMSODRAFT_944675 [Armillaria solidipes] | -1.16 | 208.56 |
| chr01_AA_00057 | hypothetical protein ARMGADRAFT_1026134 [Armillaria gallica] | -1.19 | 235.44 |
| chr05_AA_00527 | hypothetical protein NEOLEDRAFT_1026610, partial [Neolentinus lepideus HHB14362 ss-1] | -1.54 | 202.30 |

The first two rows represent gene ID and gene annotation respectively. "NA" represents no annotation information. The third row represents the value of log2(fold change) derived from average value of 1,099 differentially expressed genes shared in three treated groups compared to the control group. The fourth row represents the connectivity value, standing for connectivity of every hub gene in the MEblue module.

Supplementary Table S6. Information of 859 edges in the co-expression network.

| Gene1 | Gene1_annotation | Gene2 | Gene2_annotation | Weight |
| --- | --- | --- | --- | --- |
| chr08_AA_01027 | hypothetical protein CYLTODRAFT_357890 [Cylindrobasidium torrendii FP15055 ss-10] | chr08_AA_01046 | MFS general substrate transporter [Armillaria gallica] | 0.217404189 |
| chr08_AA_01027 | hypothetical protein CYLTODRAFT_357890 [Cylindrobasidium torrendii FP15055 ss-10] | chr08_AA_01069 | alpha/beta-hydrolase [Armillaria solidipes] | 0.120331821 |
| chr08_AA_01027 | hypothetical protein CYLTODRAFT_357890 [Cylindrobasidium torrendii FP15055 ss-10] | chr08_AA_01077 | GroES-like protein [Cylindrobasidium torrendii FP15055 ss-10] | 0.109853792 |
| chr08_AA_01027 | hypothetical protein CYLTODRAFT_357890 [Cylindrobasidium torrendii FP15055 ss-10] | chr08_AA_01081 | uncharacterized protein ARMOST_20173 [Armillaria ostoyae] | 0.251015162 |
| chr08_AA_01027 | hypothetical protein CYLTODRAFT_357890 [Cylindrobasidium torrendii FP15055 ss-10] | chr08_AA_01091 | uncharacterized protein ARMOST_03386 [Armillaria ostoyae] | 0.250393489 |
| chr08_AA_01027 | hypothetical protein CYLTODRAFT_357890 [Cylindrobasidium torrendii FP15055 ss-10] | chr08_AA_01097 | hypothetical protein GYMLUDRAFT_32937 [Gymnopus luxurians FD-317 M1] | 0.186448141 |
| chr08_AA_01027 | hypothetical protein CYLTODRAFT_357890 [Cylindrobasidium torrendii FP15055 ss-10] | chr08_AA_01155 | related to 3-oxoacyl CoA thiolase [Armillaria ostoyae] | 0.163862111 |
| chr08_AA_01027 | hypothetical protein CYLTODRAFT_357890 [Cylindrobasidium torrendii FP15055 ss-10] | chr08_AA_01172 | carbohydrate esterase family 1 protein, partial [Cylindrobasidium torrendii FP15055 ss-10] | 0.147348614 |
| chr08_AA_01027 | hypothetical protein CYLTODRAFT_357890 [Cylindrobasidium torrendii FP15055 ss-10] | chr08_AA_01176 | hypothetical protein HYPSUDRAFT_61393 [Hypholoma sublateritium FD-334 SS-4] | 0.242149859 |
| chr08_AA_01027 | hypothetical protein CYLTODRAFT_357890 [Cylindrobasidium torrendii FP15055 ss-10] | chr08_AA_01257 | hypothetical protein ARMSODRAFT_949486 [Armillaria solidipes] | 0.294244609 |
| chr08_AA_01027 | hypothetical protein CYLTODRAFT_357890 [Cylindrobasidium torrendii FP15055 ss-10] | chr08_AA_01259 | Aldo/keto reductase [Armillaria gallica] | 0.253022423 |
| chr08_AA_01027 | hypothetical protein CYLTODRAFT_357890 [Cylindrobasidium torrendii FP15055 ss-10] | chr09_AA_00036 | hypothetical protein ARMGADRAFT_1070289 [Armillaria gallica] | 0.172049402 |
| chr08_AA_01027 | hypothetical protein CYLTODRAFT_357890 [Cylindrobasidium torrendii FP15055 ss-10] | chr09_AA_00051 | GMC oxidoreductase [Cylindrobasidium torrendii FP15055 ss-10] | 0.272866089 |
| chr08_AA_01027 | hypothetical protein CYLTODRAFT_357890 [Cylindrobasidium torrendii FP15055 ss-10] | chr09_AA_00052 | GMC oxidoreductase [Cylindrobasidium torrendii FP15055 ss-10] | 0.252087774 |
| chr08_AA_01027 | hypothetical protein CYLTODRAFT_357890 [Cylindrobasidium torrendii FP15055 ss-10] | chr09_AA_00095 | hypothetical protein CYLTODRAFT_422329 [Cylindrobasidium torrendii FP15055 ss-10] | 0.230140623 |
| chr08_AA_01027 | hypothetical protein CYLTODRAFT_357890 [Cylindrobasidium torrendii FP15055 ss-10] | chr09_AA_00100 | hypothetical protein K503DRAFT_238266 [Rhizopogon vinicolor AM-OR11-026] | 0.22067171 |
| chr08_AA_01027 | hypothetical protein CYLTODRAFT_357890 [Cylindrobasidium torrendii FP15055 ss-10] | chr09_AA_00139 | hypothetical protein CYLTODRAFT_424555 [Cylindrobasidium torrendii FP15055 ss-10] | 0.1105647 |
| chr08_AA_01027 | hypothetical protein CYLTODRAFT_357890 [Cylindrobasidium torrendii FP15055 ss-10] | chr09_AA_00162 | hypothetical protein ARMGADRAFT_961910 [Armillaria gallica] | 0.228295167 |
| chr08_AA_01027 | hypothetical protein CYLTODRAFT_357890 [Cylindrobasidium torrendii FP15055 ss-10] | chr09_AA_00190 | putative exo-beta-1,3-glucanase [Flammulina velutipes] | 0.152528369 |
| chr08_AA_01027 | hypothetical protein CYLTODRAFT_357890 [Cylindrobasidium torrendii FP15055 ss-10] | chr09_AA_00191 | exo-beta-1,3-glucanase [Armillaria solidipes] | 0.14999023 |
| chr08_AA_01027 | hypothetical protein CYLTODRAFT_357890 [Cylindrobasidium torrendii FP15055 ss-10] | chr09_AA_00229 | succinyl-CoA:3-ketoacid-coenzyme A transferase [Cylindrobasidium torrendii FP15055 ss-10] | 0.178779278 |
| chr08_AA_01027 | hypothetical protein CYLTODRAFT_357890 [Cylindrobasidium torrendii FP15055 ss-10] | chr09_AA_00241 | SNF2 chromatin remodeling protein [Armillaria solidipes] | 0.278613472 |
| chr08_AA_01027 | hypothetical protein CYLTODRAFT_357890 [Cylindrobasidium torrendii FP15055 ss-10] | chr09_AA_00312 | putative beta-glucosidase [Flammulina velutipes] | 0.212011925 |
| chr08_AA_01027 | hypothetical protein CYLTODRAFT_357890 [Cylindrobasidium torrendii FP15055 ss-10] | chr09_AA_00406 | uncharacterized protein ARMOST_17180 [Armillaria ostoyae] | 0.27391632 |
| chr08_AA_01027 | hypothetical protein CYLTODRAFT_357890 [Cylindrobasidium torrendii FP15055 ss-10] | chr09_AA_00518 | AAA-domain-containing protein [Cylindrobasidium torrendii FP15055 ss-10] | 0.276953543 |
| chr08_AA_01027 | hypothetical protein CYLTODRAFT_357890 [Cylindrobasidium torrendii FP15055 ss-10] | chr09_AA_00551 | hypothetical protein CYLTODRAFT_392634 [Cylindrobasidium torrendii FP15055 ss-10] | 0.119780936 |
| chr08_AA_01027 | hypothetical protein CYLTODRAFT_357890 [Cylindrobasidium torrendii FP15055 ss-10] | chr09_AA_00680 | hypothetical protein CYLTODRAFT_395198 [Cylindrobasidium torrendii FP15055 ss-10] | 0.24007311 |
| chr08_AA_01027 | hypothetical protein CYLTODRAFT_357890 [Cylindrobasidium torrendii FP15055 ss-10] | chr09_AA_00719 | hypothetical protein PLEOSDRAFT_1093927 [Pleurotus ostreatus PC15] | 0.24081154 |
| chr08_AA_01027 | hypothetical protein CYLTODRAFT_357890 [Cylindrobasidium torrendii FP15055 ss-10] | chr09_AA_00762 | transketolase [Armillaria solidipes] | 0.103835471 |
| chr08_AA_01027 | hypothetical protein CYLTODRAFT_357890 [Cylindrobasidium torrendii FP15055 ss-10] | chr09_AA_00816 | mandelate racemase muconate lactonizing enzyme family protein [Cylindrobasidium torrendii FP15055 ss-10] | 0.122735574 |
| chr08_AA_01027 | hypothetical protein CYLTODRAFT_357890 [Cylindrobasidium torrendii FP15055 ss-10] | chr09_AA_00838 | cytochrome P450 [Armillaria gallica] | 0.189567818 |
| chr08_AA_01027 | hypothetical protein CYLTODRAFT_357890 [Cylindrobasidium torrendii FP15055 ss-10] | chr09_AA_00892 | hypothetical protein CYLTODRAFT_431446 [Cylindrobasidium torrendii FP15055 ss-10] | 0.258022519 |
| chr08_AA_01027 | hypothetical protein CYLTODRAFT_357890 [Cylindrobasidium torrendii FP15055 ss-10] | chr09_AA_00955 | putative endo-1,4-beta-xylanase precursor [Flammulina velutipes] | 0.149990638 |
| chr08_AA_01027 | hypothetical protein CYLTODRAFT_357890 [Cylindrobasidium torrendii FP15055 ss-10] | chr09_AA_01058 | hypothetical protein ARMSODRAFT_976614 [Armillaria solidipes] | 0.2807245 |
| chr08_AA_01027 | hypothetical protein CYLTODRAFT_357890 [Cylindrobasidium torrendii FP15055 ss-10] | chr09_AA_01099 | general substrate transporter [Armillaria gallica] | 0.191679458 |
| chr08_AA_01027 | hypothetical protein CYLTODRAFT_357890 [Cylindrobasidium torrendii FP15055 ss-10] | chr09_AA_01103 | Metallo-dependent phosphatase [Armillaria solidipes] | 0.220875977 |
| chr08_AA_01027 | hypothetical protein CYLTODRAFT_357890 [Cylindrobasidium torrendii FP15055 ss-10] | chr09_AA_01106 | multidrug transporter [Cylindrobasidium torrendii FP15055 ss-10] | 0.165573383 |
| chr08_AA_01027 | hypothetical protein CYLTODRAFT_357890 [Cylindrobasidium torrendii FP15055 ss-10] | chr09_AA_01141 | hypothetical protein ARMGADRAFT_1063089 [Armillaria gallica] | 0.243103642 |
| chr08_AA_01027 | hypothetical protein CYLTODRAFT_357890 [Cylindrobasidium torrendii FP15055 ss-10] | chr09_AA_01190 | potassium/sodium eff [Cylindrobasidium torrendii FP15055 ss-10] | 0.19334503 |
| chr08_AA_01027 | hypothetical protein CYLTODRAFT_357890 [Cylindrobasidium torrendii FP15055 ss-10] | chr09_AA_01214 | hypothetical protein CYLTODRAFT_417942 [Cylindrobasidium torrendii FP15055 ss-10] | 0.214195141 |
| chr08_AA_01027 | hypothetical protein CYLTODRAFT_357890 [Cylindrobasidium torrendii FP15055 ss-10] | chr09_AA_01224 | uncharacterized protein ARMOST_22339 [Armillaria ostoyae] | 0.146468382 |
| chr08_AA_01027 | hypothetical protein CYLTODRAFT_357890 [Cylindrobasidium torrendii FP15055 ss-10] | chr09_AA_01225 | NAD(P)-binding protein [Cylindrobasidium torrendii FP15055 ss-10] | 0.127355519 |
| chr08_AA_01027 | hypothetical protein CYLTODRAFT_357890 [Cylindrobasidium torrendii FP15055 ss-10] | chr09_AA_01410 | hypothetical protein CYLTODRAFT_489277 [Cylindrobasidium torrendii FP15055 ss-10] | 0.178241918 |
| chr08_AA_01027 | hypothetical protein CYLTODRAFT_357890 [Cylindrobasidium torrendii FP15055 ss-10] | chr10_AA_00134 | hypothetical protein CYLTODRAFT_349864 [Cylindrobasidium torrendii FP15055 ss-10] | 0.170097176 |
| chr08_AA_01027 | hypothetical protein CYLTODRAFT_357890 [Cylindrobasidium torrendii FP15055 ss-10] | chr10_AA_00164 | dehydrogenase E1 and transketolase domain-containing protein 1 [Cylindrobasidium torrendii FP15055 ss-10] | 0.215779219 |
| chr08_AA_01027 | hypothetical protein CYLTODRAFT_357890 [Cylindrobasidium torrendii FP15055 ss-10] | chr10_AA_00220 | hypothetical protein ARMGADRAFT_962646, partial [Armillaria gallica] | 0.280934962 |
| chr08_AA_01027 | hypothetical protein CYLTODRAFT_357890 [Cylindrobasidium torrendii FP15055 ss-10] | chr10_AA_00256 | uncharacterized protein ARMOST_03672 [Armillaria ostoyae] | 0.222930886 |
| chr08_AA_01027 | hypothetical protein CYLTODRAFT_357890 [Cylindrobasidium torrendii FP15055 ss-10] | chr10_AA_00270 | branched-chain alpha-keto acid dehydrogenase E1-alpha subunit [Cylindrobasidium torrendii FP15055 ss-10] | 0.258032074 |
| chr08_AA_01027 | hypothetical protein CYLTODRAFT_357890 [Cylindrobasidium torrendii FP15055 ss-10] | chr10_AA_00441 | alpha/beta-hydrolase [Armillaria solidipes] | 0.273844542 |
| chr08_AA_01027 | hypothetical protein CYLTODRAFT_357890 [Cylindrobasidium torrendii FP15055 ss-10] | chr10_AA_00499 | hypothetical protein WG66_16220 [Moniliophthora roreri] | 0.234498071 |
| chr08_AA_01027 | hypothetical protein CYLTODRAFT_357890 [Cylindrobasidium torrendii FP15055 ss-10] | chr10_AA_00517 | hypothetical protein CYLTODRAFT_256211 [Cylindrobasidium torrendii FP15055 ss-10] | 0.22388645 |
| chr08_AA_01027 | hypothetical protein CYLTODRAFT_357890 [Cylindrobasidium torrendii FP15055 ss-10] | chr10_AA_00651 | hypothetical protein ARMGADRAFT_897215, partial [Armillaria gallica] | 0.243214708 |
| chr08_AA_01027 | hypothetical protein CYLTODRAFT_357890 [Cylindrobasidium torrendii FP15055 ss-10] | chr10_AA_00662 | Ureohydrolase [Armillaria gallica] | 0.261367144 |
| chr08_AA_01027 | hypothetical protein CYLTODRAFT_357890 [Cylindrobasidium torrendii FP15055 ss-10] | chr10_AA_00693 | hypothetical protein ARMSODRAFT_947720 [Armillaria solidipes] | 0.265924454 |
| chr08_AA_01027 | hypothetical protein CYLTODRAFT_357890 [Cylindrobasidium torrendii FP15055 ss-10] | chr10_AA_00735 | glycoside hydrolase family 31 protein [Cylindrobasidium torrendii FP15055 ss-10] | 0.17212492 |
| chr08_AA_01027 | hypothetical protein CYLTODRAFT_357890 [Cylindrobasidium torrendii FP15055 ss-10] | chr10_AA_00912 | hypothetical protein ARMSODRAFT_948409 [Armillaria solidipes] | 0.117444469 |
| chr08_AA_01027 | hypothetical protein CYLTODRAFT_357890 [Cylindrobasidium torrendii FP15055 ss-10] | chr10_AA_00944 | general substrate transporter [Cylindrobasidium torrendii FP15055 ss-10] | 0.224810858 |
| chr08_AA_01027 | hypothetical protein CYLTODRAFT_357890 [Cylindrobasidium torrendii FP15055 ss-10] | chr10_AA_01056 | hypothetical protein ARMGADRAFT_1020988 [Armillaria gallica] | 0.197097734 |
| chr08_AA_01027 | hypothetical protein CYLTODRAFT_357890 [Cylindrobasidium torrendii FP15055 ss-10] | chr10_AA_01063 | glycoside hydrolase family 43 protein [Amanita thiersii Skay4041] | 0.211731256 |
| chr08_AA_01027 | hypothetical protein CYLTODRAFT_357890 [Cylindrobasidium torrendii FP15055 ss-10] | chr10_AA_01143 | uncharacterized protein ARMOST_08409 [Armillaria ostoyae] | 0.23825473 |
| chr08_AA_01027 | hypothetical protein CYLTODRAFT_357890 [Cylindrobasidium torrendii FP15055 ss-10] | chr10_AA_01157 | glycoside hydrolase family 53 protein [Cylindrobasidium torrendii FP15055 ss-10] | 0.236126902 |
| chr08_AA_01027 | hypothetical protein CYLTODRAFT_357890 [Cylindrobasidium torrendii FP15055 ss-10] | chr10_AA_01201 | acyltransferase ChoActase/COT/CPT [Armillaria gallica] | 0.158225166 |
| chr08_AA_01027 | hypothetical protein CYLTODRAFT_357890 [Cylindrobasidium torrendii FP15055 ss-10] | chr10_AA_01213 | hypothetical protein JAAARDRAFT_210932 [Jaapia argillacea MUCL 33604] | 0.245495406 |
| chr08_AA_01027 | hypothetical protein CYLTODRAFT_357890 [Cylindrobasidium torrendii FP15055 ss-10] | chr10_AA_01231 | hypothetical protein ARMSODRAFT_1019532 [Armillaria solidipes] | 0.208551899 |
| chr08_AA_01027 | hypothetical protein CYLTODRAFT_357890 [Cylindrobasidium torrendii FP15055 ss-10] | chr10_AA_01309 | hypothetical protein PHLCEN_2v2282 [Phlebia centrifuga] | 0.206094293 |
| chr08_AA_01027 | hypothetical protein CYLTODRAFT_357890 [Cylindrobasidium torrendii FP15055 ss-10] | chr10_AA_01322 | putative laccase 5 [Flammulina velutipes] | 0.135940589 |
| chr08_AA_01027 | hypothetical protein CYLTODRAFT_357890 [Cylindrobasidium torrendii FP15055 ss-10] | chr10_AA_01341 | glucuronyl hydrolase [Armillaria solidipes] | 0.255369306 |
| chr08_AA_01027 | hypothetical protein CYLTODRAFT_357890 [Cylindrobasidium torrendii FP15055 ss-10] | chr10_AA_01576 | D-arabinitol 2-dehydrogenase [ribulose-forming] [Hypsizygus marmoreus] | 0.138110484 |
| chr08_AA_01027 | hypothetical protein CYLTODRAFT_357890 [Cylindrobasidium torrendii FP15055 ss-10] | chr10_AA_01664 | hypothetical protein CYLTODRAFT_404266 [Cylindrobasidium torrendii FP15055 ss-10] | 0.249292633 |
| chr08_AA_01027 | hypothetical protein CYLTODRAFT_357890 [Cylindrobasidium torrendii FP15055 ss-10] | chr11_AA_00042 | kinase-like protein [Cylindrobasidium torrendii FP15055 ss-10] | 0.216795509 |
| chr08_AA_01027 | hypothetical protein CYLTODRAFT_357890 [Cylindrobasidium torrendii FP15055 ss-10] | chr11_AA_00094 | homogentisate 1,2-dioxygenase [Armillaria solidipes] | 0.266675712 |
| chr08_AA_01027 | hypothetical protein CYLTODRAFT_357890 [Cylindrobasidium torrendii FP15055 ss-10] | chr11_AA_00103 | hypothetical protein M413DRAFT_370146 [Hebeloma cylindrosporum h7] | 0.20879576 |
| chr08_AA_01027 | hypothetical protein CYLTODRAFT_357890 [Cylindrobasidium torrendii FP15055 ss-10] | chr11_AA_00233 | hypothetical protein ARMGADRAFT_977028 [Armillaria gallica] | 0.230767233 |
| chr08_AA_01027 | hypothetical protein CYLTODRAFT_357890 [Cylindrobasidium torrendii FP15055 ss-10] | chr11_AA_00265 | FAD-linked oxidoreductase [Armillaria solidipes] | 0.194893659 |
| chr08_AA_01027 | hypothetical protein CYLTODRAFT_357890 [Cylindrobasidium torrendii FP15055 ss-10] | chr11_AA_00277 | uncharacterized protein ARMOST_10602 [Armillaria ostoyae] | 0.203958721 |
| chr08_AA_01027 | hypothetical protein CYLTODRAFT_357890 [Cylindrobasidium torrendii FP15055 ss-10] | chr11_AA_00499 | GroES-like protein [Armillaria solidipes] | 0.267039548 |
| chr08_AA_01027 | hypothetical protein CYLTODRAFT_357890 [Cylindrobasidium torrendii FP15055 ss-10] | chr11_AA_00504 | MFS general substrate transporter [Cylindrobasidium torrendii FP15055 ss-10] | 0.267795322 |
| chr08_AA_01027 | hypothetical protein CYLTODRAFT_357890 [Cylindrobasidium torrendii FP15055 ss-10] | chr11_AA_00581 | laccase [Flammulina velutipes] | 0.11552464 |
| chr08_AA_01027 | hypothetical protein CYLTODRAFT_357890 [Cylindrobasidium torrendii FP15055 ss-10] | chr11_AA_00606 | hypothetical protein CYLTODRAFT_418833 [Cylindrobasidium torrendii FP15055 ss-10] | 0.166823523 |
| chr08_AA_01027 | hypothetical protein CYLTODRAFT_357890 [Cylindrobasidium torrendii FP15055 ss-10] | chr11_AA_00638 | hypothetical protein ARMSODRAFT_953107 [Armillaria solidipes] | 0.244386277 |
| chr08_AA_01027 | hypothetical protein CYLTODRAFT_357890 [Cylindrobasidium torrendii FP15055 ss-10] | chr11_AA_00719 | hypothetical protein ARMGADRAFT_1019784, partial [Armillaria gallica] | 0.197582158 |
| chr08_AA_01027 | hypothetical protein CYLTODRAFT_357890 [Cylindrobasidium torrendii FP15055 ss-10] | chr11_AA_00724 | WD40 repeat-like protein [Armillaria solidipes] | 0.298950619 |
| chr08_AA_01027 | hypothetical protein CYLTODRAFT_357890 [Cylindrobasidium torrendii FP15055 ss-10] | chr11_AA_00790 | uncharacterized protein ARMOST_05401 [Armillaria ostoyae] | 0.119397113 |
| chr08_AA_01027 | hypothetical protein CYLTODRAFT_357890 [Cylindrobasidium torrendii FP15055 ss-10] | chr11_AA_00878 | hypothetical protein CYLTODRAFT_430286 [Cylindrobasidium torrendii FP15055 ss-10] | 0.216910679 |
| chr08_AA_01027 | hypothetical protein CYLTODRAFT_357890 [Cylindrobasidium torrendii FP15055 ss-10] | chr11_AA_00928 | putative malate synthase [Flammulina velutipes] | 0.22052917 |
| chr08_AA_01027 | hypothetical protein CYLTODRAFT_357890 [Cylindrobasidium torrendii FP15055 ss-10] | chr11_AA_00997 | Annexin [Cylindrobasidium torrendii FP15055 ss-10] | 0.197661398 |
| chr08_AA_01027 | hypothetical protein CYLTODRAFT_357890 [Cylindrobasidium torrendii FP15055 ss-10] | chr11_AA_01033 | glycoside hydrolase family 51 protein [Cylindrobasidium torrendii FP15055 ss-10] | 0.270739419 |
| chr08_AA_01027 | hypothetical protein CYLTODRAFT_357890 [Cylindrobasidium torrendii FP15055 ss-10] | chr11_AA_01042 | acetoin reductase family protein [Cylindrobasidium torrendii FP15055 ss-10] | 0.135407081 |
| chr08_AA_01027 | hypothetical protein CYLTODRAFT_357890 [Cylindrobasidium torrendii FP15055 ss-10] | chr11_AA_01046 | hypothetical protein CYLTODRAFT_428160 [Cylindrobasidium torrendii FP15055 ss-10] | 0.180674646 |
| chr08_AA_01027 | hypothetical protein CYLTODRAFT_357890 [Cylindrobasidium torrendii FP15055 ss-10] | chr11_AA_01097 | glycoside hydrolase family 3 protein [Cylindrobasidium torrendii FP15055 ss-10] | 0.15241103 |
| chr08_AA_01027 | hypothetical protein CYLTODRAFT_357890 [Cylindrobasidium torrendii FP15055 ss-10] | chr11_AA_01106 | related to stomatin [Armillaria ostoyae] | 0.152899136 |
| chr08_AA_01027 | hypothetical protein CYLTODRAFT_357890 [Cylindrobasidium torrendii FP15055 ss-10] | chr11_AA_01313 | hypothetical protein Hypma_000372 [Hypsizygus marmoreus] | 0.268187947 |
| chr08_AA_01027 | hypothetical protein CYLTODRAFT_357890 [Cylindrobasidium torrendii FP15055 ss-10] | chr11_AA_01324 | terpenoid synthase [Cylindrobasidium torrendii FP15055 ss-10] | 0.260576999 |
| chr08_AA_01027 | hypothetical protein CYLTODRAFT_357890 [Cylindrobasidium torrendii FP15055 ss-10] | chr11_AA_01456 | uncharacterized protein ARMOST_16541 [Armillaria ostoyae] | 0.196008101 |
| chr08_AA_01027 | hypothetical protein CYLTODRAFT_357890 [Cylindrobasidium torrendii FP15055 ss-10] | chr11_AA_01461 | ricin B-like lectin [Armillaria gallica] | 0.194734191 |
| chr08_AA_01027 | hypothetical protein CYLTODRAFT_357890 [Cylindrobasidium torrendii FP15055 ss-10] | chr11_AA_01636 | hypothetical protein CYLTODRAFT_421880 [Cylindrobasidium torrendii FP15055 ss-10] | 0.19864105 |
| chr08_AA_01027 | hypothetical protein CYLTODRAFT_357890 [Cylindrobasidium torrendii FP15055 ss-10] | chr11_AA_01644 | hypothetical protein CYLTODRAFT_438918 [Cylindrobasidium torrendii FP15055 ss-10] | 0.174881714 |
| chr08_AA_01027 | hypothetical protein CYLTODRAFT_357890 [Cylindrobasidium torrendii FP15055 ss-10] | chr11_AA_01664 | hypothetical protein CYLTODRAFT_421876 [Cylindrobasidium torrendii FP15055 ss-10] | 0.147351254 |
| chr08_AA_01027 | hypothetical protein CYLTODRAFT_357890 [Cylindrobasidium torrendii FP15055 ss-10] | chr11_AA_01685 | 1-aminocyclopropane-1-carboxylate deaminase [Armillaria solidipes] | 0.161151596 |
| chr08_AA_01027 | hypothetical protein CYLTODRAFT_357890 [Cylindrobasidium torrendii FP15055 ss-10] | chr11_AA_01686 | hypothetical protein CYLTODRAFT_348076 [Cylindrobasidium torrendii FP15055 ss-10] | 0.280723115 |
| chr08_AA_01027 | hypothetical protein CYLTODRAFT_357890 [Cylindrobasidium torrendii FP15055 ss-10] | chr11_AA_01711 | hypothetical protein ARMGADRAFT_1046867 [Armillaria gallica] | 0.263937691 |
| chr08_AA_01027 | hypothetical protein CYLTODRAFT_357890 [Cylindrobasidium torrendii FP15055 ss-10] | chr11_AA_01731 | benzoquinone reductase [Cylindrobasidium torrendii FP15055 ss-10] | 0.140978765 |
| chr08_AA_01027 | hypothetical protein CYLTODRAFT_357890 [Cylindrobasidium torrendii FP15055 ss-10] | chr11_AA_01732 | glycoside hydrolase family 76 protein [Cylindrobasidium torrendii FP15055 ss-10] | 0.196980489 |
| chr08_AA_01027 | hypothetical protein CYLTODRAFT_357890 [Cylindrobasidium torrendii FP15055 ss-10] | chr11_AA_01733 | glycoside hydrolase family 76 protein [Cylindrobasidium torrendii FP15055 ss-10] | 0.203555239 |
| chr11_AA_01033 | glycoside hydrolase family 51 protein [Cylindrobasidium torrendii FP15055 ss-10] | chr11_AA_01042 | acetoin reductase family protein [Cylindrobasidium torrendii FP15055 ss-10] | 0.144557294 |
| chr11_AA_01033 | glycoside hydrolase family 51 protein [Cylindrobasidium torrendii FP15055 ss-10] | chr11_AA_01046 | hypothetical protein CYLTODRAFT_428160 [Cylindrobasidium torrendii FP15055 ss-10] | 0.194116965 |
| chr11_AA_01033 | glycoside hydrolase family 51 protein [Cylindrobasidium torrendii FP15055 ss-10] | chr11_AA_01097 | glycoside hydrolase family 3 protein [Cylindrobasidium torrendii FP15055 ss-10] | 0.154577155 |
| chr11_AA_01033 | glycoside hydrolase family 51 protein [Cylindrobasidium torrendii FP15055 ss-10] | chr11_AA_01106 | related to stomatin [Armillaria ostoyae] | 0.166486305 |
| chr11_AA_01033 | glycoside hydrolase family 51 protein [Cylindrobasidium torrendii FP15055 ss-10] | chr11_AA_01313 | hypothetical protein Hypma_000372 [Hypsizygus marmoreus] | 0.262287218 |
| chr11_AA_01033 | glycoside hydrolase family 51 protein [Cylindrobasidium torrendii FP15055 ss-10] | chr11_AA_01324 | terpenoid synthase [Cylindrobasidium torrendii FP15055 ss-10] | 0.213795713 |
| chr11_AA_01033 | glycoside hydrolase family 51 protein [Cylindrobasidium torrendii FP15055 ss-10] | chr11_AA_01456 | uncharacterized protein ARMOST_16541 [Armillaria ostoyae] | 0.198146859 |
| chr11_AA_01033 | glycoside hydrolase family 51 protein [Cylindrobasidium torrendii FP15055 ss-10] | chr11_AA_01461 | ricin B-like lectin [Armillaria gallica] | 0.183710189 |
| chr11_AA_01033 | glycoside hydrolase family 51 protein [Cylindrobasidium torrendii FP15055 ss-10] | chr11_AA_01636 | hypothetical protein CYLTODRAFT_421880 [Cylindrobasidium torrendii FP15055 ss-10] | 0.221441734 |
| chr11_AA_01033 | glycoside hydrolase family 51 protein [Cylindrobasidium torrendii FP15055 ss-10] | chr11_AA_01644 | hypothetical protein CYLTODRAFT_438918 [Cylindrobasidium torrendii FP15055 ss-10] | 0.167735089 |
| chr11_AA_01033 | glycoside hydrolase family 51 protein [Cylindrobasidium torrendii FP15055 ss-10] | chr11_AA_01664 | hypothetical protein CYLTODRAFT_421876 [Cylindrobasidium torrendii FP15055 ss-10] | 0.159099282 |
| chr11_AA_01033 | glycoside hydrolase family 51 protein [Cylindrobasidium torrendii FP15055 ss-10] | chr11_AA_01685 | 1-aminocyclopropane-1-carboxylate deaminase [Armillaria solidipes] | 0.16347996 |
| chr11_AA_01033 | glycoside hydrolase family 51 protein [Cylindrobasidium torrendii FP15055 ss-10] | chr11_AA_01686 | hypothetical protein CYLTODRAFT_348076 [Cylindrobasidium torrendii FP15055 ss-10] | 0.270625716 |
| chr11_AA_01033 | glycoside hydrolase family 51 protein [Cylindrobasidium torrendii FP15055 ss-10] | chr11_AA_01711 | hypothetical protein ARMGADRAFT_1046867 [Armillaria gallica] | 0.278544962 |
| chr11_AA_01033 | glycoside hydrolase family 51 protein [Cylindrobasidium torrendii FP15055 ss-10] | chr11_AA_01731 | benzoquinone reductase [Cylindrobasidium torrendii FP15055 ss-10] | 0.127636808 |
| chr11_AA_01033 | glycoside hydrolase family 51 protein [Cylindrobasidium torrendii FP15055 ss-10] | chr11_AA_01732 | glycoside hydrolase family 76 protein [Cylindrobasidium torrendii FP15055 ss-10] | 0.192346238 |
| chr11_AA_01033 | glycoside hydrolase family 51 protein [Cylindrobasidium torrendii FP15055 ss-10] | chr11_AA_01733 | glycoside hydrolase family 76 protein [Cylindrobasidium torrendii FP15055 ss-10] | 0.208859744 |
| chr10_AA_00693 | hypothetical protein ARMSODRAFT_947720 [Armillaria solidipes] | chr10_AA_00733 | Zinc-regulated transporter 1 [Hypsizygus marmoreus] | 0.189680508 |
| chr10_AA_00693 | hypothetical protein ARMSODRAFT_947720 [Armillaria solidipes] | chr10_AA_00735 | glycoside hydrolase family 31 protein [Cylindrobasidium torrendii FP15055 ss-10] | 0.202518169 |
| chr10_AA_00693 | hypothetical protein ARMSODRAFT_947720 [Armillaria solidipes] | chr10_AA_00944 | general substrate transporter [Cylindrobasidium torrendii FP15055 ss-10] | 0.26762573 |
| chr10_AA_00693 | hypothetical protein ARMSODRAFT_947720 [Armillaria solidipes] | chr10_AA_01056 | hypothetical protein ARMGADRAFT_1020988 [Armillaria gallica] | 0.131876217 |
| chr10_AA_00693 | hypothetical protein ARMSODRAFT_947720 [Armillaria solidipes] | chr10_AA_01063 | glycoside hydrolase family 43 protein [Amanita thiersii Skay4041] | 0.27215411 |
| chr10_AA_00693 | hypothetical protein ARMSODRAFT_947720 [Armillaria solidipes] | chr10_AA_01143 | uncharacterized protein ARMOST_08409 [Armillaria ostoyae] | 0.260900941 |
| chr10_AA_00693 | hypothetical protein ARMSODRAFT_947720 [Armillaria solidipes] | chr10_AA_01157 | glycoside hydrolase family 53 protein [Cylindrobasidium torrendii FP15055 ss-10] | 0.317435921 |
| chr10_AA_00693 | hypothetical protein ARMSODRAFT_947720 [Armillaria solidipes] | chr10_AA_01201 | acyltransferase ChoActase/COT/CPT [Armillaria gallica] | 0.270345753 |
| chr10_AA_00693 | hypothetical protein ARMSODRAFT_947720 [Armillaria solidipes] | chr10_AA_01213 | hypothetical protein JAAARDRAFT_210932 [Jaapia argillacea MUCL 33604] | 0.20939695 |
| chr10_AA_00693 | hypothetical protein ARMSODRAFT_947720 [Armillaria solidipes] | chr10_AA_01231 | hypothetical protein ARMSODRAFT_1019532 [Armillaria solidipes] | 0.255111781 |
| chr10_AA_00693 | hypothetical protein ARMSODRAFT_947720 [Armillaria solidipes] | chr10_AA_01309 | hypothetical protein PHLCEN_2v2282 [Phlebia centrifuga] | 0.125620013 |
| chr10_AA_00693 | hypothetical protein ARMSODRAFT_947720 [Armillaria solidipes] | chr10_AA_01322 | putative laccase 5 [Flammulina velutipes] | 0.108881975 |
| chr10_AA_00693 | hypothetical protein ARMSODRAFT_947720 [Armillaria solidipes] | chr10_AA_01341 | glucuronyl hydrolase [Armillaria solidipes] | 0.316246532 |
| chr10_AA_00693 | hypothetical protein ARMSODRAFT_947720 [Armillaria solidipes] | chr10_AA_01576 | D-arabinitol 2-dehydrogenase [ribulose-forming] [Hypsizygus marmoreus] | 0.257601296 |
| chr10_AA_00693 | hypothetical protein ARMSODRAFT_947720 [Armillaria solidipes] | chr10_AA_01664 | hypothetical protein CYLTODRAFT_404266 [Cylindrobasidium torrendii FP15055 ss-10] | 0.306590828 |
| chr10_AA_00693 | hypothetical protein ARMSODRAFT_947720 [Armillaria solidipes] | chr11_AA_00027 | hypothetical protein CYLTODRAFT_423477 [Cylindrobasidium torrendii FP15055 ss-10] | 0.133225324 |
| chr10_AA_00693 | hypothetical protein ARMSODRAFT_947720 [Armillaria solidipes] | chr11_AA_00042 | kinase-like protein [Cylindrobasidium torrendii FP15055 ss-10] | 0.292581414 |
| chr10_AA_00693 | hypothetical protein ARMSODRAFT_947720 [Armillaria solidipes] | chr11_AA_00094 | homogentisate 1,2-dioxygenase [Armillaria solidipes] | 0.177087949 |
| chr10_AA_00693 | hypothetical protein ARMSODRAFT_947720 [Armillaria solidipes] | chr11_AA_00103 | hypothetical protein M413DRAFT_370146 [Hebeloma cylindrosporum h7] | 0.292886743 |
| chr10_AA_00693 | hypothetical protein ARMSODRAFT_947720 [Armillaria solidipes] | chr11_AA_00145 | hypothetical protein CYLTODRAFT_243491 [Cylindrobasidium torrendii FP15055 ss-10] | 0.188021275 |
| chr10_AA_00693 | hypothetical protein ARMSODRAFT_947720 [Armillaria solidipes] | chr11_AA_00233 | hypothetical protein ARMGADRAFT_977028 [Armillaria gallica] | 0.143502813 |
| chr10_AA_00693 | hypothetical protein ARMSODRAFT_947720 [Armillaria solidipes] | chr11_AA_00265 | FAD-linked oxidoreductase [Armillaria solidipes] | 0.271121836 |
| chr10_AA_00693 | hypothetical protein ARMSODRAFT_947720 [Armillaria solidipes] | chr11_AA_00277 | uncharacterized protein ARMOST_10602 [Armillaria ostoyae] | 0.141549958 |
| chr10_AA_00693 | hypothetical protein ARMSODRAFT_947720 [Armillaria solidipes] | chr11_AA_00499 | GroES-like protein [Armillaria solidipes] | 0.275623365 |
| chr10_AA_00693 | hypothetical protein ARMSODRAFT_947720 [Armillaria solidipes] | chr11_AA_00504 | MFS general substrate transporter [Cylindrobasidium torrendii FP15055 ss-10] | 0.292675236 |
| chr10_AA_00693 | hypothetical protein ARMSODRAFT_947720 [Armillaria solidipes] | chr11_AA_00606 | hypothetical protein CYLTODRAFT_418833 [Cylindrobasidium torrendii FP15055 ss-10] | 0.262162254 |
| chr10_AA_00693 | hypothetical protein ARMSODRAFT_947720 [Armillaria solidipes] | chr11_AA_00638 | hypothetical protein ARMSODRAFT_953107 [Armillaria solidipes] | 0.180655794 |
| chr10_AA_00693 | hypothetical protein ARMSODRAFT_947720 [Armillaria solidipes] | chr11_AA_00701 | uncharacterized protein ARMOST_05344 [Armillaria ostoyae] | 0.175782263 |
| chr10_AA_00693 | hypothetical protein ARMSODRAFT_947720 [Armillaria solidipes] | chr11_AA_00719 | hypothetical protein ARMGADRAFT_1019784, partial [Armillaria gallica] | 0.133416118 |
| chr10_AA_00693 | hypothetical protein ARMSODRAFT_947720 [Armillaria solidipes] | chr11_AA_00724 | WD40 repeat-like protein [Armillaria solidipes] | 0.343658709 |
| chr10_AA_00693 | hypothetical protein ARMSODRAFT_947720 [Armillaria solidipes] | chr11_AA_00790 | uncharacterized protein ARMOST_05401 [Armillaria ostoyae] | 0.243019451 |
| chr10_AA_00693 | hypothetical protein ARMSODRAFT_947720 [Armillaria solidipes] | chr11_AA_00878 | hypothetical protein CYLTODRAFT_430286 [Cylindrobasidium torrendii FP15055 ss-10] | 0.282325736 |
| chr10_AA_00693 | hypothetical protein ARMSODRAFT_947720 [Armillaria solidipes] | chr11_AA_00928 | putative malate synthase [Flammulina velutipes] | 0.30368841 |
| chr10_AA_00693 | hypothetical protein ARMSODRAFT_947720 [Armillaria solidipes] | chr11_AA_00997 | Annexin [Cylindrobasidium torrendii FP15055 ss-10] | 0.230377628 |
| chr10_AA_00693 | hypothetical protein ARMSODRAFT_947720 [Armillaria solidipes] | chr11_AA_01033 | glycoside hydrolase family 51 protein [Cylindrobasidium torrendii FP15055 ss-10] | 0.27888494 |
| chr10_AA_00693 | hypothetical protein ARMSODRAFT_947720 [Armillaria solidipes] | chr11_AA_01042 | acetoin reductase family protein [Cylindrobasidium torrendii FP15055 ss-10] | 0.166153259 |
| chr10_AA_00693 | hypothetical protein ARMSODRAFT_947720 [Armillaria solidipes] | chr11_AA_01046 | hypothetical protein CYLTODRAFT_428160 [Cylindrobasidium torrendii FP15055 ss-10] | 0.258689771 |
| chr10_AA_00693 | hypothetical protein ARMSODRAFT_947720 [Armillaria solidipes] | chr11_AA_01097 | glycoside hydrolase family 3 protein [Cylindrobasidium torrendii FP15055 ss-10] | 0.262887522 |
| chr10_AA_00693 | hypothetical protein ARMSODRAFT_947720 [Armillaria solidipes] | chr11_AA_01106 | related to stomatin [Armillaria ostoyae] | 0.204131866 |
| chr10_AA_00693 | hypothetical protein ARMSODRAFT_947720 [Armillaria solidipes] | chr11_AA_01313 | hypothetical protein Hypma_000372 [Hypsizygus marmoreus] | 0.237800996 |
| chr10_AA_00693 | hypothetical protein ARMSODRAFT_947720 [Armillaria solidipes] | chr11_AA_01324 | terpenoid synthase [Cylindrobasidium torrendii FP15055 ss-10] | 0.176138365 |
| chr10_AA_00693 | hypothetical protein ARMSODRAFT_947720 [Armillaria solidipes] | chr11_AA_01445 | hypothetical protein ARMSODRAFT_950555 [Armillaria solidipes] &gt;SJL10115.1 uncharacterized protein ARMOST_13499 [Armillaria ostoyae] | 0.147429918 |
| chr10_AA_00693 | hypothetical protein ARMSODRAFT_947720 [Armillaria solidipes] | chr11_AA_01456 | uncharacterized protein ARMOST_16541 [Armillaria ostoyae] | 0.210950626 |
| chr10_AA_00693 | hypothetical protein ARMSODRAFT_947720 [Armillaria solidipes] | chr11_AA_01461 | ricin B-like lectin [Armillaria gallica] | 0.226231807 |
| chr10_AA_00693 | hypothetical protein ARMSODRAFT_947720 [Armillaria solidipes] | chr11_AA_01510 | hypothetical protein ARMGADRAFT_1015707 [Armillaria gallica] | 0.186958536 |
| chr10_AA_00693 | hypothetical protein ARMSODRAFT_947720 [Armillaria solidipes] | chr11_AA_01636 | hypothetical protein CYLTODRAFT_421880 [Cylindrobasidium torrendii FP15055 ss-10] | 0.248111132 |
| chr10_AA_00693 | hypothetical protein ARMSODRAFT_947720 [Armillaria solidipes] | chr11_AA_01644 | hypothetical protein CYLTODRAFT_438918 [Cylindrobasidium torrendii FP15055 ss-10] | 0.128827309 |
| chr10_AA_00693 | hypothetical protein ARMSODRAFT_947720 [Armillaria solidipes] | chr11_AA_01664 | hypothetical protein CYLTODRAFT_421876 [Cylindrobasidium torrendii FP15055 ss-10] | 0.24786329 |
| chr10_AA_00693 | hypothetical protein ARMSODRAFT_947720 [Armillaria solidipes] | chr11_AA_01685 | 1-aminocyclopropane-1-carboxylate deaminase [Armillaria solidipes] | 0.204843151 |
| chr10_AA_00693 | hypothetical protein ARMSODRAFT_947720 [Armillaria solidipes] | chr11_AA_01686 | hypothetical protein CYLTODRAFT_348076 [Cylindrobasidium torrendii FP15055 ss-10] | 0.22862488 |
| chr10_AA_00693 | hypothetical protein ARMSODRAFT_947720 [Armillaria solidipes] | chr11_AA_01711 | hypothetical protein ARMGADRAFT_1046867 [Armillaria gallica] | 0.272990894 |
| chr10_AA_00693 | hypothetical protein ARMSODRAFT_947720 [Armillaria solidipes] | chr11_AA_01732 | glycoside hydrolase family 76 protein [Cylindrobasidium torrendii FP15055 ss-10] | 0.238934958 |
| chr10_AA_00693 | hypothetical protein ARMSODRAFT_947720 [Armillaria solidipes] | chr11_AA_01733 | glycoside hydrolase family 76 protein [Cylindrobasidium torrendii FP15055 ss-10] | 0.239373291 |
| chr09_AA_00241 | SNF2 chromatin remodeling protein [Armillaria solidipes] | chr09_AA_00312 | putative beta-glucosidase [Flammulina velutipes] | 0.22414486 |
| chr09_AA_00241 | SNF2 chromatin remodeling protein [Armillaria solidipes] | chr09_AA_00406 | uncharacterized protein ARMOST_17180 [Armillaria ostoyae] | 0.275631541 |
| chr09_AA_00241 | SNF2 chromatin remodeling protein [Armillaria solidipes] | chr09_AA_00518 | AAA-domain-containing protein [Cylindrobasidium torrendii FP15055 ss-10] | 0.294819004 |
| chr09_AA_00241 | SNF2 chromatin remodeling protein [Armillaria solidipes] | chr09_AA_00551 | hypothetical protein CYLTODRAFT_392634 [Cylindrobasidium torrendii FP15055 ss-10] | 0.120551583 |
| chr09_AA_00241 | SNF2 chromatin remodeling protein [Armillaria solidipes] | chr09_AA_00680 | hypothetical protein CYLTODRAFT_395198 [Cylindrobasidium torrendii FP15055 ss-10] | 0.253973235 |
| chr09_AA_00241 | SNF2 chromatin remodeling protein [Armillaria solidipes] | chr09_AA_00719 | hypothetical protein PLEOSDRAFT_1093927 [Pleurotus ostreatus PC15] | 0.205481786 |
| chr09_AA_00241 | SNF2 chromatin remodeling protein [Armillaria solidipes] | chr09_AA_00816 | mandelate racemase muconate lactonizing enzyme family protein [Cylindrobasidium torrendii FP15055 ss-10] | 0.109709168 |
| chr09_AA_00241 | SNF2 chromatin remodeling protein [Armillaria solidipes] | chr09_AA_00838 | cytochrome P450 [Armillaria gallica] | 0.193092155 |
| chr09_AA_00241 | SNF2 chromatin remodeling protein [Armillaria solidipes] | chr09_AA_00892 | hypothetical protein CYLTODRAFT_431446 [Cylindrobasidium torrendii FP15055 ss-10] | 0.246434993 |
| chr09_AA_00241 | SNF2 chromatin remodeling protein [Armillaria solidipes] | chr09_AA_00955 | putative endo-1,4-beta-xylanase precursor [Flammulina velutipes] | 0.162306682 |
| chr09_AA_00241 | SNF2 chromatin remodeling protein [Armillaria solidipes] | chr09_AA_01058 | hypothetical protein ARMSODRAFT_976614 [Armillaria solidipes] | 0.281005579 |
| chr09_AA_00241 | SNF2 chromatin remodeling protein [Armillaria solidipes] | chr09_AA_01099 | general substrate transporter [Armillaria gallica] | 0.165480815 |
| chr09_AA_00241 | SNF2 chromatin remodeling protein [Armillaria solidipes] | chr09_AA_01103 | Metallo-dependent phosphatase [Armillaria solidipes] | 0.19758601 |
| chr09_AA_00241 | SNF2 chromatin remodeling protein [Armillaria solidipes] | chr09_AA_01106 | multidrug transporter [Cylindrobasidium torrendii FP15055 ss-10] | 0.121780698 |
| chr09_AA_00241 | SNF2 chromatin remodeling protein [Armillaria solidipes] | chr09_AA_01141 | hypothetical protein ARMGADRAFT_1063089 [Armillaria gallica] | 0.262922995 |
| chr09_AA_00241 | SNF2 chromatin remodeling protein [Armillaria solidipes] | chr09_AA_01190 | potassium/sodium eff [Cylindrobasidium torrendii FP15055 ss-10] | 0.202237876 |
| chr09_AA_00241 | SNF2 chromatin remodeling protein [Armillaria solidipes] | chr09_AA_01214 | hypothetical protein CYLTODRAFT_417942 [Cylindrobasidium torrendii FP15055 ss-10] | 0.203838054 |
| chr09_AA_00241 | SNF2 chromatin remodeling protein [Armillaria solidipes] | chr09_AA_01224 | uncharacterized protein ARMOST_22339 [Armillaria ostoyae] | 0.115464428 |
| chr09_AA_00241 | SNF2 chromatin remodeling protein [Armillaria solidipes] | chr09_AA_01225 | NAD(P)-binding protein [Cylindrobasidium torrendii FP15055 ss-10] | 0.145171107 |
| chr09_AA_00241 | SNF2 chromatin remodeling protein [Armillaria solidipes] | chr09_AA_01324 | uncharacterized protein ARMOST_03294 [Armillaria ostoyae] | 0.113981878 |
| chr09_AA_00241 | SNF2 chromatin remodeling protein [Armillaria solidipes] | chr09_AA_01410 | hypothetical protein CYLTODRAFT_489277 [Cylindrobasidium torrendii FP15055 ss-10] | 0.224368267 |
| chr09_AA_00241 | SNF2 chromatin remodeling protein [Armillaria solidipes] | chr10_AA_00134 | hypothetical protein CYLTODRAFT_349864 [Cylindrobasidium torrendii FP15055 ss-10] | 0.175229549 |
| chr09_AA_00241 | SNF2 chromatin remodeling protein [Armillaria solidipes] | chr10_AA_00164 | dehydrogenase E1 and transketolase domain-containing protein 1 [Cylindrobasidium torrendii FP15055 ss-10] | 0.199295627 |
| chr09_AA_00241 | SNF2 chromatin remodeling protein [Armillaria solidipes] | chr10_AA_00220 | hypothetical protein ARMGADRAFT_962646, partial [Armillaria gallica] | 0.277140173 |
| chr09_AA_00241 | SNF2 chromatin remodeling protein [Armillaria solidipes] | chr10_AA_00256 | uncharacterized protein ARMOST_03672 [Armillaria ostoyae] | 0.242469341 |
| chr09_AA_00241 | SNF2 chromatin remodeling protein [Armillaria solidipes] | chr10_AA_00270 | branched-chain alpha-keto acid dehydrogenase E1-alpha subunit [Cylindrobasidium torrendii FP15055 ss-10] | 0.268765255 |
| chr09_AA_00241 | SNF2 chromatin remodeling protein [Armillaria solidipes] | chr10_AA_00441 | alpha/beta-hydrolase [Armillaria solidipes] | 0.267341069 |
| chr09_AA_00241 | SNF2 chromatin remodeling protein [Armillaria solidipes] | chr10_AA_00499 | hypothetical protein WG66_16220 [Moniliophthora roreri] | 0.207315153 |
| chr09_AA_00241 | SNF2 chromatin remodeling protein [Armillaria solidipes] | chr10_AA_00517 | hypothetical protein CYLTODRAFT_256211 [Cylindrobasidium torrendii FP15055 ss-10] | 0.247639392 |
| chr09_AA_00241 | SNF2 chromatin remodeling protein [Armillaria solidipes] | chr10_AA_00651 | hypothetical protein ARMGADRAFT_897215, partial [Armillaria gallica] | 0.219213511 |
| chr09_AA_00241 | SNF2 chromatin remodeling protein [Armillaria solidipes] | chr10_AA_00662 | Ureohydrolase [Armillaria gallica] | 0.245105009 |
| chr09_AA_00241 | SNF2 chromatin remodeling protein [Armillaria solidipes] | chr10_AA_00693 | hypothetical protein ARMSODRAFT_947720 [Armillaria solidipes] | 0.243993698 |
| chr09_AA_00241 | SNF2 chromatin remodeling protein [Armillaria solidipes] | chr10_AA_00735 | glycoside hydrolase family 31 protein [Cylindrobasidium torrendii FP15055 ss-10] | 0.192081825 |
| chr09_AA_00241 | SNF2 chromatin remodeling protein [Armillaria solidipes] | chr10_AA_00912 | hypothetical protein ARMSODRAFT_948409 [Armillaria solidipes] | 0.140483447 |
| chr09_AA_00241 | SNF2 chromatin remodeling protein [Armillaria solidipes] | chr10_AA_00944 | general substrate transporter [Cylindrobasidium torrendii FP15055 ss-10] | 0.199072013 |
| chr09_AA_00241 | SNF2 chromatin remodeling protein [Armillaria solidipes] | chr10_AA_01056 | hypothetical protein ARMGADRAFT_1020988 [Armillaria gallica] | 0.241608063 |
| chr09_AA_00241 | SNF2 chromatin remodeling protein [Armillaria solidipes] | chr10_AA_01063 | glycoside hydrolase family 43 protein [Amanita thiersii Skay4041] | 0.181560747 |
| chr09_AA_00241 | SNF2 chromatin remodeling protein [Armillaria solidipes] | chr10_AA_01143 | uncharacterized protein ARMOST_08409 [Armillaria ostoyae] | 0.228445183 |
| chr09_AA_00241 | SNF2 chromatin remodeling protein [Armillaria solidipes] | chr10_AA_01157 | glycoside hydrolase family 53 protein [Cylindrobasidium torrendii FP15055 ss-10] | 0.203762777 |
| chr09_AA_00241 | SNF2 chromatin remodeling protein [Armillaria solidipes] | chr10_AA_01201 | acyltransferase ChoActase/COT/CPT [Armillaria gallica] | 0.135937293 |
| chr09_AA_00241 | SNF2 chromatin remodeling protein [Armillaria solidipes] | chr10_AA_01213 | hypothetical protein JAAARDRAFT_210932 [Jaapia argillacea MUCL 33604] | 0.288408654 |
| chr09_AA_00241 | SNF2 chromatin remodeling protein [Armillaria solidipes] | chr10_AA_01231 | hypothetical protein ARMSODRAFT_1019532 [Armillaria solidipes] | 0.210873444 |
| chr09_AA_00241 | SNF2 chromatin remodeling protein [Armillaria solidipes] | chr10_AA_01309 | hypothetical protein PHLCEN_2v2282 [Phlebia centrifuga] | 0.203053191 |
| chr09_AA_00241 | SNF2 chromatin remodeling protein [Armillaria solidipes] | chr10_AA_01322 | putative laccase 5 [Flammulina velutipes] | 0.146082144 |
| chr09_AA_00241 | SNF2 chromatin remodeling protein [Armillaria solidipes] | chr10_AA_01341 | glucuronyl hydrolase [Armillaria solidipes] | 0.209175566 |
| chr09_AA_00241 | SNF2 chromatin remodeling protein [Armillaria solidipes] | chr10_AA_01390 | hypothetical protein CYLTODRAFT_414265 [Cylindrobasidium torrendii FP15055 ss-10] | 0.112557816 |
| chr09_AA_00241 | SNF2 chromatin remodeling protein [Armillaria solidipes] | chr10_AA_01576 | D-arabinitol 2-dehydrogenase [ribulose-forming] [Hypsizygus marmoreus] | 0.118758041 |
| chr09_AA_00241 | SNF2 chromatin remodeling protein [Armillaria solidipes] | chr10_AA_01664 | hypothetical protein CYLTODRAFT_404266 [Cylindrobasidium torrendii FP15055 ss-10] | 0.220819517 |
| chr09_AA_00241 | SNF2 chromatin remodeling protein [Armillaria solidipes] | chr11_AA_00042 | kinase-like protein [Cylindrobasidium torrendii FP15055 ss-10] | 0.192608948 |
| chr09_AA_00241 | SNF2 chromatin remodeling protein [Armillaria solidipes] | chr11_AA_00094 | homogentisate 1,2-dioxygenase [Armillaria solidipes] | 0.292137718 |
| chr09_AA_00241 | SNF2 chromatin remodeling protein [Armillaria solidipes] | chr11_AA_00103 | hypothetical protein M413DRAFT_370146 [Hebeloma cylindrosporum h7] | 0.204341626 |
| chr09_AA_00241 | SNF2 chromatin remodeling protein [Armillaria solidipes] | chr11_AA_00233 | hypothetical protein ARMGADRAFT_977028 [Armillaria gallica] | 0.281673962 |
| chr09_AA_00241 | SNF2 chromatin remodeling protein [Armillaria solidipes] | chr11_AA_00265 | FAD-linked oxidoreductase [Armillaria solidipes] | 0.180612172 |
| chr09_AA_00241 | SNF2 chromatin remodeling protein [Armillaria solidipes] | chr11_AA_00277 | uncharacterized protein ARMOST_10602 [Armillaria ostoyae] | 0.251819743 |
| chr09_AA_00241 | SNF2 chromatin remodeling protein [Armillaria solidipes] | chr11_AA_00499 | GroES-like protein [Armillaria solidipes] | 0.274576019 |
| chr09_AA_00241 | SNF2 chromatin remodeling protein [Armillaria solidipes] | chr11_AA_00504 | MFS general substrate transporter [Cylindrobasidium torrendii FP15055 ss-10] | 0.249885616 |
| chr09_AA_00241 | SNF2 chromatin remodeling protein [Armillaria solidipes] | chr11_AA_00581 | laccase [Flammulina velutipes] | 0.124944821 |
| chr09_AA_00241 | SNF2 chromatin remodeling protein [Armillaria solidipes] | chr11_AA_00606 | hypothetical protein CYLTODRAFT_418833 [Cylindrobasidium torrendii FP15055 ss-10] | 0.148724123 |
| chr09_AA_00241 | SNF2 chromatin remodeling protein [Armillaria solidipes] | chr11_AA_00638 | hypothetical protein ARMSODRAFT_953107 [Armillaria solidipes] | 0.273745938 |
| chr09_AA_00241 | SNF2 chromatin remodeling protein [Armillaria solidipes] | chr11_AA_00719 | hypothetical protein ARMGADRAFT_1019784, partial [Armillaria gallica] | 0.227513857 |
| chr09_AA_00241 | SNF2 chromatin remodeling protein [Armillaria solidipes] | chr11_AA_00724 | WD40 repeat-like protein [Armillaria solidipes] | 0.282006284 |
| chr09_AA_00241 | SNF2 chromatin remodeling protein [Armillaria solidipes] | chr11_AA_00790 | uncharacterized protein ARMOST_05401 [Armillaria ostoyae] | 0.102328246 |
| chr09_AA_00241 | SNF2 chromatin remodeling protein [Armillaria solidipes] | chr11_AA_00878 | hypothetical protein CYLTODRAFT_430286 [Cylindrobasidium torrendii FP15055 ss-10] | 0.21950421 |
| chr09_AA_00241 | SNF2 chromatin remodeling protein [Armillaria solidipes] | chr11_AA_00928 | putative malate synthase [Flammulina velutipes] | 0.217086871 |
| chr09_AA_00241 | SNF2 chromatin remodeling protein [Armillaria solidipes] | chr11_AA_00997 | Annexin [Cylindrobasidium torrendii FP15055 ss-10] | 0.191821563 |
| chr09_AA_00241 | SNF2 chromatin remodeling protein [Armillaria solidipes] | chr11_AA_01033 | glycoside hydrolase family 51 protein [Cylindrobasidium torrendii FP15055 ss-10] | 0.274647276 |
| chr09_AA_00241 | SNF2 chromatin remodeling protein [Armillaria solidipes] | chr11_AA_01042 | acetoin reductase family protein [Cylindrobasidium torrendii FP15055 ss-10] | 0.144674166 |
| chr09_AA_00241 | SNF2 chromatin remodeling protein [Armillaria solidipes] | chr11_AA_01046 | hypothetical protein CYLTODRAFT_428160 [Cylindrobasidium torrendii FP15055 ss-10] | 0.173337209 |
| chr09_AA_00241 | SNF2 chromatin remodeling protein [Armillaria solidipes] | chr11_AA_01097 | glycoside hydrolase family 3 protein [Cylindrobasidium torrendii FP15055 ss-10] | 0.124327368 |
| chr09_AA_00241 | SNF2 chromatin remodeling protein [Armillaria solidipes] | chr11_AA_01106 | related to stomatin [Armillaria ostoyae] | 0.145130962 |
| chr09_AA_00241 | SNF2 chromatin remodeling protein [Armillaria solidipes] | chr11_AA_01313 | hypothetical protein Hypma_000372 [Hypsizygus marmoreus] | 0.288087399 |
| chr09_AA_00241 | SNF2 chromatin remodeling protein [Armillaria solidipes] | chr11_AA_01324 | terpenoid synthase [Cylindrobasidium torrendii FP15055 ss-10] | 0.283133156 |
| chr09_AA_00241 | SNF2 chromatin remodeling protein [Armillaria solidipes] | chr11_AA_01456 | uncharacterized protein ARMOST_16541 [Armillaria ostoyae] | 0.197137323 |
| chr09_AA_00241 | SNF2 chromatin remodeling protein [Armillaria solidipes] | chr11_AA_01461 | ricin B-like lectin [Armillaria gallica] | 0.203244371 |
| chr09_AA_00241 | SNF2 chromatin remodeling protein [Armillaria solidipes] | chr11_AA_01636 | hypothetical protein CYLTODRAFT_421880 [Cylindrobasidium torrendii FP15055 ss-10] | 0.184918297 |
| chr09_AA_00241 | SNF2 chromatin remodeling protein [Armillaria solidipes] | chr11_AA_01644 | hypothetical protein CYLTODRAFT_438918 [Cylindrobasidium torrendii FP15055 ss-10] | 0.221249019 |
| chr09_AA_00241 | SNF2 chromatin remodeling protein [Armillaria solidipes] | chr11_AA_01664 | hypothetical protein CYLTODRAFT_421876 [Cylindrobasidium torrendii FP15055 ss-10] | 0.13308971 |
| chr09_AA_00241 | SNF2 chromatin remodeling protein [Armillaria solidipes] | chr11_AA_01685 | 1-aminocyclopropane-1-carboxylate deaminase [Armillaria solidipes] | 0.16424469 |
| chr09_AA_00241 | SNF2 chromatin remodeling protein [Armillaria solidipes] | chr11_AA_01686 | hypothetical protein CYLTODRAFT_348076 [Cylindrobasidium torrendii FP15055 ss-10] | 0.252263937 |
| chr09_AA_00241 | SNF2 chromatin remodeling protein [Armillaria solidipes] | chr11_AA_01711 | hypothetical protein ARMGADRAFT_1046867 [Armillaria gallica] | 0.267416159 |
| chr09_AA_00241 | SNF2 chromatin remodeling protein [Armillaria solidipes] | chr11_AA_01731 | benzoquinone reductase [Cylindrobasidium torrendii FP15055 ss-10] | 0.167947907 |
| chr09_AA_00241 | SNF2 chromatin remodeling protein [Armillaria solidipes] | chr11_AA_01732 | glycoside hydrolase family 76 protein [Cylindrobasidium torrendii FP15055 ss-10] | 0.183483669 |
| chr09_AA_00241 | SNF2 chromatin remodeling protein [Armillaria solidipes] | chr11_AA_01733 | glycoside hydrolase family 76 protein [Cylindrobasidium torrendii FP15055 ss-10] | 0.191609973 |
| chr08_AA_00858 | cytochrome P450 [Cylindrobasidium torrendii FP15055 ss-10] | chr08_AA_00936 | MFS general substrate transporter [Armillaria gallica] | 0.21043837 |
| chr08_AA_00858 | cytochrome P450 [Cylindrobasidium torrendii FP15055 ss-10] | chr11_AA_00724 | WD40 repeat-like protein [Armillaria solidipes] | 0.340182168 |
| chr08_AA_00858 | cytochrome P450 [Cylindrobasidium torrendii FP15055 ss-10] | chr09_AA_00052 | GMC oxidoreductase [Cylindrobasidium torrendii FP15055 ss-10] | 0.317928396 |
| chr08_AA_00858 | cytochrome P450 [Cylindrobasidium torrendii FP15055 ss-10] | chr10_AA_00693 | hypothetical protein ARMSODRAFT_947720 [Armillaria solidipes] | 0.306224709 |
| chr08_AA_00858 | cytochrome P450 [Cylindrobasidium torrendii FP15055 ss-10] | chr10_AA_01664 | hypothetical protein CYLTODRAFT_404266 [Cylindrobasidium torrendii FP15055 ss-10] | 0.306177205 |
| chr08_AA_00858 | cytochrome P450 [Cylindrobasidium torrendii FP15055 ss-10] | chr10_AA_01157 | glycoside hydrolase family 53 protein [Cylindrobasidium torrendii FP15055 ss-10] | 0.299414708 |
| chr08_AA_00858 | cytochrome P450 [Cylindrobasidium torrendii FP15055 ss-10] | chr10_AA_01341 | glucuronyl hydrolase [Armillaria solidipes] | 0.294525843 |
| chr08_AA_00858 | cytochrome P450 [Cylindrobasidium torrendii FP15055 ss-10] | chr10_AA_01143 | uncharacterized protein ARMOST_08409 [Armillaria ostoyae] | 0.294071292 |
| chr08_AA_00858 | cytochrome P450 [Cylindrobasidium torrendii FP15055 ss-10] | chr09_AA_00051 | GMC oxidoreductase [Cylindrobasidium torrendii FP15055 ss-10] | 0.291836496 |
| chr08_AA_00858 | cytochrome P450 [Cylindrobasidium torrendii FP15055 ss-10] | chr10_AA_00651 | hypothetical protein ARMGADRAFT_897215, partial [Armillaria gallica] | 0.285601074 |
| chr08_AA_00858 | cytochrome P450 [Cylindrobasidium torrendii FP15055 ss-10] | chr11_AA_00042 | kinase-like protein [Cylindrobasidium torrendii FP15055 ss-10] | 0.28475182 |
| chr08_AA_00858 | cytochrome P450 [Cylindrobasidium torrendii FP15055 ss-10] | chr10_AA_00662 | Ureohydrolase [Armillaria gallica] | 0.28341031 |
| chr08_AA_00858 | cytochrome P450 [Cylindrobasidium torrendii FP15055 ss-10] | chr10_AA_00499 | hypothetical protein WG66_16220 [Moniliophthora roreri] | 0.283253571 |
| chr08_AA_00858 | cytochrome P450 [Cylindrobasidium torrendii FP15055 ss-10] | chr09_AA_01103 | Metallo-dependent phosphatase [Armillaria solidipes] | 0.27909326 |
| chr08_AA_00858 | cytochrome P450 [Cylindrobasidium torrendii FP15055 ss-10] | chr11_AA_01711 | hypothetical protein ARMGADRAFT_1046867 [Armillaria gallica] | 0.273412527 |
| chr08_AA_00858 | cytochrome P450 [Cylindrobasidium torrendii FP15055 ss-10] | chr11_AA_00504 | MFS general substrate transporter [Cylindrobasidium torrendii FP15055 ss-10] | 0.2688623 |
| chr08_AA_00858 | cytochrome P450 [Cylindrobasidium torrendii FP15055 ss-10] | chr10_AA_00441 | alpha/beta-hydrolase [Armillaria solidipes] | 0.268184998 |
| chr08_AA_00858 | cytochrome P450 [Cylindrobasidium torrendii FP15055 ss-10] | chr08_AA_01091 | uncharacterized protein ARMOST_03386 [Armillaria ostoyae] | 0.26791624 |
| chr08_AA_00858 | cytochrome P450 [Cylindrobasidium torrendii FP15055 ss-10] | chr11_AA_01636 | hypothetical protein CYLTODRAFT_421880 [Cylindrobasidium torrendii FP15055 ss-10] | 0.257437199 |
| chr08_AA_00858 | cytochrome P450 [Cylindrobasidium torrendii FP15055 ss-10] | chr09_AA_01214 | hypothetical protein CYLTODRAFT_417942 [Cylindrobasidium torrendii FP15055 ss-10] | 0.255745707 |
| chr08_AA_00858 | cytochrome P450 [Cylindrobasidium torrendii FP15055 ss-10] | chr11_AA_00928 | putative malate synthase [Flammulina velutipes] | 0.25466938 |
| chr08_AA_00858 | cytochrome P450 [Cylindrobasidium torrendii FP15055 ss-10] | chr11_AA_01033 | glycoside hydrolase family 51 protein [Cylindrobasidium torrendii FP15055 ss-10] | 0.254242245 |
| chr08_AA_00858 | cytochrome P450 [Cylindrobasidium torrendii FP15055 ss-10] | chr11_AA_01686 | hypothetical protein CYLTODRAFT_348076 [Cylindrobasidium torrendii FP15055 ss-10] | 0.254059109 |
| chr08_AA_00858 | cytochrome P450 [Cylindrobasidium torrendii FP15055 ss-10] | chr08_AA_01257 | hypothetical protein ARMSODRAFT_949486 [Armillaria solidipes] | 0.253167168 |
| chr08_AA_00858 | cytochrome P450 [Cylindrobasidium torrendii FP15055 ss-10] | chr08_AA_01027 | hypothetical protein CYLTODRAFT_357890 [Cylindrobasidium torrendii FP15055 ss-10] | 0.25284542 |
| chr08_AA_00858 | cytochrome P450 [Cylindrobasidium torrendii FP15055 ss-10] | chr10_AA_00164 | dehydrogenase E1 and transketolase domain-containing protein 1 [Cylindrobasidium torrendii FP15055 ss-10] | 0.250445244 |
| chr08_AA_00858 | cytochrome P450 [Cylindrobasidium torrendii FP15055 ss-10] | chr08_AA_01097 | hypothetical protein GYMLUDRAFT_32937 [Gymnopus luxurians FD-317 M1] | 0.249465583 |
| chr08_AA_00858 | cytochrome P450 [Cylindrobasidium torrendii FP15055 ss-10] | chr09_AA_00719 | hypothetical protein PLEOSDRAFT_1093927 [Pleurotus ostreatus PC15] | 0.248533639 |
| chr08_AA_00858 | cytochrome P450 [Cylindrobasidium torrendii FP15055 ss-10] | chr10_AA_01231 | hypothetical protein ARMSODRAFT_1019532 [Armillaria solidipes] | 0.247023897 |
| chr08_AA_00858 | cytochrome P450 [Cylindrobasidium torrendii FP15055 ss-10] | chr09_AA_00036 | hypothetical protein ARMGADRAFT_1070289 [Armillaria gallica] | 0.246924369 |
| chr08_AA_00858 | cytochrome P450 [Cylindrobasidium torrendii FP15055 ss-10] | chr11_AA_00499 | GroES-like protein [Armillaria solidipes] | 0.246358016 |
| chr08_AA_00858 | cytochrome P450 [Cylindrobasidium torrendii FP15055 ss-10] | chr08_AA_01155 | related to 3-oxoacyl CoA thiolase [Armillaria ostoyae] | 0.245145253 |
| chr08_AA_00858 | cytochrome P450 [Cylindrobasidium torrendii FP15055 ss-10] | chr11_AA_00606 | hypothetical protein CYLTODRAFT_418833 [Cylindrobasidium torrendii FP15055 ss-10] | 0.244269671 |
| chr08_AA_00858 | cytochrome P450 [Cylindrobasidium torrendii FP15055 ss-10] | chr11_AA_00878 | hypothetical protein CYLTODRAFT_430286 [Cylindrobasidium torrendii FP15055 ss-10] | 0.24189433 |
| chr08_AA_00858 | cytochrome P450 [Cylindrobasidium torrendii FP15055 ss-10] | chr11_AA_01313 | hypothetical protein Hypma_000372 [Hypsizygus marmoreus] | 0.240134019 |
| chr08_AA_00858 | cytochrome P450 [Cylindrobasidium torrendii FP15055 ss-10] | chr09_AA_00241 | SNF2 chromatin remodeling protein [Armillaria solidipes] | 0.23977207 |
| chr08_AA_00858 | cytochrome P450 [Cylindrobasidium torrendii FP15055 ss-10] | chr09_AA_00892 | hypothetical protein CYLTODRAFT_431446 [Cylindrobasidium torrendii FP15055 ss-10] | 0.239184473 |
| chr08_AA_00858 | cytochrome P450 [Cylindrobasidium torrendii FP15055 ss-10] | chr09_AA_01190 | potassium/sodium eff [Cylindrobasidium torrendii FP15055 ss-10] | 0.238770299 |
| chr08_AA_00858 | cytochrome P450 [Cylindrobasidium torrendii FP15055 ss-10] | chr10_AA_00944 | general substrate transporter [Cylindrobasidium torrendii FP15055 ss-10] | 0.236533687 |
| chr08_AA_00858 | cytochrome P450 [Cylindrobasidium torrendii FP15055 ss-10] | chr11_AA_00103 | hypothetical protein M413DRAFT_370146 [Hebeloma cylindrosporum h7] | 0.236322315 |
| chr08_AA_00858 | cytochrome P450 [Cylindrobasidium torrendii FP15055 ss-10] | chr08_AA_01259 | Aldo/keto reductase [Armillaria gallica] | 0.235312267 |
| chr08_AA_00858 | cytochrome P450 [Cylindrobasidium torrendii FP15055 ss-10] | chr08_AA_01046 | MFS general substrate transporter [Armillaria gallica] | 0.235223805 |
| chr08_AA_00858 | cytochrome P450 [Cylindrobasidium torrendii FP15055 ss-10] | chr09_AA_00838 | cytochrome P450 [Armillaria gallica] | 0.231789901 |
| chr08_AA_00858 | cytochrome P450 [Cylindrobasidium torrendii FP15055 ss-10] | chr10_AA_00220 | hypothetical protein ARMGADRAFT_962646, partial [Armillaria gallica] | 0.229868064 |
| chr08_AA_00858 | cytochrome P450 [Cylindrobasidium torrendii FP15055 ss-10] | chr11_AA_01046 | hypothetical protein CYLTODRAFT_428160 [Cylindrobasidium torrendii FP15055 ss-10] | 0.229112122 |
| chr08_AA_00858 | cytochrome P450 [Cylindrobasidium torrendii FP15055 ss-10] | chr09_AA_00162 | hypothetical protein ARMGADRAFT_961910 [Armillaria gallica] | 0.229044133 |
| chr08_AA_00858 | cytochrome P450 [Cylindrobasidium torrendii FP15055 ss-10] | chr11_AA_00265 | FAD-linked oxidoreductase [Armillaria solidipes] | 0.224412835 |
| chr08_AA_00858 | cytochrome P450 [Cylindrobasidium torrendii FP15055 ss-10] | chr10_AA_00270 | branched-chain alpha-keto acid dehydrogenase E1-alpha subunit [Cylindrobasidium torrendii FP15055 ss-10] | 0.223759328 |
| chr08_AA_00858 | cytochrome P450 [Cylindrobasidium torrendii FP15055 ss-10] | chr09_AA_00191 | exo-beta-1,3-glucanase [Armillaria solidipes] | 0.222389911 |
| chr08_AA_00858 | cytochrome P450 [Cylindrobasidium torrendii FP15055 ss-10] | chr09_AA_00190 | putative exo-beta-1,3-glucanase [Flammulina velutipes] | 0.221767627 |
| chr08_AA_00858 | cytochrome P450 [Cylindrobasidium torrendii FP15055 ss-10] | chr11_AA_01733 | glycoside hydrolase family 76 protein [Cylindrobasidium torrendii FP15055 ss-10] | 0.219017694 |
| chr08_AA_00858 | cytochrome P450 [Cylindrobasidium torrendii FP15055 ss-10] | chr09_AA_01099 | general substrate transporter [Armillaria gallica] | 0.21765669 |
| chr08_AA_00858 | cytochrome P450 [Cylindrobasidium torrendii FP15055 ss-10] | chr08_AA_00984 | cytochrome P450 monooxygenase pc-bph [Armillaria gallica] | 0.217635949 |
| chr08_AA_00858 | cytochrome P450 [Cylindrobasidium torrendii FP15055 ss-10] | chr09_AA_00518 | AAA-domain-containing protein [Cylindrobasidium torrendii FP15055 ss-10] | 0.215259194 |
| chr08_AA_00858 | cytochrome P450 [Cylindrobasidium torrendii FP15055 ss-10] | chr08_AA_00948 | probable glutamate dehydrogenase, NAD(+)-specific [Armillaria ostoyae] | 0.213047722 |
| chr08_AA_00858 | cytochrome P450 [Cylindrobasidium torrendii FP15055 ss-10] | chr11_AA_01097 | glycoside hydrolase family 3 protein [Cylindrobasidium torrendii FP15055 ss-10] | 0.213041739 |
| chr08_AA_00858 | cytochrome P450 [Cylindrobasidium torrendii FP15055 ss-10] | chr11_AA_01664 | hypothetical protein CYLTODRAFT_421876 [Cylindrobasidium torrendii FP15055 ss-10] | 0.211400741 |
| chr08_AA_00858 | cytochrome P450 [Cylindrobasidium torrendii FP15055 ss-10] | chr11_AA_01732 | glycoside hydrolase family 76 protein [Cylindrobasidium torrendii FP15055 ss-10] | 0.210755279 |
| chr08_AA_00858 | cytochrome P450 [Cylindrobasidium torrendii FP15055 ss-10] | chr09_AA_01058 | hypothetical protein ARMSODRAFT_976614 [Armillaria solidipes] | 0.210650085 |
| chr08_AA_00858 | cytochrome P450 [Cylindrobasidium torrendii FP15055 ss-10] | chr09_AA_00680 | hypothetical protein CYLTODRAFT_395198 [Cylindrobasidium torrendii FP15055 ss-10] | 0.210380218 |
| chr08_AA_00858 | cytochrome P450 [Cylindrobasidium torrendii FP15055 ss-10] | chr11_AA_00997 | Annexin [Cylindrobasidium torrendii FP15055 ss-10] | 0.209410425 |
| chr08_AA_00858 | cytochrome P450 [Cylindrobasidium torrendii FP15055 ss-10] | chr09_AA_00229 | succinyl-CoA:3-ketoacid-coenzyme A transferase [Cylindrobasidium torrendii FP15055 ss-10] | 0.204838015 |
| chr08_AA_00858 | cytochrome P450 [Cylindrobasidium torrendii FP15055 ss-10] | chr09_AA_01224 | uncharacterized protein ARMOST_22339 [Armillaria ostoyae] | 0.20275113 |
| chr08_AA_00858 | cytochrome P450 [Cylindrobasidium torrendii FP15055 ss-10] | chr09_AA_00095 | hypothetical protein CYLTODRAFT_422329 [Cylindrobasidium torrendii FP15055 ss-10] | 0.199988167 |
| chr08_AA_00858 | cytochrome P450 [Cylindrobasidium torrendii FP15055 ss-10] | chr10_AA_01201 | acyltransferase ChoActase/COT/CPT [Armillaria gallica] | 0.199619513 |
| chr08_AA_00858 | cytochrome P450 [Cylindrobasidium torrendii FP15055 ss-10] | chr11_AA_01456 | uncharacterized protein ARMOST_16541 [Armillaria ostoyae] | 0.199130379 |
| chr08_AA_00858 | cytochrome P450 [Cylindrobasidium torrendii FP15055 ss-10] | chr09_AA_01106 | multidrug transporter [Cylindrobasidium torrendii FP15055 ss-10] | 0.197764908 |
| chr08_AA_00858 | cytochrome P450 [Cylindrobasidium torrendii FP15055 ss-10] | chr10_AA_01063 | glycoside hydrolase family 43 protein [Amanita thiersii Skay4041] | 0.196534132 |
| chr08_AA_00858 | cytochrome P450 [Cylindrobasidium torrendii FP15055 ss-10] | chr10_AA_01576 | D-arabinitol 2-dehydrogenase [ribulose-forming] [Hypsizygus marmoreus] | 0.191553616 |
| chr08_AA_00858 | cytochrome P450 [Cylindrobasidium torrendii FP15055 ss-10] | chr09_AA_00406 | uncharacterized protein ARMOST_17180 [Armillaria ostoyae] | 0.189432609 |
| chr08_AA_00858 | cytochrome P450 [Cylindrobasidium torrendii FP15055 ss-10] | chr11_AA_01106 | related to stomatin [Armillaria ostoyae] | 0.187645776 |
| chr08_AA_00858 | cytochrome P450 [Cylindrobasidium torrendii FP15055 ss-10] | chr10_AA_00517 | hypothetical protein CYLTODRAFT_256211 [Cylindrobasidium torrendii FP15055 ss-10] | 0.186631222 |
| chr08_AA_00858 | cytochrome P450 [Cylindrobasidium torrendii FP15055 ss-10] | chr10_AA_00134 | hypothetical protein CYLTODRAFT_349864 [Cylindrobasidium torrendii FP15055 ss-10] | 0.18222519 |
| chr08_AA_00858 | cytochrome P450 [Cylindrobasidium torrendii FP15055 ss-10] | chr08_AA_01069 | alpha/beta-hydrolase [Armillaria solidipes] | 0.181470653 |
| chr08_AA_00858 | cytochrome P450 [Cylindrobasidium torrendii FP15055 ss-10] | chr10_AA_01213 | hypothetical protein JAAARDRAFT_210932 [Jaapia argillacea MUCL 33604] | 0.176489194 |
| chr08_AA_00858 | cytochrome P450 [Cylindrobasidium torrendii FP15055 ss-10] | chr11_AA_00790 | uncharacterized protein ARMOST_05401 [Armillaria ostoyae] | 0.175414261 |
| chr08_AA_00858 | cytochrome P450 [Cylindrobasidium torrendii FP15055 ss-10] | chr09_AA_00100 | hypothetical protein K503DRAFT_238266 [Rhizopogon vinicolor AM-OR11-026] | 0.171885372 |
| chr08_AA_00858 | cytochrome P450 [Cylindrobasidium torrendii FP15055 ss-10] | chr09_AA_01141 | hypothetical protein ARMGADRAFT_1063089 [Armillaria gallica] | 0.169785364 |
| chr08_AA_00858 | cytochrome P450 [Cylindrobasidium torrendii FP15055 ss-10] | chr10_AA_00733 | Zinc-regulated transporter 1 [Hypsizygus marmoreus] | 0.169706665 |
| chr08_AA_00858 | cytochrome P450 [Cylindrobasidium torrendii FP15055 ss-10] | chr08_AA_01172 | carbohydrate esterase family 1 protein, partial [Cylindrobasidium torrendii FP15055 ss-10] | 0.164913998 |
| chr08_AA_00858 | cytochrome P450 [Cylindrobasidium torrendii FP15055 ss-10] | chr11_AA_01042 | acetoin reductase family protein [Cylindrobasidium torrendii FP15055 ss-10] | 0.162874205 |
| chr08_AA_00858 | cytochrome P450 [Cylindrobasidium torrendii FP15055 ss-10] | chr10_AA_00256 | uncharacterized protein ARMOST_03672 [Armillaria ostoyae] | 0.16260099 |
| chr08_AA_00858 | cytochrome P450 [Cylindrobasidium torrendii FP15055 ss-10] | chr09_AA_00762 | transketolase [Armillaria solidipes] | 0.162089641 |
| chr08_AA_00858 | cytochrome P450 [Cylindrobasidium torrendii FP15055 ss-10] | chr08_AA_01176 | hypothetical protein HYPSUDRAFT_61393 [Hypholoma sublateritium FD-334 SS-4] | 0.161649521 |
| chr08_AA_00858 | cytochrome P450 [Cylindrobasidium torrendii FP15055 ss-10] | chr11_AA_00094 | homogentisate 1,2-dioxygenase [Armillaria solidipes] | 0.15897855 |
| chr08_AA_00858 | cytochrome P450 [Cylindrobasidium torrendii FP15055 ss-10] | chr11_AA_00145 | hypothetical protein CYLTODRAFT_243491 [Cylindrobasidium torrendii FP15055 ss-10] | 0.157068575 |
| chr08_AA_00858 | cytochrome P450 [Cylindrobasidium torrendii FP15055 ss-10] | chr11_AA_01324 | terpenoid synthase [Cylindrobasidium torrendii FP15055 ss-10] | 0.15591916 |
| chr08_AA_00858 | cytochrome P450 [Cylindrobasidium torrendii FP15055 ss-10] | chr11_AA_01461 | ricin B-like lectin [Armillaria gallica] | 0.155149248 |
| chr08_AA_00858 | cytochrome P450 [Cylindrobasidium torrendii FP15055 ss-10] | chr10_AA_00735 | glycoside hydrolase family 31 protein [Cylindrobasidium torrendii FP15055 ss-10] | 0.152032207 |
| chr08_AA_00858 | cytochrome P450 [Cylindrobasidium torrendii FP15055 ss-10] | chr09_AA_00410 | hypothetical protein AMATHDRAFT_73341 [Amanita thiersii Skay4041] | 0.147996529 |
| chr08_AA_00858 | cytochrome P450 [Cylindrobasidium torrendii FP15055 ss-10] | chr11_AA_00638 | hypothetical protein ARMSODRAFT_953107 [Armillaria solidipes] | 0.147571637 |
| chr08_AA_00858 | cytochrome P450 [Cylindrobasidium torrendii FP15055 ss-10] | chr11_AA_01685 | 1-aminocyclopropane-1-carboxylate deaminase [Armillaria solidipes] | 0.1459427 |
| chr08_AA_00858 | cytochrome P450 [Cylindrobasidium torrendii FP15055 ss-10] | chr08_AA_01081 | uncharacterized protein ARMOST_20173 [Armillaria ostoyae] | 0.145390208 |
| chr08_AA_00858 | cytochrome P450 [Cylindrobasidium torrendii FP15055 ss-10] | chr11_AA_00233 | hypothetical protein ARMGADRAFT_977028 [Armillaria gallica] | 0.144757508 |
| chr08_AA_00858 | cytochrome P450 [Cylindrobasidium torrendii FP15055 ss-10] | chr09_AA_00955 | putative endo-1,4-beta-xylanase precursor [Flammulina velutipes] | 0.141443458 |
| chr08_AA_00858 | cytochrome P450 [Cylindrobasidium torrendii FP15055 ss-10] | chr09_AA_00816 | mandelate racemase muconate lactonizing enzyme family protein [Cylindrobasidium torrendii FP15055 ss-10] | 0.141186481 |
| chr08_AA_00858 | cytochrome P450 [Cylindrobasidium torrendii FP15055 ss-10] | chr11_AA_00701 | uncharacterized protein ARMOST_05344 [Armillaria ostoyae] | 0.139772686 |
| chr08_AA_00858 | cytochrome P450 [Cylindrobasidium torrendii FP15055 ss-10] | chr09_AA_00312 | putative beta-glucosidase [Flammulina velutipes] | 0.12746722 |
| chr08_AA_00858 | cytochrome P450 [Cylindrobasidium torrendii FP15055 ss-10] | chr08_AA_01077 | GroES-like protein [Cylindrobasidium torrendii FP15055 ss-10] | 0.126925946 |
| chr08_AA_00858 | cytochrome P450 [Cylindrobasidium torrendii FP15055 ss-10] | chr11_AA_01510 | hypothetical protein ARMGADRAFT_1015707 [Armillaria gallica] | 0.126898217 |
| chr08_AA_00858 | cytochrome P450 [Cylindrobasidium torrendii FP15055 ss-10] | chr10_AA_01309 | hypothetical protein PHLCEN_2v2282 [Phlebia centrifuga] | 0.123869637 |
| chr08_AA_00858 | cytochrome P450 [Cylindrobasidium torrendii FP15055 ss-10] | chr09_AA_01410 | hypothetical protein CYLTODRAFT_489277 [Cylindrobasidium torrendii FP15055 ss-10] | 0.120307187 |
| chr08_AA_00858 | cytochrome P450 [Cylindrobasidium torrendii FP15055 ss-10] | chr11_AA_00719 | hypothetical protein ARMGADRAFT_1019784, partial [Armillaria gallica] | 0.115065345 |
| chr08_AA_00858 | cytochrome P450 [Cylindrobasidium torrendii FP15055 ss-10] | chr11_AA_00277 | uncharacterized protein ARMOST_10602 [Armillaria ostoyae] | 0.113426308 |
| chr08_AA_00858 | cytochrome P450 [Cylindrobasidium torrendii FP15055 ss-10] | chr11_AA_01644 | hypothetical protein CYLTODRAFT_438918 [Cylindrobasidium torrendii FP15055 ss-10] | 0.111186817 |
| chr08_AA_00858 | cytochrome P450 [Cylindrobasidium torrendii FP15055 ss-10] | chr10_AA_01056 | hypothetical protein ARMGADRAFT_1020988 [Armillaria gallica] | 0.108885879 |
| chr08_AA_00858 | cytochrome P450 [Cylindrobasidium torrendii FP15055 ss-10] | chr09_AA_00023 | hypothetical protein WG66_107 [Moniliophthora roreri] | 0.107791527 |
| chr11_AA_00724 | WD40 repeat-like protein [Armillaria solidipes] | chr11_AA_00790 | uncharacterized protein ARMOST_05401 [Armillaria ostoyae] | 0.270684903 |
| chr11_AA_00724 | WD40 repeat-like protein [Armillaria solidipes] | chr11_AA_00878 | hypothetical protein CYLTODRAFT_430286 [Cylindrobasidium torrendii FP15055 ss-10] | 0.332888215 |
| chr11_AA_00724 | WD40 repeat-like protein [Armillaria solidipes] | chr11_AA_00928 | putative malate synthase [Flammulina velutipes] | 0.344057784 |
| chr11_AA_00724 | WD40 repeat-like protein [Armillaria solidipes] | chr11_AA_00997 | Annexin [Cylindrobasidium torrendii FP15055 ss-10] | 0.283242262 |
| chr11_AA_00724 | WD40 repeat-like protein [Armillaria solidipes] | chr11_AA_01033 | glycoside hydrolase family 51 protein [Cylindrobasidium torrendii FP15055 ss-10] | 0.313680276 |
| chr11_AA_00724 | WD40 repeat-like protein [Armillaria solidipes] | chr11_AA_01042 | acetoin reductase family protein [Cylindrobasidium torrendii FP15055 ss-10] | 0.226872824 |
| chr11_AA_00724 | WD40 repeat-like protein [Armillaria solidipes] | chr11_AA_01046 | hypothetical protein CYLTODRAFT_428160 [Cylindrobasidium torrendii FP15055 ss-10] | 0.310511005 |
| chr11_AA_00724 | WD40 repeat-like protein [Armillaria solidipes] | chr11_AA_01097 | glycoside hydrolase family 3 protein [Cylindrobasidium torrendii FP15055 ss-10] | 0.285135179 |
| chr11_AA_00724 | WD40 repeat-like protein [Armillaria solidipes] | chr11_AA_01106 | related to stomatin [Armillaria ostoyae] | 0.258964522 |
| chr11_AA_00724 | WD40 repeat-like protein [Armillaria solidipes] | chr11_AA_01313 | hypothetical protein Hypma_000372 [Hypsizygus marmoreus] | 0.272752882 |
| chr11_AA_00724 | WD40 repeat-like protein [Armillaria solidipes] | chr11_AA_01324 | terpenoid synthase [Cylindrobasidium torrendii FP15055 ss-10] | 0.20123578 |
| chr11_AA_00724 | WD40 repeat-like protein [Armillaria solidipes] | chr11_AA_01445 | hypothetical protein ARMSODRAFT_950555 [Armillaria solidipes] &gt;SJL10115.1 uncharacterized protein ARMOST_13499 [Armillaria ostoyae] | 0.135115861 |
| chr11_AA_00724 | WD40 repeat-like protein [Armillaria solidipes] | chr11_AA_01456 | uncharacterized protein ARMOST_16541 [Armillaria ostoyae] | 0.260607794 |
| chr11_AA_00724 | WD40 repeat-like protein [Armillaria solidipes] | chr11_AA_01461 | ricin B-like lectin [Armillaria gallica] | 0.278345718 |
| chr11_AA_00724 | WD40 repeat-like protein [Armillaria solidipes] | chr11_AA_01510 | hypothetical protein ARMGADRAFT_1015707 [Armillaria gallica] | 0.232335573 |
| chr11_AA_00724 | WD40 repeat-like protein [Armillaria solidipes] | chr11_AA_01636 | hypothetical protein CYLTODRAFT_421880 [Cylindrobasidium torrendii FP15055 ss-10] | 0.303389876 |
| chr11_AA_00724 | WD40 repeat-like protein [Armillaria solidipes] | chr11_AA_01644 | hypothetical protein CYLTODRAFT_438918 [Cylindrobasidium torrendii FP15055 ss-10] | 0.164698013 |
| chr11_AA_00724 | WD40 repeat-like protein [Armillaria solidipes] | chr11_AA_01664 | hypothetical protein CYLTODRAFT_421876 [Cylindrobasidium torrendii FP15055 ss-10] | 0.298499354 |
| chr11_AA_00724 | WD40 repeat-like protein [Armillaria solidipes] | chr11_AA_01685 | 1-aminocyclopropane-1-carboxylate deaminase [Armillaria solidipes] | 0.254656501 |
| chr11_AA_00724 | WD40 repeat-like protein [Armillaria solidipes] | chr11_AA_01686 | hypothetical protein CYLTODRAFT_348076 [Cylindrobasidium torrendii FP15055 ss-10] | 0.209496004 |
| chr11_AA_00724 | WD40 repeat-like protein [Armillaria solidipes] | chr11_AA_01711 | hypothetical protein ARMGADRAFT_1046867 [Armillaria gallica] | 0.325436555 |
| chr11_AA_00724 | WD40 repeat-like protein [Armillaria solidipes] | chr11_AA_01731 | benzoquinone reductase [Cylindrobasidium torrendii FP15055 ss-10] | 0.111774512 |
| chr11_AA_00724 | WD40 repeat-like protein [Armillaria solidipes] | chr11_AA_01732 | glycoside hydrolase family 76 protein [Cylindrobasidium torrendii FP15055 ss-10] | 0.282744751 |
| chr11_AA_00724 | WD40 repeat-like protein [Armillaria solidipes] | chr11_AA_01733 | glycoside hydrolase family 76 protein [Cylindrobasidium torrendii FP15055 ss-10] | 0.285986348 |
| chr02_AA_00171 | hypothetical protein CYLTODRAFT_425697 [Cylindrobasidium torrendii FP15055 ss-10] | chr02_AA_00341 | oxidoreductase [Armillaria solidipes] | 0.227488956 |
| chr02_AA_00171 | hypothetical protein CYLTODRAFT_425697 [Cylindrobasidium torrendii FP15055 ss-10] | chr02_AA_00393 | related to GRE2-methylglyoxal reductase (NADPH-dependent) [Armillaria ostoyae] | 0.129042502 |
| chr02_AA_00171 | hypothetical protein CYLTODRAFT_425697 [Cylindrobasidium torrendii FP15055 ss-10] | chr03_AA_00240 | hypothetical protein CYLTODRAFT_426591 [Cylindrobasidium torrendii FP15055 ss-10] | 0.166362065 |
| chr02_AA_00171 | hypothetical protein CYLTODRAFT_425697 [Cylindrobasidium torrendii FP15055 ss-10] | chr03_AA_00291 | hypothetical protein ARMGADRAFT_1170747 [Armillaria gallica] | 0.188602515 |
| chr02_AA_00171 | hypothetical protein CYLTODRAFT_425697 [Cylindrobasidium torrendii FP15055 ss-10] | chr03_AA_00294 | hypothetical protein CYLTODRAFT_489528 [Cylindrobasidium torrendii FP15055 ss-10] | 0.18377361 |
| chr02_AA_00171 | hypothetical protein CYLTODRAFT_425697 [Cylindrobasidium torrendii FP15055 ss-10] | chr03_AA_00298 | hypothetical protein CYLTODRAFT_458738 [Cylindrobasidium torrendii FP15055 ss-10] | 0.158504596 |
| chr02_AA_00171 | hypothetical protein CYLTODRAFT_425697 [Cylindrobasidium torrendii FP15055 ss-10] | chr03_AA_00325 | hypothetical protein CYLTODRAFT_421760 [Cylindrobasidium torrendii FP15055 ss-10] | 0.298118065 |
| chr02_AA_00171 | hypothetical protein CYLTODRAFT_425697 [Cylindrobasidium torrendii FP15055 ss-10] | chr03_AA_00403 | hypothetical protein CYLTODRAFT_424860 [Cylindrobasidium torrendii FP15055 ss-10] | 0.184420742 |
| chr02_AA_00171 | hypothetical protein CYLTODRAFT_425697 [Cylindrobasidium torrendii FP15055 ss-10] | chr04_AA_00052 | hypothetical protein CYLTODRAFT_421165 [Cylindrobasidium torrendii FP15055 ss-10] | 0.151641369 |
| chr02_AA_00171 | hypothetical protein CYLTODRAFT_425697 [Cylindrobasidium torrendii FP15055 ss-10] | chr04_AA_00456 | hypothetical protein CYLTODRAFT_376171 [Cylindrobasidium torrendii FP15055 ss-10] | 0.158926107 |
| chr02_AA_00171 | hypothetical protein CYLTODRAFT_425697 [Cylindrobasidium torrendii FP15055 ss-10] | chr04_AA_00470 | hypothetical protein PLICRDRAFT_179967 [Plicaturopsis crispa FD-325 SS-3] | 0.113235539 |
| chr02_AA_00171 | hypothetical protein CYLTODRAFT_425697 [Cylindrobasidium torrendii FP15055 ss-10] | chr04_AA_00484 | hypothetical protein CYLTODRAFT_422976 [Cylindrobasidium torrendii FP15055 ss-10] | 0.239186616 |
| chr02_AA_00171 | hypothetical protein CYLTODRAFT_425697 [Cylindrobasidium torrendii FP15055 ss-10] | chr04_AA_00685 | hypothetical protein CYLTODRAFT_369578 [Cylindrobasidium torrendii FP15055 ss-10] | 0.166438635 |
| chr02_AA_00171 | hypothetical protein CYLTODRAFT_425697 [Cylindrobasidium torrendii FP15055 ss-10] | chr05_AA_00056 | glycoside hydrolase family 20 protein [Cylindrobasidium torrendii FP15055 ss-10] | 0.21488546 |
| chr02_AA_00171 | hypothetical protein CYLTODRAFT_425697 [Cylindrobasidium torrendii FP15055 ss-10] | chr05_AA_00082 | glycoside hydrolase family 20 protein [Cylindrobasidium torrendii FP15055 ss-10] | 0.235014473 |
| chr02_AA_00171 | hypothetical protein CYLTODRAFT_425697 [Cylindrobasidium torrendii FP15055 ss-10] | chr05_AA_00112 | hypothetical protein STEHIDRAFT_155150 [Stereum hirsutum FP-91666 SS1] &gt;EIM87778.1 hypothetical protein STEHIDRAFT_155150 [Stereum hirsutum FP-91666 SS1] | 0.151699156 |
| chr02_AA_00171 | hypothetical protein CYLTODRAFT_425697 [Cylindrobasidium torrendii FP15055 ss-10] | chr05_AA_00118 | glycoside hydrolase family 37 protein [Cylindrobasidium torrendii FP15055 ss-10] | 0.211672827 |
| chr02_AA_00171 | hypothetical protein CYLTODRAFT_425697 [Cylindrobasidium torrendii FP15055 ss-10] | chr05_AA_00317 | hypothetical protein CYLTODRAFT_450559 [Cylindrobasidium torrendii FP15055 ss-10] | 0.271233467 |
| chr02_AA_00171 | hypothetical protein CYLTODRAFT_425697 [Cylindrobasidium torrendii FP15055 ss-10] | chr05_AA_00370 | hypothetical protein GALMADRAFT_90776 [Galerina marginata CBS 339.88] | 0.200139656 |
| chr02_AA_00171 | hypothetical protein CYLTODRAFT_425697 [Cylindrobasidium torrendii FP15055 ss-10] | chr05_AA_00421 | ribosomal protein S5 domain 2-like protein [Cylindrobasidium torrendii FP15055 ss-10] | 0.130248022 |
| chr02_AA_00171 | hypothetical protein CYLTODRAFT_425697 [Cylindrobasidium torrendii FP15055 ss-10] | chr05_AA_00473 | hypothetical protein CYLTODRAFT_449880 [Cylindrobasidium torrendii FP15055 ss-10] | 0.170973203 |
| chr02_AA_00171 | hypothetical protein CYLTODRAFT_425697 [Cylindrobasidium torrendii FP15055 ss-10] | chr05_AA_00481 | hypothetical protein CYLTODRAFT_424727 [Cylindrobasidium torrendii FP15055 ss-10] | 0.241768835 |
| chr02_AA_00171 | hypothetical protein CYLTODRAFT_425697 [Cylindrobasidium torrendii FP15055 ss-10] | chr05_AA_00527 | hypothetical protein NEOLEDRAFT_1026610, partial [Neolentinus lepideus HHB14362 ss-1] | 0.151011482 |
| chr02_AA_00171 | hypothetical protein CYLTODRAFT_425697 [Cylindrobasidium torrendii FP15055 ss-10] | chr05_AA_00599 | glycoside hydrolase family 2 protein [Cylindrobasidium torrendii FP15055 ss-10] | 0.192400236 |
| chr02_AA_00171 | hypothetical protein CYLTODRAFT_425697 [Cylindrobasidium torrendii FP15055 ss-10] | chr05_AA_00607 | hypothetical protein CYLTODRAFT_458172 [Cylindrobasidium torrendii FP15055 ss-10] | 0.117367438 |
| chr02_AA_00171 | hypothetical protein CYLTODRAFT_425697 [Cylindrobasidium torrendii FP15055 ss-10] | chr05_AA_00686 | glycoside hydrolase family 95 protein [Cylindrobasidium torrendii FP15055 ss-10] | 0.27052172 |
| chr02_AA_00171 | hypothetical protein CYLTODRAFT_425697 [Cylindrobasidium torrendii FP15055 ss-10] | chr05_AA_00812 | acid protease [Armillaria gallica] | 0.124957577 |
| chr02_AA_00171 | hypothetical protein CYLTODRAFT_425697 [Cylindrobasidium torrendii FP15055 ss-10] | chr05_AA_00845 | general substrate transporter [Armillaria gallica] | 0.181364293 |
| chr02_AA_00171 | hypothetical protein CYLTODRAFT_425697 [Cylindrobasidium torrendii FP15055 ss-10] | chr05_AA_00860 | hypothetical protein ARMGADRAFT_633196 [Armillaria gallica] | 0.269147301 |
| chr02_AA_00171 | hypothetical protein CYLTODRAFT_425697 [Cylindrobasidium torrendii FP15055 ss-10] | chr06_AA_00237 | acyl-CoA dehydrogenase NM domain-like protein, partial [Armillaria solidipes] | 0.291923222 |
| chr02_AA_00171 | hypothetical protein CYLTODRAFT_425697 [Cylindrobasidium torrendii FP15055 ss-10] | chr06_AA_00308 | hypothetical protein CYLTODRAFT_397357 [Cylindrobasidium torrendii FP15055 ss-10] | 0.107825238 |
| chr02_AA_00171 | hypothetical protein CYLTODRAFT_425697 [Cylindrobasidium torrendii FP15055 ss-10] | chr06_AA_00315 | mitochondrial carrier [Cylindrobasidium torrendii FP15055 ss-10] | 0.232275516 |
| chr02_AA_00171 | hypothetical protein CYLTODRAFT_425697 [Cylindrobasidium torrendii FP15055 ss-10] | chr06_AA_00366 | uncharacterized protein ARMOST_02422 [Armillaria ostoyae] | 0.117429311 |
| chr02_AA_00171 | hypothetical protein CYLTODRAFT_425697 [Cylindrobasidium torrendii FP15055 ss-10] | chr06_AA_00433 | putative ectomycorrhiza-upregulated exo-beta-1,3-glucanase GH5 [Flammulina velutipes] | 0.181754879 |
| chr02_AA_00171 | hypothetical protein CYLTODRAFT_425697 [Cylindrobasidium torrendii FP15055 ss-10] | chr06_AA_00511 | hypothetical protein CYLTODRAFT_487823 [Cylindrobasidium torrendii FP15055 ss-10] | 0.231468064 |
| chr02_AA_00171 | hypothetical protein CYLTODRAFT_425697 [Cylindrobasidium torrendii FP15055 ss-10] | chr06_AA_00524 | hypothetical protein ARMGADRAFT_1111301 [Armillaria gallica] | 0.153747824 |
| chr02_AA_00171 | hypothetical protein CYLTODRAFT_425697 [Cylindrobasidium torrendii FP15055 ss-10] | chr06_AA_00666 | hypothetical protein [Flammulina velutipes] | 0.115792146 |
| chr02_AA_00171 | hypothetical protein CYLTODRAFT_425697 [Cylindrobasidium torrendii FP15055 ss-10] | chr06_AA_00682 | hypothetical protein HYDPIDRAFT_112868 [Hydnomerulius pinastri MD-312] | 0.111983931 |
| chr02_AA_00171 | hypothetical protein CYLTODRAFT_425697 [Cylindrobasidium torrendii FP15055 ss-10] | chr06_AA_00694 | Zinc-regulated transporter 1 [Hypsizygus marmoreus] | 0.183062345 |
| chr02_AA_00171 | hypothetical protein CYLTODRAFT_425697 [Cylindrobasidium torrendii FP15055 ss-10] | chr06_AA_00703 | hypothetical protein CYLTODRAFT_398145 [Cylindrobasidium torrendii FP15055 ss-10] | 0.241586628 |
| chr02_AA_00171 | hypothetical protein CYLTODRAFT_425697 [Cylindrobasidium torrendii FP15055 ss-10] | chr06_AA_00707 | glycerol kinase [Armillaria solidipes] | 0.228386905 |
| chr02_AA_00171 | hypothetical protein CYLTODRAFT_425697 [Cylindrobasidium torrendii FP15055 ss-10] | chr06_AA_00738 | pyridoxal phosphate-dependent enzyme, beta subunit [Cylindrobasidium torrendii FP15055 ss-10] | 0.253620824 |
| chr02_AA_00171 | hypothetical protein CYLTODRAFT_425697 [Cylindrobasidium torrendii FP15055 ss-10] | chr06_AA_00949 | hypothetical protein CYLTODRAFT_427642 [Cylindrobasidium torrendii FP15055 ss-10] | 0.145748483 |
| chr02_AA_00171 | hypothetical protein CYLTODRAFT_425697 [Cylindrobasidium torrendii FP15055 ss-10] | chr06_AA_01031 | glycoside hydrolase family 43 protein [Periconia macrospinosa] | 0.166019872 |
| chr02_AA_00171 | hypothetical protein CYLTODRAFT_425697 [Cylindrobasidium torrendii FP15055 ss-10] | chr07_AA_00156 | uncharacterized protein ARMOST_00326 [Armillaria ostoyae] | 0.237107399 |
| chr02_AA_00171 | hypothetical protein CYLTODRAFT_425697 [Cylindrobasidium torrendii FP15055 ss-10] | chr07_AA_00159 | FAD-binding domain-containing protein [Armillaria solidipes] | 0.108373651 |
| chr02_AA_00171 | hypothetical protein CYLTODRAFT_425697 [Cylindrobasidium torrendii FP15055 ss-10] | chr07_AA_00281 | NAD(P)-binding protein [Armillaria gallica] | 0.252702065 |
| chr02_AA_00171 | hypothetical protein CYLTODRAFT_425697 [Cylindrobasidium torrendii FP15055 ss-10] | chr07_AA_00380 | ectomycorrhiza-regulated esterase [Armillaria solidipes] | 0.179528867 |
| chr02_AA_00171 | hypothetical protein CYLTODRAFT_425697 [Cylindrobasidium torrendii FP15055 ss-10] | chr07_AA_00414 | MFS general substrate transporter [Armillaria gallica] | 0.239927401 |
| chr02_AA_00171 | hypothetical protein CYLTODRAFT_425697 [Cylindrobasidium torrendii FP15055 ss-10] | chr07_AA_00419 | hypothetical protein CYLTODRAFT_277105 [Cylindrobasidium torrendii FP15055 ss-10] | 0.107405747 |
| chr02_AA_00171 | hypothetical protein CYLTODRAFT_425697 [Cylindrobasidium torrendii FP15055 ss-10] | chr07_AA_00464 | cytochrome P450 [Cylindrobasidium torrendii FP15055 ss-10] | 0.147290718 |
| chr02_AA_00171 | hypothetical protein CYLTODRAFT_425697 [Cylindrobasidium torrendii FP15055 ss-10] | chr07_AA_00560 | hypothetical protein CYLTODRAFT_416229 [Cylindrobasidium torrendii FP15055 ss-10] | 0.253343331 |
| chr02_AA_00171 | hypothetical protein CYLTODRAFT_425697 [Cylindrobasidium torrendii FP15055 ss-10] | chr07_AA_00561 | hypothetical protein ARMGADRAFT_1031622 [Armillaria gallica] | 0.12650683 |
| chr02_AA_00171 | hypothetical protein CYLTODRAFT_425697 [Cylindrobasidium torrendii FP15055 ss-10] | chr07_AA_00567 | hypothetical protein CYLTODRAFT_406012 [Cylindrobasidium torrendii FP15055 ss-10] | 0.124634244 |
| chr02_AA_00171 | hypothetical protein CYLTODRAFT_425697 [Cylindrobasidium torrendii FP15055 ss-10] | chr07_AA_00601 | hypothetical protein GYMLUDRAFT_71620 [Gymnopus luxurians FD-317 M1] | 0.13260454 |
| chr02_AA_00171 | hypothetical protein CYLTODRAFT_425697 [Cylindrobasidium torrendii FP15055 ss-10] | chr07_AA_00617 | NAD-dependent formate dehydrogenase [Moniliophthora perniciosa] | 0.282835664 |
| chr02_AA_00171 | hypothetical protein CYLTODRAFT_425697 [Cylindrobasidium torrendii FP15055 ss-10] | chr07_AA_00645 | aldehyde dehydrogenase [Armillaria gallica] | 0.177070945 |
| chr02_AA_00171 | hypothetical protein CYLTODRAFT_425697 [Cylindrobasidium torrendii FP15055 ss-10] | chr07_AA_00703 | potassium/sodium eff [Cylindrobasidium torrendii FP15055 ss-10] | 0.259748138 |
| chr02_AA_00171 | hypothetical protein CYLTODRAFT_425697 [Cylindrobasidium torrendii FP15055 ss-10] | chr07_AA_00722 | hypothetical protein K443DRAFT_93998 [Laccaria amethystina LaAM-08-1] | 0.202166721 |
| chr02_AA_00171 | hypothetical protein CYLTODRAFT_425697 [Cylindrobasidium torrendii FP15055 ss-10] | chr07_AA_00739 | hypothetical protein CYLTODRAFT_417357 [Cylindrobasidium torrendii FP15055 ss-10] | 0.235979705 |
| chr02_AA_00171 | hypothetical protein CYLTODRAFT_425697 [Cylindrobasidium torrendii FP15055 ss-10] | chr07_AA_00756 | SAICAR synthase-like protein [Cylindrobasidium torrendii FP15055 ss-10] | 0.138274877 |
| chr02_AA_00171 | hypothetical protein CYLTODRAFT_425697 [Cylindrobasidium torrendii FP15055 ss-10] | chr07_AA_00826 | Metallo-hydrolase/oxidoreductase [Armillaria gallica] | 0.146567065 |
| chr02_AA_00171 | hypothetical protein CYLTODRAFT_425697 [Cylindrobasidium torrendii FP15055 ss-10] | chr07_AA_00827 | hypothetical protein LENED_003150 [Lentinula edodes] | 0.20669002 |
| chr02_AA_00171 | hypothetical protein CYLTODRAFT_425697 [Cylindrobasidium torrendii FP15055 ss-10] | chr07_AA_00829 | general substrate transporter [Cylindrobasidium torrendii FP15055 ss-10] | 0.20060531 |
| chr02_AA_00171 | hypothetical protein CYLTODRAFT_425697 [Cylindrobasidium torrendii FP15055 ss-10] | chr07_AA_00907 | uncharacterized protein ARMOST_00985 [Armillaria ostoyae] | 0.207983232 |
| chr02_AA_00171 | hypothetical protein CYLTODRAFT_425697 [Cylindrobasidium torrendii FP15055 ss-10] | chr07_AA_00951 | acyl-CoA dehydrogenase domain-containing protein [Cylindrobasidium torrendii FP15055 ss-10] | 0.146843958 |
| chr02_AA_00171 | hypothetical protein CYLTODRAFT_425697 [Cylindrobasidium torrendii FP15055 ss-10] | chr07_AA_01020 | extracellular GDSL-like lipase/acylhydrolase [Glonium stellatum] | 0.201065193 |
| chr02_AA_00171 | hypothetical protein CYLTODRAFT_425697 [Cylindrobasidium torrendii FP15055 ss-10] | chr07_AA_01021 | hypothetical protein PLEOSDRAFT_1096996 [Pleurotus ostreatus PC15] | 0.22439598 |
| chr02_AA_00171 | hypothetical protein CYLTODRAFT_425697 [Cylindrobasidium torrendii FP15055 ss-10] | chr07_AA_01044 | uncharacterized protein ARMOST_19175 [Armillaria ostoyae] | 0.249404522 |
| chr02_AA_00171 | hypothetical protein CYLTODRAFT_425697 [Cylindrobasidium torrendii FP15055 ss-10] | chr07_AA_01051 | MFS general substrate transporter [Cylindrobasidium torrendii FP15055 ss-10] | 0.189935104 |
| chr02_AA_00171 | hypothetical protein CYLTODRAFT_425697 [Cylindrobasidium torrendii FP15055 ss-10] | chr07_AA_01089 | cysteine proteinase [Cylindrobasidium torrendii FP15055 ss-10] | 0.256427858 |
| chr02_AA_00171 | hypothetical protein CYLTODRAFT_425697 [Cylindrobasidium torrendii FP15055 ss-10] | chr08_AA_00018 | putative peroxiredoxin Q [Flammulina velutipes] | 0.222754925 |
| chr02_AA_00171 | hypothetical protein CYLTODRAFT_425697 [Cylindrobasidium torrendii FP15055 ss-10] | chr08_AA_00106 | hypothetical protein ARMGADRAFT_100162 [Armillaria gallica] | 0.151578454 |
| chr02_AA_00171 | hypothetical protein CYLTODRAFT_425697 [Cylindrobasidium torrendii FP15055 ss-10] | chr08_AA_00260 | CDF-like metal transporter [Armillaria solidipes] | 0.179601495 |
| chr02_AA_00171 | hypothetical protein CYLTODRAFT_425697 [Cylindrobasidium torrendii FP15055 ss-10] | chr08_AA_00287 | hypothetical protein PLICRDRAFT_118622 [Plicaturopsis crispa FD-325 SS-3] | 0.143851837 |
| chr02_AA_00171 | hypothetical protein CYLTODRAFT_425697 [Cylindrobasidium torrendii FP15055 ss-10] | chr08_AA_00338 | DUF1479-domain-containing protein [Cylindrobasidium torrendii FP15055 ss-10] | 0.274113951 |
| chr02_AA_00171 | hypothetical protein CYLTODRAFT_425697 [Cylindrobasidium torrendii FP15055 ss-10] | chr08_AA_00383 | NAD(P)-binding protein [Armillaria gallica] | 0.103037622 |
| chr02_AA_00171 | hypothetical protein CYLTODRAFT_425697 [Cylindrobasidium torrendii FP15055 ss-10] | chr08_AA_00413 | hypothetical protein ARMGADRAFT_391312 [Armillaria gallica] | 0.180349139 |
| chr02_AA_00171 | hypothetical protein CYLTODRAFT_425697 [Cylindrobasidium torrendii FP15055 ss-10] | chr08_AA_00501 | hypothetical protein CYLTODRAFT_416851 [Cylindrobasidium torrendii FP15055 ss-10] | 0.231479831 |
| chr02_AA_00171 | hypothetical protein CYLTODRAFT_425697 [Cylindrobasidium torrendii FP15055 ss-10] | chr08_AA_00542 | hypothetical protein ARMGADRAFT_1159894 [Armillaria gallica] | 0.227734482 |
| chr02_AA_00171 | hypothetical protein CYLTODRAFT_425697 [Cylindrobasidium torrendii FP15055 ss-10] | chr08_AA_00621 | hypothetical protein ARMGADRAFT_982650 [Armillaria gallica] | 0.219376282 |
| chr02_AA_00171 | hypothetical protein CYLTODRAFT_425697 [Cylindrobasidium torrendii FP15055 ss-10] | chr08_AA_00651 | related to isp4-oligopeptide transporter [Armillaria ostoyae] | 0.165843423 |
| chr02_AA_00171 | hypothetical protein CYLTODRAFT_425697 [Cylindrobasidium torrendii FP15055 ss-10] | chr08_AA_00687 | Clavaminate synthase-like protein [Armillaria solidipes] | 0.297006505 |
| chr02_AA_00171 | hypothetical protein CYLTODRAFT_425697 [Cylindrobasidium torrendii FP15055 ss-10] | chr08_AA_00856 | cytochrome p450 [Moniliophthora roreri MCA 2997] | 0.162465743 |
| chr02_AA_00171 | hypothetical protein CYLTODRAFT_425697 [Cylindrobasidium torrendii FP15055 ss-10] | chr08_AA_00858 | cytochrome P450 [Cylindrobasidium torrendii FP15055 ss-10] | 0.268708598 |
| chr02_AA_00171 | hypothetical protein CYLTODRAFT_425697 [Cylindrobasidium torrendii FP15055 ss-10] | chr08_AA_00936 | MFS general substrate transporter [Armillaria gallica] | 0.204266802 |
| chr02_AA_00171 | hypothetical protein CYLTODRAFT_425697 [Cylindrobasidium torrendii FP15055 ss-10] | chr08_AA_00948 | probable glutamate dehydrogenase, NAD(+)-specific [Armillaria ostoyae] | 0.193335803 |
| chr02_AA_00171 | hypothetical protein CYLTODRAFT_425697 [Cylindrobasidium torrendii FP15055 ss-10] | chr08_AA_00984 | cytochrome P450 monooxygenase pc-bph [Armillaria gallica] | 0.205347279 |
| chr02_AA_00171 | hypothetical protein CYLTODRAFT_425697 [Cylindrobasidium torrendii FP15055 ss-10] | chr08_AA_01027 | hypothetical protein CYLTODRAFT_357890 [Cylindrobasidium torrendii FP15055 ss-10] | 0.262020897 |
| chr02_AA_00171 | hypothetical protein CYLTODRAFT_425697 [Cylindrobasidium torrendii FP15055 ss-10] | chr08_AA_01046 | MFS general substrate transporter [Armillaria gallica] | 0.229087068 |
| chr02_AA_00171 | hypothetical protein CYLTODRAFT_425697 [Cylindrobasidium torrendii FP15055 ss-10] | chr08_AA_01069 | alpha/beta-hydrolase [Armillaria solidipes] | 0.153571582 |
| chr02_AA_00171 | hypothetical protein CYLTODRAFT_425697 [Cylindrobasidium torrendii FP15055 ss-10] | chr08_AA_01077 | GroES-like protein [Cylindrobasidium torrendii FP15055 ss-10] | 0.124135248 |
| chr02_AA_00171 | hypothetical protein CYLTODRAFT_425697 [Cylindrobasidium torrendii FP15055 ss-10] | chr08_AA_01081 | uncharacterized protein ARMOST_20173 [Armillaria ostoyae] | 0.151943692 |
| chr02_AA_00171 | hypothetical protein CYLTODRAFT_425697 [Cylindrobasidium torrendii FP15055 ss-10] | chr08_AA_01091 | uncharacterized protein ARMOST_03386 [Armillaria ostoyae] | 0.260468743 |
| chr02_AA_00171 | hypothetical protein CYLTODRAFT_425697 [Cylindrobasidium torrendii FP15055 ss-10] | chr08_AA_01097 | hypothetical protein GYMLUDRAFT_32937 [Gymnopus luxurians FD-317 M1] | 0.225595023 |
| chr02_AA_00171 | hypothetical protein CYLTODRAFT_425697 [Cylindrobasidium torrendii FP15055 ss-10] | chr08_AA_01155 | related to 3-oxoacyl CoA thiolase [Armillaria ostoyae] | 0.235846826 |
| chr02_AA_00171 | hypothetical protein CYLTODRAFT_425697 [Cylindrobasidium torrendii FP15055 ss-10] | chr08_AA_01172 | carbohydrate esterase family 1 protein, partial [Cylindrobasidium torrendii FP15055 ss-10] | 0.147569786 |
| chr02_AA_00171 | hypothetical protein CYLTODRAFT_425697 [Cylindrobasidium torrendii FP15055 ss-10] | chr08_AA_01176 | hypothetical protein HYPSUDRAFT_61393 [Hypholoma sublateritium FD-334 SS-4] | 0.167711729 |
| chr02_AA_00171 | hypothetical protein CYLTODRAFT_425697 [Cylindrobasidium torrendii FP15055 ss-10] | chr08_AA_01257 | hypothetical protein ARMSODRAFT_949486 [Armillaria solidipes] | 0.260564651 |
| chr02_AA_00171 | hypothetical protein CYLTODRAFT_425697 [Cylindrobasidium torrendii FP15055 ss-10] | chr08_AA_01259 | Aldo/keto reductase [Armillaria gallica] | 0.235426384 |
| chr02_AA_00171 | hypothetical protein CYLTODRAFT_425697 [Cylindrobasidium torrendii FP15055 ss-10] | chr09_AA_00036 | hypothetical protein ARMGADRAFT_1070289 [Armillaria gallica] | 0.225720769 |
| chr02_AA_00171 | hypothetical protein CYLTODRAFT_425697 [Cylindrobasidium torrendii FP15055 ss-10] | chr09_AA_00051 | GMC oxidoreductase [Cylindrobasidium torrendii FP15055 ss-10] | 0.282683246 |
| chr02_AA_00171 | hypothetical protein CYLTODRAFT_425697 [Cylindrobasidium torrendii FP15055 ss-10] | chr09_AA_00052 | GMC oxidoreductase [Cylindrobasidium torrendii FP15055 ss-10] | 0.309321349 |
| chr02_AA_00171 | hypothetical protein CYLTODRAFT_425697 [Cylindrobasidium torrendii FP15055 ss-10] | chr09_AA_00095 | hypothetical protein CYLTODRAFT_422329 [Cylindrobasidium torrendii FP15055 ss-10] | 0.202304734 |
| chr02_AA_00171 | hypothetical protein CYLTODRAFT_425697 [Cylindrobasidium torrendii FP15055 ss-10] | chr09_AA_00100 | hypothetical protein K503DRAFT_238266 [Rhizopogon vinicolor AM-OR11-026] | 0.171259972 |
| chr02_AA_00171 | hypothetical protein CYLTODRAFT_425697 [Cylindrobasidium torrendii FP15055 ss-10] | chr09_AA_00162 | hypothetical protein ARMGADRAFT_961910 [Armillaria gallica] | 0.22477545 |
| chr02_AA_00171 | hypothetical protein CYLTODRAFT_425697 [Cylindrobasidium torrendii FP15055 ss-10] | chr09_AA_00190 | putative exo-beta-1,3-glucanase [Flammulina velutipes] | 0.194977033 |
| chr02_AA_00171 | hypothetical protein CYLTODRAFT_425697 [Cylindrobasidium torrendii FP15055 ss-10] | chr09_AA_00191 | exo-beta-1,3-glucanase [Armillaria solidipes] | 0.194650969 |
| chr02_AA_00171 | hypothetical protein CYLTODRAFT_425697 [Cylindrobasidium torrendii FP15055 ss-10] | chr09_AA_00229 | succinyl-CoA:3-ketoacid-coenzyme A transferase [Cylindrobasidium torrendii FP15055 ss-10] | 0.199385023 |
| chr02_AA_00171 | hypothetical protein CYLTODRAFT_425697 [Cylindrobasidium torrendii FP15055 ss-10] | chr09_AA_00241 | SNF2 chromatin remodeling protein [Armillaria solidipes] | 0.247827433 |
| chr02_AA_00171 | hypothetical protein CYLTODRAFT_425697 [Cylindrobasidium torrendii FP15055 ss-10] | chr09_AA_00312 | putative beta-glucosidase [Flammulina velutipes] | 0.136324982 |
| chr02_AA_00171 | hypothetical protein CYLTODRAFT_425697 [Cylindrobasidium torrendii FP15055 ss-10] | chr09_AA_00406 | uncharacterized protein ARMOST_17180 [Armillaria ostoyae] | 0.196736549 |
| chr02_AA_00171 | hypothetical protein CYLTODRAFT_425697 [Cylindrobasidium torrendii FP15055 ss-10] | chr09_AA_00410 | hypothetical protein AMATHDRAFT_73341 [Amanita thiersii Skay4041] | 0.123904404 |
| chr02_AA_00171 | hypothetical protein CYLTODRAFT_425697 [Cylindrobasidium torrendii FP15055 ss-10] | chr09_AA_00518 | AAA-domain-containing protein [Cylindrobasidium torrendii FP15055 ss-10] | 0.222002627 |
| chr02_AA_00171 | hypothetical protein CYLTODRAFT_425697 [Cylindrobasidium torrendii FP15055 ss-10] | chr09_AA_00680 | hypothetical protein CYLTODRAFT_395198 [Cylindrobasidium torrendii FP15055 ss-10] | 0.213757139 |
| chr02_AA_00171 | hypothetical protein CYLTODRAFT_425697 [Cylindrobasidium torrendii FP15055 ss-10] | chr09_AA_00719 | hypothetical protein PLEOSDRAFT_1093927 [Pleurotus ostreatus PC15] | 0.260970873 |
| chr02_AA_00171 | hypothetical protein CYLTODRAFT_425697 [Cylindrobasidium torrendii FP15055 ss-10] | chr09_AA_00762 | transketolase [Armillaria solidipes] | 0.147687826 |
| chr02_AA_00171 | hypothetical protein CYLTODRAFT_425697 [Cylindrobasidium torrendii FP15055 ss-10] | chr09_AA_00816 | mandelate racemase muconate lactonizing enzyme family protein [Cylindrobasidium torrendii FP15055 ss-10] | 0.125771058 |
| chr02_AA_00171 | hypothetical protein CYLTODRAFT_425697 [Cylindrobasidium torrendii FP15055 ss-10] | chr09_AA_00838 | cytochrome P450 [Armillaria gallica] | 0.212627719 |
| chr02_AA_00171 | hypothetical protein CYLTODRAFT_425697 [Cylindrobasidium torrendii FP15055 ss-10] | chr09_AA_00892 | hypothetical protein CYLTODRAFT_431446 [Cylindrobasidium torrendii FP15055 ss-10] | 0.232624409 |
| chr02_AA_00171 | hypothetical protein CYLTODRAFT_425697 [Cylindrobasidium torrendii FP15055 ss-10] | chr09_AA_00955 | putative endo-1,4-beta-xylanase precursor [Flammulina velutipes] | 0.147110476 |
| chr02_AA_00171 | hypothetical protein CYLTODRAFT_425697 [Cylindrobasidium torrendii FP15055 ss-10] | chr09_AA_01058 | hypothetical protein ARMSODRAFT_976614 [Armillaria solidipes] | 0.217288874 |
| chr02_AA_00171 | hypothetical protein CYLTODRAFT_425697 [Cylindrobasidium torrendii FP15055 ss-10] | chr09_AA_01099 | general substrate transporter [Armillaria gallica] | 0.201713187 |
| chr02_AA_00171 | hypothetical protein CYLTODRAFT_425697 [Cylindrobasidium torrendii FP15055 ss-10] | chr09_AA_01103 | Metallo-dependent phosphatase [Armillaria solidipes] | 0.265248138 |
| chr02_AA_00171 | hypothetical protein CYLTODRAFT_425697 [Cylindrobasidium torrendii FP15055 ss-10] | chr09_AA_01106 | multidrug transporter [Cylindrobasidium torrendii FP15055 ss-10] | 0.190149769 |
| chr02_AA_00171 | hypothetical protein CYLTODRAFT_425697 [Cylindrobasidium torrendii FP15055 ss-10] | chr09_AA_01141 | hypothetical protein ARMGADRAFT_1063089 [Armillaria gallica] | 0.168915274 |
| chr02_AA_00171 | hypothetical protein CYLTODRAFT_425697 [Cylindrobasidium torrendii FP15055 ss-10] | chr09_AA_01190 | potassium/sodium eff [Cylindrobasidium torrendii FP15055 ss-10] | 0.226559165 |
| chr02_AA_00171 | hypothetical protein CYLTODRAFT_425697 [Cylindrobasidium torrendii FP15055 ss-10] | chr09_AA_01214 | hypothetical protein CYLTODRAFT_417942 [Cylindrobasidium torrendii FP15055 ss-10] | 0.231932965 |
| chr02_AA_00171 | hypothetical protein CYLTODRAFT_425697 [Cylindrobasidium torrendii FP15055 ss-10] | chr09_AA_01224 | uncharacterized protein ARMOST_22339 [Armillaria ostoyae] | 0.182961761 |
| chr02_AA_00171 | hypothetical protein CYLTODRAFT_425697 [Cylindrobasidium torrendii FP15055 ss-10] | chr09_AA_01410 | hypothetical protein CYLTODRAFT_489277 [Cylindrobasidium torrendii FP15055 ss-10] | 0.115850384 |
| chr02_AA_00171 | hypothetical protein CYLTODRAFT_425697 [Cylindrobasidium torrendii FP15055 ss-10] | chr10_AA_00134 | hypothetical protein CYLTODRAFT_349864 [Cylindrobasidium torrendii FP15055 ss-10] | 0.179113554 |
| chr02_AA_00171 | hypothetical protein CYLTODRAFT_425697 [Cylindrobasidium torrendii FP15055 ss-10] | chr10_AA_00164 | dehydrogenase E1 and transketolase domain-containing protein 1 [Cylindrobasidium torrendii FP15055 ss-10] | 0.238339359 |
| chr02_AA_00171 | hypothetical protein CYLTODRAFT_425697 [Cylindrobasidium torrendii FP15055 ss-10] | chr10_AA_00220 | hypothetical protein ARMGADRAFT_962646, partial [Armillaria gallica] | 0.231159531 |
| chr02_AA_00171 | hypothetical protein CYLTODRAFT_425697 [Cylindrobasidium torrendii FP15055 ss-10] | chr10_AA_00256 | uncharacterized protein ARMOST_03672 [Armillaria ostoyae] | 0.160086155 |
| chr02_AA_00171 | hypothetical protein CYLTODRAFT_425697 [Cylindrobasidium torrendii FP15055 ss-10] | chr10_AA_00270 | branched-chain alpha-keto acid dehydrogenase E1-alpha subunit [Cylindrobasidium torrendii FP15055 ss-10] | 0.224794032 |
| chr02_AA_00171 | hypothetical protein CYLTODRAFT_425697 [Cylindrobasidium torrendii FP15055 ss-10] | chr10_AA_00441 | alpha/beta-hydrolase [Armillaria solidipes] | 0.268723323 |
| chr02_AA_00171 | hypothetical protein CYLTODRAFT_425697 [Cylindrobasidium torrendii FP15055 ss-10] | chr10_AA_00499 | hypothetical protein WG66_16220 [Moniliophthora roreri] | 0.265215095 |
| chr02_AA_00171 | hypothetical protein CYLTODRAFT_425697 [Cylindrobasidium torrendii FP15055 ss-10] | chr10_AA_00517 | hypothetical protein CYLTODRAFT_256211 [Cylindrobasidium torrendii FP15055 ss-10] | 0.189893042 |
| chr02_AA_00171 | hypothetical protein CYLTODRAFT_425697 [Cylindrobasidium torrendii FP15055 ss-10] | chr10_AA_00651 | hypothetical protein ARMGADRAFT_897215, partial [Armillaria gallica] | 0.272841544 |
| chr02_AA_00171 | hypothetical protein CYLTODRAFT_425697 [Cylindrobasidium torrendii FP15055 ss-10] | chr10_AA_00662 | Ureohydrolase [Armillaria gallica] | 0.27488554 |
| chr02_AA_00171 | hypothetical protein CYLTODRAFT_425697 [Cylindrobasidium torrendii FP15055 ss-10] | chr10_AA_00693 | hypothetical protein ARMSODRAFT_947720 [Armillaria solidipes] | 0.301448221 |
| chr02_AA_00171 | hypothetical protein CYLTODRAFT_425697 [Cylindrobasidium torrendii FP15055 ss-10] | chr10_AA_00733 | Zinc-regulated transporter 1 [Hypsizygus marmoreus] | 0.153327739 |
| chr02_AA_00171 | hypothetical protein CYLTODRAFT_425697 [Cylindrobasidium torrendii FP15055 ss-10] | chr10_AA_00735 | glycoside hydrolase family 31 protein [Cylindrobasidium torrendii FP15055 ss-10] | 0.143935347 |
| chr02_AA_00171 | hypothetical protein CYLTODRAFT_425697 [Cylindrobasidium torrendii FP15055 ss-10] | chr10_AA_00944 | general substrate transporter [Cylindrobasidium torrendii FP15055 ss-10] | 0.238134089 |
| chr02_AA_00171 | hypothetical protein CYLTODRAFT_425697 [Cylindrobasidium torrendii FP15055 ss-10] | chr10_AA_01056 | hypothetical protein ARMGADRAFT_1020988 [Armillaria gallica] | 0.114664092 |
| chr02_AA_00171 | hypothetical protein CYLTODRAFT_425697 [Cylindrobasidium torrendii FP15055 ss-10] | chr10_AA_01063 | glycoside hydrolase family 43 protein [Amanita thiersii Skay4041] | 0.192931132 |
| chr02_AA_00171 | hypothetical protein CYLTODRAFT_425697 [Cylindrobasidium torrendii FP15055 ss-10] | chr10_AA_01143 | uncharacterized protein ARMOST_08409 [Armillaria ostoyae] | 0.276422682 |
| chr02_AA_00171 | hypothetical protein CYLTODRAFT_425697 [Cylindrobasidium torrendii FP15055 ss-10] | chr10_AA_01157 | glycoside hydrolase family 53 protein [Cylindrobasidium torrendii FP15055 ss-10] | 0.283061017 |
| chr02_AA_00171 | hypothetical protein CYLTODRAFT_425697 [Cylindrobasidium torrendii FP15055 ss-10] | chr10_AA_01201 | acyltransferase ChoActase/COT/CPT [Armillaria gallica] | 0.18425622 |
| chr02_AA_00171 | hypothetical protein CYLTODRAFT_425697 [Cylindrobasidium torrendii FP15055 ss-10] | chr10_AA_01213 | hypothetical protein JAAARDRAFT_210932 [Jaapia argillacea MUCL 33604] | 0.175684973 |
| chr02_AA_00171 | hypothetical protein CYLTODRAFT_425697 [Cylindrobasidium torrendii FP15055 ss-10] | chr10_AA_01231 | hypothetical protein ARMSODRAFT_1019532 [Armillaria solidipes] | 0.235036287 |
| chr02_AA_00171 | hypothetical protein CYLTODRAFT_425697 [Cylindrobasidium torrendii FP15055 ss-10] | chr10_AA_01309 | hypothetical protein PHLCEN_2v2282 [Phlebia centrifuga] | 0.127144447 |
| chr02_AA_00171 | hypothetical protein CYLTODRAFT_425697 [Cylindrobasidium torrendii FP15055 ss-10] | chr10_AA_01341 | glucuronyl hydrolase [Armillaria solidipes] | 0.285263563 |
| chr02_AA_00171 | hypothetical protein CYLTODRAFT_425697 [Cylindrobasidium torrendii FP15055 ss-10] | chr10_AA_01576 | D-arabinitol 2-dehydrogenase [ribulose-forming] [Hypsizygus marmoreus] | 0.177300237 |
| chr02_AA_00171 | hypothetical protein CYLTODRAFT_425697 [Cylindrobasidium torrendii FP15055 ss-10] | chr10_AA_01664 | hypothetical protein CYLTODRAFT_404266 [Cylindrobasidium torrendii FP15055 ss-10] | 0.296389798 |
| chr02_AA_00171 | hypothetical protein CYLTODRAFT_425697 [Cylindrobasidium torrendii FP15055 ss-10] | chr11_AA_00042 | kinase-like protein [Cylindrobasidium torrendii FP15055 ss-10] | 0.278660942 |
| chr02_AA_00171 | hypothetical protein CYLTODRAFT_425697 [Cylindrobasidium torrendii FP15055 ss-10] | chr11_AA_00094 | homogentisate 1,2-dioxygenase [Armillaria solidipes] | 0.169529472 |
| chr02_AA_00171 | hypothetical protein CYLTODRAFT_425697 [Cylindrobasidium torrendii FP15055 ss-10] | chr11_AA_00103 | hypothetical protein M413DRAFT_370146 [Hebeloma cylindrosporum h7] | 0.229789956 |
| chr02_AA_00171 | hypothetical protein CYLTODRAFT_425697 [Cylindrobasidium torrendii FP15055 ss-10] | chr11_AA_00145 | hypothetical protein CYLTODRAFT_243491 [Cylindrobasidium torrendii FP15055 ss-10] | 0.145438174 |
| chr02_AA_00171 | hypothetical protein CYLTODRAFT_425697 [Cylindrobasidium torrendii FP15055 ss-10] | chr11_AA_00233 | hypothetical protein ARMGADRAFT_977028 [Armillaria gallica] | 0.158214089 |
| chr02_AA_00171 | hypothetical protein CYLTODRAFT_425697 [Cylindrobasidium torrendii FP15055 ss-10] | chr11_AA_00265 | FAD-linked oxidoreductase [Armillaria solidipes] | 0.204845038 |
| chr02_AA_00171 | hypothetical protein CYLTODRAFT_425697 [Cylindrobasidium torrendii FP15055 ss-10] | chr11_AA_00277 | uncharacterized protein ARMOST_10602 [Armillaria ostoyae] | 0.114228472 |
| chr02_AA_00171 | hypothetical protein CYLTODRAFT_425697 [Cylindrobasidium torrendii FP15055 ss-10] | chr11_AA_00499 | GroES-like protein [Armillaria solidipes] | 0.23684416 |
| chr02_AA_00171 | hypothetical protein CYLTODRAFT_425697 [Cylindrobasidium torrendii FP15055 ss-10] | chr11_AA_00504 | MFS general substrate transporter [Cylindrobasidium torrendii FP15055 ss-10] | 0.266804418 |
| chr02_AA_00171 | hypothetical protein CYLTODRAFT_425697 [Cylindrobasidium torrendii FP15055 ss-10] | chr11_AA_00606 | hypothetical protein CYLTODRAFT_418833 [Cylindrobasidium torrendii FP15055 ss-10] | 0.231282817 |
| chr02_AA_00171 | hypothetical protein CYLTODRAFT_425697 [Cylindrobasidium torrendii FP15055 ss-10] | chr11_AA_00638 | hypothetical protein ARMSODRAFT_953107 [Armillaria solidipes] | 0.15017919 |
| chr02_AA_00171 | hypothetical protein CYLTODRAFT_425697 [Cylindrobasidium torrendii FP15055 ss-10] | chr11_AA_00701 | uncharacterized protein ARMOST_05344 [Armillaria ostoyae] | 0.125794057 |
| chr02_AA_00171 | hypothetical protein CYLTODRAFT_425697 [Cylindrobasidium torrendii FP15055 ss-10] | chr11_AA_00719 | hypothetical protein ARMGADRAFT_1019784, partial [Armillaria gallica] | 0.115412095 |
| chr02_AA_00171 | hypothetical protein CYLTODRAFT_425697 [Cylindrobasidium torrendii FP15055 ss-10] | chr11_AA_00724 | WD40 repeat-like protein [Armillaria solidipes] | 0.331523591 |
| chr02_AA_00171 | hypothetical protein CYLTODRAFT_425697 [Cylindrobasidium torrendii FP15055 ss-10] | chr11_AA_00790 | uncharacterized protein ARMOST_05401 [Armillaria ostoyae] | 0.152690479 |
| chr02_AA_00171 | hypothetical protein CYLTODRAFT_425697 [Cylindrobasidium torrendii FP15055 ss-10] | chr11_AA_00878 | hypothetical protein CYLTODRAFT_430286 [Cylindrobasidium torrendii FP15055 ss-10] | 0.228575725 |
| chr02_AA_00171 | hypothetical protein CYLTODRAFT_425697 [Cylindrobasidium torrendii FP15055 ss-10] | chr11_AA_00928 | putative malate synthase [Flammulina velutipes] | 0.237953993 |
| chr02_AA_00171 | hypothetical protein CYLTODRAFT_425697 [Cylindrobasidium torrendii FP15055 ss-10] | chr11_AA_00997 | Annexin [Cylindrobasidium torrendii FP15055 ss-10] | 0.200941785 |
| chr02_AA_00171 | hypothetical protein CYLTODRAFT_425697 [Cylindrobasidium torrendii FP15055 ss-10] | chr11_AA_01033 | glycoside hydrolase family 51 protein [Cylindrobasidium torrendii FP15055 ss-10] | 0.257538092 |
| chr02_AA_00171 | hypothetical protein CYLTODRAFT_425697 [Cylindrobasidium torrendii FP15055 ss-10] | chr11_AA_01042 | acetoin reductase family protein [Cylindrobasidium torrendii FP15055 ss-10] | 0.158430188 |
| chr02_AA_00171 | hypothetical protein CYLTODRAFT_425697 [Cylindrobasidium torrendii FP15055 ss-10] | chr11_AA_01046 | hypothetical protein CYLTODRAFT_428160 [Cylindrobasidium torrendii FP15055 ss-10] | 0.222000477 |
| chr02_AA_00171 | hypothetical protein CYLTODRAFT_425697 [Cylindrobasidium torrendii FP15055 ss-10] | chr11_AA_01097 | glycoside hydrolase family 3 protein [Cylindrobasidium torrendii FP15055 ss-10] | 0.196804533 |
| chr02_AA_00171 | hypothetical protein CYLTODRAFT_425697 [Cylindrobasidium torrendii FP15055 ss-10] | chr11_AA_01106 | related to stomatin [Armillaria ostoyae] | 0.177198671 |
| chr02_AA_00171 | hypothetical protein CYLTODRAFT_425697 [Cylindrobasidium torrendii FP15055 ss-10] | chr11_AA_01313 | hypothetical protein Hypma_000372 [Hypsizygus marmoreus] | 0.245634605 |
| chr02_AA_00171 | hypothetical protein CYLTODRAFT_425697 [Cylindrobasidium torrendii FP15055 ss-10] | chr11_AA_01324 | terpenoid synthase [Cylindrobasidium torrendii FP15055 ss-10] | 0.164179403 |
| chr02_AA_00171 | hypothetical protein CYLTODRAFT_425697 [Cylindrobasidium torrendii FP15055 ss-10] | chr11_AA_01456 | uncharacterized protein ARMOST_16541 [Armillaria ostoyae] | 0.198199712 |
| chr02_AA_00171 | hypothetical protein CYLTODRAFT_425697 [Cylindrobasidium torrendii FP15055 ss-10] | chr11_AA_01461 | ricin B-like lectin [Armillaria gallica] | 0.151021709 |
| chr02_AA_00171 | hypothetical protein CYLTODRAFT_425697 [Cylindrobasidium torrendii FP15055 ss-10] | chr11_AA_01510 | hypothetical protein ARMGADRAFT_1015707 [Armillaria gallica] | 0.113360617 |
| chr02_AA_00171 | hypothetical protein CYLTODRAFT_425697 [Cylindrobasidium torrendii FP15055 ss-10] | chr11_AA_01636 | hypothetical protein CYLTODRAFT_421880 [Cylindrobasidium torrendii FP15055 ss-10] | 0.248510324 |
| chr02_AA_00171 | hypothetical protein CYLTODRAFT_425697 [Cylindrobasidium torrendii FP15055 ss-10] | chr11_AA_01644 | hypothetical protein CYLTODRAFT_438918 [Cylindrobasidium torrendii FP15055 ss-10] | 0.114306805 |
| chr02_AA_00171 | hypothetical protein CYLTODRAFT_425697 [Cylindrobasidium torrendii FP15055 ss-10] | chr11_AA_01664 | hypothetical protein CYLTODRAFT_421876 [Cylindrobasidium torrendii FP15055 ss-10] | 0.199508379 |
| chr02_AA_00171 | hypothetical protein CYLTODRAFT_425697 [Cylindrobasidium torrendii FP15055 ss-10] | chr11_AA_01685 | 1-aminocyclopropane-1-carboxylate deaminase [Armillaria solidipes] | 0.136525489 |
| chr02_AA_00171 | hypothetical protein CYLTODRAFT_425697 [Cylindrobasidium torrendii FP15055 ss-10] | chr11_AA_01686 | hypothetical protein CYLTODRAFT_348076 [Cylindrobasidium torrendii FP15055 ss-10] | 0.262666015 |
| chr02_AA_00171 | hypothetical protein CYLTODRAFT_425697 [Cylindrobasidium torrendii FP15055 ss-10] | chr11_AA_01711 | hypothetical protein ARMGADRAFT_1046867 [Armillaria gallica] | 0.27207836 |
| chr02_AA_00171 | hypothetical protein CYLTODRAFT_425697 [Cylindrobasidium torrendii FP15055 ss-10] | chr11_AA_01732 | glycoside hydrolase family 76 protein [Cylindrobasidium torrendii FP15055 ss-10] | 0.212026783 |
| chr02_AA_00171 | hypothetical protein CYLTODRAFT_425697 [Cylindrobasidium torrendii FP15055 ss-10] | chr11_AA_01733 | glycoside hydrolase family 76 protein [Cylindrobasidium torrendii FP15055 ss-10] | 0.218760366 |
| chr08_AA_00687 | Clavaminate synthase-like protein [Armillaria solidipes] | chr08_AA_00856 | cytochrome p450 [Moniliophthora roreri MCA 2997] | 0.23440294 |
| chr08_AA_00687 | Clavaminate synthase-like protein [Armillaria solidipes] | chr08_AA_00858 | cytochrome P450 [Cylindrobasidium torrendii FP15055 ss-10] | 0.294991574 |
| chr08_AA_00687 | Clavaminate synthase-like protein [Armillaria solidipes] | chr08_AA_00936 | MFS general substrate transporter [Armillaria gallica] | 0.257895547 |
| chr08_AA_00687 | Clavaminate synthase-like protein [Armillaria solidipes] | chr08_AA_00948 | probable glutamate dehydrogenase, NAD(+)-specific [Armillaria ostoyae] | 0.211877283 |
| chr08_AA_00687 | Clavaminate synthase-like protein [Armillaria solidipes] | chr08_AA_00984 | cytochrome P450 monooxygenase pc-bph [Armillaria gallica] | 0.256481636 |
| chr08_AA_00687 | Clavaminate synthase-like protein [Armillaria solidipes] | chr08_AA_01027 | hypothetical protein CYLTODRAFT_357890 [Cylindrobasidium torrendii FP15055 ss-10] | 0.278699038 |
| chr08_AA_00687 | Clavaminate synthase-like protein [Armillaria solidipes] | chr08_AA_01046 | MFS general substrate transporter [Armillaria gallica] | 0.276351221 |
| chr08_AA_00687 | Clavaminate synthase-like protein [Armillaria solidipes] | chr08_AA_01069 | alpha/beta-hydrolase [Armillaria solidipes] | 0.177445718 |
| chr08_AA_00687 | Clavaminate synthase-like protein [Armillaria solidipes] | chr08_AA_01077 | GroES-like protein [Cylindrobasidium torrendii FP15055 ss-10] | 0.185660946 |
| chr08_AA_00687 | Clavaminate synthase-like protein [Armillaria solidipes] | chr08_AA_01081 | uncharacterized protein ARMOST_20173 [Armillaria ostoyae] | 0.180052208 |
| chr08_AA_00687 | Clavaminate synthase-like protein [Armillaria solidipes] | chr08_AA_01091 | uncharacterized protein ARMOST_03386 [Armillaria ostoyae] | 0.288914212 |
| chr08_AA_00687 | Clavaminate synthase-like protein [Armillaria solidipes] | chr08_AA_01097 | hypothetical protein GYMLUDRAFT_32937 [Gymnopus luxurians FD-317 M1] | 0.176873325 |
| chr08_AA_00687 | Clavaminate synthase-like protein [Armillaria solidipes] | chr08_AA_01155 | related to 3-oxoacyl CoA thiolase [Armillaria ostoyae] | 0.258344218 |
| chr08_AA_00687 | Clavaminate synthase-like protein [Armillaria solidipes] | chr08_AA_01172 | carbohydrate esterase family 1 protein, partial [Cylindrobasidium torrendii FP15055 ss-10] | 0.195586095 |
| chr08_AA_00687 | Clavaminate synthase-like protein [Armillaria solidipes] | chr08_AA_01176 | hypothetical protein HYPSUDRAFT_61393 [Hypholoma sublateritium FD-334 SS-4] | 0.255433828 |
| chr08_AA_00687 | Clavaminate synthase-like protein [Armillaria solidipes] | chr08_AA_01257 | hypothetical protein ARMSODRAFT_949486 [Armillaria solidipes] | 0.276424661 |
| chr08_AA_00687 | Clavaminate synthase-like protein [Armillaria solidipes] | chr08_AA_01259 | Aldo/keto reductase [Armillaria gallica] | 0.280178956 |
| chr08_AA_00687 | Clavaminate synthase-like protein [Armillaria solidipes] | chr09_AA_00023 | hypothetical protein WG66_107 [Moniliophthora roreri] | 0.112188828 |
| chr08_AA_00687 | Clavaminate synthase-like protein [Armillaria solidipes] | chr09_AA_00036 | hypothetical protein ARMGADRAFT_1070289 [Armillaria gallica] | 0.174981217 |
| chr08_AA_00687 | Clavaminate synthase-like protein [Armillaria solidipes] | chr09_AA_00051 | GMC oxidoreductase [Cylindrobasidium torrendii FP15055 ss-10] | 0.273417923 |
| chr08_AA_00687 | Clavaminate synthase-like protein [Armillaria solidipes] | chr09_AA_00052 | GMC oxidoreductase [Cylindrobasidium torrendii FP15055 ss-10] | 0.253240979 |
| chr08_AA_00687 | Clavaminate synthase-like protein [Armillaria solidipes] | chr09_AA_00095 | hypothetical protein CYLTODRAFT_422329 [Cylindrobasidium torrendii FP15055 ss-10] | 0.274668328 |
| chr08_AA_00687 | Clavaminate synthase-like protein [Armillaria solidipes] | chr09_AA_00100 | hypothetical protein K503DRAFT_238266 [Rhizopogon vinicolor AM-OR11-026] | 0.254424158 |
| chr08_AA_00687 | Clavaminate synthase-like protein [Armillaria solidipes] | chr09_AA_00139 | hypothetical protein CYLTODRAFT_424555 [Cylindrobasidium torrendii FP15055 ss-10] | 0.174750818 |
| chr08_AA_00687 | Clavaminate synthase-like protein [Armillaria solidipes] | chr09_AA_00162 | hypothetical protein ARMGADRAFT_961910 [Armillaria gallica] | 0.299509243 |
| chr08_AA_00687 | Clavaminate synthase-like protein [Armillaria solidipes] | chr09_AA_00190 | putative exo-beta-1,3-glucanase [Flammulina velutipes] | 0.149108116 |
| chr08_AA_00687 | Clavaminate synthase-like protein [Armillaria solidipes] | chr09_AA_00191 | exo-beta-1,3-glucanase [Armillaria solidipes] | 0.150748829 |
| chr08_AA_00687 | Clavaminate synthase-like protein [Armillaria solidipes] | chr09_AA_00229 | succinyl-CoA:3-ketoacid-coenzyme A transferase [Cylindrobasidium torrendii FP15055 ss-10] | 0.263349468 |
| chr08_AA_00687 | Clavaminate synthase-like protein [Armillaria solidipes] | chr09_AA_00241 | SNF2 chromatin remodeling protein [Armillaria solidipes] | 0.264615484 |
| chr08_AA_00687 | Clavaminate synthase-like protein [Armillaria solidipes] | chr09_AA_00312 | putative beta-glucosidase [Flammulina velutipes] | 0.197436638 |
| chr08_AA_00687 | Clavaminate synthase-like protein [Armillaria solidipes] | chr09_AA_00406 | uncharacterized protein ARMOST_17180 [Armillaria ostoyae] | 0.209962522 |
| chr08_AA_00687 | Clavaminate synthase-like protein [Armillaria solidipes] | chr09_AA_00410 | hypothetical protein AMATHDRAFT_73341 [Amanita thiersii Skay4041] | 0.104271623 |
| chr08_AA_00687 | Clavaminate synthase-like protein [Armillaria solidipes] | chr09_AA_00518 | AAA-domain-containing protein [Cylindrobasidium torrendii FP15055 ss-10] | 0.235967288 |
| chr08_AA_00687 | Clavaminate synthase-like protein [Armillaria solidipes] | chr09_AA_00551 | hypothetical protein CYLTODRAFT_392634 [Cylindrobasidium torrendii FP15055 ss-10] | 0.185417437 |
| chr08_AA_00687 | Clavaminate synthase-like protein [Armillaria solidipes] | chr09_AA_00680 | hypothetical protein CYLTODRAFT_395198 [Cylindrobasidium torrendii FP15055 ss-10] | 0.278882584 |
| chr08_AA_00687 | Clavaminate synthase-like protein [Armillaria solidipes] | chr09_AA_00719 | hypothetical protein PLEOSDRAFT_1093927 [Pleurotus ostreatus PC15] | 0.270602697 |
| chr08_AA_00687 | Clavaminate synthase-like protein [Armillaria solidipes] | chr09_AA_00762 | transketolase [Armillaria solidipes] | 0.19545069 |
| chr08_AA_00687 | Clavaminate synthase-like protein [Armillaria solidipes] | chr09_AA_00816 | mandelate racemase muconate lactonizing enzyme family protein [Cylindrobasidium torrendii FP15055 ss-10] | 0.192533055 |
| chr08_AA_00687 | Clavaminate synthase-like protein [Armillaria solidipes] | chr09_AA_00838 | cytochrome P450 [Armillaria gallica] | 0.239483945 |
| chr08_AA_00687 | Clavaminate synthase-like protein [Armillaria solidipes] | chr09_AA_00892 | hypothetical protein CYLTODRAFT_431446 [Cylindrobasidium torrendii FP15055 ss-10] | 0.267943846 |
| chr08_AA_00687 | Clavaminate synthase-like protein [Armillaria solidipes] | chr09_AA_00955 | putative endo-1,4-beta-xylanase precursor [Flammulina velutipes] | 0.218403487 |
| chr08_AA_00687 | Clavaminate synthase-like protein [Armillaria solidipes] | chr09_AA_01058 | hypothetical protein ARMSODRAFT_976614 [Armillaria solidipes] | 0.266568697 |
| chr08_AA_00687 | Clavaminate synthase-like protein [Armillaria solidipes] | chr09_AA_01099 | general substrate transporter [Armillaria gallica] | 0.231563587 |
| chr08_AA_00687 | Clavaminate synthase-like protein [Armillaria solidipes] | chr09_AA_01103 | Metallo-dependent phosphatase [Armillaria solidipes] | 0.289125912 |
| chr08_AA_00687 | Clavaminate synthase-like protein [Armillaria solidipes] | chr09_AA_01106 | multidrug transporter [Cylindrobasidium torrendii FP15055 ss-10] | 0.148462434 |
| chr08_AA_00687 | Clavaminate synthase-like protein [Armillaria solidipes] | chr09_AA_01141 | hypothetical protein ARMGADRAFT_1063089 [Armillaria gallica] | 0.246339073 |
| chr08_AA_00687 | Clavaminate synthase-like protein [Armillaria solidipes] | chr09_AA_01190 | potassium/sodium eff [Cylindrobasidium torrendii FP15055 ss-10] | 0.267217148 |
| chr08_AA_00687 | Clavaminate synthase-like protein [Armillaria solidipes] | chr09_AA_01214 | hypothetical protein CYLTODRAFT_417942 [Cylindrobasidium torrendii FP15055 ss-10] | 0.273111956 |
| chr08_AA_00687 | Clavaminate synthase-like protein [Armillaria solidipes] | chr09_AA_01224 | uncharacterized protein ARMOST_22339 [Armillaria ostoyae] | 0.165723056 |
| chr08_AA_00687 | Clavaminate synthase-like protein [Armillaria solidipes] | chr09_AA_01225 | NAD(P)-binding protein [Cylindrobasidium torrendii FP15055 ss-10] | 0.159933362 |
| chr08_AA_00687 | Clavaminate synthase-like protein [Armillaria solidipes] | chr09_AA_01324 | uncharacterized protein ARMOST_03294 [Armillaria ostoyae] | 0.121310011 |
| chr08_AA_00687 | Clavaminate synthase-like protein [Armillaria solidipes] | chr09_AA_01410 | hypothetical protein CYLTODRAFT_489277 [Cylindrobasidium torrendii FP15055 ss-10] | 0.158818809 |
| chr08_AA_00687 | Clavaminate synthase-like protein [Armillaria solidipes] | chr10_AA_00134 | hypothetical protein CYLTODRAFT_349864 [Cylindrobasidium torrendii FP15055 ss-10] | 0.275076542 |
| chr08_AA_00687 | Clavaminate synthase-like protein [Armillaria solidipes] | chr10_AA_00164 | dehydrogenase E1 and transketolase domain-containing protein 1 [Cylindrobasidium torrendii FP15055 ss-10] | 0.29169015 |
| chr08_AA_00687 | Clavaminate synthase-like protein [Armillaria solidipes] | chr10_AA_00220 | hypothetical protein ARMGADRAFT_962646, partial [Armillaria gallica] | 0.27361884 |
| chr08_AA_00687 | Clavaminate synthase-like protein [Armillaria solidipes] | chr10_AA_00256 | uncharacterized protein ARMOST_03672 [Armillaria ostoyae] | 0.246114799 |
| chr08_AA_00687 | Clavaminate synthase-like protein [Armillaria solidipes] | chr10_AA_00270 | branched-chain alpha-keto acid dehydrogenase E1-alpha subunit [Cylindrobasidium torrendii FP15055 ss-10] | 0.281139789 |
| chr08_AA_00687 | Clavaminate synthase-like protein [Armillaria solidipes] | chr10_AA_00441 | alpha/beta-hydrolase [Armillaria solidipes] | 0.283278597 |
| chr08_AA_00687 | Clavaminate synthase-like protein [Armillaria solidipes] | chr10_AA_00499 | hypothetical protein WG66_16220 [Moniliophthora roreri] | 0.27111132 |
| chr08_AA_00687 | Clavaminate synthase-like protein [Armillaria solidipes] | chr10_AA_00517 | hypothetical protein CYLTODRAFT_256211 [Cylindrobasidium torrendii FP15055 ss-10] | 0.262343032 |
| chr08_AA_00687 | Clavaminate synthase-like protein [Armillaria solidipes] | chr10_AA_00651 | hypothetical protein ARMGADRAFT_897215, partial [Armillaria gallica] | 0.250606771 |
| chr08_AA_00687 | Clavaminate synthase-like protein [Armillaria solidipes] | chr10_AA_00662 | Ureohydrolase [Armillaria gallica] | 0.281158058 |
| chr08_AA_00687 | Clavaminate synthase-like protein [Armillaria solidipes] | chr10_AA_00693 | hypothetical protein ARMSODRAFT_947720 [Armillaria solidipes] | 0.291655973 |
| chr08_AA_00687 | Clavaminate synthase-like protein [Armillaria solidipes] | chr10_AA_00733 | Zinc-regulated transporter 1 [Hypsizygus marmoreus] | 0.185662909 |
| chr08_AA_00687 | Clavaminate synthase-like protein [Armillaria solidipes] | chr10_AA_00735 | glycoside hydrolase family 31 protein [Cylindrobasidium torrendii FP15055 ss-10] | 0.261253409 |
| chr08_AA_00687 | Clavaminate synthase-like protein [Armillaria solidipes] | chr10_AA_00912 | hypothetical protein ARMSODRAFT_948409 [Armillaria solidipes] | 0.103241341 |
| chr08_AA_00687 | Clavaminate synthase-like protein [Armillaria solidipes] | chr10_AA_00944 | general substrate transporter [Cylindrobasidium torrendii FP15055 ss-10] | 0.278317761 |
| chr08_AA_00687 | Clavaminate synthase-like protein [Armillaria solidipes] | chr10_AA_01056 | hypothetical protein ARMGADRAFT_1020988 [Armillaria gallica] | 0.177768987 |
| chr08_AA_00687 | Clavaminate synthase-like protein [Armillaria solidipes] | chr10_AA_01063 | glycoside hydrolase family 43 protein [Amanita thiersii Skay4041] | 0.221432823 |
| chr08_AA_00687 | Clavaminate synthase-like protein [Armillaria solidipes] | chr10_AA_01143 | uncharacterized protein ARMOST_08409 [Armillaria ostoyae] | 0.254193847 |
| chr08_AA_00687 | Clavaminate synthase-like protein [Armillaria solidipes] | chr10_AA_01157 | glycoside hydrolase family 53 protein [Cylindrobasidium torrendii FP15055 ss-10] | 0.241734442 |
| chr08_AA_00687 | Clavaminate synthase-like protein [Armillaria solidipes] | chr10_AA_01201 | acyltransferase ChoActase/COT/CPT [Armillaria gallica] | 0.244570645 |
| chr08_AA_00687 | Clavaminate synthase-like protein [Armillaria solidipes] | chr10_AA_01213 | hypothetical protein JAAARDRAFT_210932 [Jaapia argillacea MUCL 33604] | 0.234257894 |
| chr08_AA_00687 | Clavaminate synthase-like protein [Armillaria solidipes] | chr10_AA_01231 | hypothetical protein ARMSODRAFT_1019532 [Armillaria solidipes] | 0.279093168 |
| chr08_AA_00687 | Clavaminate synthase-like protein [Armillaria solidipes] | chr10_AA_01309 | hypothetical protein PHLCEN_2v2282 [Phlebia centrifuga] | 0.15797543 |
| chr08_AA_00687 | Clavaminate synthase-like protein [Armillaria solidipes] | chr10_AA_01310 | DUF124-domain-containing protein [Cylindrobasidium torrendii FP15055 ss-10] | 0.106315566 |
| chr08_AA_00687 | Clavaminate synthase-like protein [Armillaria solidipes] | chr10_AA_01322 | putative laccase 5 [Flammulina velutipes] | 0.169782255 |
| chr08_AA_00687 | Clavaminate synthase-like protein [Armillaria solidipes] | chr10_AA_01341 | glucuronyl hydrolase [Armillaria solidipes] | 0.241386007 |
| chr08_AA_00687 | Clavaminate synthase-like protein [Armillaria solidipes] | chr10_AA_01576 | D-arabinitol 2-dehydrogenase [ribulose-forming] [Hypsizygus marmoreus] | 0.227053066 |
| chr08_AA_00687 | Clavaminate synthase-like protein [Armillaria solidipes] | chr10_AA_01664 | hypothetical protein CYLTODRAFT_404266 [Cylindrobasidium torrendii FP15055 ss-10] | 0.25769082 |
| chr08_AA_00687 | Clavaminate synthase-like protein [Armillaria solidipes] | chr11_AA_00042 | kinase-like protein [Cylindrobasidium torrendii FP15055 ss-10] | 0.295623638 |
| chr08_AA_00687 | Clavaminate synthase-like protein [Armillaria solidipes] | chr11_AA_00094 | homogentisate 1,2-dioxygenase [Armillaria solidipes] | 0.200473408 |
| chr08_AA_00687 | Clavaminate synthase-like protein [Armillaria solidipes] | chr11_AA_00103 | hypothetical protein M413DRAFT_370146 [Hebeloma cylindrosporum h7] | 0.279725287 |
| chr08_AA_00687 | Clavaminate synthase-like protein [Armillaria solidipes] | chr11_AA_00145 | hypothetical protein CYLTODRAFT_243491 [Cylindrobasidium torrendii FP15055 ss-10] | 0.187214279 |
| chr08_AA_00687 | Clavaminate synthase-like protein [Armillaria solidipes] | chr11_AA_00233 | hypothetical protein ARMGADRAFT_977028 [Armillaria gallica] | 0.193401278 |
| chr08_AA_00687 | Clavaminate synthase-like protein [Armillaria solidipes] | chr11_AA_00265 | FAD-linked oxidoreductase [Armillaria solidipes] | 0.271880225 |
| chr08_AA_00687 | Clavaminate synthase-like protein [Armillaria solidipes] | chr11_AA_00277 | uncharacterized protein ARMOST_10602 [Armillaria ostoyae] | 0.16953016 |
| chr08_AA_00687 | Clavaminate synthase-like protein [Armillaria solidipes] | chr11_AA_00499 | GroES-like protein [Armillaria solidipes] | 0.276685614 |
| chr08_AA_00687 | Clavaminate synthase-like protein [Armillaria solidipes] | chr11_AA_00504 | MFS general substrate transporter [Cylindrobasidium torrendii FP15055 ss-10] | 0.286085051 |
| chr08_AA_00687 | Clavaminate synthase-like protein [Armillaria solidipes] | chr11_AA_00581 | laccase [Flammulina velutipes] | 0.107916078 |
| chr08_AA_00687 | Clavaminate synthase-like protein [Armillaria solidipes] | chr11_AA_00606 | hypothetical protein CYLTODRAFT_418833 [Cylindrobasidium torrendii FP15055 ss-10] | 0.257138552 |
| chr08_AA_00687 | Clavaminate synthase-like protein [Armillaria solidipes] | chr11_AA_00638 | hypothetical protein ARMSODRAFT_953107 [Armillaria solidipes] | 0.214869276 |
| chr08_AA_00687 | Clavaminate synthase-like protein [Armillaria solidipes] | chr11_AA_00701 | uncharacterized protein ARMOST_05344 [Armillaria ostoyae] | 0.169295677 |
| chr08_AA_00687 | Clavaminate synthase-like protein [Armillaria solidipes] | chr11_AA_00719 | hypothetical protein ARMGADRAFT_1019784, partial [Armillaria gallica] | 0.185956123 |
| chr08_AA_00687 | Clavaminate synthase-like protein [Armillaria solidipes] | chr11_AA_00724 | WD40 repeat-like protein [Armillaria solidipes] | 0.326030328 |
| chr08_AA_00687 | Clavaminate synthase-like protein [Armillaria solidipes] | chr11_AA_00790 | uncharacterized protein ARMOST_05401 [Armillaria ostoyae] | 0.202210764 |
| chr08_AA_00687 | Clavaminate synthase-like protein [Armillaria solidipes] | chr11_AA_00878 | hypothetical protein CYLTODRAFT_430286 [Cylindrobasidium torrendii FP15055 ss-10] | 0.283463434 |
| chr08_AA_00687 | Clavaminate synthase-like protein [Armillaria solidipes] | chr11_AA_00928 | putative malate synthase [Flammulina velutipes] | 0.265955548 |
| chr08_AA_00687 | Clavaminate synthase-like protein [Armillaria solidipes] | chr11_AA_00997 | Annexin [Cylindrobasidium torrendii FP15055 ss-10] | 0.295220868 |
| chr08_AA_00687 | Clavaminate synthase-like protein [Armillaria solidipes] | chr11_AA_01033 | glycoside hydrolase family 51 protein [Cylindrobasidium torrendii FP15055 ss-10] | 0.288178903 |
| chr08_AA_00687 | Clavaminate synthase-like protein [Armillaria solidipes] | chr11_AA_01042 | acetoin reductase family protein [Cylindrobasidium torrendii FP15055 ss-10] | 0.220001526 |
| chr08_AA_00687 | Clavaminate synthase-like protein [Armillaria solidipes] | chr11_AA_01046 | hypothetical protein CYLTODRAFT_428160 [Cylindrobasidium torrendii FP15055 ss-10] | 0.277481036 |
| chr08_AA_00687 | Clavaminate synthase-like protein [Armillaria solidipes] | chr11_AA_01097 | glycoside hydrolase family 3 protein [Cylindrobasidium torrendii FP15055 ss-10] | 0.225806282 |
| chr08_AA_00687 | Clavaminate synthase-like protein [Armillaria solidipes] | chr11_AA_01106 | related to stomatin [Armillaria ostoyae] | 0.263911004 |
| chr08_AA_00687 | Clavaminate synthase-like protein [Armillaria solidipes] | chr11_AA_01313 | hypothetical protein Hypma_000372 [Hypsizygus marmoreus] | 0.270450793 |
| chr08_AA_00687 | Clavaminate synthase-like protein [Armillaria solidipes] | chr11_AA_01324 | terpenoid synthase [Cylindrobasidium torrendii FP15055 ss-10] | 0.20977126 |
| chr08_AA_00687 | Clavaminate synthase-like protein [Armillaria solidipes] | chr11_AA_01456 | uncharacterized protein ARMOST_16541 [Armillaria ostoyae] | 0.275454737 |
| chr08_AA_00687 | Clavaminate synthase-like protein [Armillaria solidipes] | chr11_AA_01461 | ricin B-like lectin [Armillaria gallica] | 0.255872028 |
| chr08_AA_00687 | Clavaminate synthase-like protein [Armillaria solidipes] | chr11_AA_01510 | hypothetical protein ARMGADRAFT_1015707 [Armillaria gallica] | 0.179175707 |
| chr08_AA_00687 | Clavaminate synthase-like protein [Armillaria solidipes] | chr11_AA_01636 | hypothetical protein CYLTODRAFT_421880 [Cylindrobasidium torrendii FP15055 ss-10] | 0.28371303 |
| chr08_AA_00687 | Clavaminate synthase-like protein [Armillaria solidipes] | chr11_AA_01644 | hypothetical protein CYLTODRAFT_438918 [Cylindrobasidium torrendii FP15055 ss-10] | 0.200333327 |
| chr08_AA_00687 | Clavaminate synthase-like protein [Armillaria solidipes] | chr11_AA_01664 | hypothetical protein CYLTODRAFT_421876 [Cylindrobasidium torrendii FP15055 ss-10] | 0.249194236 |
| chr08_AA_00687 | Clavaminate synthase-like protein [Armillaria solidipes] | chr11_AA_01685 | 1-aminocyclopropane-1-carboxylate deaminase [Armillaria solidipes] | 0.256699556 |
| chr08_AA_00687 | Clavaminate synthase-like protein [Armillaria solidipes] | chr11_AA_01686 | hypothetical protein CYLTODRAFT_348076 [Cylindrobasidium torrendii FP15055 ss-10] | 0.208079492 |
| chr08_AA_00687 | Clavaminate synthase-like protein [Armillaria solidipes] | chr11_AA_01711 | hypothetical protein ARMGADRAFT_1046867 [Armillaria gallica] | 0.297438568 |
| chr08_AA_00687 | Clavaminate synthase-like protein [Armillaria solidipes] | chr11_AA_01731 | benzoquinone reductase [Cylindrobasidium torrendii FP15055 ss-10] | 0.143726629 |
| chr08_AA_00687 | Clavaminate synthase-like protein [Armillaria solidipes] | chr11_AA_01732 | glycoside hydrolase family 76 protein [Cylindrobasidium torrendii FP15055 ss-10] | 0.282261579 |
| chr08_AA_00687 | Clavaminate synthase-like protein [Armillaria solidipes] | chr11_AA_01733 | glycoside hydrolase family 76 protein [Cylindrobasidium torrendii FP15055 ss-10] | 0.28098972 |
| chr11_AA_01711 | hypothetical protein ARMGADRAFT_1046867 [Armillaria gallica] | chr11_AA_01731 | benzoquinone reductase [Cylindrobasidium torrendii FP15055 ss-10] | 0.14563098 |
| chr11_AA_01711 | hypothetical protein ARMGADRAFT_1046867 [Armillaria gallica] | chr11_AA_01732 | glycoside hydrolase family 76 protein [Cylindrobasidium torrendii FP15055 ss-10] | 0.231058976 |
| chr11_AA_01711 | hypothetical protein ARMGADRAFT_1046867 [Armillaria gallica] | chr11_AA_01733 | glycoside hydrolase family 76 protein [Cylindrobasidium torrendii FP15055 ss-10] | 0.23701906 |
| chr06_AA_00738 | pyridoxal phosphate-dependent enzyme, beta subunit [Cylindrobasidium torrendii FP15055 ss-10] | chr06_AA_00949 | hypothetical protein CYLTODRAFT_427642 [Cylindrobasidium torrendii FP15055 ss-10] | 0.209657615 |
| chr06_AA_00738 | pyridoxal phosphate-dependent enzyme, beta subunit [Cylindrobasidium torrendii FP15055 ss-10] | chr06_AA_01031 | glycoside hydrolase family 43 protein [Periconia macrospinosa] | 0.143280005 |
| chr06_AA_00738 | pyridoxal phosphate-dependent enzyme, beta subunit [Cylindrobasidium torrendii FP15055 ss-10] | chr07_AA_00098 | hypothetical protein ARMGADRAFT_926019 [Armillaria gallica] | 0.113984376 |
| chr06_AA_00738 | pyridoxal phosphate-dependent enzyme, beta subunit [Cylindrobasidium torrendii FP15055 ss-10] | chr07_AA_00156 | uncharacterized protein ARMOST_00326 [Armillaria ostoyae] | 0.24098164 |
| chr06_AA_00738 | pyridoxal phosphate-dependent enzyme, beta subunit [Cylindrobasidium torrendii FP15055 ss-10] | chr07_AA_00159 | FAD-binding domain-containing protein [Armillaria solidipes] | 0.211906711 |
| chr06_AA_00738 | pyridoxal phosphate-dependent enzyme, beta subunit [Cylindrobasidium torrendii FP15055 ss-10] | chr07_AA_00281 | NAD(P)-binding protein [Armillaria gallica] | 0.273470203 |
| chr06_AA_00738 | pyridoxal phosphate-dependent enzyme, beta subunit [Cylindrobasidium torrendii FP15055 ss-10] | chr07_AA_00380 | ectomycorrhiza-regulated esterase [Armillaria solidipes] | 0.255561049 |
| chr06_AA_00738 | pyridoxal phosphate-dependent enzyme, beta subunit [Cylindrobasidium torrendii FP15055 ss-10] | chr07_AA_00414 | MFS general substrate transporter [Armillaria gallica] | 0.246642194 |
| chr06_AA_00738 | pyridoxal phosphate-dependent enzyme, beta subunit [Cylindrobasidium torrendii FP15055 ss-10] | chr07_AA_00419 | hypothetical protein CYLTODRAFT_277105 [Cylindrobasidium torrendii FP15055 ss-10] | 0.177610127 |
| chr06_AA_00738 | pyridoxal phosphate-dependent enzyme, beta subunit [Cylindrobasidium torrendii FP15055 ss-10] | chr07_AA_00464 | cytochrome P450 [Cylindrobasidium torrendii FP15055 ss-10] | 0.225934219 |
| chr06_AA_00738 | pyridoxal phosphate-dependent enzyme, beta subunit [Cylindrobasidium torrendii FP15055 ss-10] | chr07_AA_00560 | hypothetical protein CYLTODRAFT_416229 [Cylindrobasidium torrendii FP15055 ss-10] | 0.222379946 |
| chr06_AA_00738 | pyridoxal phosphate-dependent enzyme, beta subunit [Cylindrobasidium torrendii FP15055 ss-10] | chr07_AA_00561 | hypothetical protein ARMGADRAFT_1031622 [Armillaria gallica] | 0.211653187 |
| chr06_AA_00738 | pyridoxal phosphate-dependent enzyme, beta subunit [Cylindrobasidium torrendii FP15055 ss-10] | chr07_AA_00567 | hypothetical protein CYLTODRAFT_406012 [Cylindrobasidium torrendii FP15055 ss-10] | 0.181339886 |
| chr06_AA_00738 | pyridoxal phosphate-dependent enzyme, beta subunit [Cylindrobasidium torrendii FP15055 ss-10] | chr07_AA_00601 | hypothetical protein GYMLUDRAFT_71620 [Gymnopus luxurians FD-317 M1] | 0.200303268 |
| chr06_AA_00738 | pyridoxal phosphate-dependent enzyme, beta subunit [Cylindrobasidium torrendii FP15055 ss-10] | chr07_AA_00617 | NAD-dependent formate dehydrogenase [Moniliophthora perniciosa] | 0.202380533 |
| chr06_AA_00738 | pyridoxal phosphate-dependent enzyme, beta subunit [Cylindrobasidium torrendii FP15055 ss-10] | chr07_AA_00645 | aldehyde dehydrogenase [Armillaria gallica] | 0.242047891 |
| chr06_AA_00738 | pyridoxal phosphate-dependent enzyme, beta subunit [Cylindrobasidium torrendii FP15055 ss-10] | chr07_AA_00675 | multidrug resistance protein 1 [Armillaria gallica] | 0.114088988 |
| chr06_AA_00738 | pyridoxal phosphate-dependent enzyme, beta subunit [Cylindrobasidium torrendii FP15055 ss-10] | chr07_AA_00703 | potassium/sodium eff [Cylindrobasidium torrendii FP15055 ss-10] | 0.249983421 |
| chr06_AA_00738 | pyridoxal phosphate-dependent enzyme, beta subunit [Cylindrobasidium torrendii FP15055 ss-10] | chr07_AA_00722 | hypothetical protein K443DRAFT_93998 [Laccaria amethystina LaAM-08-1] | 0.258392948 |
| chr06_AA_00738 | pyridoxal phosphate-dependent enzyme, beta subunit [Cylindrobasidium torrendii FP15055 ss-10] | chr07_AA_00739 | hypothetical protein CYLTODRAFT_417357 [Cylindrobasidium torrendii FP15055 ss-10] | 0.217822908 |
| chr06_AA_00738 | pyridoxal phosphate-dependent enzyme, beta subunit [Cylindrobasidium torrendii FP15055 ss-10] | chr07_AA_00756 | SAICAR synthase-like protein [Cylindrobasidium torrendii FP15055 ss-10] | 0.120070198 |
| chr06_AA_00738 | pyridoxal phosphate-dependent enzyme, beta subunit [Cylindrobasidium torrendii FP15055 ss-10] | chr07_AA_00826 | Metallo-hydrolase/oxidoreductase [Armillaria gallica] | 0.175407201 |
| chr06_AA_00738 | pyridoxal phosphate-dependent enzyme, beta subunit [Cylindrobasidium torrendii FP15055 ss-10] | chr07_AA_00827 | hypothetical protein LENED_003150 [Lentinula edodes] | 0.155974237 |
| chr06_AA_00738 | pyridoxal phosphate-dependent enzyme, beta subunit [Cylindrobasidium torrendii FP15055 ss-10] | chr07_AA_00829 | general substrate transporter [Cylindrobasidium torrendii FP15055 ss-10] | 0.183924172 |
| chr06_AA_00738 | pyridoxal phosphate-dependent enzyme, beta subunit [Cylindrobasidium torrendii FP15055 ss-10] | chr07_AA_00907 | uncharacterized protein ARMOST_00985 [Armillaria ostoyae] | 0.179345203 |
| chr06_AA_00738 | pyridoxal phosphate-dependent enzyme, beta subunit [Cylindrobasidium torrendii FP15055 ss-10] | chr07_AA_00951 | acyl-CoA dehydrogenase domain-containing protein [Cylindrobasidium torrendii FP15055 ss-10] | 0.21934958 |
| chr06_AA_00738 | pyridoxal phosphate-dependent enzyme, beta subunit [Cylindrobasidium torrendii FP15055 ss-10] | chr07_AA_01020 | extracellular GDSL-like lipase/acylhydrolase [Glonium stellatum] | 0.177975712 |
| chr06_AA_00738 | pyridoxal phosphate-dependent enzyme, beta subunit [Cylindrobasidium torrendii FP15055 ss-10] | chr07_AA_01021 | hypothetical protein PLEOSDRAFT_1096996 [Pleurotus ostreatus PC15] | 0.158025296 |
| chr06_AA_00738 | pyridoxal phosphate-dependent enzyme, beta subunit [Cylindrobasidium torrendii FP15055 ss-10] | chr07_AA_01044 | uncharacterized protein ARMOST_19175 [Armillaria ostoyae] | 0.216079523 |
| chr06_AA_00738 | pyridoxal phosphate-dependent enzyme, beta subunit [Cylindrobasidium torrendii FP15055 ss-10] | chr07_AA_01051 | MFS general substrate transporter [Cylindrobasidium torrendii FP15055 ss-10] | 0.164212957 |
| chr06_AA_00738 | pyridoxal phosphate-dependent enzyme, beta subunit [Cylindrobasidium torrendii FP15055 ss-10] | chr07_AA_01089 | cysteine proteinase [Cylindrobasidium torrendii FP15055 ss-10] | 0.291878433 |
| chr06_AA_00738 | pyridoxal phosphate-dependent enzyme, beta subunit [Cylindrobasidium torrendii FP15055 ss-10] | chr08_AA_00018 | putative peroxiredoxin Q [Flammulina velutipes] | 0.233491045 |
| chr06_AA_00738 | pyridoxal phosphate-dependent enzyme, beta subunit [Cylindrobasidium torrendii FP15055 ss-10] | chr08_AA_00106 | hypothetical protein ARMGADRAFT_100162 [Armillaria gallica] | 0.105989982 |
| chr06_AA_00738 | pyridoxal phosphate-dependent enzyme, beta subunit [Cylindrobasidium torrendii FP15055 ss-10] | chr08_AA_00260 | CDF-like metal transporter [Armillaria solidipes] | 0.212280685 |
| chr06_AA_00738 | pyridoxal phosphate-dependent enzyme, beta subunit [Cylindrobasidium torrendii FP15055 ss-10] | chr08_AA_00287 | hypothetical protein PLICRDRAFT_118622 [Plicaturopsis crispa FD-325 SS-3] | 0.198459662 |
| chr06_AA_00738 | pyridoxal phosphate-dependent enzyme, beta subunit [Cylindrobasidium torrendii FP15055 ss-10] | chr08_AA_00338 | DUF1479-domain-containing protein [Cylindrobasidium torrendii FP15055 ss-10] | 0.24221986 |
| chr06_AA_00738 | pyridoxal phosphate-dependent enzyme, beta subunit [Cylindrobasidium torrendii FP15055 ss-10] | chr08_AA_00413 | hypothetical protein ARMGADRAFT_391312 [Armillaria gallica] | 0.239319051 |
| chr06_AA_00738 | pyridoxal phosphate-dependent enzyme, beta subunit [Cylindrobasidium torrendii FP15055 ss-10] | chr08_AA_00496 | amidase signature enzyme [Sanghuangporus baumii] | 0.138046275 |
| chr06_AA_00738 | pyridoxal phosphate-dependent enzyme, beta subunit [Cylindrobasidium torrendii FP15055 ss-10] | chr08_AA_00501 | hypothetical protein CYLTODRAFT_416851 [Cylindrobasidium torrendii FP15055 ss-10] | 0.237417768 |
| chr06_AA_00738 | pyridoxal phosphate-dependent enzyme, beta subunit [Cylindrobasidium torrendii FP15055 ss-10] | chr08_AA_00542 | hypothetical protein ARMGADRAFT_1159894 [Armillaria gallica] | 0.272499756 |
| chr06_AA_00738 | pyridoxal phosphate-dependent enzyme, beta subunit [Cylindrobasidium torrendii FP15055 ss-10] | chr08_AA_00621 | hypothetical protein ARMGADRAFT_982650 [Armillaria gallica] | 0.226417067 |
| chr06_AA_00738 | pyridoxal phosphate-dependent enzyme, beta subunit [Cylindrobasidium torrendii FP15055 ss-10] | chr08_AA_00651 | related to isp4-oligopeptide transporter [Armillaria ostoyae] | 0.159503115 |
| chr06_AA_00738 | pyridoxal phosphate-dependent enzyme, beta subunit [Cylindrobasidium torrendii FP15055 ss-10] | chr08_AA_00687 | Clavaminate synthase-like protein [Armillaria solidipes] | 0.292595437 |
| chr06_AA_00738 | pyridoxal phosphate-dependent enzyme, beta subunit [Cylindrobasidium torrendii FP15055 ss-10] | chr08_AA_00856 | cytochrome p450 [Moniliophthora roreri MCA 2997] | 0.222830665 |
| chr06_AA_00738 | pyridoxal phosphate-dependent enzyme, beta subunit [Cylindrobasidium torrendii FP15055 ss-10] | chr08_AA_00858 | cytochrome P450 [Cylindrobasidium torrendii FP15055 ss-10] | 0.25457094 |
| chr06_AA_00738 | pyridoxal phosphate-dependent enzyme, beta subunit [Cylindrobasidium torrendii FP15055 ss-10] | chr08_AA_00936 | MFS general substrate transporter [Armillaria gallica] | 0.180317263 |
| chr06_AA_00738 | pyridoxal phosphate-dependent enzyme, beta subunit [Cylindrobasidium torrendii FP15055 ss-10] | chr08_AA_00948 | probable glutamate dehydrogenase, NAD(+)-specific [Armillaria ostoyae] | 0.187677898 |
| chr06_AA_00738 | pyridoxal phosphate-dependent enzyme, beta subunit [Cylindrobasidium torrendii FP15055 ss-10] | chr08_AA_00984 | cytochrome P450 monooxygenase pc-bph [Armillaria gallica] | 0.18597182 |
| chr06_AA_00738 | pyridoxal phosphate-dependent enzyme, beta subunit [Cylindrobasidium torrendii FP15055 ss-10] | chr08_AA_01027 | hypothetical protein CYLTODRAFT_357890 [Cylindrobasidium torrendii FP15055 ss-10] | 0.253973016 |
| chr06_AA_00738 | pyridoxal phosphate-dependent enzyme, beta subunit [Cylindrobasidium torrendii FP15055 ss-10] | chr08_AA_01046 | MFS general substrate transporter [Armillaria gallica] | 0.208382589 |
| chr06_AA_00738 | pyridoxal phosphate-dependent enzyme, beta subunit [Cylindrobasidium torrendii FP15055 ss-10] | chr08_AA_01069 | alpha/beta-hydrolase [Armillaria solidipes] | 0.137571114 |
| chr06_AA_00738 | pyridoxal phosphate-dependent enzyme, beta subunit [Cylindrobasidium torrendii FP15055 ss-10] | chr08_AA_01077 | GroES-like protein [Cylindrobasidium torrendii FP15055 ss-10] | 0.156503909 |
| chr06_AA_00738 | pyridoxal phosphate-dependent enzyme, beta subunit [Cylindrobasidium torrendii FP15055 ss-10] | chr08_AA_01081 | uncharacterized protein ARMOST_20173 [Armillaria ostoyae] | 0.199690093 |
| chr06_AA_00738 | pyridoxal phosphate-dependent enzyme, beta subunit [Cylindrobasidium torrendii FP15055 ss-10] | chr08_AA_01091 | uncharacterized protein ARMOST_03386 [Armillaria ostoyae] | 0.233425952 |
| chr06_AA_00738 | pyridoxal phosphate-dependent enzyme, beta subunit [Cylindrobasidium torrendii FP15055 ss-10] | chr08_AA_01097 | hypothetical protein GYMLUDRAFT_32937 [Gymnopus luxurians FD-317 M1] | 0.181614262 |
| chr06_AA_00738 | pyridoxal phosphate-dependent enzyme, beta subunit [Cylindrobasidium torrendii FP15055 ss-10] | chr08_AA_01155 | related to 3-oxoacyl CoA thiolase [Armillaria ostoyae] | 0.183774736 |
| chr06_AA_00738 | pyridoxal phosphate-dependent enzyme, beta subunit [Cylindrobasidium torrendii FP15055 ss-10] | chr08_AA_01172 | carbohydrate esterase family 1 protein, partial [Cylindrobasidium torrendii FP15055 ss-10] | 0.149214977 |
| chr06_AA_00738 | pyridoxal phosphate-dependent enzyme, beta subunit [Cylindrobasidium torrendii FP15055 ss-10] | chr08_AA_01176 | hypothetical protein HYPSUDRAFT_61393 [Hypholoma sublateritium FD-334 SS-4] | 0.228118941 |
| chr06_AA_00738 | pyridoxal phosphate-dependent enzyme, beta subunit [Cylindrobasidium torrendii FP15055 ss-10] | chr08_AA_01257 | hypothetical protein ARMSODRAFT_949486 [Armillaria solidipes] | 0.255992144 |
| chr06_AA_00738 | pyridoxal phosphate-dependent enzyme, beta subunit [Cylindrobasidium torrendii FP15055 ss-10] | chr08_AA_01259 | Aldo/keto reductase [Armillaria gallica] | 0.259568976 |
| chr06_AA_00738 | pyridoxal phosphate-dependent enzyme, beta subunit [Cylindrobasidium torrendii FP15055 ss-10] | chr09_AA_00036 | hypothetical protein ARMGADRAFT_1070289 [Armillaria gallica] | 0.146236232 |
| chr06_AA_00738 | pyridoxal phosphate-dependent enzyme, beta subunit [Cylindrobasidium torrendii FP15055 ss-10] | chr09_AA_00051 | GMC oxidoreductase [Cylindrobasidium torrendii FP15055 ss-10] | 0.252477981 |
| chr06_AA_00738 | pyridoxal phosphate-dependent enzyme, beta subunit [Cylindrobasidium torrendii FP15055 ss-10] | chr09_AA_00052 | GMC oxidoreductase [Cylindrobasidium torrendii FP15055 ss-10] | 0.243564278 |
| chr06_AA_00738 | pyridoxal phosphate-dependent enzyme, beta subunit [Cylindrobasidium torrendii FP15055 ss-10] | chr09_AA_00095 | hypothetical protein CYLTODRAFT_422329 [Cylindrobasidium torrendii FP15055 ss-10] | 0.244751407 |
| chr06_AA_00738 | pyridoxal phosphate-dependent enzyme, beta subunit [Cylindrobasidium torrendii FP15055 ss-10] | chr09_AA_00100 | hypothetical protein K503DRAFT_238266 [Rhizopogon vinicolor AM-OR11-026] | 0.231291265 |
| chr06_AA_00738 | pyridoxal phosphate-dependent enzyme, beta subunit [Cylindrobasidium torrendii FP15055 ss-10] | chr09_AA_00139 | hypothetical protein CYLTODRAFT_424555 [Cylindrobasidium torrendii FP15055 ss-10] | 0.143721996 |
| chr06_AA_00738 | pyridoxal phosphate-dependent enzyme, beta subunit [Cylindrobasidium torrendii FP15055 ss-10] | chr09_AA_00162 | hypothetical protein ARMGADRAFT_961910 [Armillaria gallica] | 0.252028521 |
| chr06_AA_00738 | pyridoxal phosphate-dependent enzyme, beta subunit [Cylindrobasidium torrendii FP15055 ss-10] | chr09_AA_00190 | putative exo-beta-1,3-glucanase [Flammulina velutipes] | 0.142971933 |
| chr06_AA_00738 | pyridoxal phosphate-dependent enzyme, beta subunit [Cylindrobasidium torrendii FP15055 ss-10] | chr09_AA_00191 | exo-beta-1,3-glucanase [Armillaria solidipes] | 0.14308062 |
| chr06_AA_00738 | pyridoxal phosphate-dependent enzyme, beta subunit [Cylindrobasidium torrendii FP15055 ss-10] | chr09_AA_00229 | succinyl-CoA:3-ketoacid-coenzyme A transferase [Cylindrobasidium torrendii FP15055 ss-10] | 0.213998737 |
| chr06_AA_00738 | pyridoxal phosphate-dependent enzyme, beta subunit [Cylindrobasidium torrendii FP15055 ss-10] | chr09_AA_00241 | SNF2 chromatin remodeling protein [Armillaria solidipes] | 0.259339078 |
| chr06_AA_00738 | pyridoxal phosphate-dependent enzyme, beta subunit [Cylindrobasidium torrendii FP15055 ss-10] | chr09_AA_00312 | putative beta-glucosidase [Flammulina velutipes] | 0.183943408 |
| chr06_AA_00738 | pyridoxal phosphate-dependent enzyme, beta subunit [Cylindrobasidium torrendii FP15055 ss-10] | chr09_AA_00406 | uncharacterized protein ARMOST_17180 [Armillaria ostoyae] | 0.208186768 |
| chr06_AA_00738 | pyridoxal phosphate-dependent enzyme, beta subunit [Cylindrobasidium torrendii FP15055 ss-10] | chr09_AA_00518 | AAA-domain-containing protein [Cylindrobasidium torrendii FP15055 ss-10] | 0.246845022 |
| chr06_AA_00738 | pyridoxal phosphate-dependent enzyme, beta subunit [Cylindrobasidium torrendii FP15055 ss-10] | chr09_AA_00551 | hypothetical protein CYLTODRAFT_392634 [Cylindrobasidium torrendii FP15055 ss-10] | 0.133706367 |
| chr06_AA_00738 | pyridoxal phosphate-dependent enzyme, beta subunit [Cylindrobasidium torrendii FP15055 ss-10] | chr09_AA_00680 | hypothetical protein CYLTODRAFT_395198 [Cylindrobasidium torrendii FP15055 ss-10] | 0.255951573 |
| chr06_AA_00738 | pyridoxal phosphate-dependent enzyme, beta subunit [Cylindrobasidium torrendii FP15055 ss-10] | chr09_AA_00719 | hypothetical protein PLEOSDRAFT_1093927 [Pleurotus ostreatus PC15] | 0.213115195 |
| chr06_AA_00738 | pyridoxal phosphate-dependent enzyme, beta subunit [Cylindrobasidium torrendii FP15055 ss-10] | chr09_AA_00762 | transketolase [Armillaria solidipes] | 0.122159627 |
| chr06_AA_00738 | pyridoxal phosphate-dependent enzyme, beta subunit [Cylindrobasidium torrendii FP15055 ss-10] | chr09_AA_00816 | mandelate racemase muconate lactonizing enzyme family protein [Cylindrobasidium torrendii FP15055 ss-10] | 0.129968313 |
| chr06_AA_00738 | pyridoxal phosphate-dependent enzyme, beta subunit [Cylindrobasidium torrendii FP15055 ss-10] | chr09_AA_00838 | cytochrome P450 [Armillaria gallica] | 0.258109125 |
| chr06_AA_00738 | pyridoxal phosphate-dependent enzyme, beta subunit [Cylindrobasidium torrendii FP15055 ss-10] | chr09_AA_00892 | hypothetical protein CYLTODRAFT_431446 [Cylindrobasidium torrendii FP15055 ss-10] | 0.237932718 |
| chr06_AA_00738 | pyridoxal phosphate-dependent enzyme, beta subunit [Cylindrobasidium torrendii FP15055 ss-10] | chr09_AA_00955 | putative endo-1,4-beta-xylanase precursor [Flammulina velutipes] | 0.169685463 |
| chr06_AA_00738 | pyridoxal phosphate-dependent enzyme, beta subunit [Cylindrobasidium torrendii FP15055 ss-10] | chr09_AA_01058 | hypothetical protein ARMSODRAFT_976614 [Armillaria solidipes] | 0.24346036 |
| chr06_AA_00738 | pyridoxal phosphate-dependent enzyme, beta subunit [Cylindrobasidium torrendii FP15055 ss-10] | chr09_AA_01099 | general substrate transporter [Armillaria gallica] | 0.174919269 |
| chr06_AA_00738 | pyridoxal phosphate-dependent enzyme, beta subunit [Cylindrobasidium torrendii FP15055 ss-10] | chr09_AA_01103 | Metallo-dependent phosphatase [Armillaria solidipes] | 0.229989942 |
| chr06_AA_00738 | pyridoxal phosphate-dependent enzyme, beta subunit [Cylindrobasidium torrendii FP15055 ss-10] | chr09_AA_01106 | multidrug transporter [Cylindrobasidium torrendii FP15055 ss-10] | 0.111275323 |
| chr06_AA_00738 | pyridoxal phosphate-dependent enzyme, beta subunit [Cylindrobasidium torrendii FP15055 ss-10] | chr09_AA_01141 | hypothetical protein ARMGADRAFT_1063089 [Armillaria gallica] | 0.237860327 |
| chr06_AA_00738 | pyridoxal phosphate-dependent enzyme, beta subunit [Cylindrobasidium torrendii FP15055 ss-10] | chr09_AA_01190 | potassium/sodium eff [Cylindrobasidium torrendii FP15055 ss-10] | 0.252058024 |
| chr06_AA_00738 | pyridoxal phosphate-dependent enzyme, beta subunit [Cylindrobasidium torrendii FP15055 ss-10] | chr09_AA_01214 | hypothetical protein CYLTODRAFT_417942 [Cylindrobasidium torrendii FP15055 ss-10] | 0.239824483 |
| chr06_AA_00738 | pyridoxal phosphate-dependent enzyme, beta subunit [Cylindrobasidium torrendii FP15055 ss-10] | chr09_AA_01224 | uncharacterized protein ARMOST_22339 [Armillaria ostoyae] | 0.125502946 |
| chr06_AA_00738 | pyridoxal phosphate-dependent enzyme, beta subunit [Cylindrobasidium torrendii FP15055 ss-10] | chr09_AA_01225 | NAD(P)-binding protein [Cylindrobasidium torrendii FP15055 ss-10] | 0.160110644 |
| chr06_AA_00738 | pyridoxal phosphate-dependent enzyme, beta subunit [Cylindrobasidium torrendii FP15055 ss-10] | chr09_AA_01324 | uncharacterized protein ARMOST_03294 [Armillaria ostoyae] | 0.103461725 |
| chr06_AA_00738 | pyridoxal phosphate-dependent enzyme, beta subunit [Cylindrobasidium torrendii FP15055 ss-10] | chr09_AA_01410 | hypothetical protein CYLTODRAFT_489277 [Cylindrobasidium torrendii FP15055 ss-10] | 0.21768577 |
| chr06_AA_00738 | pyridoxal phosphate-dependent enzyme, beta subunit [Cylindrobasidium torrendii FP15055 ss-10] | chr10_AA_00134 | hypothetical protein CYLTODRAFT_349864 [Cylindrobasidium torrendii FP15055 ss-10] | 0.218688374 |
| chr06_AA_00738 | pyridoxal phosphate-dependent enzyme, beta subunit [Cylindrobasidium torrendii FP15055 ss-10] | chr10_AA_00164 | dehydrogenase E1 and transketolase domain-containing protein 1 [Cylindrobasidium torrendii FP15055 ss-10] | 0.239286911 |
| chr06_AA_00738 | pyridoxal phosphate-dependent enzyme, beta subunit [Cylindrobasidium torrendii FP15055 ss-10] | chr10_AA_00220 | hypothetical protein ARMGADRAFT_962646, partial [Armillaria gallica] | 0.246806937 |
| chr06_AA_00738 | pyridoxal phosphate-dependent enzyme, beta subunit [Cylindrobasidium torrendii FP15055 ss-10] | chr10_AA_00256 | uncharacterized protein ARMOST_03672 [Armillaria ostoyae] | 0.226700852 |
| chr06_AA_00738 | pyridoxal phosphate-dependent enzyme, beta subunit [Cylindrobasidium torrendii FP15055 ss-10] | chr10_AA_00270 | branched-chain alpha-keto acid dehydrogenase E1-alpha subunit [Cylindrobasidium torrendii FP15055 ss-10] | 0.269348911 |
| chr06_AA_00738 | pyridoxal phosphate-dependent enzyme, beta subunit [Cylindrobasidium torrendii FP15055 ss-10] | chr10_AA_00441 | alpha/beta-hydrolase [Armillaria solidipes] | 0.255729985 |
| chr06_AA_00738 | pyridoxal phosphate-dependent enzyme, beta subunit [Cylindrobasidium torrendii FP15055 ss-10] | chr10_AA_00499 | hypothetical protein WG66_16220 [Moniliophthora roreri] | 0.230101537 |
| chr06_AA_00738 | pyridoxal phosphate-dependent enzyme, beta subunit [Cylindrobasidium torrendii FP15055 ss-10] | chr10_AA_00517 | hypothetical protein CYLTODRAFT_256211 [Cylindrobasidium torrendii FP15055 ss-10] | 0.233912851 |
| chr06_AA_00738 | pyridoxal phosphate-dependent enzyme, beta subunit [Cylindrobasidium torrendii FP15055 ss-10] | chr10_AA_00651 | hypothetical protein ARMGADRAFT_897215, partial [Armillaria gallica] | 0.232522039 |
| chr06_AA_00738 | pyridoxal phosphate-dependent enzyme, beta subunit [Cylindrobasidium torrendii FP15055 ss-10] | chr10_AA_00662 | Ureohydrolase [Armillaria gallica] | 0.27564701 |
| chr06_AA_00738 | pyridoxal phosphate-dependent enzyme, beta subunit [Cylindrobasidium torrendii FP15055 ss-10] | chr10_AA_00693 | hypothetical protein ARMSODRAFT_947720 [Armillaria solidipes] | 0.260739061 |
| chr06_AA_00738 | pyridoxal phosphate-dependent enzyme, beta subunit [Cylindrobasidium torrendii FP15055 ss-10] | chr10_AA_00733 | Zinc-regulated transporter 1 [Hypsizygus marmoreus] | 0.120347079 |
| chr06_AA_00738 | pyridoxal phosphate-dependent enzyme, beta subunit [Cylindrobasidium torrendii FP15055 ss-10] | chr10_AA_00735 | glycoside hydrolase family 31 protein [Cylindrobasidium torrendii FP15055 ss-10] | 0.219198493 |
| chr06_AA_00738 | pyridoxal phosphate-dependent enzyme, beta subunit [Cylindrobasidium torrendii FP15055 ss-10] | chr10_AA_00912 | hypothetical protein ARMSODRAFT_948409 [Armillaria solidipes] | 0.105197716 |
| chr06_AA_00738 | pyridoxal phosphate-dependent enzyme, beta subunit [Cylindrobasidium torrendii FP15055 ss-10] | chr10_AA_00944 | general substrate transporter [Cylindrobasidium torrendii FP15055 ss-10] | 0.204645379 |
| chr06_AA_00738 | pyridoxal phosphate-dependent enzyme, beta subunit [Cylindrobasidium torrendii FP15055 ss-10] | chr10_AA_01056 | hypothetical protein ARMGADRAFT_1020988 [Armillaria gallica] | 0.182250371 |
| chr06_AA_00738 | pyridoxal phosphate-dependent enzyme, beta subunit [Cylindrobasidium torrendii FP15055 ss-10] | chr10_AA_01063 | glycoside hydrolase family 43 protein [Amanita thiersii Skay4041] | 0.171337142 |
| chr06_AA_00738 | pyridoxal phosphate-dependent enzyme, beta subunit [Cylindrobasidium torrendii FP15055 ss-10] | chr10_AA_01143 | uncharacterized protein ARMOST_08409 [Armillaria ostoyae] | 0.266250383 |
| chr06_AA_00738 | pyridoxal phosphate-dependent enzyme, beta subunit [Cylindrobasidium torrendii FP15055 ss-10] | chr10_AA_01157 | glycoside hydrolase family 53 protein [Cylindrobasidium torrendii FP15055 ss-10] | 0.216415743 |
| chr06_AA_00738 | pyridoxal phosphate-dependent enzyme, beta subunit [Cylindrobasidium torrendii FP15055 ss-10] | chr10_AA_01201 | acyltransferase ChoActase/COT/CPT [Armillaria gallica] | 0.169349593 |
| chr06_AA_00738 | pyridoxal phosphate-dependent enzyme, beta subunit [Cylindrobasidium torrendii FP15055 ss-10] | chr10_AA_01213 | hypothetical protein JAAARDRAFT_210932 [Jaapia argillacea MUCL 33604] | 0.25262569 |
| chr06_AA_00738 | pyridoxal phosphate-dependent enzyme, beta subunit [Cylindrobasidium torrendii FP15055 ss-10] | chr10_AA_01231 | hypothetical protein ARMSODRAFT_1019532 [Armillaria solidipes] | 0.249124876 |
| chr06_AA_00738 | pyridoxal phosphate-dependent enzyme, beta subunit [Cylindrobasidium torrendii FP15055 ss-10] | chr10_AA_01309 | hypothetical protein PHLCEN_2v2282 [Phlebia centrifuga] | 0.163696958 |
| chr06_AA_00738 | pyridoxal phosphate-dependent enzyme, beta subunit [Cylindrobasidium torrendii FP15055 ss-10] | chr10_AA_01310 | DUF124-domain-containing protein [Cylindrobasidium torrendii FP15055 ss-10] | 0.111739112 |
| chr06_AA_00738 | pyridoxal phosphate-dependent enzyme, beta subunit [Cylindrobasidium torrendii FP15055 ss-10] | chr10_AA_01322 | putative laccase 5 [Flammulina velutipes] | 0.132918287 |
| chr06_AA_00738 | pyridoxal phosphate-dependent enzyme, beta subunit [Cylindrobasidium torrendii FP15055 ss-10] | chr10_AA_01341 | glucuronyl hydrolase [Armillaria solidipes] | 0.204405593 |
| chr06_AA_00738 | pyridoxal phosphate-dependent enzyme, beta subunit [Cylindrobasidium torrendii FP15055 ss-10] | chr10_AA_01390 | hypothetical protein CYLTODRAFT_414265 [Cylindrobasidium torrendii FP15055 ss-10] | 0.101849068 |
| chr06_AA_00738 | pyridoxal phosphate-dependent enzyme, beta subunit [Cylindrobasidium torrendii FP15055 ss-10] | chr10_AA_01576 | D-arabinitol 2-dehydrogenase [ribulose-forming] [Hypsizygus marmoreus] | 0.150350439 |
| chr06_AA_00738 | pyridoxal phosphate-dependent enzyme, beta subunit [Cylindrobasidium torrendii FP15055 ss-10] | chr10_AA_01664 | hypothetical protein CYLTODRAFT_404266 [Cylindrobasidium torrendii FP15055 ss-10] | 0.239224819 |
| chr06_AA_00738 | pyridoxal phosphate-dependent enzyme, beta subunit [Cylindrobasidium torrendii FP15055 ss-10] | chr11_AA_00042 | kinase-like protein [Cylindrobasidium torrendii FP15055 ss-10] | 0.227807261 |
| chr06_AA_00738 | pyridoxal phosphate-dependent enzyme, beta subunit [Cylindrobasidium torrendii FP15055 ss-10] | chr11_AA_00094 | homogentisate 1,2-dioxygenase [Armillaria solidipes] | 0.213432205 |
| chr06_AA_00738 | pyridoxal phosphate-dependent enzyme, beta subunit [Cylindrobasidium torrendii FP15055 ss-10] | chr11_AA_00103 | hypothetical protein M413DRAFT_370146 [Hebeloma cylindrosporum h7] | 0.22688012 |
| chr06_AA_00738 | pyridoxal phosphate-dependent enzyme, beta subunit [Cylindrobasidium torrendii FP15055 ss-10] | chr11_AA_00145 | hypothetical protein CYLTODRAFT_243491 [Cylindrobasidium torrendii FP15055 ss-10] | 0.113114872 |
| chr06_AA_00738 | pyridoxal phosphate-dependent enzyme, beta subunit [Cylindrobasidium torrendii FP15055 ss-10] | chr11_AA_00233 | hypothetical protein ARMGADRAFT_977028 [Armillaria gallica] | 0.219461175 |
| chr06_AA_00738 | pyridoxal phosphate-dependent enzyme, beta subunit [Cylindrobasidium torrendii FP15055 ss-10] | chr11_AA_00265 | FAD-linked oxidoreductase [Armillaria solidipes] | 0.220539422 |
| chr06_AA_00738 | pyridoxal phosphate-dependent enzyme, beta subunit [Cylindrobasidium torrendii FP15055 ss-10] | chr11_AA_00277 | uncharacterized protein ARMOST_10602 [Armillaria ostoyae] | 0.195751299 |
| chr06_AA_00738 | pyridoxal phosphate-dependent enzyme, beta subunit [Cylindrobasidium torrendii FP15055 ss-10] | chr11_AA_00499 | GroES-like protein [Armillaria solidipes] | 0.273670316 |
| chr06_AA_00738 | pyridoxal phosphate-dependent enzyme, beta subunit [Cylindrobasidium torrendii FP15055 ss-10] | chr11_AA_00504 | MFS general substrate transporter [Cylindrobasidium torrendii FP15055 ss-10] | 0.246516162 |
| chr06_AA_00738 | pyridoxal phosphate-dependent enzyme, beta subunit [Cylindrobasidium torrendii FP15055 ss-10] | chr11_AA_00606 | hypothetical protein CYLTODRAFT_418833 [Cylindrobasidium torrendii FP15055 ss-10] | 0.190282602 |
| chr06_AA_00738 | pyridoxal phosphate-dependent enzyme, beta subunit [Cylindrobasidium torrendii FP15055 ss-10] | chr11_AA_00638 | hypothetical protein ARMSODRAFT_953107 [Armillaria solidipes] | 0.216310324 |
| chr06_AA_00738 | pyridoxal phosphate-dependent enzyme, beta subunit [Cylindrobasidium torrendii FP15055 ss-10] | chr11_AA_00701 | uncharacterized protein ARMOST_05344 [Armillaria ostoyae] | 0.108206127 |
| chr06_AA_00738 | pyridoxal phosphate-dependent enzyme, beta subunit [Cylindrobasidium torrendii FP15055 ss-10] | chr11_AA_00719 | hypothetical protein ARMGADRAFT_1019784, partial [Armillaria gallica] | 0.199670253 |
| chr06_AA_00738 | pyridoxal phosphate-dependent enzyme, beta subunit [Cylindrobasidium torrendii FP15055 ss-10] | chr11_AA_00724 | WD40 repeat-like protein [Armillaria solidipes] | 0.316138045 |
| chr06_AA_00738 | pyridoxal phosphate-dependent enzyme, beta subunit [Cylindrobasidium torrendii FP15055 ss-10] | chr11_AA_00790 | uncharacterized protein ARMOST_05401 [Armillaria ostoyae] | 0.138522128 |
| chr06_AA_00738 | pyridoxal phosphate-dependent enzyme, beta subunit [Cylindrobasidium torrendii FP15055 ss-10] | chr11_AA_00878 | hypothetical protein CYLTODRAFT_430286 [Cylindrobasidium torrendii FP15055 ss-10] | 0.243668476 |
| chr06_AA_00738 | pyridoxal phosphate-dependent enzyme, beta subunit [Cylindrobasidium torrendii FP15055 ss-10] | chr11_AA_00928 | putative malate synthase [Flammulina velutipes] | 0.233035855 |
| chr06_AA_00738 | pyridoxal phosphate-dependent enzyme, beta subunit [Cylindrobasidium torrendii FP15055 ss-10] | chr11_AA_00997 | Annexin [Cylindrobasidium torrendii FP15055 ss-10] | 0.240691062 |
| chr06_AA_00738 | pyridoxal phosphate-dependent enzyme, beta subunit [Cylindrobasidium torrendii FP15055 ss-10] | chr11_AA_01033 | glycoside hydrolase family 51 protein [Cylindrobasidium torrendii FP15055 ss-10] | 0.267707507 |
| chr06_AA_00738 | pyridoxal phosphate-dependent enzyme, beta subunit [Cylindrobasidium torrendii FP15055 ss-10] | chr11_AA_01042 | acetoin reductase family protein [Cylindrobasidium torrendii FP15055 ss-10] | 0.184003532 |
| chr06_AA_00738 | pyridoxal phosphate-dependent enzyme, beta subunit [Cylindrobasidium torrendii FP15055 ss-10] | chr11_AA_01046 | hypothetical protein CYLTODRAFT_428160 [Cylindrobasidium torrendii FP15055 ss-10] | 0.215292987 |
| chr06_AA_00738 | pyridoxal phosphate-dependent enzyme, beta subunit [Cylindrobasidium torrendii FP15055 ss-10] | chr11_AA_01097 | glycoside hydrolase family 3 protein [Cylindrobasidium torrendii FP15055 ss-10] | 0.15337217 |
| chr06_AA_00738 | pyridoxal phosphate-dependent enzyme, beta subunit [Cylindrobasidium torrendii FP15055 ss-10] | chr11_AA_01106 | related to stomatin [Armillaria ostoyae] | 0.198648103 |
| chr06_AA_00738 | pyridoxal phosphate-dependent enzyme, beta subunit [Cylindrobasidium torrendii FP15055 ss-10] | chr11_AA_01313 | hypothetical protein Hypma_000372 [Hypsizygus marmoreus] | 0.27075897 |
| chr06_AA_00738 | pyridoxal phosphate-dependent enzyme, beta subunit [Cylindrobasidium torrendii FP15055 ss-10] | chr11_AA_01324 | terpenoid synthase [Cylindrobasidium torrendii FP15055 ss-10] | 0.214480384 |
| chr06_AA_00738 | pyridoxal phosphate-dependent enzyme, beta subunit [Cylindrobasidium torrendii FP15055 ss-10] | chr11_AA_01456 | uncharacterized protein ARMOST_16541 [Armillaria ostoyae] | 0.224264475 |
| chr06_AA_00738 | pyridoxal phosphate-dependent enzyme, beta subunit [Cylindrobasidium torrendii FP15055 ss-10] | chr11_AA_01461 | ricin B-like lectin [Armillaria gallica] | 0.211196225 |
| chr06_AA_00738 | pyridoxal phosphate-dependent enzyme, beta subunit [Cylindrobasidium torrendii FP15055 ss-10] | chr11_AA_01510 | hypothetical protein ARMGADRAFT_1015707 [Armillaria gallica] | 0.110517164 |
| chr06_AA_00738 | pyridoxal phosphate-dependent enzyme, beta subunit [Cylindrobasidium torrendii FP15055 ss-10] | chr11_AA_01636 | hypothetical protein CYLTODRAFT_421880 [Cylindrobasidium torrendii FP15055 ss-10] | 0.232082494 |
| chr06_AA_00738 | pyridoxal phosphate-dependent enzyme, beta subunit [Cylindrobasidium torrendii FP15055 ss-10] | chr11_AA_01644 | hypothetical protein CYLTODRAFT_438918 [Cylindrobasidium torrendii FP15055 ss-10] | 0.199346971 |
| chr06_AA_00738 | pyridoxal phosphate-dependent enzyme, beta subunit [Cylindrobasidium torrendii FP15055 ss-10] | chr11_AA_01664 | hypothetical protein CYLTODRAFT_421876 [Cylindrobasidium torrendii FP15055 ss-10] | 0.178434434 |
| chr06_AA_00738 | pyridoxal phosphate-dependent enzyme, beta subunit [Cylindrobasidium torrendii FP15055 ss-10] | chr11_AA_01685 | 1-aminocyclopropane-1-carboxylate deaminase [Armillaria solidipes] | 0.193365344 |
| chr06_AA_00738 | pyridoxal phosphate-dependent enzyme, beta subunit [Cylindrobasidium torrendii FP15055 ss-10] | chr11_AA_01686 | hypothetical protein CYLTODRAFT_348076 [Cylindrobasidium torrendii FP15055 ss-10] | 0.219769794 |
| chr06_AA_00738 | pyridoxal phosphate-dependent enzyme, beta subunit [Cylindrobasidium torrendii FP15055 ss-10] | chr11_AA_01711 | hypothetical protein ARMGADRAFT_1046867 [Armillaria gallica] | 0.279578751 |
| chr06_AA_00738 | pyridoxal phosphate-dependent enzyme, beta subunit [Cylindrobasidium torrendii FP15055 ss-10] | chr11_AA_01731 | benzoquinone reductase [Cylindrobasidium torrendii FP15055 ss-10] | 0.157075017 |
| chr06_AA_00738 | pyridoxal phosphate-dependent enzyme, beta subunit [Cylindrobasidium torrendii FP15055 ss-10] | chr11_AA_01732 | glycoside hydrolase family 76 protein [Cylindrobasidium torrendii FP15055 ss-10] | 0.208591244 |
| chr06_AA_00738 | pyridoxal phosphate-dependent enzyme, beta subunit [Cylindrobasidium torrendii FP15055 ss-10] | chr11_AA_01733 | glycoside hydrolase family 76 protein [Cylindrobasidium torrendii FP15055 ss-10] | 0.212078557 |

Gene1 represent hub nodes while gene2 represent associated nodes in the co-expression network. The value of weight represents the correlation between two genes in a edge.

Supplementary Table S7. Information of 19 stress response related genes

| Gene ID | Gene annotation | log2(fold change） |
| --- | --- | --- |
| chr09_AA_00190 | exo-beta-1,3-glucanase | 5.18 |
| chr09_AA_00191 | exo-beta-1,3-glucanase | 4.71 |
| chr06_AA_00433 | exo-beta-1,3-glucanase | 6.02 |
| chr11_AA_00595 | chitin deacetylase | 3.51 |
| chr08_AA_00984 | cytochrome P450 | 4.32 |
| chr07_AA_00464 | cytochrome P450 | 2.16 |
| chr08_AA_00856 | cytochrome p450 | 1.59 |
| chr07_AA_01008 | cytochrome p450 | 1.53 |
| chr09_AA_00838 | cytochrome P450 | 1.92 |
| chr08_AA_00858 | cytochrome P450 | 2.50 |
| chr09_AA_01114 | glutathione S-transferase | 3.26 |
| chr08_AA_00687 | Clavaminate synthase-like protein | 1.42 |
| chr11_AA_00504 | MFS general substrate transporter | 1.57 |
| chr08_AA_01046 | MFS general substrate transporter | 3.78 |
| chr08_AA_00936 | MFS general substrate transporter | 1.52 |
| chr07_AA_01051 | MFS general substrate transporter | 2.53 |
| chr07_AA_00414 | MFS general substrate transporter | 2.51 |
| chr08_AA_01259 | Aldo/keto reductase | 1.88 |
| chr10_AA_00996 | tyrosinase | 1.55 |
